# Theme-centered interaction and developmental tasks as research method and pedagogical tool regarding identity development in VET

**DOI:** 10.3389/fpsyg.2023.1201305

**Published:** 2023-10-10

**Authors:** Christiane Thole

**Affiliations:** Faculty of Education, Universität Hamburg, Hamburg, Germany

**Keywords:** vocational identity, qualitative research, theme-centered interaction, developmental tasks, case studies, design based research, mixed methods

## Abstract

This paper presents the methodology of a PhD project on identity development in German dual track VET. The mixed methods approach comprises theory-driven longitudinal case studies using qualitative and quantitative data and a document analysis of curricula. The study was part of a larger design-based research project and aimed to evaluate a newly developed and implemented VET curriculum for the retail sector from the learners’ point of view. The new curriculum contained a special competence dimension to foster vocational identity development and a major interest was to investigate the extent to which the curriculum succeeded to do so. The paper will start with a summary and assessment of limitations and advantages of research designs applied in different existing studies examining identity development. The author will then outline identity-relevant theories and approaches that proved to be useful for her research interest. Subsequently, she will describe the resulting design for collecting and analyzing her data. The core result consists of a theme-centered process analysis visualizing the individual state of developmental tasks. With the presentation of three exemplary cases she will illustrate how her approach allowed her to deeply understand the identity of the respective person and at the same time generate general insights about identity development of learners in VET. Finally, strengths and limitations of her approach will be discussed. This research approach is also suited to supporting pedagogical design, representing major added value.

## 1. Introduction

Faced with economic and ecological constraints, dynamic change, digitalization, crises etc., constructing a vocational identity has become a major developmental task for learners in VET. Employees’ and employers’ efforts to cope with a multitude of challenges bring forth increasingly subjectivized, individualized, and boundaryless vocational profiles and biographies (Beck, 1986; Unger, 2007; Kutscha, 2015) that entail active reflection, decisions, and actions by employees to shape their work and private life.

### 1.1. State of research

Although the number of studies on vocational identity development in VET is rising, evidence about this process is still limited. One reason is the lack of appropriate research methods that manage to grasp the complexity of identity development despite limited resources (Heinrichs et al., 2022). Research studying vocational identity development addresses courses at VET schools, academic courses, and workplace and career learning. An overview of selected studies (Supplementary material 1) shows a large variety of research designs and theoretical approaches. Some quantitative studies apply short questionnaires (Dick et al., 2008; Heinemann et al., 2009; Gammoh et al., 2014; Klotz et al., 2014; Berg, 2017). In terms of theory they focus on notions like identification,^1^ commitment, work satisfaction, and morale. Interestingly, Marcia (1966) famous assessment of adolescents’ identity status (Achievement, Diffusion, Moratorium, Foreclosure) is rarely applied. The studies rather refer to vocational identity as a social identity (Lave and Wenger, 1991; Tajfel and Turner, 2004) and analyze the person-environment-fit. Vocation-specific aspects, e.g., differences between manual trades and retailing, are often left out intentionally to be able to analyze shared phenomena or compare vocations (e.g., Heinemann et al., 2009; Klotz et al., 2014). These studies generate knowledge about these concepts and their interdependencies and differences among vocations but have difficulty explaining the findings. Quantitative studies seeking to generate more comprehensive evidence use other theoretical concepts like interdependencies between job characteristics and personality traits (Kohn and Schooler, 1982) or criteria influencing discrepancy and congruence experience (Heinzer and Reichenbach, 2013). They generate general insights about mediating factors, interdependence of variables, and universal characteristics of identity development, but do little to explain individuation processes and—as the aforementioned studies are cross-sectional—developmental aspects over time. In addition, underlying models and data analysis tend to become complex and require adequate resources.

As identity development is a subjective construct many researchers opt for a qualitative or mixed methods approach. Beck (2000), Lewalter et al. (2001), and Prenzel et al. (2001) use observations and interviews to validate their quantitative findings. Witzel and Kühn (1999), Grote and Raeder (2003), Haasler (2007), Raeder and Grote (2007), and Kutscha et al. (2009) combine the advantages of quantitative (larger more representative samples) and qualitative (deeper analysis) approaches. Homburg et al. (2009) carry out an extensive qualitative study to get insights required for constructing quantitative scales. Kaak et al. (2013) use a complex quantitative questionnaire based on a model derived from vocational choice theories (Super, 1957; Holland, 1985) to assess learners’ state of career choice competencies. For pedagogical purposes the authors recommend to combine the questionnaire with a comprehensive individual qualitative analysis (Driesel-Lange et al., 2010). The example shows that a mix of quantitative and qualitative approaches can produce an added value including pedagogical applications (Kelle and Erzberger, 2010). The theoretical approaches vary depending on the research focus. Studies interested in learning motivation apply the self-determination theory by Ryan and Deci (2000) based on basic psychological needs (Lewalter et al., 2001; Prenzel et al., 2001). This approach also proves to be useful for analyzing difficulties at the beginning of an apprenticeship (Lange, 2019). Kohlberg (1984) theory of moral development is applied and partially falsified by researchers interested in moral judgment (Hoff et al., 1991; Beck, 2000). Studies analyzing the subjective development of learners during VET courses prefer the concept of developmental tasks by Havighurst (1974), however their understanding of the concept varies. Gruschka (1985) and Haasler (2007) as well as Bremer and Haasler (2004) assume that vocational identity is closely linked with vocation-specific competence development. Therefore, they apply evaluation tasks testing competence development to infer that identity development is taking place in a presumed way. On the contrary, Hericks (2006), Kutscha et al. (2009), Casper-Kroll (2011), Lange (2019), and Thole (2021) are interested in the learners’ subjective experience and reconstruct social reality in an inductive way. Other qualitative studies use numerous concepts such as vocational awareness (Beck, 2019; Kirchknopf and Kögler, 2020), subjectification of work (Bühler, 2007), boundaryless careers (Kirpal et al., 2007), reflexive action (Billett, 2007), ecology of human development (Bronfenbrenner, 1979; Kutscha et al., 2009), biographical transitions (Nohl, 2006), the process model of identity development by Hausser (1995), Grote and Raeder (2003), Voswinkel and Korzekwa (2005), and Raeder and Grote (2007), the service profit chain (Homburg et al., 2009), or role play (Voswinkel and Korzekwa, 2005; Ashforth et al., 2008). Some studies are designed for a certain sector such as retail (Kutscha et al., 2009; Duemmler et al., 2017), automobile (Bremer and Haasler, 2004; Haasler, 2007), or childcare (Gruschka, 1985) and may require adaptation if transferred to other sectors. Only few studies follow a multi-perspective design including the point of view of management (Grote and Raeder, 2003; Voswinkel and Korzekwa, 2005; Kirpal et al., 2007; Homburg et al., 2009) although this approach stands to reason in an interactionist understanding of identity development. Despite a considerable amount of qualitative research and the subjectivity and complexity of the concept of identity, encompassing case studies are rare (e.g., Nohl, 2006; Billett, 2007; Lange, 2019). Most studies analyze typologies (e.g., Witzel and Kühn, 1999; Grote and Raeder, 2003; Bremer and Haasler, 2004; Bühler, 2007) or universal patterns (e.g., Kirpal et al., 2007; Kutscha et al., 2009; Duemmler et al., 2017; Kirchknopf and Kögler, 2020). In this paper the author suggests a case-study-based methodology that aims to reconstruct the respondents’ real life identity conflicts in a complex and holistic way as a means to develop useful theoretical concepts for pedagogical practice.

### 1.2. The DBR project Evanet EH

**Figure 1:**
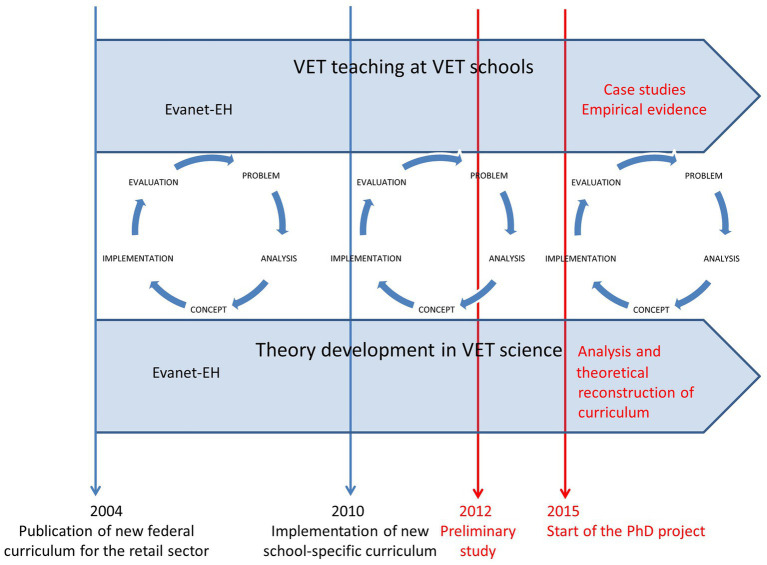
Design-based research process of curriculum development (Thole, 2021, p. 28).

The author’s PhD project (Thole, 2021) was a cycle of the design-based research (DBR) project *Evanet EH* (DBR Collective, 2003; Barab and Squire, 2004; Anderson and Shattuck, 2012) initiated by four vocational schools and a team of scholars led by her doctoral supervisor (Tramm et al., 2009; Supplementary material 2).^2^ The project’s goal was to implement a new curriculum according to new guidelines released by the *Conference of Ministers of Culture* (KMK, 2004/2016). The core idea of the DBR design was to improve VET practice and theory iteratively: While VET schools were interested in implementing and evaluating the new curriculum, Tramm et al. (2009) aimed to improve their curriculum theory to apply it to other contexts. The author had already carried out an evaluation from the teachers’ point of view in 2012 (Supplementary material 3). She had found that teachers appreciate the new curriculum as it facilitates teaching and teamwork, but learners’ development needed further clarification and support. Her assignment and intention for the PhD project was to generate a multi-perspectival understanding of the curriculum by evaluating it from the learners’ point of view (Figure 1).

**Figure 2:**
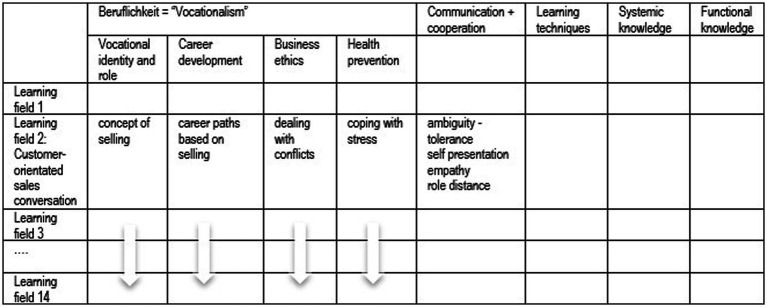
Learning field competence matrix—Evanet EH curriculum (Thole, 2021, p. 194).

She had been asked to focus on a competence dimension called *Beruflichkeit* (term corresponding to *vocational* self-concept) that had been included in the school-specific curriculum to foster vocational identity development. It is divided into four sub-dimensions called *vocational identity and role*, *career development*, *business ethics*, and *health prevention* (Figure 2; Thole, 2017). *Beruflichkeit* is one of five competence dimensions representing vocational action competence. The core idea is to develop the required competencies in a spiral curriculum. The latter consists of a sequence of so-called learning fields which represent typical complex work processes such as selling and purchasing. In previous cycles, scholars and practitioners had agreed on the content of *Beruflichkeit* by common sense but a theoretical justification was still missing. This was also the case for the assumption that the logic of the aspired development could be explained by means of the concept of developmental tasks (Havighurst, 1974; Krille et al., 2014). Furthermore, the curriculum guidelines (KMK, 2021) hardly mentioned identity development, but stated a mandate of Bildung^3^ and a range of target competences instead.

To prepare her research design, the author therefore first had to clarify her theoretical understanding of the concept of identity development. For this purpose she conducted literature research on core notions of the curriculum. This allowed her to reconstruct the curriculum content and to identify relevant categories for data collection and analysis. Given the state of research methodology it was also necessary to clarify the researcher’s paradigmatic point of view and assess suitable research methods. The following section outlines the results of this process.

## 2. Paradigmatic, methodological, and theoretical considerations

### 2.1. Paradigmatic positioning

Given the intention to explore respondents’ subjective point of view to design adequate didactics and in the face of the complex, constructivist, and subjective nature of the concept of identity, paradigmatic decisions were already implied: The aim was to reconstruct the subjective and social reality experienced by learners facing real life problems during the VET course. In this sense the approach is hermeneutic and dialectical. As evidence is generated to improve pedagogical practice and stimulate learners’ development in a desirable way it is explicitly normative. An approach fitting these features has been put forth by Holzkamp (1995) and was called *Subjektwissenschaftliche Grundlegung* (basics of subject-scientific learning theory). It is critical as it strives to enlarge learners’ scope of action for problem-solving and constructive as it aims to improve their life quality.

The paradigmatic decision also influences the research design and methodological choices. The DBR context proved to be ideal for this paradigmatic position: It made it possible to study learners in authentic real life settings and participants were interested in using empirical evidence to improve practice. In fact, curriculum guidelines stipulate an official mandate of Bildung for VET schools: The aim is to develop an overall vocational action competence not only for typical vocational situations, but also “*for participating and sustainably shaping society and the world of work in social, economic, ecological, and individual responsibility*” (KMK, 2021, p. 14, translated from German) which is a core issue of identity development (section 2.2). In addition, the project context facilitated access to the research field for longitudinal in-depth interviews and made it possible to adapt data collection instruments precisely to the research interest.

However, established research methods have to be assessed critically in the light of this research interest: Methods deriving from social sciences serve to examine social constructs. The individual case serves as both an *example* and a *means* for research. However, the individual case is the *end* in educational science. Therefore, pedagogy is interested in both: general knowledge about the population of learners as well as the holistic understanding of the individual case (Tiefel, 2005; Marotzki, 2010). The latter becomes indispensable in the face of heterogeneous classes to be prepared for a subjectivized world of work that demands individualized didactics. Another important issue is the *end* of the study. Pedagogical evidence produced in DBR projects serves to design adequate pedagogical approaches while social studies do not necessarily aim to change the research field. Among the studies mentioned in section 1.1, only Kaak et al. (2013) questionnaire and model were designed to be applied in schools. Other studies derive recommendations for VET practice (Heinemann et al., 2009; Kutscha et al., 2009; Duemmler et al., 2017) and the workplace (Grote and Raeder, 2003).

The requirement to individualize didactics and the complexity of identity development seem to predestine a qualitative approach. It enables the researcher to gather extensive information from the respondent and is thus helpful for clarifying inconsistencies and for understanding and explaining emerging phenomena. However, most qualitative research methods aim to gain cross-case or collective knowledge (Bohnsack, 2010, 2014), help interpreting quantitative data (Mayring, 2002, pp. 41f.), build theory, or reveal social structures and processes (Glaser and Strauss, 1967). For instance, *Qualitative Content Analysis* (Mayring, 2000) which is often applied in educational studies, literally disassembles a case into categories. Identity development is the opposite: establishing coherence among disparate elements. The documentary method (Bohnsack, 2010, 2014) elucidates implicit collective knowledge and is thus suited to discovering indicators for social identity, but it does not lead to an understanding of individuation. *Grounded Theory* (Glaser and Strauss, 1967) would theoretically be suitable, but needs adaptation when applied to an educational context involving subjective meaning and individual development (Tiefel, 2005). Biographical research has developed a variety of methods analyzing curricula vitae (Nohl, 2006; Marotzki, 2010) that allow us to understand crucial life passages and individuation, but these are not designed to analyze situational identity balance (Goffman, 1959; Krappmann, 1969). Objective hermeneutics (Oevermann et al., 1987; Bohnsack, 2014, pp. 71–84) are suitable for discovering singularities and inconsistencies, but the method is laborious. Given these strengths and limitations a triangulation of several concepts and methodologies is required to get a holistic picture of an individual’s identity (Eisenhardt, 1989; Flick, 2010).

### 2.2. The theoretical concept of identity development

Another major challenge for research on identity development is defining the subject matter as a prerequisite for methodological decisions. In fact, there are a multitude of identity theories. Fortunately, this does not necessarily indicate disagreements on the notion itself, but rather reflects the complexity of the concept and different foci (Frey and Haußer, 1987; Keupp et al., 2013). The core common denominator is that people reflect continuously on their self and their relationship with their environment. This satisfies anthropological needs for *coherence*, *continuity*, *individuality*, and *meaning* (Mead, 1934; Frey and Haußer, 1987; Giddens, 1991; Keupp et al., 2013; Thole, 2021, p. 37ff.). Furthermore, a text analysis reveals that the mandate of Bildung in the KMK guidelines and Humboldt (1792) concept of Bildung are very close to this notion of identity as reflected in contemporary literature (Thole, 2021, p. 177ff.).

**Figure 3:**
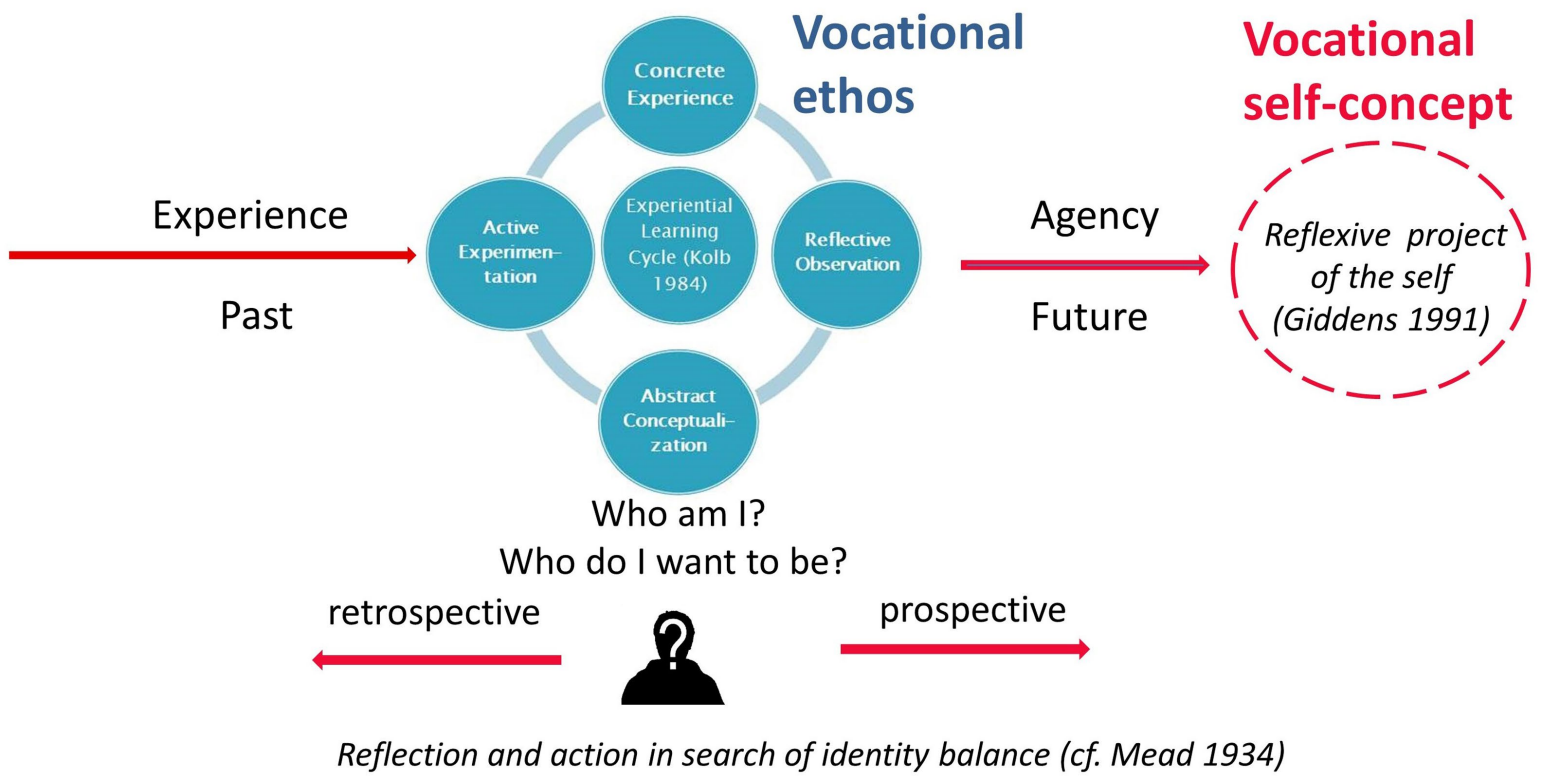
Diachronic and synchronic perspective of identity development (Thole, 2021, p. 285).

**Figure 4:**
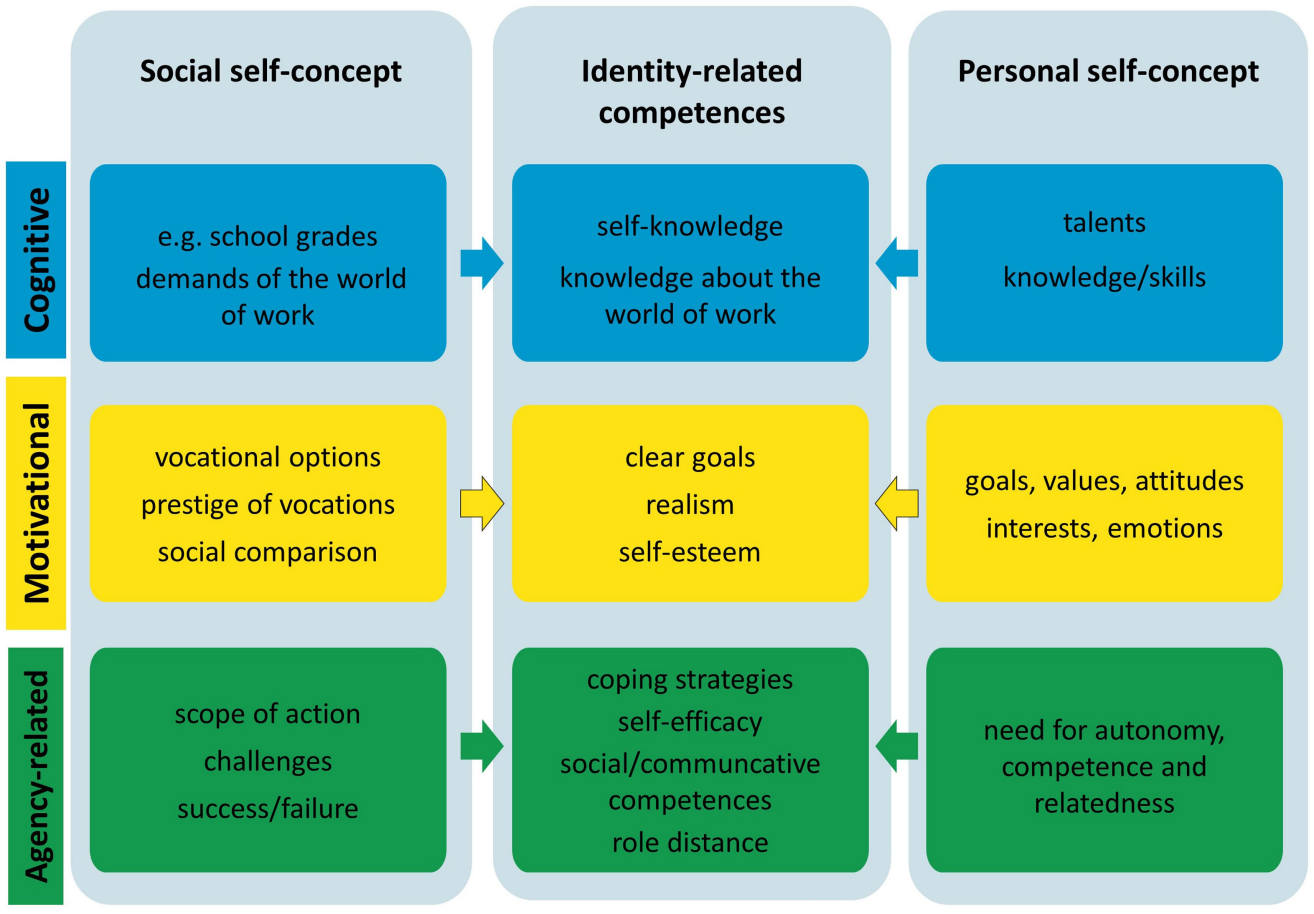
Identity balance in social interaction (Thole, 2021, p. 282).

Figures 3, 4 visualize a synthesis of core findings of the literature research. Figure 3 illustrates the process of identity development in a holistic way: Individuals evaluate their experience *retrospectively* to find out who they are and what they want to be (Giddens, 1991; Hausser, 1995). This serves to project their current self-concept^4^ *prospectively* into the future to set goals for their personal development (Mollenhauer, 1983; Giddens, 1991). This *diachronic* aspect of identity development relates to the sub-dimension *career development*. During a situation that requires action the reflective activity follows the typical stages of an experiential learning cycle (Kolb, 1984): concrete experience, reflective observation, abstract conceptualization, and active experimentation (center of Figure 3). This *synchronic* aspect of identity development (Mead, 1934; Goffman, 1959; Krappmann, 1969) relates to the sub-dimensions *vocational identity and role*, *business ethics*, and *mental health*. Vocational identity is a decisive component of the overall identity because professional prospects determine income, status, and social milieu (Super, 1957; Beck, 1986; Gini, 1998). It becomes necessary to work on one’s identity when disparities arise between personal aspirations and social demands. In a vocational context, this is especially the case during transitions in work biographies (Busshoff, 2001; Nohl, 2006; Marotzki, 2010) or when employees face conflicts at their workplace (Voswinkel and Korzekwa, 2005; Ashforth et al., 2008). In complex and rapidly changing job markets individuals’ vocational identities are challenged regularly.

Symbolic interactionism (Mead, 1934; Krappmann, 1969) assumes that such social interactions stimulate identity development. Figure 4 visualizes what happens in case of conflicts: individuals have to mediate between their social concept, which aims to meet and internalize societal demands (left column), and the inner personal self-concept, which comprises the person’s interests, values, and aspirations (right column). To achieve personal fulfillment, individuals strive to reconcile both parts of the self-concept. However, in a world characterized by fluid boundaries, contradictions, uncertainty, ambiguities, ambivalence, and multiple options, it becomes harder to match social and personal self-concept and to establish coherence among areas of life (Keupp et al., 2013). Therefore, a set of competences is required (see middle column) (Super, 1957; Hausser, 1995; Driesel-Lange et al., 2010). The cognitive dimension comprises self-knowledge and knowledge about the world of work; the motivational dimension involves goal-setting, realism, and self-esteem; while the action-orientated dimension includes self-regulation, resilience, and coping strategies. In addition, individuals need a set of social and communicative competences (Krappmann, 1969; Veith, 2010) to balance their self-concept in social interaction. Social and personal self-concept both derive partially from belonging to certain groups (e.g., community of practice, religion, nationality). These elements are called *social identity* (Tajfel and Turner, 2004), while the aspects that reflect the individuality of the person form the *personal identity* (Goffman, 1963, pp. 51ff.)

Against the background of this model, the different methodological approaches cited in section 1.1 can be contextualized. Approaches focusing on identification measure the quality of matching of social and personal self-concept at a certain point of time for certain aspects, but do not explain the causes. Kaak et al. (2013) questionnaire analyzes the career learning competencies in the middle column in the figure. The concept of developmental tasks assumes that at a certain stage of life, e.g., the beginning of an apprenticeship, large gaps between the current personal self-concept and the desired vocational social self-concept appear and that these need to be reduced and balanced during the VET course. Approaches analyzing boundaryless careers and subjectivized work focus the individualized vocational self-concept (personal identity) while most of the quantitative studies refer to the social identity that results from belonging to a community of practice. The analysis shows that most studies focus on certain isolated aspects. However, this impedes an in-depth understanding of individual cases. Therefore, the author strove for a more holistic approach. This required a triangulation of theoretical concepts.

### 2.3. Triangulation of theoretical concepts

To gain a holistic impression of a case, theoretical categories should cover synchronic and diachronic aspects, personal and social identity as well as related competences. As resources are restricted, it will rarely be feasible to analyze all aspects in-depth in a cross-case perspective, but it makes sense to collect a broad range of data as it is not known in advance which aspects will be relevant for the interpretation of cases.

First of all, as identity development is a reflexive activity, *self-reflection* is a crucial prerequisite. However, studies (Daudert, 2001; Fonagy et al., 2016) show that people differ in this respect and that there is a close correlation with bonding experience in early childhood. Resources will usually not be sufficient to apply Fonagy et al. (2016) *Reflective Functioning Questionnaire* but it offers useful criteria to trace reflection skills in respondents’ biographies and ways of arguing.

Assuming that self-reflection is not impaired, individuals can be considered to be experts in their own identity. The reported *self-concept* comprises a large variety of aspects. Figures 3, 4 show useful categories. To collect extensive information the respondents need to be stimulated to speak about themselves, their workplace, and other relevant environments. To grasp synchronic and diachronic aspects it is important to address biographical issues as well as challenging situations. As the reported self-image is subjective it is also crucial to identify passages that indicate discrepancies or congruence with the perceptions of others. If the design does not allow to interview relevant others the respondents can nonetheless be asked about feedback received from peers, family, and colleagues or to put themselves in third parties’ position. Asking about personal strengths, weaknesses, interests, values, and preferences will generate relevant features of the respondent’s *personal* identity. For many of the aspects included in Figure 3 quantitative scales do exist and can be combined with qualitative approaches, e.g., self-efficacy scales (Schwarzer and Jerusalem, 1999), resilience (Schumacher et al., 2005), self-esteem (von Collani and Herzberg, 2003), values (Lindeman and Verkasalo, 2005), ambiguity tolerance (Lind, 1987), emotional competence (Rindermann, 2009), and self-concept dimensions (Deusinger, 1986). Some exist in self-assessment and third party versions (e.g., Deusinger, 1986; Rindermann, 2009) which can help to discover (mis-)matches.

Bronfenbrenner (1979) theory about the ecology of human development provides valuable categories for studying the *person-environment fit* from a pedagogical point of view. He spells out conditions in the learning environment that facilitate personal development. He identifies the following factors: a broad role repertoire, communication and compatibility among life areas, trustful dyadic relations with mentors, and subjective assessments. The following case from the author’s study can illustrate what this means in practice: Ahmet (Thole, 2021, pp. 453–456) reports that he is obliged to do nothing but cleaning (restricted role repertoire). He is regularly in conflict with his superior, nobody shows him how to fulfill his tasks (no mentoring). It is almost impossible to reconcile expected working hours, school attendance, and exam preparation (no compatibility and communication among life areas). So none of the mentioned conditions is fulfilled but nevertheless Ahmet regards his apprenticeship as a springboard for further education and is therefore determined to finish it (subjective meaning). He uses all existing possibilities (e.g., refusing additional working hours, sick notes, leave for exam preparation) to achieve his goal. Thanks to Ahmet’s meaningful interpretation of the situation, he is able to use it as opportunity, although in a defensive way (Holzkamp, 1995). Learners with a negative subjective meaning would be likely to drop out under such conditions.

There is evidence that individuals’ social background also has a decisive impact on their educational and vocational career and aspirations (e.g., Maaz et al., 2011). Bourdieu’s habitus theory (Bourdieu, 2012) can explain this, as the chances to get a recognized place in society (symbolic capital) are influenced by relations (social capital), monetary resources (economic capital), and education (cultural capital). Therefore it makes sense to interpret Ahmet’s situation reported above in the light of his social background. He describes how he starts his vocational career from a disadvantaged position without social and economic capital:

*“Until I was 20, I wasn’t ambitious. I did not have a person who told me: do an apprenticeship.”* (Thole, 2021, Ahmet I, 32)

*“As long as I can remember I have been living on social benefits. […] My father has never been there, he didn’t give any support.”* (Thole, 2021, pp. 50–56)

*Motivational aspects* are another important issue as motivation is the force for action and closely related to interests, which are a precursor of identification (Lewalter et al., 2001). According to Ryan and Deci (2000) motivation derives from the satisfaction of basic psychological needs for achievement, autonomy, and relatedness. The significance of these needs differs between individuals and leads to different rank orders of life goals that influence a person’s decisions, vocational ethos, and self-concept. It is therefore productive to ask about major interests, values, and life goals, and their ranking.

As identity needs to be balanced in social interaction, relevant *social and communicative competences* also need to be recorded. According to Krappmann (1969) the following skills are crucial: self-presentation, ambiguity tolerance, empathy, and role distance. Veith (2010) has modernized and enlarged this set: As social expectations connected to a role and social norms tend to become more diverse, he states, social perspectives need to be co-constructed, and a self has to be performed in a creative way. In addition, individual norms and values influencing action must be reflected. The competences can be inferred from the way the respondents present themselves and their statements about social interactions. Kohlberg (1984) offers a theory to classify the types of moral reasoning in dilemma situations and to compare them to other cases. Lind (1985)^5^ developed a tool based on Kohlberg’s theory to assess moral arguments in a structured and differentiated way.

Reflecting on one’s identity is an integral part of agency (Simons, 2021). In a world of work characterized by a multitude of challenges it is therefore crucial to have functional *coping strategies* (Welbourne et al., 2007; Böhnisch et al., 2009; Rees et al., 2015) and thus a well-balanced identity. Using concepts like ambiguity tolerance, role-distance, emotional competence, self-efficacy, self-esteem, attribution style, resilience, or certain self-concept scales allows us to describe a person’s coping style and to find differentiated causes why individuals follow either defensive strategies that meet others’ demands or expansive coping strategies that try to enlarge their own scope of action (Holzkamp, 1995; Thole, 2018).

Again, one of the author’s case studies can show how a coping style can be analyzed. Markus reports:

Attribution style: *“I did not get any feedback during my apprenticeship—especially during the probation period. There was no communication between me and my superior.”* (Thole, 2021, Markus I, 30)

Resilience/self-esteem: *“They wanted to sack me after probation period. […] Now I am the best salesperson in the ranking of apprentices.”* (Thole, 2021, Markus I, 24)

Lack of resilience: *“And then when I had to write my reports, I could not express anything. I don’t know, I collapsed.”* (Thole, 2021, Markus II, 2)

Expansive/defensive style: *“And then I resigned—without notice as my superior requested. […] And then I was immediately off the payroll and my public transport season ticket was cancelled.”* (Thole, 2021, Markus II, 2)

As a result of a series of successful experiences Markus knows his strengths and talents. But success alternates with serious setbacks (illness, threat of dismissal, mental breakdown). His preferred coping strategies are perfectionism and meeting others’ demands in a defensive way. As he fails to reflect his interests and to attend to his mental condition these strategies are dysfunctional and provoke new setbacks.

*Developmental tasks* were a core research interest of the reported DBR project and are also generally interesting for pedagogical purposes. The notion conceptualizes certain periods in biographies that are characterized by a gap between social and personal self-concept caused by societal demands and/or personal aspirations (Havighurst, 1974). These tasks also require intense (synchronic and diachronic) identity-related reflection and action and lead to individual developmental goals that can become the starting point of individualized didactics. Havighurst (1974) identifies eight developmental tasks for adolescence—among others preparing for an economic career, marriage, and family life; emotional independence from parents, and the acquisition of an ethical system. Research studying VET courses usually relates to the first task and breaks it down into sub-tasks (Gruschka, 1985; Bremer and Haasler, 2004; Hericks, 2006). The concept of developmental tasks can be used constructively by including them intentionally in a curriculum. Research then aims to evaluate to what extent the real subjective development follows the intended objective model. On the other hand, developmental tasks can be considered a social reality that needs to be reconstructed. Depending on the way of interpreting developmental tasks research methods vary. The author applied methods of reconstructive social research (Oevermann et al., 1987; Tiefel, 2005; Nohl, 2006; Bohnsack, 2010, 2014) as well as quantitative scales. This triangulation of methodologies will be presented in the following section.

### 2.4. Triangulation of methodologies

There are several reasons to use a mixed methods approach. It makes sense to combine not only qualitative and quantitative approaches but also different qualitative methodologies: Qualitative interpretations risk being subjective and depending on the researcher’s point of view and previous knowledge (Meinefeld, 2010). Including quantitative data creates an opportunity to validate qualitative results with objective, reliable, and valid information, helps to cover missing aspects, and facilitates case comparisons due to existing standard score norms. Furthermore, depending on the choice of methodology qualitative designs may leave out crucial aspects (section 2.1). Each approach has a core idea and special strengths. This enables the researcher to use different methodologies like a selection of pairs of glasses. Depending on which glasses the researcher wears, he/she will discover different aspects in the data and peculiarities that would have been overlooked by other approaches. For this purpose, it is not necessary to analyze all the data with all the methods in the minutest detail, but it does make sense to ask whether a particular methodology is likely to generate a deeper understanding of a case or phenomenon. Triangulating different approaches produces a spectrum of findings that together form a mosaic that sheds light on a person’s identity. Some approaches that can produce interesting information will be presented below. Their added value will be illustrated with examples from case studies.

#### 2.4.1. Objective hermeneutics: discovering singularity and (in-)coherence

As outlined in section 2.2, coherence between a multitude of aspects and areas of life is a crucial function of identity balance. *Objective hermeneutics* (Oevermann et al., 1987) can help to analyze the quality of coherence and discover singularities. One of the core ideas is a sequential comparison of expectable and actual statements depending on the context. The following example will demonstrate how this can produce interesting insights.

Hauke’s way of commenting experienced violence as a toddler is surprising as he shows no empathy for the little child (himself). Instead, he takes his mother’s perspective and does not mention any crying or pain. The incident was supposedly of no concern for him although it obviously was for his mother.

*“Were there any critical incidents in your life?”* H*: “I have to remember* […] *Oh yes. This story was often retold; my Mum still feels ashamed. I was four or five years old. I was a petulant child* […] *I was screaming, trampling, and then my mum lost her temper, took a wooden spoon and spanked my ass. Then I was quiet. And then my behavior improved. That was very early in my life. Retrospectively I think it did not matter.* […] *I told her: Mum, it’s not so bad.”* (Thole, 2021, Hauke Ia, 4–8)

There is another striking statement about his job choice:

*“For 5000 € I would clean toilets.* […] *I would not mind even if others would laugh at me.”* (Thole, 2021, Hauke Ib, 68–70)

Immediately before this he had reported:

*“I would say with the experience I have got,* it [retail in consumer electronics] *unites two things that I am experienced in, thus two competences. Deal somehow with other people, approach them and help other people, and the technical domain. Yes, that fits, that suits me.* […] *I like it and I can imagine it theoretically, but I would prefer to improve my skills.”* (Thole, 2021)

In other passages in the interview Hauke describes himself as a rational, patient, diplomatic, and empathetic person (Thole, 2021, Hauke Ia, 38–40). Many examples from his private and work life, including the two cited above, validate this self-assessment. However, his self-reflection is contradictory. On the one hand, competence and recognition are important to him, on the other hand he denies this. He shows empathy for others, but not for himself. His exposure to violence as a child may have had a traumatic effect (Streeck-Fischer, 2014). As harmony is a major value for him, he denies his self. As a result, he has difficulty pursuing his interests. For instance, he reports feeling afraid of acquisition and self-marketing.

#### 2.4.2. Documentary method: revealing social identity

By means of vocations individuals become members of a community of practice and acquire a recognized place in society that is associated with a certain social status. Their qualifications and vocational experience become part of their self-concept (Super, 1957; Beck and Brater, 1977). Thus, vocations are an important aspect of *social* identity (Heinrichs et al., 2022).

The latter can be inferred from the implicit knowledge that is typical of a particular social group. The documentary method has been designed to study this by systematic comparison of *how* something is said (Bohnsack, 2010, 2014). Even individuals who do not know each other share common knowledge, e.g., because they belong to the same generation or hold the same vocational role (Nohl, 2006). The following example illustrates that statements imply a lot more than what is literally said:

Using the we form, Jennifer shows she identifies with the commercial reasoning of the management and colleagues:

*“We do not give price guarantees any more. Most products we sell are loss-making anyway.* […] *Although we sell less products now because it is more expensive, we earn more now.”* (Thole, 2021, Jennifer II, 22)

As a salesperson, she thinks in terms of margins, not consumer selling prices like her peers would. Her statement is also a document of the struggles of the bricks-and-mortar retail sector and implies that only certain customers fit the employer’s business model.

Another example shows that the role as apprentice implicates shared developmental tasks. These tasks are not necessarily explicitly formulated, but young people have an implicit knowledge about societal demands and expectations. Therefore they can be considered part of apprentices’ social identity.

Alina’s and Ahmet’s backgrounds are different (the former a middle class college dropout employed in telecommunications sales, the latter a lower class welfare recipient employed in a filling station), but they are working on the same developmental tasks: to find a vocation that fits their personal preferences and to search for a recognized place in the world of work.

*“I always liked being in contact with others. And then I decided to choose a commercial apprenticeship that allows me to be in contact with customers rather than sitting in an office.”* (Thole, 2021, Alina II, 55)

*“And then I told myself: ‘Okay, what do you want? Where do you want to go? I am not talented in handcraft.’ And then I thought: ‘I prefer the commercial domain.’”* (Thole, 2021, Ahmet I, 32)

*“You start as a junior salesperson and then you can move up and specialize.”* (Thole, 2021, Alina II, 22-32)

*“I feel it is a stigma to work at a filling station.* […] *It is really the pits.”* (Thole, 2021, Ahmet II, 60)

It is striking that Ahmet and Alina “play” the same vocational role despite their different social backgrounds.

#### 2.4.3. Grounded theory: analyzing biographical learning

As Figure 3 shows, identity development is an experiential learning process (Dewey, 1938; Kolb, 1984) incurred by reflection on the self in action. By analyzing and assessing present and previous action, a meaningful and authentic thread of self-actualization is constructed (Giddens, 1991, p. 78). Depending on the quality of the experience and subjective prerequisites, experience can foster or suppress personal deployment (Dewey, 1938, pp. 33ff.; Lazarus and Folkman, 1987). Tiefel (2005) developed an adapted coding paradigm based on *Grounded Theory* (Glaser and Strauss, 1967) to analyze these processes for pedagogical purposes. She focusses on the *meaning* constructed by the learners, the *structure between the subject and his/her environment*, and *patterns of agency*. Tiefel’s approach includes categories developed by biographical research such as *planned action schemes*, *contingent pathways*, and institutionally induced *life stages* (Schütze, 1983; Nohl, 2006, p. 20f.).

These approaches are here applied to the example of Alina who gave up her studies in product design:

Structure: *“Growing up in a large family has had a positive on my life* […].” (Thole, 2021, Alina II, 4)

Meaning: *“Family is very important for me …”* (Thole, 2021, Alina II, 4) […] *“Friends, family, my boyfriend of course. That is what it’s all about here.”* (Thole, 2021, Alina II, 69)

Agency/contingent pathways: “[…] *then I realized that I want to have a reliable source of income, and not have to worry.”* (Thole, 2021, Alina II, 51)

Agency/planned action scheme: *“And then I strive for being taken on after the apprenticeship. There are several career pathways.”* (Thole, 2021, Alina II, 6)

Alina has an intense need for security and relatedness. She tries to control her career path and avoids unforeseen risks and uncertainties. Therefore, she is actively looking for safe havens in work life. Remaining close to her family, partner and friends is more important to her than pursuing her vocational career. Work is rather a means to enable her to enjoy her private life.

Tiefel (2005) draws mainly on the methodology of biographical research, but concepts like coping, habitus or ecology of human development (section 2.3) can also be helpful for exploring patterns of agency or the structure of the person-environment fit.

#### 2.4.4. Quantitative scales

As laid out in section 2.3 numerous scales are available. In the present study the self-concept scales by Deusinger (1986) and Rindermann (2009) questionnaire on emotional competence have produced valuable insights for triangulation. The scales describe and compare relevant attitudes in a standardized way. They are multidimensional as they refer to different features of the person.

Deusinger (1986) self-concept scales were developed to create a reliable differentiated tool to measure transformations of self-concept after interventions. Based on research on attitudes she constructed independent scales capturing 10 different aspects of the self-concept referring to achievement, self-worth, and emotional and psychosocial dispositions.

Emotions indicate how we experience and assess certain situations. Although emotions like anger, fear, or joy are anthropologically universal, individuals react differently to the same stimuli due to previous experience (Lazarus and Folkman, 1987; Ekman, 2007). Emotions can be a useful indicator of the assessment of a situation, e.g., as a warning, but they can also be inadequate, e.g., as result of a trauma. That is why emotions require attentiveness, assessment, and regulation to be able to act adequately. In addition, the counterpart’s emotions also influence social interactions and need to be taken into account (Hacker, 2009). These can be inferred from facial expressions and nonverbal signals (Ekman, 2007). As a result, developing vocational action competence requires a person to perceive and regulate their own feelings and those of others. Rindermann (2009) measures this competence by means of five scales: RE (regulating own emotions), EE (perceiving own emotions), RA (regulate others’ emotions), EA (perceiving others’ emotions), and Exp (expressing own emotions). Awareness and regulation of others’ emotions can also be seen as an indicator of empathy and co-construction of social perspectives in the sense described by Krappmann (1969) and Veith (2010).

The list of possible methodologies is not exhausted, but the reported approaches allow a wide range of crucial features of identity development to be covered. The major challenge consists in applying them in an efficient manner given restricted resources. The following section will lay out how the author proceeded.

## 3. Methods

To achieve her paradigmatic goals (section 2.1), the author opted for qualitative longitudinal case studies (Eisenhardt, 1989). This would allow her to analyze cases in-depth including their development during the VET course, but also to identify cross-case patterns and build theory simultaneously. She performed the following steps suggested by Eisenhardt (1989).

### 3.1. Getting started

The first step consisted of preparations for a focused research procedure. This included paradigmatic considerations, the definition of the research question and the collection of theoretical constructs and methodological approaches that seemed suitable to ground the subject matter (section 2). The intention was *not* to choose a theory or find hypotheses, but on the contrary to maintain theoretical flexibility and to prepare data collection procedures (Eisenhardt, 1989). In the author’s case, the research question was the following: *How can identity work in VET courses enable learners to cope with challenges in the world of work?* Both the research question and the constructs have a tentative character at this early stage. Starting theory-building research with the ideal of *“no theory under consideration”* means keeping the process open without any preliminary decisions (Eisenhardt, 1989, p. 536).

Another important issue was the question of how to deal with the researcher’s previous knowledge. Biographical learning influences people’s assessments of situations, and this is also true of interpretations in a qualitative research process (Meinefeld, 2010). As it is not possible to lock out the researcher’s identity, objectivity was an unrealistic goal. In addition, the researcher’s identity could also be considered a resource for the research process, because her biographical experience would allow her to generate insights. The compromise was to include quantitative data (section 2.4) and to observe quality criteria (section 3.9) to increase intersubjectivity and disclose the author’s subjective interest and point of view regarding the research topic (Thole, 2021, pp. 44–47). To achieve less subjective results, Eisenhardt (1989) recommends using multiple investigators. In her PhD project, this was not possible, but the author discussed cases with students in seminars and with the persons who transcribed the interviews.

### 3.2. Selecting cases

The selection of the population and sample is an important step that serves to reduce extraneous variations, clarify the scope of the study, and make provisions that facilitate the emergence of relevant evidence despite restricted resources (Eisenhardt, 1989). In the author’s case, the population was retail VET learners at two VET schools in Hamburg. The curriculum had also been implemented in two other VET schools. In addition, the curriculum strategy has been applied to other vocations, e.g., office clerks in Berlin.^6^ This means that the findings would also be relevant for other federal states and vocations. In addition, the retail sector is well researched. This offered the possibility to validate the results. Furthermore, the retail sector has very high numbers of apprentices. Because of this, evidence concerning this sector is of high relevance for German VET, even if results are not transferable to other domains. In addition, the retail sector attracts a lot of disadvantaged young people who have no chance to enter other sectors. For this reason, it was probable to find a multitude of problems that would challenge learners’ identity. The goal of theoretical sampling was to choose cases which would allow to replicate, falsify or expand the emergent theory. For the author the most important selection criterion was involvement in the retail sector (e.g., filling station, food discount, consumer electronics, telecommunication, jewelry) and the size of the employer as it was known that conditions for apprentices are usually better in larger companies than in small ones (Beicht et al., 2009). In addition, filling stations and consumer electronic sales are polar opposites regarding prestige and professional competence demands. In addition, it was important to have participants from supermarkets as they represent about one third of the retail industry and working conditions there deviate significantly (BIBB and BAuA, 2012). In addition, the author made sure that learners with migrant backgrounds and both genders were represented in the sample (Supplementary material 5). Of 14 participants, only one dropped out of the study. Two further apprentices dropped out of the apprenticeship but took part in the second interview.

### 3.3. Crafting instruments and protocols

Eisenhardt (1989) emphasizes that a triangulation of multiple qualitative and quantitative data serves to strengthen the empirical evidence for theory development, fosters divergent perspectives, and may produce confirmative evidence, especially between quantitative and qualitative data. As the author had limited resources and restricted access to the research field, the challenge was to include the presented constructs in two interview guidelines. The interviews would take about an hour. The resulting guidelines (Supplementary material 4) included questions concerning social background, self-concept, career choice motives, life goals, typical vocational tasks, experienced conflicts and coping, expectations of superiors, customers and colleagues, a moral dilemma (Lind, 1985), perception of the curriculum at the VET school, and the following quantitative scales:

- Self-concept scales FSAP (problem solving), FSVE (decision making), FSKU (sociableness) and FSST (stableness toward groups and others) by Deusinger (1986). The first two scales are an integral part of action competence and the last ones are crucial for social interaction which is constitutive of identity development.
- Emotional competence scales (Rindermann, 2009)
- Ambiguity tolerance scale (Lind, 1987)

### 3.4. Entering the field

Eisenhardt (1989) states that it is typical of theory-generating research to overlap data collection and analysis and to adjust the research design when relevant themes or case features emerge. This flexibility serves to improve in-depth understanding of cases which is crucial for theory building. Eisenhardt (1989) argues this does not mean to be unsystematic, but rather pragmatic to understand unique cases. In the author’s research, this was the case for the construct of reflexivity. Ciara’s case (section 4.3.2) had drawn the author’s attention to differences in self-reflection styles and the corresponding research (Daudert, 2001; Fonagy et al., 2016). As a result, the reflexivity style was introduced to the coding scheme for data analysis (Supplementary material 6).

The author started with transcriptions and data analysis immediately after the first interviews. The first interview took place in the second half of the first year. This generated extensive insights about the starting period of the apprenticeship. The second survey was carried out during the second half of the second year, when some respondents had already prepared for exams of a two-year course or curtailed three-year course. The expectation was to discover transformations since the first interview and to gain validation of interpretations of the first interview. At the beginning of the study, the sample was continuously enlarged with new cases likely to contribute new insights. Conducting new interviews while analyzing the first ones enhanced the researcher’s awareness for cross-case patterns. After the first cycle of interviews, the research focus changed. Rather than analyzing each case in-depth and carrying out cross-case analysis for all constructs, the author concentrated on developmental tasks, curriculum analysis, and relevant literature, as she had discovered a fundamental negligence of subjective aspects in German VET’s understanding of action orientation (Thole, 2021, p. 190ff.).

### 3.5. Analyzing data

#### 3.5.1. Within-case analysis

According to Eisenhardt (1989) it is often difficult to find and describe analysis strategies that lead from large amounts of data to conclusions. She recommends detailed descriptive case-study write-ups to structure the data. The overall aim is to become intimately familiar with each case as a stand-alone entity. For within-case analysis, the author used a coding scheme based on the constructs described in section 3 (Supplementary material 6; Ciara’s example Supplementary material 7). To produce them, the interviews were transcribed in the software MAXQDA and coded with categories derived from the cited constructs (Supplementary material 8). Then, relevant information was manually transferred to the coding scheme.

#### 3.5.2. Cross-case patterns

**Figure 5:**
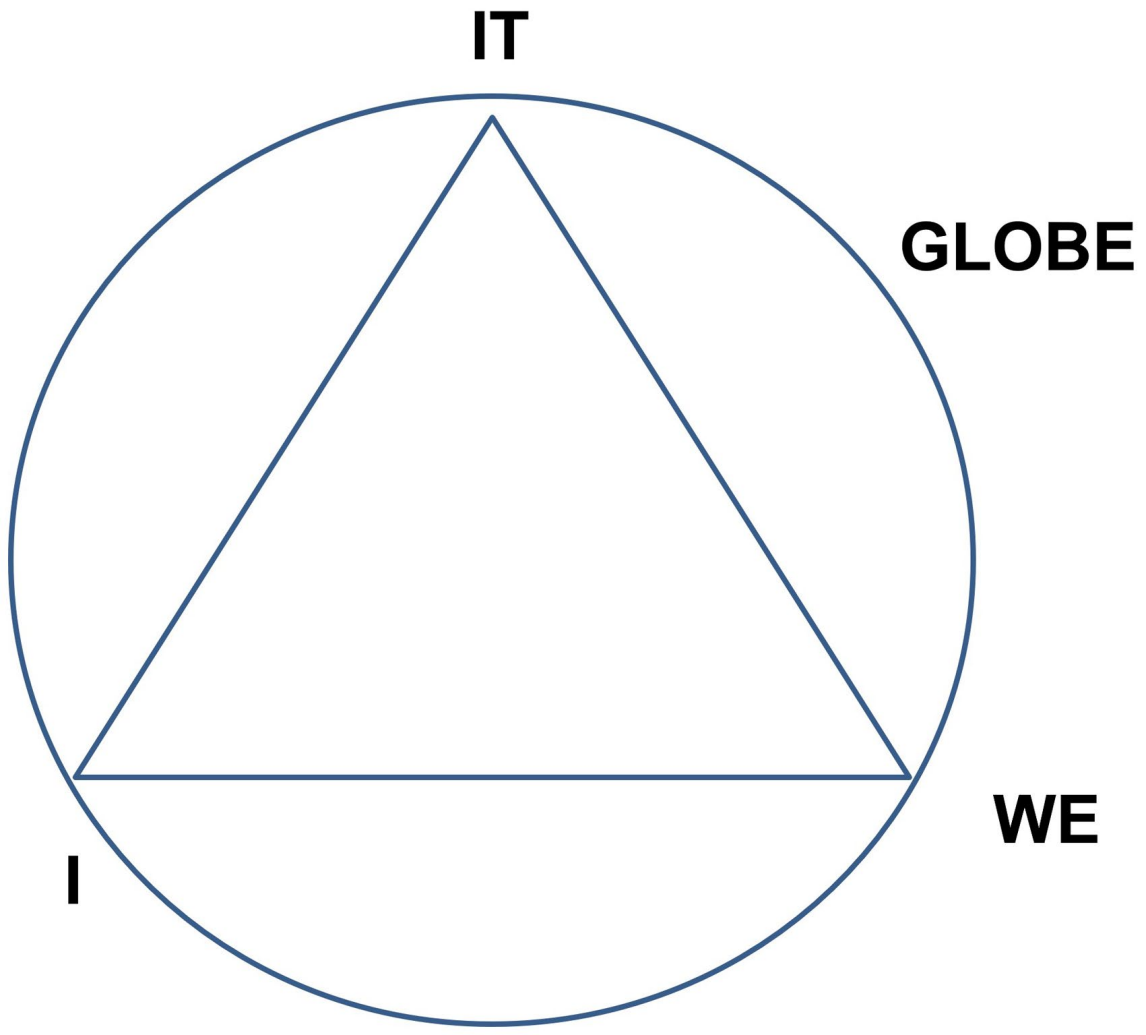
Four-factor-model of theme centered interaction (Schneider-Landolf et al., 2017, pp. 101ff.).

At a later stage, the author started to use visualization techniques based on *theme-centered process analysis* (Lotz, 2012) to condense major case features using the construct of developmental tasks. *Theme centered interaction* (TCI) is an approach developed by the psychoanalyst Cohn (1975). It serves to lead groups based on human values and is often applied in pedagogical contexts. The core idea is that the common cause at hand, in this case the occupation (IT), others’ expectations (WE), and own aspirations (I) need to be balanced within the scope of action allowed by the respective environment (GLOBE) (Schneider-Landolf et al., 2017). This process is visualized with the TCI model (Figure 5). Lotz (2012) used the model to analyze social situations. The model was useful for the present research interest as it includes the idea of shaping the relation between the subject and his/her environment in a balanced way, which is also the core function of identity development. This method was adapted and used as a heuristic approach for within-case analysis (section 4.3).

**Figure 6:**
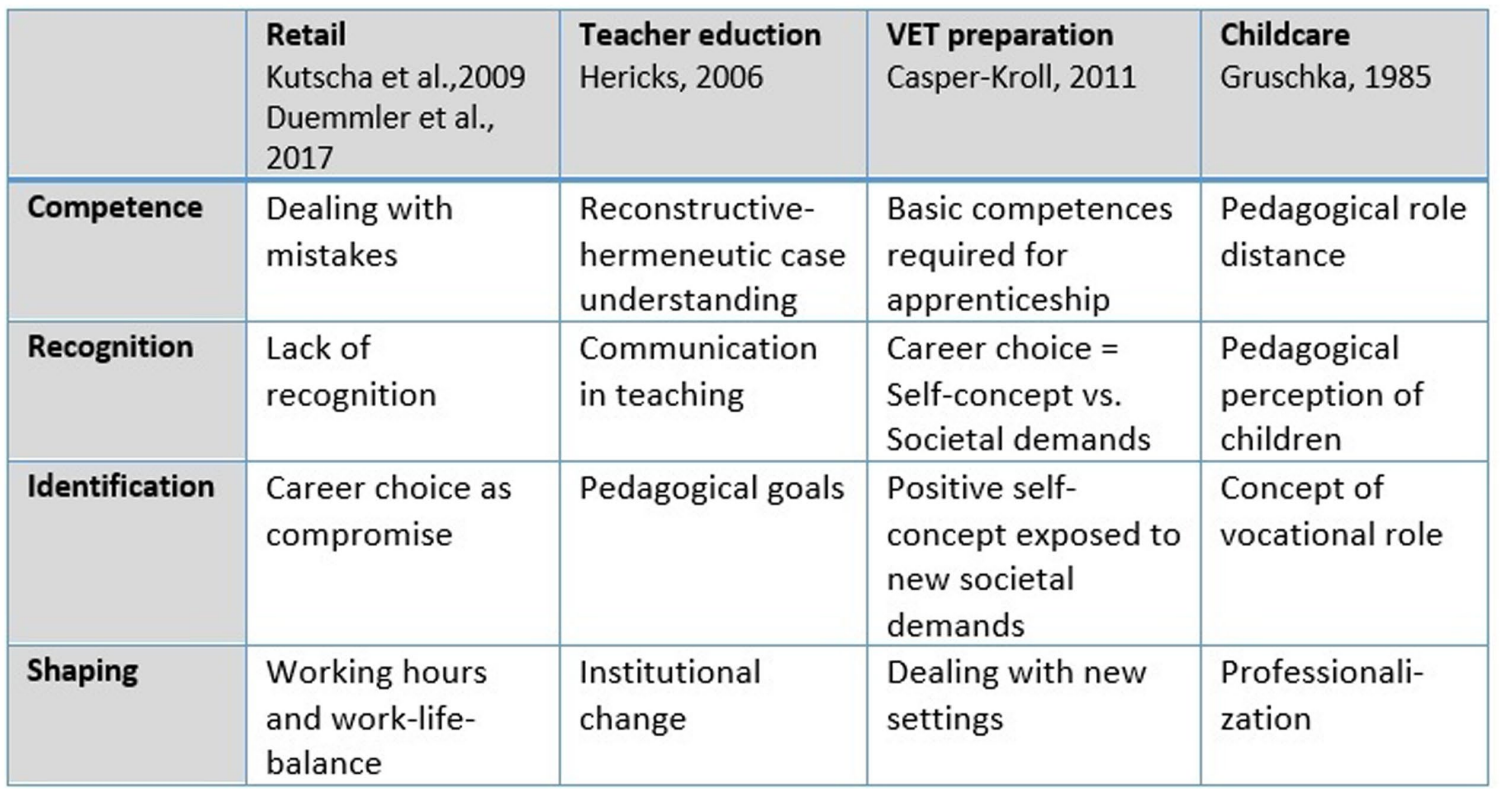
Developmental tasks in VET studies (Thole, 2021, p. 276).

Eisenhardt (1989) suggests looking at data in many divergent ways to avoid premature conclusions and bias in initial subjective interpretation of cross-case patterns. The author started with an Excel sheet (Supplementary material 9) displaying the cases in lines and recorded quantitative and qualitative features in columns. This allowed her to change the within-case and cross-case perspective iteratively. To enhance the detection of within-case (in)coherence and cross-case differences and commonalities, aspects indicating possible identity conflicts were marked in red, features reflecting successful identity balance were marked in green. She also looked for extreme results. This sharpened her perception of commonalities in the remaining group. It was for instance striking that all participants scored extremely high on Deusinger (1986) sociableness scale FSKU except Ciara who felt she was in the wrong place. This indicates that sociableness is an important factor influencing career choice in the retail sector. Another strategy was to look specifically for patterns regarding the concept of developmental tasks. On the one hand, she used categories that aimed to replicate or falsify results regarding typical conflicts found in other retail studies (Kutscha et al., 2009; Duemmler et al., 2017). On the other hand, she compared results of studies using the concept of developmental tasks in different sectors (Figure 6). Formulating them in a more abstract way revealed a recurring pattern allowing her to use the abstract generic terms *identification*, *recognition*, *competence*, and *shaping* as categories. Beyond that, she was also searching inductively for new patterns. Overall, the categories gained from the constructs presented in section 2.3 proved to be comprehensive and only a few categories had to be added (Supplementary material 8).

### 3.6. Shaping theory

A good theory is empirically grounded, plausible, useful, and compatible with existing evidence (or falsifies it) (Horlebein, 2009, pp. 21ff.). For theory-building Eisenhardt (1989) suggests an interactive process triangulating initial tentative patterns with evidence from each case in order to refine the constructs and build evidence supporting them. The author sharpened and triangulated her emerging model by iteratively relating several sources to each other: the above-mentioned categories from other retail and VET studies, her own perceptions of recurring questions in the data, comparisons of the different studies in question, assignment of empirical data and identity theories to the emerging model. For the interpretation of data, she was also able to draw on observations of teaching she gathered during her study on the teachers’ view on the curriculum (Supplementary material 3). Finally, the resulting model was applicable to all cases (Thole, 2021, pp. 443–481) and compatible with findings from other studies (Figure 6) and theoretical assumptions regarding identity development (section 2.2). The evidence found on typical conflicts in the retail sector turned out to be a concretion of the more abstract developmental tasks observed in various VET courses. The final model was compared to the federal and school-specific curriculum (section 4.4). A core outcome was that three of four developmental tasks were not taken into account in the federal curriculum (KMK, 2004/2016). The school-specific curriculum (Tramm et al., 2009) did theoretically cover all of them, but the empirical cases indicated an urgent need for support regarding *identification*, *recognition*, and *shaping* (section 4.3, Thole, 2021, pp. 85–94, 177–203).

### 3.7. Enfolding literature

Eisenhardt (1989) states that it is important to embed the researcher’s own findings in the state of research. It is especially important to reflect on literature that contradicts the researcher’s own results and to find explications to maintain confidence in own findings. In the author’s case, the reported evidence was of far-reaching significance not only for her research field, but also for VET at a federal level. Therefore, the author gave up doing analysis on other constructs but started enfolding relevant literature. This confirmed that the gaps discovered in the federal curriculum (KMK, 2004/2016) had also been found in other VET curricula (Fischer and Hantke, 2018). Literature showed considerable evidence that supporting apprentices in all four developmental tasks is indispensable to develop vocational action competence (Thole, 2021, pp. 97–175; Heinrichs et al., 2022). It also revealed evidence of systematic negligence of subjective matters in pedagogy and economics (Thole, 2021, pp. 205–236) and provided pedagogical concepts aiming to support identity development (Thole, 2021, pp. 237–307). There were also pedagogical positions advocating the exclusion of subjective and identity matters. In her thesis, the author referred to these dissenting opinions and refuted them with arguments (Thole, 2021, pp. 215–236). Her within-case analysis also produced a contradiction with Gruschka (1985) assumptions on the temporal order of developmental tasks. While he assumed there was a logical sequence and was unable to find corresponding empirical evidence, the author’s results showed idiosyncratic states of developmental tasks suggesting that the four tasks interact and that most persons have individual key areas of conflict to start with (Thole, 2021, p. 277).

### 3.8. Reaching closure

Eisenhardt (1989) suggests identifying points of saturation to stop adding cases and iterating data and theory. The author closed the sample when 14 respondents were reached while Eisenhardt (1989) suggests a sample of 4–10 cases. The higher number was chosen as dropouts were probable. Consequently, some cases in the sample were alike and incremental learning by adding more cases was low. Theory-building was stopped when the empirical data of all cases fitted the model. As the collected data material covers numerous concepts that have not yet been analyzed, it would allow other interesting aspects to be studied, such as transformations between the two interviews, coping strategies, nature of moral judgment, developmental tasks in private life, discrimination, or typologies with respect to certain criteria.

### 3.9. Quality criteria

*Detailed documentation of methodology* as presented in this paper is only one of six quality criteria for qualitative research suggested by Mayring (2002). The author also strove to respect the others:

- *Supporting interpretations by arguments:* The examples in section 4.3 show how the author arrives at conclusions from the data. One important criterion was the coherence of various pieces of information regarding the case and plausibility with respect to the relevant state of research.
- *Regularized methodology:* The rules are outlined in sections 3.1–3.8.
- *Proximity to the research object*: Research was conducted at the VET schools in a real setting. Interviews allowed close contact with the stakeholders.
- *Validation by communication:* Referring to conclusions from the first interview during the second allowed validation with the point of view of the interviewee.
- *Triangulation* is extensively described in section 2.

As the PhD project was an important part of the author’s work biography she developed her own criteria for her research with respect to the nature of the research object *identity.* She set value on *continuity*, *coherence*, *consistency*, and *individuality* with respect to her own work biography and state of VET science (Thole, 2021, pp. 338–340). The described methodology led to the following results.

## 4. Results

The following findings have led to the conclusions reported in sections 3.6 and 3.7.

### 4.1. State of research

The retail studies (Kutscha et al., 2009; Duemmler et al., 2017) had identified four major challenges retail apprentices had to cope with:

- *Identification*: Most learners consider retail a second or even last choice after failing to find an apprenticeship in their preferred vocation.
- *Competence*: Apprentices are either expected to show competence like experienced staff and/or lack opportunities for competence development.
- *Recognition*: Students suffer from the low prestige of the retail sector and experience a lack of recognition by customers, supervisors, and society in their role as apprentice.
- *Shaping*: Apprentices must balance private life with unattractive working hours.

Both studies emphasize the necessity to support apprentices at VET school to cope with these challenges. Prenzel et al. (2001) also state that opportunities for motivated learning are rare in the retail business and that learners therefore need more support from VET schools. Heinemann et al. (2009) conclude that supporting vocational identity development is an explicit task of VET.

### 4.2. Cross-case analysis

The aforementioned findings were replicated by the author. Only three respondents wanted to stay in the retail sector in the long term. For 10 of them an apprenticeship in the retail sector was a compromise. Ten interviewees were afraid to make mistakes. Another 10 reported low self-esteem in their role as apprentice. Twelve respondents had difficulties balancing working hours and leisure time (Thole, 2021, pp. 85ff.). Overall, apprentices reflected on four crucial questions:

- Do I want to stay in this occupation? (*Identification*)
- Am I seen as a fully-fledged member of staff? (*Competence*)
- Will I be offered permanent employment after the apprenticeship? (*Recognition*)
- What will be the next career step after the apprenticeship? (*Shaping*)

This pattern also replicated the findings of other VET studies (Figure 6). However, within-case analysis showed that the concrete occurrence of the universal developmental tasks was very idiosyncratic and that the learners were left on their own regarding challenging developmental tasks. The following three exemplary cases illustrate this finding.

### 4.3. Within-case analysis

#### 4.3.1. Nils: career as vocation

Nils (Thole, 2021, pp. 456–458) is interested in commerce and has intentionally chosen the retail sector to build his career. He strives for an additional qualification for selling fresh products at serve-over counters and plans to take a further qualification as sales administrator (*Handelsfachwirt*). During the second interview he is shocked as his additional qualification has become futile due to company reorganization.

Identification: “*Due to the management my calling becomes void.”* (Thole, 2021)

Competence: *“The clientele at this place is not the right one for my professional competence.”* (Thole, 2021)

Recognition: *“Body language shows that I am part of the community.”* […] *“The head of the market does not come down and say: Well done.* […] *In all markets there is a lack of recognition.”* (Thole, 2021)

Shaping: *“There is a need for sales administrators.* […] *Because the career is important, for my future existence, my family* […] *This is why I am in the world* […] *That I am solvent, money makes the world go around, money is power.”* (Thole, 2021)

**Figure 7:**
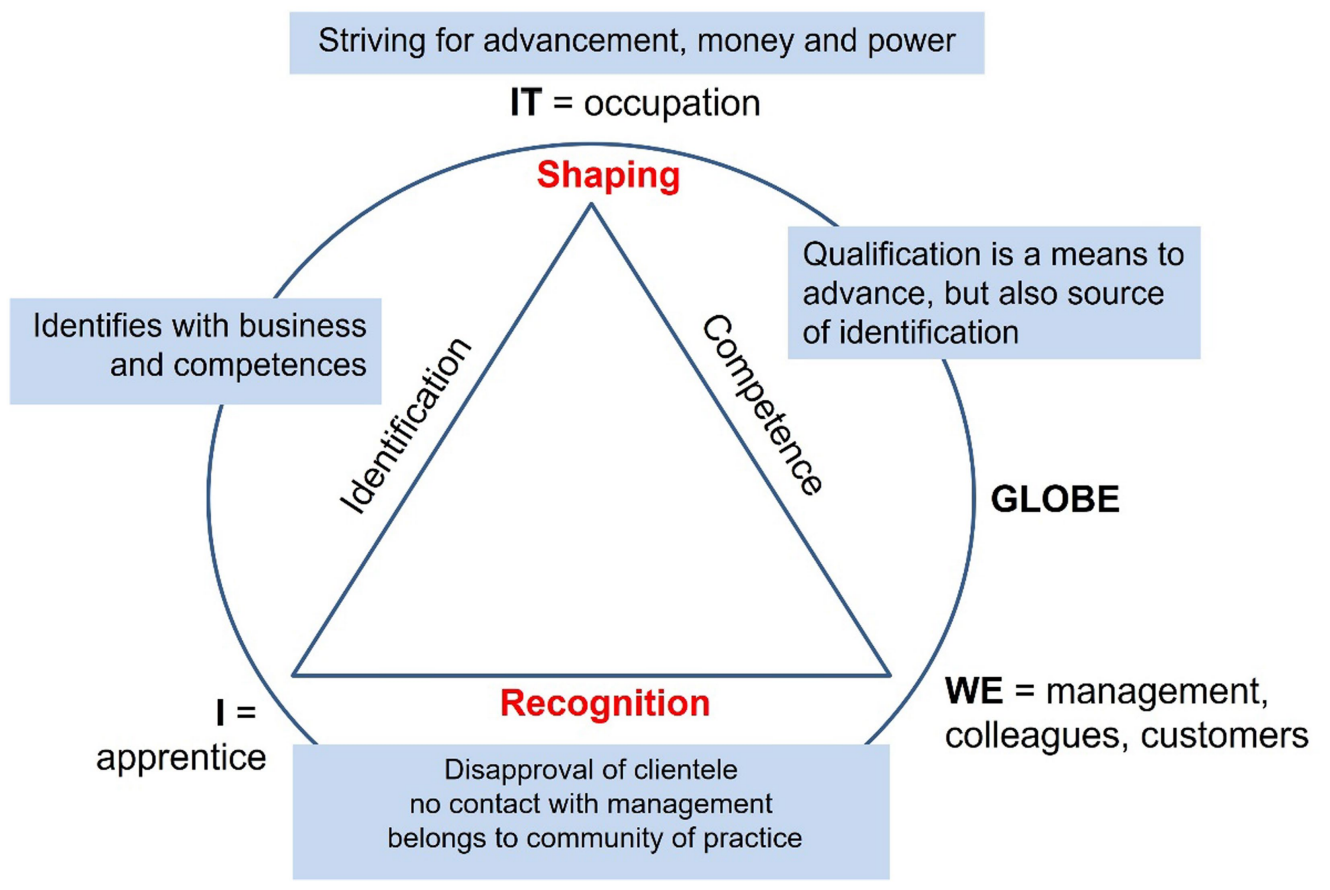
Nils' case—career as vocation (Thole, 2021, pp. 91ff.).

Figure 7 visualizes Nils’ state of developmental tasks in the TCI model. He identifies with his occupation and a standardized career and acquires competences in a purposeful way. High scores in all self-concept and emotional competence dimensions (Deusinger, 1986; Rindermann, 2009) reflect his professional self-confidence. This is also demonstrated by the way he speaks (Bohnsack, 2010) as he presents himself as a fully-fledged skilled employee and reports feeling more adult than his peers due to severe illness during childhood.

*“Actually I am specialized in office management”* […] *“I am also a specialized vendor for serve-over counters.”* (Thole, 2021, pp. 456–458)

However, a sequential analysis following objective hermeneutics (Oevermann et al., 1987) shows that his self-presentation alternates between his desired professional self-concept and his current role as apprentice. Furthermore, he hesitates when he is asked about his career choice motives and gives a reason that is not confirmed in practice as he detests the customers. In fact, his shaping strategy is very materialistic.

“*I have to reflect on it. I assume that it is because I like to work with customers.”* (Thole, 2021, pp. 456–458)

*“I am an apprentice. I do not know, I’m afraid.”* (Thole, 2021)

The experienced crisis shows that his career strategy is not flexible in case of setbacks as his ambiguity tolerance is low (Lind, 1987). In addition, he is failing to get recognition from the clientele and the management although he works desperately on his *social* self-concept to meet societal expectations. From a pedagogical point of view it would be desirable to support Nils to explore his *personal* self-concept as this may help him to accept his imperfection, clarify his vocational ambitions, and start to balance experienced discrepancies and claim recognition.

#### 4.3.2. Ciara: search for herself

**Figure 8:**
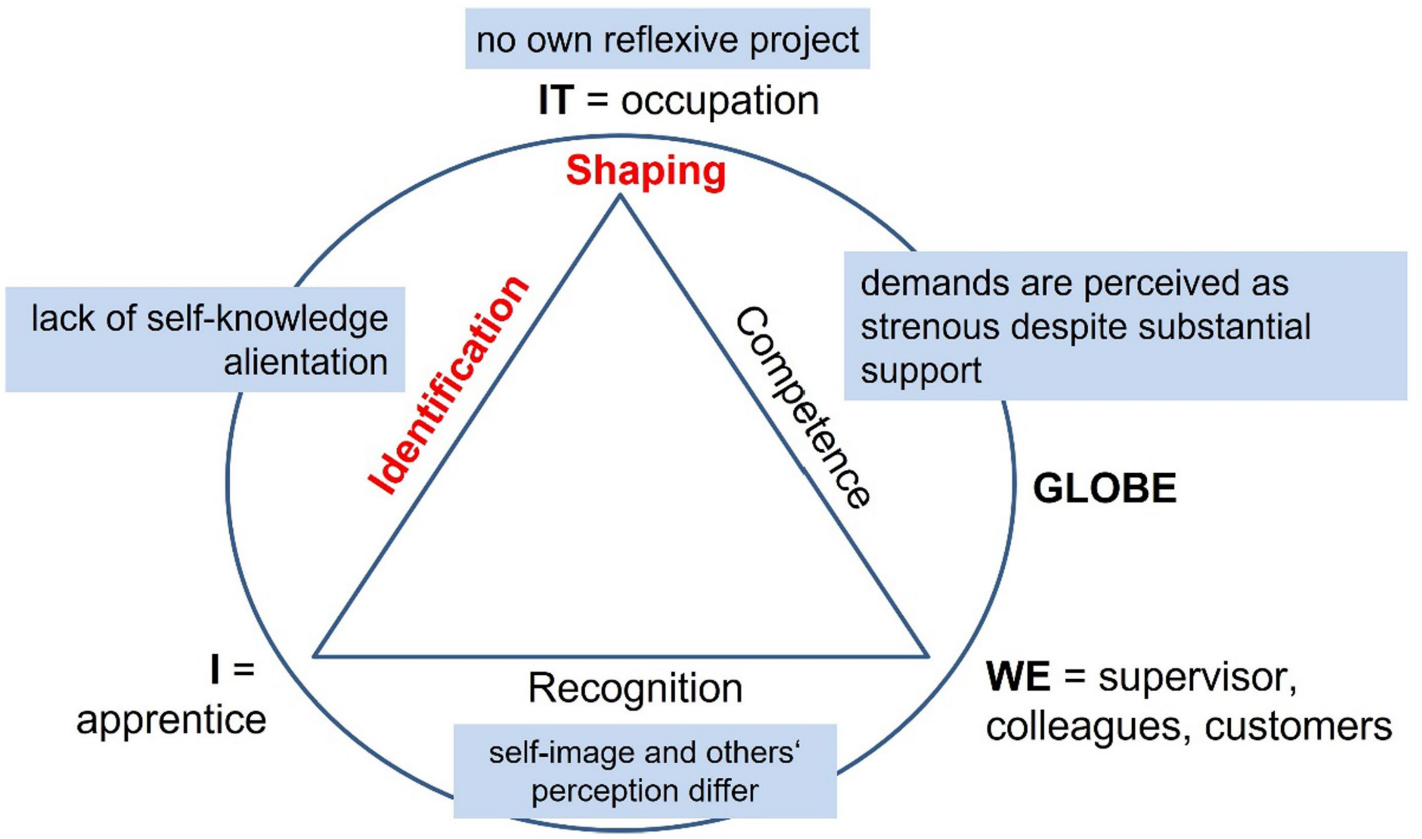
Ciara's search for herself (Thole, 2021, pp. 91ff.).

During the first interview, Ciara feels alienated (Figure 8; Thole, 2021, pp. 443f). A career counselor had advised her to apply for an apprenticeship in the retail sector, as she had no projects of her own. At her workplace she feels uncomfortable despite positive feedback from customers, but is unable to identify why. At the time of the second interview, she has dropped out and found a training place at a nursery.

Identification: *“It was not the right occupation for me.* […] *I knew it from the very beginning, that retail is not the right thing for me. But somehow I wound up there.”* (Thole, 2021)

Competence: “*Retail sales assistant is a very demanding occupation, I think* […] *because you have to look after a lot of things and pay attention to a great deal and you have to know a lot—although you wouldn’t think so.”* (Thole, 2021)

Recognition: *“Others tell me that customers like to talk to me, that I am good at socializing with customers, although I do not feel so, because I am the type of person who says ‘okay, actually I do not manage to do so,’ that is my biggest problem.”* (Thole, 2021)

Shaping: *“Somehow I slipped into this business and yes: at a certain point in time I started to reflect and to collect information and then I decided that the social sector is more suitable for me.”* (Thole, 2021)

Her statements reveal low self-reflection skills, which is plausible in the light of Fonagy et al. (2016) theory about interrelations between early bonding and mentalizing, as Ciara grew up with a single father without contact with her mother. She also scores high on Rindermann (2009) scales for awareness and regulation of *others*’ emotions and very low on perception and expression of her *own* emotions. She was the only participant who scored low on Deusinger (1986) sociableness scale FSKU. In addition, her manner of speaking expresses a large distance between herself and her occupation (Bohnsack, 2010): *“… I wound up here” … “I slipped into this business” … “Others tell me.”* So retail does not become part of her social identity. It is also striking that she reports being overtaxed although objectively her intelligence and talents should be capable of meeting the demands of the apprenticeship (Oevermann et al., 1987).

Her main developmental task is the lack of a self-concept and consequently a vocational vision. She needs pedagogical support to discover herself and develop reflective skills to design a suitable career path.

#### 4.3.3. Kostas: from rags-to-riches?

**Figure 9:**
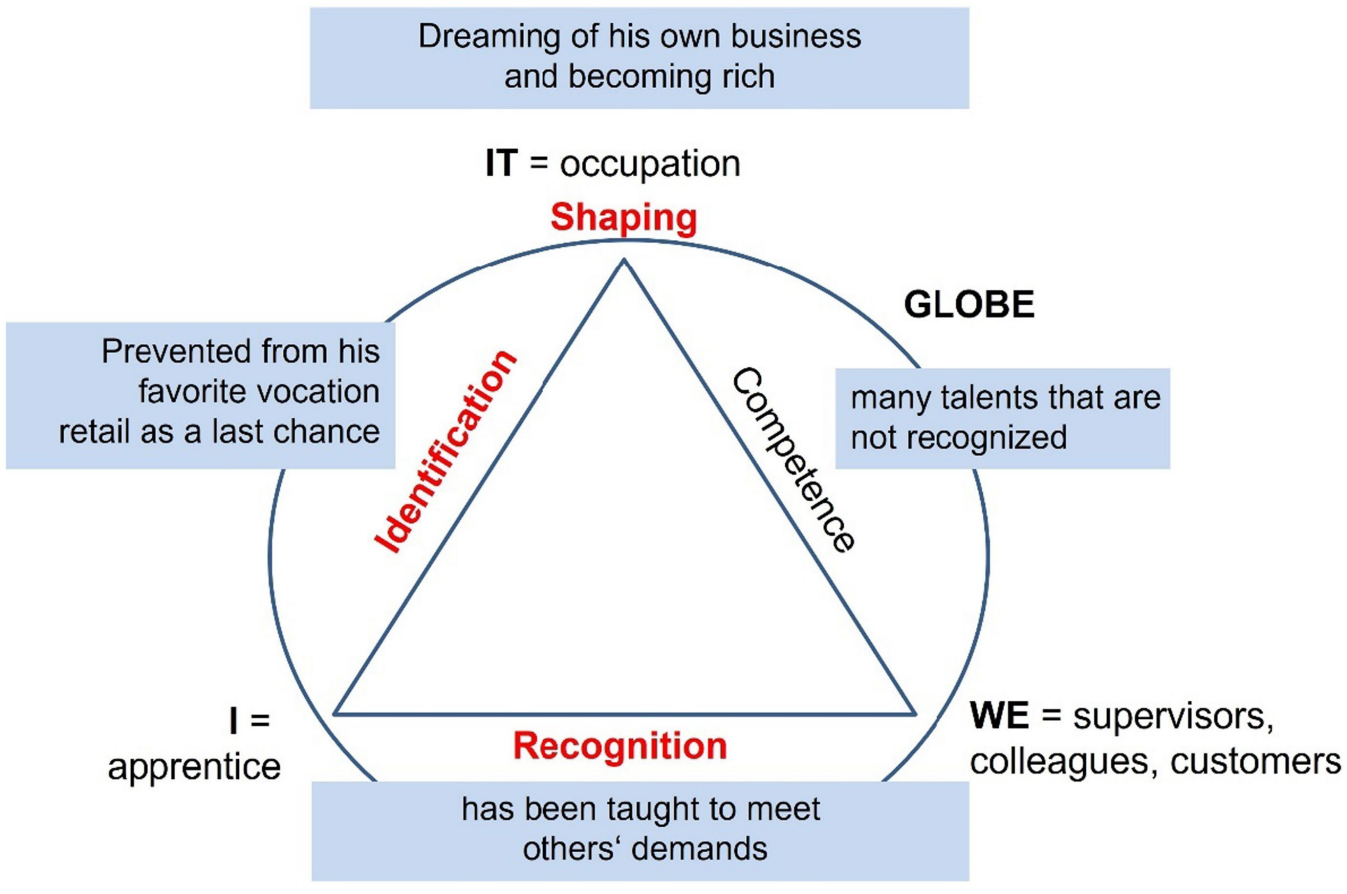
Kostas’ dream of rag-to-riches (Thole, 2021, pp. 91ff.).

Kostas (Figure 9; Thole, 2021, pp. 448ff) had to drop out of an apprenticeship as electrician for health reasons. He identified with that vocation and considers retail to be his last chance to finish a VET course. His previous vocational experience is not valued by his boss.

Identification: *“This school doesn’t suit me. This occupation doesn’t suit me.”* (Thole, 2021)

Competence: *“I was still upset, because he (the boss) thought that I dared to do this although I am a first-year apprentice, and I did it right and I said it was not my fault and he didn’t believe me.”* (Thole, 2021)

Recognition: *“Exactly, I am not appreciated, and I am not treated as an equal. Do the cleaning. Dispose of the waste. Why me? Who am I? Why do I have to do the washing-up?”* (Thole, 2021)

Shaping: “*I am honest. I strive for this since my childhood: self-employment. Every foreigner dreams of having his own business, his own company. Where his fellows are. Work with them. Work for them. Who work for him. And make big money.”* (Thole, 2021)

Looking at his social competencies (Krappmann, 1969; Veith, 2010) reveals a crucial reason for his failure to balance identity:

Role distance: *“People are like chameleons. You have to adapt.”* (Krappmann, 1969; Veith, 2010, Kostas II, 8)

Moral judgment: *“I have grown up as a Christian, Orthodox.* […] *The one who is altruistic will get into paradise.* […] *Be altruistic.”* (Krappmann, 1969; Veith, 2010, Kostas I, 82)

Kostas’ statements indicate that he unilaterally adapts to social expectations notwithstanding how this impairs his well-being and personal objectives. His result on Lind (1987) scale for ambiguity tolerance is the lowest of all participants and he also scores low on expressing and regulating his own emotions (Rindermann, 2009), problem solving, and decision making (Deusinger, 1986). His style of moral judgment matches Kohlberg (1984) third conventional developmental stage, which strives to comply with social norms. He rather denies his own personal self-concept instead of accepting the existence of diverging points of view.

Kostas is talented and motivated. He has a reflexive project, but no clear strategy to get there. His current patterns of behavior fail to bring his real self to bear. He needs support to develop a career strategy and improve his self-presentation and role-distance in a way that allows him to pursue his goals.

### 4.4. Effects on the DBR context

To evaluate the school-specific curriculum, the identified needs for support had to be referred to the objectives of the school-specific curriculum. All three learners need help to clarify their vocational self-concept to achieve identification, shape their further career, and claim recognition. The mandate of Bildung implies that it is VET schools’ task to do so (section 2.1). The aim of the subdimension *Identity and role* is to facilitate the learners’ identification with their vocation and support them to take over their vocational role. This is an example from learning field 2, *Customer-oriented sales conversation* (translated from German):

*“Learners play their vocational role as a vendor to satisfy customers and their employer. […] They consider selling as appreciative service to the customer, which empowers them to pronounced self-confidence based on professional competence and empathy.”* ^7^

The vocational role is a standardized one. Using individual strengths and talents for individual role distance like in Kostas’ case is hardly addressed. Professional competence and recognition are interpreted in a rather adaptive and submissive way. The curriculum fails to raise the issue of claiming recognition proactively.

In addition, the subdimension *Career development* does not put the question of individual talents and competences beyond the retail sector. Rather, sources of identification, in case of need outside the retail sector like in Kostas’ or Ciara’s case, need to be sought for.

*“Learners know of core activities in the retail sector and related opportunities for further education.* […] *Learners understand the significance of their selling competences for future professional opportunities. They want to take advantage of opportunities to develop within their company to increase their vocational chances during and after apprenticeship.”*^8^

Although most apprentices do not want to remain in the retail business, they are trained as if they are going to spend their working life there. However, vocational orientation needs to be continued during the VET course (Meyer, 2014). In addition, the subdimension *Career development* basically relies on cognitive aspects like knowledge about the world of work and own competences. However, only in Ciara’s case is this an issue. Nils and Kostas would need to work on motivational and pragmatic aspects such as pursuing one’s goals consequently, or strategies to deal with obstacles—especially in social interaction. Unfortunately, these are hardly addressed in the curriculum. On the whole, the curriculum meets issues of identity development in a rather idealistic way but does not address learners’ real challenges.

These results are not only relevant for the DBR context but also for retail and other VET courses at federal level as they follow the same guidelines. To facilitate transfer to other contexts, the results were published in a thesis—which has gained attention among German speaking VET scholars (Thole, 2021). Within the DBR context the author reported the results to her doctoral supervisor and her colleagues at an early stage. This enabled them to include them in curricula in other DBR VET contexts. As the project Evanet EH is officially closed there will be no cycle for implementation but results have been presented to the VET schools that intend to improve didactic concepts. Didactic recommendations have been published (Thole, 2020).^9^ They follow the principles of a *Reflexive business and economic education* (Tafner, 2015, 2019; Kutscha, 2019) and underline the need for structured reflection and interaction in the classroom to clarify the person-environment relation of each individual learner. This implies that the VET teacher’s role changes. Identity development remains the students’ responsibility but VET teachers have to provide a learning environment that facilitates this process.

## 5. Discussion

Eisenhardt (1989) research design proved to be adequate for the author’s research interest. Although she finally concentrated on a single notion, basing the interview guidelines on a multitude of concepts was helpful to generate and understand the required information. It also offered the possibility to look for explanations in within-case analyses and find supporting arguments for interpretations. Although the research design may seem laborious, it can be considered efficient in terms of resources for two reasons: on the one hand, only a small sample is required to get a deep view of the research object; on the other hand, the data material can be used for secondary research with other foci of identity development.

Regarding the limitations of current research, the approach offers a methodology to pursue a three-fold goal: within-case understanding, cross-case universal evidence, and theory building. For gaining representative results, research can be combined with quantitative surveys on larger populations testing the cross-case patterns found. The methodology can also help to explain results of quantitative studies by connecting case-studies on a small sample of the initial population.

Concerning data analysis, it must be emphasized that using a unique non-adapted method from *social science* like *Qualitative Content Analysis* or *Grounded Theory* would have led to crucial aspects for designing pedagogical concepts being overlooked. Especially the understanding of interdependent factors of identity development is impeded when categories are mainly used for cross-case analysis. Within-case write-ups based on a triangulated methodology allowed a deep understanding of learners’ idiosyncratic needs of pedagogical support. The added value with respect to the original material is the theory-based presentation of the case that improves theoretical understanding and comparability. With limited resources, it was not possible to apply different methods of analysis to the entire data. Instead, text passages were selected that seemed suitable for a certain approach. This may seem arbitrary, and the process is difficult to record and present in an intersubjective way, but resulting case interpretations can be presented and justified in a documented way (section 4.3). To reduce subjectivity, a team of investigators would be desirable. If this is not possible, as in the author’s case, the research field should already be well-researched to be able to triangulate evidence from different sources.

In an *educational* DBR project (DBR Collective, 2003; Barab and Squire, 2004; Anderson and Shattuck, 2012) it is crucial that results are useful for improving practice and easy to implement. Using the *Theme-centered process analysis* (Lotz, 2012) as a heuristic model for within-case analysis contributed to understanding and communicating complex within-case interdependencies of developmental tasks and their individual origin. It also helped to show that developmental tasks are universal at an abstract level, but very idiosyncratic when it comes to concrete challenges. A major added value of this approach is that the theme-centered model can easily be used for communication with VET teachers, student teachers, learners, or other interested audiences, when it comes to discussing possible pedagogical approaches to foster individual identity development. In combination with a shortened version of the interview guideline, it may also be used as reflective tool for diagnostic purposes or learners’ self-reflection. However, this presupposes that VET schools intentionally foster a *subjective* interpretation of the VET course and provide the resources—especially time—for identifying individual developmental goals in a dialogue between VET teacher and learner. So far, this is often not fulfilled, and the model has not yet been tried out in VET practice.

Finally, it can be concluded that DBR contexts are for several reasons recommendable for studying identity development. First, they facilitate the methodology suggested in this paper. Second, they ensure that new evidence is quickly applied in practice which is crucial because there is an urgent need for improvement. They also offer the possibility to use the same tools in research and classroom. Third, transferability—which is usually restricted in DBR contexts because of the specificity of the context—is not necessarily impeded as other VET schools in Germany have to comply with the same guidelines and conditions in trades are often similar at a national level. In the present case, general insights for VET at an abstract level were generated, while concrete manifestation in other vocations or countries requires further research. Last but not least, DBR theoretically offers the possibility to do participative research which means to involve learners not only as respondents but as members of the research team.

## Author’s Note

Supplementary Datasheet 2 is reproduced with the permission of Tade Tramm.

## Data availability statement

The raw data supporting the conclusions of this article will be made available by the authors, without undue reservation.

## Ethics statement

The studies involving humans were approved by Behörde für Schule und Berufsbildung Hamburg—Institut für Bildungsmonitoring und Qualitätsentwicklung IfBQ. The studies were conducted in accordance with the local legislation and institutional requirements. The participants provided their written informed consent to participate in this study. Written informed consent was obtained from the individual(s) for the publication of any potentially identifiable images or data included in this article.

## Author contributions

CT wrote and prepared the manuscript.

## Footnotes

^1^*Identification* describes the fit between a person’s self-concept and an entity serving as reference (for example a group, organization, or vocation).

^2^*cf.* http://evaneteh.ibwhh.de/ (Accessed August 22, 2023).

^3^*Bildung* is the German concept of a broad education encompassing both knowledge and personal development. The “mandate of *Bildung*” refers to the requirement laid out in national education policy documents that *Bildung* be included in VET.

^4^The self-concept is the individual’s perception of him−/herself. It is formed through interaction with the person’s environment and influences the way he/she acts (Shavelson et al., 1976).

^5^Although this approach is controversial, it has been applied as the author considered it suitable to stimulate complex moral reasoning. It was not the aim to do a cross-case study on moral judgment but to collect information about this important aspect of the individual self-concept.

^6^*cf.* http://www.kabuenet.de/ (Accessed August 24, 2023).

^7^*cf.* http://evaneteh.ibwhh.de/index.php/curriculare-ergebnisse/vertikale-kompetenzen/15/5-b1-berufliche-identitaet.html (Accessed March 31, 2023).

^8^*cf.* http://evaneteh.ibwhh.de/index.php/curriculare-ergebnisse/kompetenzmatrix/8-be4-berufsbildung-und-perspektiven.html (Accessed March 31, 2023).

^9^*cf.* https://www.bwpat.de/ausgabe/39/thole (Accessed September 3, 2023).
