# Supplementary material for: Theme-centered interaction and developmental tasks as research method and pedagogical tool regarding identity development in VET
Source: Front Psychol. 2023 Oct 10;14:1201305. doi: 10.3389/fpsyg.2023.1201305 (PMC10597703; doi:10.3389/fpsyg.2023.1201305)
Supplement: Supplementary file 1 [file Data_Sheet_1.PDF]

Supplement 1: Studies on vocational identity development CS=cross-sectional LG=longitudinal MP=multi-perspective QN=quantitative QL=qualitative MM=Mixed methods

Authors	Year	CS LG MP	Number	QN QL MM	Research Focus	Theoretical Approach	Operationalization Data collection	Operationalization Data analysis
Kohn and Schooler	1982	LG	3101	QN	Relations between social background, job conditions and ideational flexibility and self-directedness	interactionist, social determinism complex model of occupational self-direction and job structure	Standardized questionnaire	statistical
Gruschka	1985	LG	120	QL	Evaluation of new VET course	developmental tasks subjective curriculum	tasks based on developmental tasks of objective curriculum validation: observations/interviews	objective hermeneutics (Oevermann)
Hoff,Lempert and Lappe	1991	LG	19	QL	influence of apprenticeship and work on moral judgment, work-life-balance sense of control	interactionist, developmental stage theory (Kohlberg)	Interviews, workplace observation	qualitative
Witzel and Kühn	1999	LG	198/92 (QL) 2042 (QN)	MM	vocational career strategies	BARB-Model	Questionnaires, Interviews	qualitative and statistical Typology of career strategies
Beck	2000	LG	174/97/35 (QN) 6 (QL)	MM	Moral judgment	Kohlberg's theory on moral judgment developmental stage theory	adapted Heinz-Dilemma moral judgment test Lind questionnaire and interviews	qualitative and statistical
Krapp, Lewalter and Wild	2001	LG	117 (QN) 49/71/38 (QL)	MM	interest, motivation	self determination theory (Deci and Ryan)	questionnaire - random surveys on quality of experience recorded observations	statistical
Prenzel, Kramer and Drechsel	2001	CS LG	917	QN	interest, motivation	self determination theory (Deci and Ryan)	questionnaire with Likert scales ovservation, intervention	statistical
Raeder and Grote	2004	CS MP	59 employees 14 employers	MM	flexibility in vocational career strategies	Process model of identity development (Hausser) psychological contract (Rousseau)	semi-standardized biographical interviews, expert interviews questionnaires	qualitative content analysis (Mayring) typology of career strategies statistical
Bremer and Haasler	2004	LG	240	QL	vocational identity and competence development	developmental tasks (Havighurst) acceptance of vocational standards community of practice	evaluation tasks	qualitative - typology of solutions
Voswinkel and Korzekwa	2005	CS MP	59 employees 10 management/works committee	QL	recognition in the service sector	roleplay (Goffman) - Framing backstage	case studies, semi-structured interviews, observations	qualitative data analysis
Hericks	2006	LG	24	QL	professionalization of students for teaching degree	developmental tasks	narrative interviews	triangulation objective hermeneutics documentary method (Bohnsack)
Nohl	2006		9	QL	Bildung (transformation) in work biographies midlife, adolescence, seniority	biography (action schemes, institutional passages, contingent/intrinsic transitions	narrative interviews	documentary method
Billett	2007	LG	4	QL	relations among work, identity and learning transformations of self and work	interactionist (Lave and Wenger) reflexive action (Giddens)	series of semi-structured interviews case studies	qualitative
Haasler	2007	LG	194 (QN) 90 (QL)	MM	interdependency between competence and identity development choice of occupation, identification, vocational self-awareness	developmental tasks and strategies typology of solution types	practical work evaluation tasks multiple choice questionnaire short questionnaire	qualitative statistical

Supplement 1: Studies on vocational identity development CS=cross-sectional LG=longitudinal MP=multi-perspective QN=quantitative QL=qualitative MM=Mixed methods

**Authors**	**Year**	**CS LG MP**	**Number**	**QN QL MM**	**Research Focus**	**Theoretical Approach**	**Operationalization Data collection**	**Operationalization Data analysis**
Bühler	2007	CS	46	QL	bonding between self and vocation	subjectification of work	non-structured interviews	case studies, typology sequence analysis (Oevermann) grounded theory
Kirpal, Brown and Dif	2007	CS MP	345	QL	coherence of self-picture, mobility, flexibility	boundaryless career	semi-structured, problem-centered interviews with employees and management	Grounded theory inductive evaluation criteria
Dick, van et al.	2008	CS	358/308	QN	work group and organizational identification	social identity (Tajfel and Turner)	questionnaire	statistical
Ashforth, Kulik and Tomiuk	2008	CS	105	QL	person-role-interface in the service sector	role play (Goffman)	semi-structured interviews	grounded theory
Heinemann, Maurer and Rauner	2009	CS	1560	QN	vocational identity, commitment, working morale	vocational action competence novice-to-expert paradigm	self-developed scales	statistical
Kutscha, Besener and Debie	2009	CS	65 (QL) 514 (QN)	MM	critical incidents, coping school-work-transition as developmental task	developmental tasks (Havighurst) Ecology of human development (Bronfenbrenner)	problem-centered interviews standardized questionnaire	grounded theory (QL) statistical (QN)
Homburg, Wiesecke and Hoyer	2009	CS triadic	64/15 (QL) 258 employees 597 customers	MM	social identity and service profit chain	social identity (Tajfel and Turner)	interviews standardized questionnaire	statistical
Heinzer and Reichenbach	2013	CS	1642	QN	vocational abitilities, demands, motivation (independent variables) other dependent var.	self determination theory (Deci and Ryan) model of congruence and discrepancies	self-developed scales 297 items	statistical
Kaak et al.	2013	CS	750	QN	vocational choice competence-dimensions knowledge, motivation, action	vocational development (Super; Holland)	self-developed scales 137 items	statistical
Klotz, Billett and Winther	2014	CS	504 187	MM	vocational identity, workplace identity workplace effort	Commitment (Heinemann and Rauner) Balu, Carson and Bedeian)	14-item Scale competence tests	statistical
Gammoh, Mallin and Pullins	2014	CS	246	QN	identification with brand and company	social identity (Tajfel and Turner)	questionnaire	statistical
Berg	2017	CS	382/177	QN	vocational identity, commitment, working morale	Holland 1993	11-Itm-Scale	statistical replication
Duemmler,Caprani and Felder	2017	CS	25 interviews 65 observation	QL	problems during apprenticeship	socialization theories, identity theories (Krappmann)	interviews, observation	qualitative
Krotz	2017	n. a.	n.a.		media socialization	identity theories	n.a.	n.a.
Lange	2019	LG	18	QL	coping at beginning of apprenticeship	transitions, developmental tasks stress model (Lazarus and Folkman) self-determination theory (Deci and Ryan)	problem centered interviews coping process interview	qualitative content analysis (Mayring)
Kirchknopf and Kögler	2020	CS	51	QL	source of vocational identity	vocational awareness (Beck)	structured interviews	qualitative content analysis
