# Supplementary material for: Theme-centered interaction and developmental tasks as research method and pedagogical tool regarding identity development in VET
Source: Front Psychol. 2023 Oct 10;14:1201305. doi: 10.3389/fpsyg.2023.1201305 (PMC10597703; doi:10.3389/fpsyg.2023.1201305)
Supplement: Supplementary file 2 [file Data_Sheet_2.PDF]

Universität Hamburg

Fakultät 4: Erziehungswissenschaft, Bewegungswissenschaft und Psychologie
Sektion 3: Berufliche Bildung und Lebenslanges Lernen
Institut für Berufs- und Wirtschaftspädagogik

Tade Tramm, Wiebke Hofmeister, Michaela Derner

EvaNet-EH

Evaluation des Innovationsnetzwerks Einzelhandel in Hamburg

(H1, H6, H11 und H13 in Kooperation mit dem IBW Hamburg)

Abschlussbericht zum 11.09.2009

Inhaltsverzeichnis

1 PROBLEMSTELLUNG UND VERORTUNG 1

1.1 VORGESCHICHTE UND RÜCKBLICK 1

1.2 STRUKTUR DES PROJEKTS 2

1.3 PRAKTISCHES WISSENSCHAFTSVERSTÄNDNIS UND EVALUATIV-KONSTRUKTIVE CURRICULUMSTRATEGIE 10

2 BESCHREIBUNG UND ANALYSE DES ENTWICKLUNGSPROZESSES 15

2.1 PROZESSBESCHREIBUNG 15

2.1.1 Positive Aspekte und Problemfelder an der H1 15

2.1.2 Positive Aspekte und Problemfelder an der H6 17

2.1.3 Positive Aspekte und Problemfelder an der H11 18

2.1.4 Positive Aspekte und Problemfelder an der H13 19

2.1.5 Sichtung der Produkte der curricularen Entwicklungsprozesse 20

2.2 PROZESSANALYSE 21

2.2.1 Analyse der schulinternen Entwicklungsarbeit 21

2.2.2 Der kooperative Entwicklungsprozess der vier EH-Schulen 22

2.2.3 Analyse der curricularen Produkte 23

2.3 EMPFEHLUNGEN FÜR DIE KONTINUIERLICHE WEITERENTWICKLUNG 24

2.3.1 Empfehlungen bezogen auf die schulinterne Entwicklungsarbeit 24

2.3.2 Empfehlungen bezogen auf die schulübergreifende Kooperation 25

2.3.3 Empfehlungen bezogen auf die curricularen Produkte 27

2.4 NEUE PROJEKTAUSRICHTUNG 28

3 ANALYSE UND WEITERENTWICKLUNG DER CURRICULAREN PRODUKTE 30

3.1 THEORIEGELEITETE BEGRÜNDUNG DER CURRICULAREN STRATEGIE 30

3.2 PROZESSE 40

3.2.1 Evaluativer Prozess 40

3.2.2 Konstruktive Prozesse 41

3.3 PRODUKTE 47

3.3.1 Lernfeld-Planungen 47

3.3.2 Kompetenzdimensionen 50

3.3.3 Lernfeld-Kompetenz-Matrix *52*

3.4 GESAMTREFLEXION 55

4 IMPLEMENTATION DER CURRICULAREN PRODUKTE UND VERSTETIGUNG DER ARBEITSPROZESSE 57

4.1 DER STATUS BEI PROJEKTENDE ALS AUSGANGSPUNKT ZUR WEITERENTWICKLUNG 57

4.2 INTERVIEWS MIT DEN SCHULLEITUNGEN ZUR ERFASSUNG DER EINSCHÄTZUNGEN 59

4.3 ZUSAMMENFASSUNGEN DER INTERVIEWS UND GESAMTEINDRUCK 60

4.3.1 Interviews an der H1 .... *60*

4.3.2 Interview an der H6 .... *61*

4.3.3 Interview an der H11 .... *62*

4.3.4 Interview an der H13 .... *63*

4.3.5 Zusammenfassender Eindruck aus den Interviews *63*

4.3.6 Empfehlungen für die künftige curriculare Arbeit an den vier Einzelhandelsschulen *64*

LITERATURVERZEICHNIS 70

Abbildungsverzeichnis

Abbildung 1: Vier EH-Schulen-Kooperation von 2004 bis 2007 1

Abbildung 2: Evaluationsbereiche 2

Abbildung 3: Ordnerstruktur auf der WiBeS-Plattform 20

Abbildung 4: Modelle der schulinternen Entwicklungsarbeit 21

Abbildung 5: Lernfeld-Kompetenz-Matrix von Lerne-MFA 28

Abbildung 6: Koordination der zwei Arbeitsstränge 29

Abbildung 7: Entwicklungsperspektiven fächerstrukturierter Curricula 32

Abbildung 8: Entwicklungsperspektive lernfeldorientierter Curricula 33

Abbildung 9: Ziele der Berufsschule 35

Abbildung 10: Handlungskompetenz als lernfeldübergreifende Entwicklungsperspektive 37

Abbildung 11: Verknüpfung der Entwicklungsperspektiven von Lernfeldern und Kompetenzdimensionen 38

Abbildung 12: Verknüpfung lernfeldspezifischer und lernfeldübergreifender Kompetenzentwicklung (Tramm 2008) 39

Abbildung 13: Übersicht der Produktanalyse 41

Abbildung 14: Lernfeldbezogener Entwicklungsprozess im Rahmen von EvaNet-EH 42

Abbildung 15-2: Beispiel für Zielsetzungen eines Präsenztreffens 44

Abbildung 15-1: Beispiel für eine Tagesordnung 44

Abbildung 15-3: Aufgabenstellungen für die Arbeitsgruppen 44

Abbildung 16: Idealtypische Arbeitsschritte im Workshop Kompetenzdimensionen (Tramm 2006) 46

Abbildung 17-1: Curriculare Analyse 48

Abbildung 17-2: Strukturplanung 49

Abbildung 17-3: Makroplanung 49

Abbildung 18: Kompetenzdimensionen im Einzelhandel 51

Abbildung 19: Die Lernfeld-Kompetenzmatrix auf www.ibw.uni-hamburg.de/evaneteh 53

Abbildung 20: Lernfeld 2 auf www.ibw.uni-hamburg.de/evaneteh 54

Abbildung 21: Kompetenzdimension Identität und Berufsrolle auf www.ibw.uni-hamburg.de/evaneteh 54

ABBILDUNG 22: LEITFRAGEN FÜR DIE ABSCHLUSSINTERVIEWS..59

ABBILDUNG 23: REVOLVIERENDER PROZESS 65

Tabellenverzeichnis

Tabelle 1: Zieltableau - curricularer Entwicklungsprozess schulintern 4

Tabelle 2: Zieltableau – curricularer Entwicklungsprozess schulübergreifend 4

Tabelle 3: Zieltableau – curriculare Produkte 5

Tabelle 4: Zieltableau – Implementationsprozess (Aneignung und Umsetzung) 6

Tabelle 5: Zieltableau – Implementationsprozess (Rahmenbedingungen).............................6

Tabelle 6: Maßnahmen und Zwischenberichte 9

Tabelle 7: Befunde bezogen auf die schulinternen Entwicklungs- und Implementationsprozesse 24

Tabelle 8: Befunde bezogen auf die Prozessebene der schulübergreifenden Kooperation 25

Tabelle 9: Befunde bezogen auf curriculare Produkte...27

Tabelle 10: Termine und Gegenstände des Workshops Kompetenzdimensionen 46

Tabelle 11: Kompetenzentwicklung in der Subdimension Berufsethos 52

Tabelle 12: Ansatzpunkte zur Weiterentwicklung 58

1 Problemstellung und Verortung

1.1 Vorgeschichte und Rückblick

In Hamburg werden an vier Berufsschulen Einzelhandelskaufleute sowie Verkäufer/Verkäuferinnen im Einzelhandel ausgebildet. Für diese Schulen bot es sich an, die Umsetzung der KMK-Lernfeldvorgaben gemeinsam anzugehen, zumal die Planungszeit vor der Inkraftsetzung im August 2004 äußerst knapp war.

Im April 2004 fand zu diesem Zweck eine gemeinsame Auftaktveranstaltung statt, die gemeinsam mit Prof. Tramm vom Institut für Berufs- und Wirtschaftspädagogik der Universität Hamburg vorbereitet und realisiert wurde. Die vier Schulen verständigten sich auf Eckpunkte einer gemeinsamen curricularen Entwicklungsstrategie und beschlossen, die für Hamburger Schulen zur Verfügung stehende Internetplattform WiBeS (Wissensmanagement für Berufliche Schulen in Hamburg) für diesen Prozess zu nutzen.

Es folgten drei Jahre Entwicklungs- bzw. Erprobungsphase. Entsprechend der vereinbarten Entwicklungsstrategie wurden die Lernfelder des Einzelhandelscurriculums arbeitsteilig und ihrer Chronologie folgend bearbeitet. In jeder Schule wurden Konzeptgruppen gebildet, die ihre Arbeitsergebnisse in einen zu diesem Zweck eingerichteten Ordner auf der Internetplattform WiBeS einstellten. Eine schulübergreifende Arbeitsgruppe bestehend aus je einem Lernfeldkoordinator (LFK) organisierte diesen Prozess und legte die Ablagestrukturen des virtuellen Ordners in WiBeS fest. Die Abbildung 1 zeigt diese Arbeitsteilung:

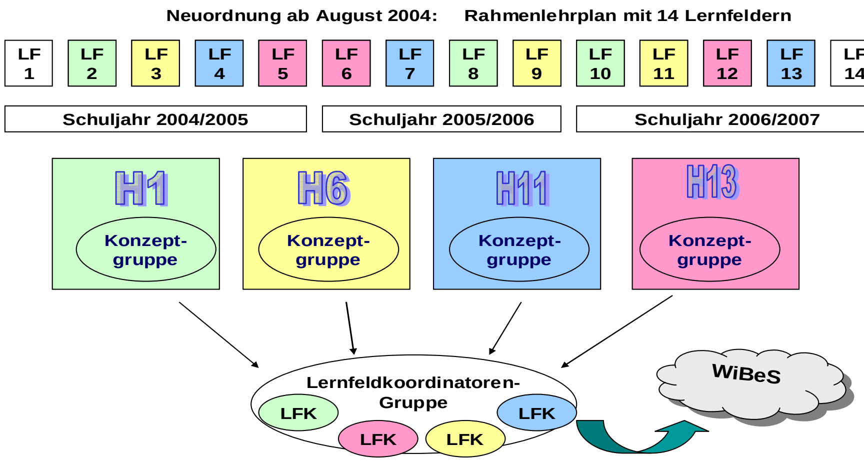

Abbildung 1: Vier EH-Schulen-Kooperation von 2004 bis 2007

Während Lernfeld 1 und 14 von allen Schulen ohne gegenseitige Austauschverpflichtung bearbeitet wurden, waren die Zuständigkeiten für die anderen Lernfelder verteilt. Jede Schule übernahm für drei Lernfelder die Verantwortung. Um den Unterricht im ersten Jahr der Umsetzung nach der Neuordnung zu gewährleisten, wurden zunächst die Lernfelder 2 bis 5, im zweiten Jahr die Lernfelder 6 bis 9 und im dritten Jahr die Lernfelder 10 bis 13 entwickelt. Häufig wurden die entsprechenden curricularen Produkte erst kurz vor Beginn der Unterrichtsphasen in WiBeS eingestellt.

Zum Ende des ersten „Durchganges“ (1. bis 3. Ausbildungsjahr), d. h. während des dritten Umsetzungsjahres (Schuljahr 2006/2007), entschieden sich die vier Schulen, diese innovative vernetzte Curriculumarbeit zu evaluieren. Anlass zur Evaluation und zur Vereinbarung eines gemeinsamen Projektes mit wissenschaftlicher Begleitung war die Vermutung, dass den curricularen Produkten ein „roter Faden“ fehle und Innovationen zu versickern drohten, sowohl in Hinblick auf die erarbeiteten Produkte als auch in Hinblick auf die schulübergreifende Kooperation. Da die arbeitsteilige Erstellung von Unterrichtsmaterial zu Lernfeld 1 bis 14 zunächst beendet schien, wurden auch die zur Verfügung gestellten Ressourcen „eingefroren“. Vom Evaluationsprojekt erhofften sich die vier Schulen neue Impulse.

Das Projekt **„Evaluation des Innovationsnetzwerkes Einzelhandel in Hamburg“** kurz (**EvaNet-EH**) begann im Februar 2007 und endete im Juni 2009. Die Konzeption des Projekts wurde von Prof. Tramm und seine Mitarbeiterinnen vom Institut für Berufs- und Wirtschaftspädagogik (IBW-Team) entwickelt und mit den Schulleitungen abgestimmt.

Zunächst traf sich das Projektteam, das sich aus dem IBW-Team und den Lernfeldkoordinatoren der vier Schulen zusammensetzte, in regelmäßigen Abständen, um die Evaluationsbereiche und Projektziele zu definieren. Das Projektteam bestand über den Projektzeitraum hinweg aus folgenden Personen:

**IBW:**	**LFK der Schulen:**
Prof. Dr. Tade Tramm	H1: Klaus Reige bis 08/07 danach Matthias Odinga
Dr. Sandra Steinemann (bis 12/07)	H6: Ulrike Byza
Michaela Derner (seit 05/07)	H11: Matthias Zastrow bis 02/08, Momke Krumm bis 02/09
Wiebke Hofmeister	danach Beatrice Nentwig-Trumpa
	H13: Stefan Wieprecht

1.2 Struktur des Projekts

Wir haben den Evaluationsgegenstand analytisch in drei Teile untergliedert, wie in der folgenden Abbildung 2 dargestellt:

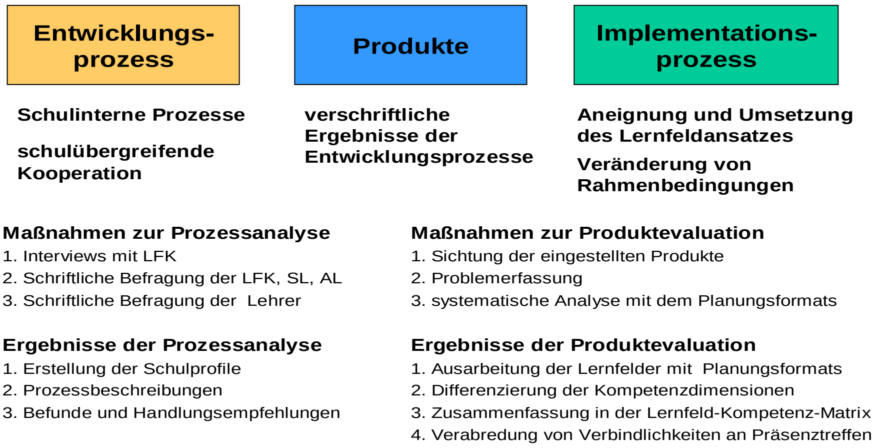

Abbildung 2: Evaluationsbereiche

In Hinblick auf den curricularen **Entwicklungsprozess** ging es darum, die zurückliegende schulinterne und schulübergreifende curriculare Arbeit der vier

Schulen möglichst genau zu erfassen, um Bereiche, Bedingungen und Aspekte zu identifizieren, die für die weitere Arbeit erfolgskritisch waren. Wir beschrieben die Entwicklungsarbeit der Konzeptgruppen und die schulübergreifende Kooperations-arbeit.

In Hinblick auf den **Implementationsprozess** versuchten wir zu erfassen, welche Einstellungen die Lehrer zum Lernfeldkonzept hatten, wie es konkret in den Schulen aufgegriffen und angenommen wurde und wie die Aneignung und Umsetzung der neu entwickelten Materialien erfolgte. Damit im engen Zusammenhang stand die Erfassung von schulischen Rahmenbedingungen, z. B. die Fragen, ob es Lehrerteams gäbe, wie die EDV-Ausstattung für Schüler und Lehrer sei oder inwieweit die räumlichen und organisatorischen Bedingungen zum Lernfeldkonzept passten.

Im dritten Evaluationsbereich analysierten wir die curricularen **Produkte**, die während des Entwicklungsprozesses sukzessive in WiBeS eingestellt wurden. Um die ca. 750 Dokumente schon bei der Sichtung grob zu klassifizieren, nutzten wir ein Planungsformat, das in diesem Bericht in Kapitel 3 detailliert beschrieben wird. Wir konnten so konzeptionelle Unterlagen von Unterrichtsmaterialien trennen und bei den konzeptionellen Unterlagen weitere Unterscheidungen treffen, etwa ob es sich um Beschreibungen von Lernsituationen, von Lernzielen oder um Sequenzierungsvorschläge handelte. Auf diese Weise erreichten wir eine Bestandsanalyse, die uns Aussagen zur Struktur und Einheitlichkeit der curricularen Produkte ermöglichte.

Zu Beginn des Projektes formulierten wir für jeden Evaluationsbereich ein Zieltableau, aus dem der Zweck, der Gegenstand, konkrete Fragen sowie Planungen zu Maßnahmen und Produkten ersichtlich wurden. Diese Zieltableaus werden im Folgenden eingefügt, weil sie einen Überblick über die zentralen Themen und Aktivitäten des Projektes geben. Fragestellungen, die im Zeitablauf revidiert wurden und geplante Maßnahmen, die aus Anpassungsgründen im Zeitablauf gestrichen wurden, sind grau schattiert.

**Curricularer Entwicklungsprozess schulintern**			
**Zweck/Nutzen der Evaluation? („Wozu?“)**	Die schulinterne curriculare Entwicklungsarbeit soll als ständiger Prozess etabliert und optimiert werden. Die kontinuierliche Verbesserung curricularer Angebote entlastet die Kollegen und nützt den Schülern.		
**Was ist Gegenstand der Evaluation? („Was?“)**	Die Prozesse der bisherigen Entwicklungsarbeit werden beschrieben. Die Einschätzung der Akteure zur Qualität des bisherigen Entwicklungsprozesses wird transparent.	Die Bereitschaft und der Widerwille der Beteiligten zur Etablierung eines dauerhaften curricularen Entwicklungsprozesses wird erhoben. Es wird untersucht, welche Faktoren die Etablierung hemmen, welche sie fördern.	Es wird untersucht, was den Erfolg curricularer Entwicklungs-arbeit definiert und welche Qualifizierungsleistungen dazu nötig sind. Es werden Optimierungsansätze zu Arbeits-prozessen, Teamstrategien und Rahmenbedingungen beschrieben.
**Welche pragmatischen Fragen stellen sich?**	- Wer hat die curriculare Arbeit unter welchen Bedingungen verrichtet?<br>- Welche Voraussetzungen waren vorhanden und welche Ziele waren vereinbart?<br>- Welche Rahmenbedingungen haben den Arbeitsprozess unterstützt, welche haben ihn behindert?<br>- Welche Arbeitsstrategien	- Findet zur Zeit Entwicklungs-arbeit statt? Welche, durch wen und in welcher Form?<br>- Welche Faktoren werden von den Beteiligten genannt, die einen dauerhaften Entwicklungsprozess unterstützen oder hemmen?<br>- Inwieweit ist unter den Beteiligten die Einsicht vorhanden, curriculare	- Welche spezifischen Rahmen-bedingungen können an den einzelnen Schulen einen dauerhaften Entwicklungs-prozess unterstützen?<br>- Wie können Teams gebildet und unterstützt werden?<br>- Welchen Fortbildungsbedarf gibt es?<br>- Welche Arbeitsstrategie ist unter den lokalen Bedingungen

	wurden eingesetzt? Welche waren erfolgreich?	Entwicklungsarbeit als einen dauerhaften Prozess anzulegen?	erfolgversprechend?<br>- Inwieweit soll der Prozess schulintern oder schulübergreifend stattfinden?
**Maßnahmen**	1) Interviews mit Lernfeldberatern<br>2) Fragebogen SL, AL, LF<br>3) Dokumentenanalyse<br>4) Fragebogen Entwickler und Anwender	1) Interviews mit Lernfeldberatern<br>2) Fragebogen SL, AL, LF<br>3) Dokumentenanalyse<br>4) Fragebogen Entwickler und Anwender	2) Fragebogen SL, AL, LF<br>4) Fragebogen Entwickler und Anwender<br>5) konzeptionelle Ergänzung der Materialien
**Produkte**	1) Transkripte<br>2) Auswertung, Analyse<br>3) Dokumentation, Ergänzung<br>4) Auswertung, Analyse	1) Transkripte<br>2) Auswertung, Analyse<br>3) Dokumentation, Ergänzung<br>4) Auswertung, Analyse	2) Auswertung, Analyse<br>4) Auswertung, Analyse
**Gesamtziel**	schulspezifische systemische Analyse des curricularen Entwicklungsprozesses		

Tabelle 1: Zieltableau - curricularer Entwicklungsprozess schulintern

**curricularer Entwicklungsprozess schulübergreifend**			
**Zweck/Nutzen der Evaluation? („Wozu?")**	Die schulübergreifende Kooperation soll optimiert werden		
**Was ist Gegenstand der Evaluation?(„Was ?")**	Es wird untersucht, welche Möglichkeiten eine schulübergreifende Kooperation bietet und welche Bedingungen optimal wären. Gegebenenfalls werden auch die Grenzen benannt.	Es wird untersucht, welche Möglichkeiten die WiBes-Plattform für die schulübergreifende Kooperation bietet. Es werden Bedingungen beschrieben, die eingehalten werden müssen, um die Nutzung dauerhaft zu etablieren.	Es wird untersucht, in welchem Maße und für welche Bereiche eine Angleichung oder Vereinheitlichung schulspezifischer Regelungen sinnvoll und die Formulierung entsprechender Standards erwünscht ist.
**Welche pragmatischen Fragen stellen sich?**	- Wollen die Beteiligten eine Kooperation?<br>- Wer kooperiert miteinander?<br>- Was war bislang und was ist jetzt Gegenstand der Kooperation?<br>- Welchem Zweck/Nutzen hat die Kooperation zur Zeit?<br>- Welche Erfahrungen aus anderen Modellversuchen nützen in diesem Zusammenhang?<br>- Welche Faktoren hemmen bzw. unterstützen die Zusammenarbeit?	- Wer benutzt WiBeS für welche Zwecke?<br>- Was wird von den Nutzern positiv bewertet und welche Probleme treten auf?<br>- Was muss getan werden, um die WiBeS zu strukturieren und zu pflegen?<br>- Wie sieht eine Struktur aus, mit der im Sinne der Kooperation gearbeitet werden kann?<br>- Welche Vereinbarungen müssen getroffen werden?<br>- Welche Anweisungen müssen formuliert werden?<br>- Welche Fortbildungsbedarfe ergeben sich?	- Welche Interessen und Befürchtungen formulieren die Kollegien der Schulen?<br>- Welche Interessen und Befürchtungen haben die Akteure der Leitungsebenen der Schulen?<br>- Welche Interessen und Befürchtungen haben die Ausbilder der Betriebe?<br>- Welche Interessen und Befürchtungen haben die Schüler?<br>- Was sind potentielle Handlungsfelder für die Einführung von Standards an den Schulen?
**Maßnahmen ggf. über** Diplomarbeiten	1) Interviews mit Lernfeldberatern<br>2) Fragebogen SL, AL, LF<br>3) Dokumentenanalyse	1) Interviews mit Lernfeldberatern<br>2) Fragebogen SL, AL, LF<br>4) Fragebogen Entwickler und Anwender	2) Fragebogen SL, AL, LF<br>4) Fragebogen Entwickler und Anwender
**Produkte**	1) Transkripte, Analyse<br>2) Auswertung, Analyse<br>3) Dokumentation, Ergänzung	1) Transkripte, Analyse<br>2) Auswertung, Analyse<br>4) Auswertung, Analyse	2) Auswertung, Analyse<br>4) Auswertung, Analyse
**Gesamtziel**	5) Beschreibung der schulübergreifenden Kooperation ggf. Handlungsempfehlungen unter Berücksichtigung der Möglichkeiten der WiBeS-Plattform.		5) Überblick über mögliche Handlungsfelder zur Vereinheitlichung schulspezifischer Regelungen auf der Grundlage bisheriger Befragungen

Tabelle 2: Zieltableau – curricularer Entwicklungsprozess schulübergreifend

curricularer Produkte			
**Zweck/Nutzen der Evaluation? („Wozu?“)**	Die curricularen Produkte sollen in Hinblick auf die Akzeptanz und Entlastungsfunktion auf Seiten des Kollegiums und auf eine Verbesserung der Orientierungsleistung optimiert werden. Die Lehr-Lern-Prozesse der Schülerinnen und Schüler sollen verbessert werden.		
**Was ist Gegenstand („Was?“)**	Theoriegeleitete Beurteilung curricularer Produkte in Hinblick auf ihre curriculare Orientierungsleistung im Sinne des Lernfeldansatzes	Kriteriengeleitete Beurteilung curricularer Produkte durch die Kollegen zur Feststellung der Konkordanz	Beschreibung der Rezeption und Nutzung curricularer Produkte durch die Kollegen zur Feststellung der praktischen Akzeptanz
**Welche pragmatischen Fragen stellen sich?**	- Welche Kompetenzdimensionen können für das Berufsbild beschrieben werden?<br>- Welche idealtypische Struktur kann als Beurteilungsleitlinie dienen?<br>- Inwieweit entsprechen die Produkte den theoretischen Ansprüchen?<br>- Welche Veränderungsschritte sind sinnvoll?	- Welche Erwartungen haben die Kollegen an curriculare Produkte?<br>- Wie beurteilen die Kollegen die vorliegenden Produkte in Bezug auf ihre Übereinstimmung mit dem Lernfeldansatz<br>- Worin besteht aus Sicht der Kollegen die spezifische Schwierigkeit sich mit und im neuen Curriculum zu orientieren?	- Wie, in welchem Umfang und in welchen Zusammenhängen werden die curricularen Produkte genutzt?<br>- Was hat sich bewährt und was nicht?
**Maßnahmen ggf. über Diplomarbeiten**	6) Analyse ausgewählter Produkte<br>7) LF-Kompetenzmatrix Inhaltszuordnung<br>8-12) Workshops zu Kompetenzdimensionen und Präsenztreffen zu LF-Planungen	4) Fragebogen Entwickler und Anwender<br>8-12) Workshops zu Kompetenzdimensionen und Präsenztreffen zu LF-Planungen	
**Produkte**	8-12) überarbeitete Version der Kompetenzdimensionen und Lernfeldplanungen<br>Dokumentation des Arbeitsprozesses		
**Gesamtziel**	Vorläufige Lernfeld-Kompetenz-Matrix für den Einzelhandel		

Tabelle 3: Zieltableau – curriculare Produkte

Implementationsprozess (Aneignung und Umsetzung)			
**Zweck/Nutzen der Evaluation? („Wozu?“)**	Der Implementationsprozess soll in Hinblick auf Nachhaltigkeit und Intensität optimiert werden. Dabei soll die Transparenz und die Verlässlichkeit curricularer Vereinbarungen sichergestellt werden.		
**Was ist Gegenstand der Evaluation? („Was?“)**	Die Prozesse der bisherigen Implementation des Lernfeld-Ansatzes werden beschrieben. Die Einschätzung der Akteure zur Qualität des bisherigen Prozesses wird transparent.	Die Bereitschaft und den Widerwille der Beteiligten zur Implementation des LF-Ansatzes erfassen. Es wird untersucht, welche Faktoren die Etablierung hemmen, welche sie fördern.	Es sollen Erfolgskriterien zur curricularen Implementation definiert und Bedingungen erfolgreicher Implementation beschrieben werden. Gegebenenfalls wird ein Wirkungsmodell erarbeitet.
**Welche pragmatischen Fragen stellen sich?**	- Welche Voraussetzungen sind vorhanden und welche Ziele sind vereinbart?<br>- Welche Rahmenbedingungen haben den Arbeitsprozess unterstützt, welche haben ihn behindert?<br>- Welche Arbeitsstrategien wurden eingesetzt? Welche waren erfolgreich?<br>- Wer nutzt wie die entwickelten curricularen Produkte? Wie wurden die Produkte übergeben? Wo sind diese hinterlegt, wie werden sie gepflegt und überarbeitet? Welchen Nutzen haben sie?	- Wie ist die Haltung und Einstellung der Kollegen (Innovationskultur) zum lernfeldorientiertem Curriculum?<br>- Wie hat sich die Unterrichtskultur (Unterrichtsplanung und –praxis) verändert?<br>- Wie hat sich die Kooperationskultur an den Schulen seit Einführung der Neuordnung verändert? Sind die Lehrer bereit ihre Individualität aufzugeben und curriculare Verbindlichkeiten einzugehen?<br>- Wie findet der Transfer von Erfahrungen statt (Lernkultur)?	- Was kann zur Motivation und positiven Einstellung in Hinblick auf die Umsetzung der Leitideen eines LF-Curriculum beigetragen werden?<br>- Welcher Grad an Verbindlichkeit kann in Bezug auf die Anwendung curricularer Produkte in der Unterrichtsplanung und –praxis vereinbart werden?<br>- Wie kann der schulinterne Austausch von Materialien und Ideen organisiert werden?<br>- Wie kann der schulinterne Transfer von Erfahrungen dauerhaft institutionalisiert werden?<br>- Welche Faktoren führen zum Erfolg? Was könnte getan werden, um den Erfolg zu sichern bzw. zu steigern?

**Maßnahmen ggf. über** Diplomarbeiten	2) Fragebogen Konzeptentwickler und Anwender<br>4) Fragebogen Entwickler und Anwender<br>13) Interviews mit Schul- und Abteilungsleitern<br>15) konzeptionelle Ergänzungen zur Vorbereitung der systemischen Gesamtsicht
**Produkte**	13) Transkription und Analyse
**Gesamtziel**	Entwicklung schulspezifischer Landkarten: Systemische Gesamtsicht, d. h. Beschreibung und Analyse der Wirkungsverhältnisse und Potentialanalysen in Bezug auf einzelne Faktoren

Tabelle 4: Zieltableau – Implementationsprozess (Aneignung und Umsetzung)

**Implementationsprozess (Rahmenbedingungen)**			
**Zweck/Nutzen der Evaluation? („Wozu?“)**	Die schulspezifischen Rahmenbedingungen zur Umsetzung des lernfeldorientierten Curriculums sollen optimiert werden		
**Was ist Gegenstand der Evaluation? („Was?“)**	Die Räumlichkeiten werden in Hinblick auf die spezifischen Anforderungen des Lernfeldunterrichts und entsprechend der lokalen Gegebenheiten beurteilt.	Der LF-Ansatz sieht den Einsatz komplexer Lehr-Lern-Modelle (Modellunternehmen, Planspiele) vor. Die Möglichkeiten des Einsatzes solcher Modelle werden geprüft.	Es soll untersucht werden, inwieweit kooperative Strukturen eingeführt wurden und wie sie sich bewährt haben. Ggf. werden Entwicklungspotentiale benannt.
**Welche pragmatischen Fragen stellen sich?**	- Welche räumlichen Voraussetzungen sind vorhanden?<br>- Welche materielle und mediale Ausstattung wird gewünscht?<br>- Inwieweit werden die Gegebenheiten genutzt und ggf. warum nicht?<br>- Welche Veränderungen könnten stattfinden?	- Welche Modelle und Arrangements werden in welchen Zusammenhängen genutzt?<br>- Gibt es den Wunsch, Modelle verbindlich und schulübergreifend einzuführen?<br>- Welche Modelle ließen sich schulübergreifend einführen?	- Wie ist der Lehrereinsatz organisiert?<br>- Wie findet die Teambildung in den Schulen statt? Auf welchen Prinzipien beruht sie?<br>- Worin besteht die Teamarbeit?<br>- Welche Ziele werden verfolgt?<br>- Gibt es eine Doppelbesetzung und wie ist die praktische Umsetzung? Wie werden Freiräume genutzt?
**Maßnahmen ggf. über** Diplomarbeiten	4) Fragebogen Entwickler und Anwender<br>13) Interviews mit Schul- und Abteilungsleitern<br>14) Besichtigung und Befragung vor Ort, z. B. Räumlichkeiten, mediale Ausstattung usw.<br>15) konzeptionelle Ergänzungen zur Vorbereitung der systemischen Gesamtsicht		
**Produkte**	13) Transkription und Analyse		
**Gesamtziel**	Entwicklung schulspezifischer Landkarten: Systemische Gesamtsicht, d. h. Beschreibung und Analyse der Wirkungsverhältnisse und Potentialanalysen in Bezug auf einzelne Faktoren (schulspezifische Potential- und Defizitanalyse)		

Tabelle 5: Zieltableau – Implementationsprozess (Rahmenbedingungen)

Parallel zur Erarbeitung dieser Zieltableaus planten wir Maßnahmen, die wir auf einer Zeitleiste abbildeten. Es wurde vereinbart, dass in angemessenen Zeitabständen Zwischenberichte vorgelegt werden. Diese beinhalteten sowohl die Dokumentation des Arbeitsprozesses als auch die Arbeitsergebnisse. Die Planungen zum Projektablauf änderten sich etwa zur Halbzeit des Projektes, da die Befunde und Handlungsempfehlungen eine Schwerpunktverlagerung der Aktivitäten erforderten. Der Schwerpunkt verlagerte sich von den Beschreibungen der Prozesse und Analyse der bestehenden Produkte hin zur konstruktiven Begleitung und Beratung bei der Überarbeitung curricularer Produkte mit Hilfe eines Planungsformates. Dieser curriculare Entwicklungsprozess und die Entscheidung der Projektbeteiligten, alle 14 Lernfelder mit Hilfe des Planungsformates zu überarbeiten und Kompetenzdimensionen auszudifferenzieren sowie beide Perspektiven zu einer Lernfeld-Kompetenz-Matrix zu verbinden, nahm nun die gesamten Kapazitäten in

Anspruch. Das führte zur Vereinbarung, dass die grau schattierten Ziele, insbesondere die Ziele zu den Rahmenbedingungen der Implementation (siehe Tabelle 5), vernachlässigt wurden. Die folgende Tabelle zeigt die Chronologie der Maßnahmen und Zwischenberichte:

**Zeit**	**Maßnahmen und Produkte**
Februar 2007	Interviews mit den vier LFK
März 2007	Transkriptionen
April-Mai 2007	- Inhaltsanalysen der Interviews<br>- Formulierung der Zieltableau<br>- Planung der Maßnahmen und Produkte
Juni-Juli 2007	- ergänzende Befragung der LFK<br>- Fragebogen gestütztes Interview mit SL und AL<br>- Analyse ausgewählter Produkte aus WiBeS<br>- Übersicht erstellter Materialien aus dem bisherigen Entwicklungsprozess
**31. Juli 2007**	**1. Zwischenbericht mit folgendem Inhalt:**<br>- Ziele und Maßnahmen des Projektes<br>- Auswertung der Einzelinterviews<br>- Schulübergreifende Synopse ausgewählter Ergebnisse<br>- Vorgehensweise zur Analyse curriculare Produkte<br>- Anhang: Fragebogen für SL und AL
August 2007	- Auswertung bisheriger Befragungen<br>- Zuordnung der Inhaltsangaben und Themen aus der Analyse sowie des RLP in eine Lernfeld-Kompetenzdimensionsmatrix
September 2007	- Erster Entwurf: Kompetenzdimensionen mit Subdimensionen<br>- Vorbereitung und Durchführung einer Auftaktveranstaltung zur Bearbeitung der Kompetenzdimensionen am 20.09.2007
Oktober-November 2007	- Dokumentenanalyse: Sichtung von Protokollen, schriftlichen Vereinbarungen, Arbeitsanweisungen zum Entwicklungsprozess<br>- Erstellung des Fragebogens für Lehrer<br>- Durchführung der Lehrerbefragung<br>- Planung und Durchführung des Workshops zur Kompetenzdimension IBBB, LAT am 30.10.2007<br>- Planung und Durchführung des Workshops zur Kompetenzdimension IBBB am 27.11.2007
Dezember 2007-Januar 2008	- Zusammenstellung der Rohdaten aus Lehrerbefragung<br>- Ergänzungen zur Vorbereitung der Schulprofile<br>- Planung und Durchführung des Workshops zur Kompetenzdimension NORM am 30.01.2008
**15. Februar 2008**	**2. Zwischenbericht mit folgendem Inhalt:**<br>- Beschreibung der bisherigen Aktivitäten<br>- Beschreibung der schulinternen Entwicklungsprozesses (Schulprofile)<br>- Beschreibung des schulübergreifenden Kooperationsprozesses mit Handlungsempfehlungen<br>- Ergebnisse zur Entwicklung der Kompetenzdimensionen
Februar-März 2008	- Ergebnisse und Handlungsempfehlungen des Zwischenberichtes werden den Schul- und Abteilungsleiter präsentiert ; Einigung über Schwerpunktverlagerung und neuer Arbeitsstrategie m Projekt<br>- Vorstellung des Planungsformates (curriculare Analyse, Strukturplanung und Makroplanung) im Projektteam<br>- Schulen sollen Planungsteams bilden, die bisheriges curriculares Material in das Planungsformat überführen<br>- Präsentation des Projektes bei den Hochschultagen in Nürnberg
April-Mai 2008	- Planung und Durchführung des Workshops zur Kompetenzdimension NORM, BWP am 08.04.2008<br>- Bildung von Planungsteams an den Schulen

	- Planung und Durchführung der Auftaktveranstaltung zur Arbeit mit den Planungsformaten am 19.05.2008<br>- Einigung über die Notwendigkeit von Präsenztreffen zur Abstimmung vorläufig verbindlicher Lernfeldplanungen<br>- Diskussionen um eine EvaNet-Homepage und WiBeS
Juni-Juli 2008	- Diskussionen um das Planungsformat, Konkretisierung der Arbeitsaufträge für die Planungsteams<br>- Betreuung der Planungsteams und Rückmeldungen zu ersten Arbeitsergebnissen<br>- Diskussionen um inhaltliche und organisatorische Ausrichtung der Präsenztreffen<br>- Zwischeninformation der Projektarbeit an Schulleiter<br>- Projektverlängerung bis Juni 2009 wird vereinbart<br>- Erstellung des Webblogs „Evanette“ zur Dokumentation der Arbeitsergebnisse der Kompetenzentwicklungsgruppe<br>- Treffen mit Herrn Deh zur Konzeption einer WiBeS-Darstellung der Lernfeld-Kompetenz-Matrix nach dem Vorbild der Lerne-MFA<br>- Erläuterung der didaktischen Jahresplanung der H1 und die Verknüpfungsmöglichkeiten zum Planungsformat
August 2008	- Auswertung und Dokumentation der Lehrerbefragung<br>- Erstellung des 3. Zwischenberichts
**31. August 2008**	**3. Zwischenbericht mit folgendem Inhalt:**<br>- Zusammenfassung der Ergebnisse der Lehrerbefragung<br>- Aufbau des Fragebogens<br>- Auswertung der Befragungsergebnisse (alle Items)
September-Oktober 2008	- Planung und Durchführung des Workshops zur Kompetenzdimension W&C am 09.09.2008<br>- Betreuung der Planungsteams für LF02 und LF03<br>- Planung und Durchführung des Präsenztreffens I am 29.09.09<br>- Nachbearbeitungen der LF02 und LF03<br>- Einstellung der Lernfeldplanungen LF02 und LF03 in WiBeS<br>- Diskussion um Kurzversion für Lernfeldplanungen
November-Dezember 2008	- Planung und Durchführung des Workshops zur Kompetenzdimension W&C am 12.11.08<br>- Planung und Durchführung des Workshops zum allgemeinen Stand der Kompetenzdimensionen sowie zu NORM am 11.12.08<br>- Betreuung der Planungsteams für LF04 und LF05<br>- Planung und Durchführung des Präsenztreffen II am 03.12.09<br>- Nachbearbeitung der Lernfeldplanungen LF04 und LF05<br>- Einstellung der Lernfeldplanungen LF04 und LF05 in WiBeS
Januar-Februar 2009	- Planung und Durchführung des Workshops zur Kompetenzdimension W&C und LAT am 19.01.09<br>- Betreuung der Planungsteams für LF06, LF07 und LF08<br>- Planung und Durchführung des Präsenztreffens III am 17.02.09<br>- Nachbearbeitung der LF06, LF07 und LF08<br>- Einstellung der Lernfeldplanungen LF06 und LF07 in WiBeS
März-April 2009	- Planung und Durchführung des Workshops zur Kompetenzdimension BWP am 04.03.09<br>- Planung und Durchführung des Workshops zur Kompetenzdimension GWR und SYST am 14.04.09<br>- Betreuung der Planungsteams für LF09, LF10 und LF11<br>- Planung und Durchführung des Präsenztreffens IV am 06.04.09<br>- Nachbearbeitung der LF09, LF10 und LF11
Mai-Juni 2009	- Planung und Durchführung des Workshops zur Kompetenzdimension LAT und KOKO am 13.05.09<br>- Planung und Durchführung des Workshops zur Kompetenzdimension BWP am 16.06.09<br>- Nachbearbeitung aller Kompetenzdimensionen und Einstellen in

	„EvaNette“<br>- Betreuung der Planungsteams für LF12, LF13 und LF14<br>- Planung und Durchführung des Präsenztreffens V am 08.06.09<br>- Nachbearbeitung der LF12, LF13 und LF14
- Projektende	
Juli-September 2009	- Überarbeitung und Anpassung aller Lernfeldplanungen sowie Ergänzung um die Arbeitsergebnisse der Kompetenzentwicklungsgruppe<br>- Interviews mit Schul- und Abteilungsleitern zum Status der Implementation und zu weiteren Planungen<br>- Erstellung des **Abschlussberichts**<br>- Vorbereitung und Durchführung der Fachtagung am 16.09.09

Tabelle 6: Maßnahmen und Zwischenberichte

Die Auswertungen der ersten Interviews mit den Lernfeldkoordinatoren sowie der Operationalisierungsprozess der Projektziele der drei Evaluationsbereiche mündeten in den **ersten Zwischenbericht (Juli 2007)**. Um Akzeptanz bei den beteiligten Akteuren zu erlangen, wurden im Bericht die Projektziele, die Arbeitsstrategie und die Projektschritte ausführlich beschrieben. Gleichzeitig wurden die Ergebnisse der Einzelinterviews und somit die Sichtweisen der Lernfeldkoordinatoren in Bezug auf die bisherige Entwicklungsarbeit veröffentlicht. Es folgte die Befragung aller Schul- und Abteilungsleiter im Juni 2007. Auf der Grundlage dieser Informationen konnten Schulprofile entwickelt werden. Diese Schulprofile und die Befunde der Dokumentenanalyse (curriculare Produkte) mündeten gemeinsam in den **zweiten Zwischenbericht (Januar 2008)**. Zu diesem Zeitpunkt bündelten wir die Befunde und formulierten Handlungsempfehlungen, die zur Schwerpunktverlagerung im Projekt führten. Im Oktober 2007 wurde den Kolleginnen und Kollegen des Lernbereichs I aller vier Schulen ein Fragebogen vorgelegt, der die Zieldimensionen zum großen Teil abdeckte. Im **dritten Zwischenbericht (August 2008)** wurden die Ergebnisse dieser Befragung vollständig und systematisch dokumentiert.

Im ersten Projektjahr stand die Erhebung von Informationen zu den bisherigen curricularen Entwicklungsarbeiten sowie die Analyse der erstellen Produkte im Vordergrund. Für die Arbeit der wissenschaftlichen Begleitung bedeutete dies, dass für den Fortgang des Projektes regelmäßige Arbeitstreffen innerhalb des Projektteams ausreichten, um Ergebnisse abzusichern und Erkenntnisse rückzukoppeln. Das Auswerten und Dokumentieren konnte im Kreise der wissenschaftlichen Begleitung durchgeführt werden. Dies änderte sich im zweiten Projektjahr, da nun intensiv mit den Lehrern an den Schulen zusammengearbeitet wurde. Insbesondere zur Vorbereitung der Präsenztreffen, an denen jeweils bestimmte Lernfeldplanungen zur Diskussion gestellt wurden, gab es mehrere Treffen in den Schulen der jeweiligen Planungsteams. Die Workshops zur Ausdifferenzierung der Kompetenzdimensionen fanden am IBW statt. Die Teilnehmer dieser Workshops waren ebenfalls Lehrer der vier Schulen. Während sich im ersten Projektjahr die Zusammenarbeit auf das Projektteam (IBW und LFK) begrenzte, erweiterte sich der Kreis der Beteiligten im zweiten Jahr erheblich. Die eigentliche Zielgruppe war nun involviert und am Projektgeschehen beteiligt.

In Hinblick auf die curricularen Entwicklungsprozesse ist es gelungen, die Befunde der dem Projektzeitraum vorgelagerten Entwicklungsarbeiten konstruktiv zu nutzen und bessere Arbeitsstrategien, Kommunikationsstrukturen und Formate einzuführen. Die vier Schulen wollten einen kooperativen Entwicklungsprozess fortführen, haben die Empfehlungen des IBW aufgenommen und die neuen Arbeitsstrategien mit großem Ressourcenaufwand unterstützt. Deswegen konnten auf den insgesamt 5

Präsenztreffen alle 14 Lernfeldplanungen bearbeitet und während der 10 Workshops der Kompetenzentwicklungsgruppe alle 7 Kompetenzdimensionen ausdifferenziert werden. Das zentrale Produkt der Projektarbeit ist die Lernfeld-Kompetenz-Matrix, die ebenso vorläufig wie verbindlich ist. Die Projektbeteiligten sind sich darüber einig, dass der jetzige „Arbeitsstand zur Erprobung“ verbindliche Grundlage der Unterrichtsplanung ist aber auch darüber, dass curriculare Arbeit ein kontinuierlicher Prozess ist und somit die Unterrichtserfahrungen evaluativ-konstruktiv in einen Überarbeitungsprozess einfließen müssen.

1.3 Praktisches Wissenschaftsverständnis und evaluativ-konstruktive Curriculumstrategie

Im Rahmen von Projekten, die wie EvaNet-EH eine enge Zusammenarbeit zwischen Wissenschaft und Praxis vorsehen, ist in Anbetracht kontroverser Diskussionen über das Verhältnis zwischen diesen beiden, insbesondere auch in der Lehrerbildung und Forschungspraxis (vgl. z. B. Reinisch 2003, Neuweg 2007), eine Positionierung notwendig, die verdeutlicht, welche begründeten Erwartungen man in solcherart Zusammenarbeit setzen kann. Dazu wird im Folgenden zum einen auf unser Verständnis der Wissenschaft-Praxis Beziehung und zum anderen auf die im Projekt verfolgte evaluativ-konstruktive Curriculumstrategie eingegangen.

a) Das Verhältnis zwischen Wissenschaft und Praxis

Sich zum Teil als beharrlich erweisende Annahmen eines dualistischen und hierarchischen Verhältnisses zwischen Wissenschaft und Praxis, unabhängig davon, ob der Wissenschaft die höhere Rationalität zugeschrieben wird und sie daher als eine Art „Weisungsbefugte“ für die Praxis fungiert oder ob die Wissenschaft streng funktionalistisch als im Dienste der Praxis zweckmäßige Lösungen generierend verstanden wird, führen nicht selten zu Frustrationen auf beiden Seiten.

> „Nach praktischen Lösungen gefragt, ertöne meist ein vielstimmiger, widersprüchlicher Chor aus der Wissenschaft, aus dem kaum Orientierungen für praktische Interventionen heraus zu hören seien. Wenn dies die Kritik von Seiten der Praxissysteme ist, so klingt die Replik von Seiten der Wissenschaft nicht selten folgendermaßen: Praktisches Handeln sei beschränkt und nicht innovationsfreudig. Es folge lieber eingefahrenen Routinen und lasse sich nicht gerne von neuen Erkenntnissen, die von außen herangetragen werden, belehren.“ (Altrichter et al. 2005, 23)

Um solchen blockierenden (Vor-)Urteilen zu entgehen, erscheint es uns notwendig, das Verhältnis zwischen Wissenschaft und Praxis als beiderseitigen Rückkopplungsprozess zu verstehen. Denn zum einen soll Wissenschaft Praxis analysieren und wird daher durchaus in ihren Gegenständen, Vorgehensweisen und Methoden durch die Praxis mitbestimmt und zum anderen soll Wissenschaft der Praxis gerade dadurch Anregung und Orientierung bieten und wirkt daher auch auf die Praxis ein.

Keinesfalls geht es um die einseitige und unmittelbare praktische Anwendung von wissenschaftlichen Theorien, Konzepten oder Modellen. Vielmehr *können* wissenschaftliche Erkenntnisse Eingang in die Praxis finden, indem sie von der Praxis rezipiert werden. Die Art der Rezeption unterliegt wiederum der Interpretation und Modifikation durch den Rezipienten in seiner aktuellen Situation (vgl. Altrichter et al. 2005, 22). Ebenfalls darf das Verhältnis der beiden nicht fehlgedeutet werden als einseitige Nützlichkeitsbeziehung im Sinne einer der Praxis *dienenden* Wissenschaft.

Wissenschaft und Praxis werden als zwei sich an unterschiedlichen Regeln, Zielsetzungen und Bedingungen orientierende Handlungsbereiche verstanden, die sich auf die gleichen Gegenstände beziehen und daher durch Perspektivenerweiterung, die auch reflexive Prozesse beinhaltet, gegenseitig bereichern können. Wir stimmen mit Altrichter et al. (2005, S. 23) überein, wenn sie schreiben, dass „professionelle Praxis, wie wir sie in modernen Gesellschaften kennen, (…) von theoretischem Wissen nicht sauber zu trennen (ist)“ und dass sich, genau umgekehrt, „wissenschaftliche Forschung auf soziale Praxis (bezieht)“. Der angenommene Dualismus von Wissenschaft und Praxis und der oft damit einhergehende höhere Rationalitätsanspruch der Wissenschaft werden genauso wenig wie der Dualismus von Denken und Handeln (vgl. dazu Tramm 1994) als gegeben betrachtet, denn so wie jede Wissenschaft ihre Praxis hat, so hat jede Praxis ihre Theorie (vgl. Altrichter et al. 2005, 23).

Im Fall des Projekts EvaNet-EH, das sich als formatives Evaluationsprojekt und entsprechend eines antidualistischen Verständnisses von Praxis und Wissenschaft konstituierte, zeigt sich das angedeutete Wissenschafts- und Forschungsverständnis vor allem in folgenden Projektmerkmalen:

- Die Rolle der Evaluatoren wurde nicht durch die wissenschaftliche Begleitung wahrgenommen sondern durch eine Kooperation von Wissenschaftlern und Lehrern, deren beider Expertise unverzichtbar für den Prozess innerhalb von EvaNet-EH war. (kooperative Evaluation)
- Entscheidungen, die für den Verlauf des Projektes eine Rolle spielten, (Ziel- und Maßnahmenentscheidungen, Veranstaltungsplanungen, Zwischenberichte etc.) wurden von den beteiligten Wissenschaftlern und Lehrern gemeinsam vorbereitet und getroffen. Dabei konnten die Wissenschaftler ihre Projekterfahrungen sowie ihre theoretischen Kenntnisse als Strukturierungshilfen des Feldes einbringen und die Lehrer ihre Praxiserfahrungen, insbesondere bezogen auf die Schülerklientel und die Unterrichtsprozesse, und ihre Interessen an der (im Übrigen durch die Schulen selbstfinanzierten) Evaluation geltend machen. (gemeinsame Entscheidungsfindung).
- Die Ergebnisse gemeinsam betriebener Praxisanalyse und konstruktiver Entwicklungsarbeit wurden während des Evaluationsprozesses kontinuierlich reflektiert und als Grundlage weiterer Gestaltungsmaßnahmen genutzt. Diese ständige Rückkopplung war nur möglich aufgrund der hohen Kommunikationsbereitschaft aller Beteiligten. Dieses Vorgehen erhöhte die Komplexität des Projektes, verhinderte aber zugleich, und dies wurde als unverzichtbar erachtet, unflexible Strukturen und Kursfestlegungen, die eine Anpassung an neue Erkenntnisse und Bedingungen unmöglich machen. (gemeinsame Reflexion und kontinuierliche Rückkopplung)
- Insgesamt folgte das Projekt einer evaluativ-konstruktiven Curriculumstrategie, die unter Bezugnahme auf curriculumtheoretische Grundannahmen eine Weiterentwicklung der mittelfristigen Curriculumforschung insofern darstellt, als dass eine Abkehr vom Entwickler-Anwender-Konzept postuliert wird, Bildungsprozesse ganzheitlich wahrgenommen werden und handlungs- und kognitionstheoretische Grundannahmen als normatives Bezugssystem dienen (vgl. Tramm 1996, S. 171). Diese evaluativ-konstruktive Curriculumstrategie, die zum einen Ausdruck des beschriebenen Verständnisses der Beziehung zwischen Praxis und Wissenschaft ist und zum anderen die Wechselwirkung zwischen

Analyse, Evaluation und Innovation betont, wird im folgenden Abschnitt bezogen auf das Projekt EvaNet-EH konkretisiert.

b) Evaluativ-konstruktive Curriculumstrategie

Im Falle des Projektes EvaNet-EH bewegten wir uns im originär curricularen Feld, nämlich der Strukturierung und Konkretisierung des lernfeldorientierten Rahmenlehrplanes für den Ausbildungsberuf der Kaufleute im Einzelhandel bzw. Verkäufer/Verkäuferinnen. Ebenso wie Tramm (1996) in seiner Arbeit zur Übungsfirma gehen wir im Rahmen von EvaNet-EH von einem weiten Curriculumverständnis als „begründeten Zusammenhang von Lernziel-, Lerninhalts- und Lernorganisationsentscheidungen“ (Meyer, 1976, S. 128 zit. nach Tramm 1996, S. 149) aus. Wie dieser begründete Zusammenhang im Einzelnen modelliert wurde, ist dem dritten Kapitel zu entnehmen.

Während Tramm (1996) seinen evaluativ-konstruktiven Forschungsansatz auf die Übungsfirmenarbeit anwendet, wurde er im Projekt EvaNet-EH auf den gesamten Komplex der curricularen Entwicklungsarbeit im Rahmen eines Bildungsganges übertragen. In beiden Anwendungsfällen geht es darum, dass „die Komplexität des curricularen Reflexionszusammenhanges grundsätzlich gesichert und doch zugleich so weit reduziert werden kann, dass die Handlungs- und Diskursfähigkeit aller Betroffenen erhalten bleibt“ (ebd., S. 148). Die notwendige Komplexitätsreduktion erfolgte im Rahmen von EvaNet-EH im Wesentlichen durch die Verwendung einheitlicher Arbeitsstrategien und -formate und war zudem von vornherein begrenzt durch den spezifischen Bildungsgang und den entsprechenden curricularen Vorgaben der KMK.

Der klassische Anspruch der Curriculumtheorie ist, curriculare Entscheidungen in Form eines rationalen gesellschaftlichen Konsens´ zu treffen (vgl. Robinsohn 1975, S. 31). Im Projekt EvaNet-EH fand dieser Anspruch seine Berücksichtigung, indem curriculare Entscheidungen stets unter Beteiligung eines großen Teils der Lehrenden und unter Berücksichtigung antizipierter Schülerinteressen und betrieblicher Interessen getroffen wurden. Die Argumentation über das Curriculum war als diskursives Verfahren angelegt, das unter Beteiligung der Lehrenden und der wissenschaftlichen Begleitung stattfand.

Die evaluativ-konstruktive Curriculumstrategie, wie Tramm (1996, S.148-176) sie beschreibt, gründet auf klassischen curriculumtheoretischen Überlegungen Robinsohns, die aber hinsichtlich ihrer übergroßen Komplexität in der Aufgabenstellung, ihrer langfristigen Anlage, ihrer Legitimations- und Implementationsprobleme sowie ihrer einseitigen Ausrichtung auf inhaltliche Aspekte des Curriculums kritisiert werden. Die Erweiterung durch Merkmale mittelfristig-fachdidaktischer Curriculumforschung bedeutet für EvaNet-EH im Wesentlichen:

- eine mittelfristige Forschungsstrategie: Die von den Wissenschaftlern vorgeschlagene Vorgehensweise zur Erarbeitung des Curriculums war (und ist) weder in ihrer theoretischen Begründung abgeschlossen noch unumstößlich. Eine umfassende systematische Evaluation der curricularen Strategie konnte noch nicht erfolgen. Ein paralleles Projekt bezogen auf den Bildungsgang der Medizinischen Fachangestellten (LerneMFA), das der gleichen curricularen Strategie folgt, wird derzeit in Teilaspekten evaluiert.
- eine fachdidaktische Ausrichtung: Ohne die Fächer des allgemeinbildenden Bereichs zu ignorieren, bezog sich die Curriculumentwicklung im Rahmen von EvaNet-EH ausschließlich auf den berufsbildenden Unterricht. Die Fächer

Wirtschaft und Gesellschaft sowie Sprache und Kommunikation waren nicht Gegenstand der Curriculumentwicklung, wurden aber, wo immer es aufgrund von inhaltlichen Berührungen und Überschneidungen notwendig war, berücksichtigt.

- die Anknüpfung an bestehende Unterrichtspraxis: Durch die Sichtung, Beurteilung und Berücksichtigung des bereits vorhandenen curricularen Materials, das ab 2004 an den vier Einzelhandelsschulen erstellt wurde, sowie durch die intensiven Diskurse flossen nicht nur bestehende Unterrichtspraxen, sondern auch die Theorien, Einstellungen und Bedürfnisse der Lehrenden in das neue Curriculum ein. Die Erfahrungen der Lehrenden ermöglichten zudem, dass die unterrichtliche Rahmenbedingungen und Ausprägungen der Beziehung zwischen Lehrenden und Schülern berücksichtigt werden konnten.
- die Verknüpfung von Analyse, Innovation und Evaluation: EvaNet-EH stellt den Versuch dar, die Prozesse und Ergebnisse der Curriculumentwicklung kontinuierlich und parallel zu analysieren, zu evaluieren und schließlich auch zu innovieren. Ausdruck dieses Versuchs sind die kontinuierlichen Anpassungen und die Flexibilität, die während der Projektlaufzeit zutage traten. Diese Verknüpfung von Analyse, Innovation und Evaluation ist zum aktuellen Zeitpunkt nicht so weit fortgeschritten, dass man mit dem Ende des Projektes auch das Ende der Aufgabe verzeichnen könnte.
- eine induktive Vorgehensweise, mit der ein innovativer Praxisansatz aufgegriffen und über Prozesse der theoriegeleiteten Evaluation in einem kooperativen Prozess analysiert und optimiert wird.
- der curriculare Bezug auf prospektive Lebenssituationen unter der doppelten Perspektive beruflicher Mündigkeit und Tüchtigkeit: In den Diskussionen über Unterrichtsinhalte und -ziele wurde die Nützlichkeit des Lerngegenstandes für relevante Lebenssituationen berücksichtigt, indem Fälle, Dilemmata und Schlüsselsituationen gesammelt wurden. Neben diesem eher funktionalistischen Arbeitsschritt, erfolgten auch Überlegungen, welche Erfahrungen den Schülern selbstverantwortliches und mündiges Handeln ermöglichen, um so den normativen Ansprüchen unseres Bildungsverständnisses Geltung zu verschaffen.

Insgesamt ließ der enge Bezug zu curriculumtheoretischen Überlegungen, insbesondere nach Ende des ersten Projektjahres, die Fragestellungen im Evaluationsbereich Implementation in den Hintergrund rücken (vgl. 1.2). Das bedeutete allerdings kein vollständiges Ausblenden der Implementationsaufgabe; vielmehr mussten Aspekte und Fragen der Implementation des Curriculums im Rahmen der wieder aufgenommenen Entwicklungsarbeit berücksichtigt werden. Des Weiteren sei hier angemerkt, dass die Prozesse der Entwicklung und Implementation kaum trennscharf sind, insbesondere dann, wenn Entwickler und Anwender zumindest im Kontext der Lernfeldworkshops die gleiche Personengruppe bezeichnen, wie es bei EvaNet-EH der Fall war. Systematische Betrachtungen und Auswertungen der Unterrichtsprozesse, konnten jedoch nicht mehr stattfinden, so dass vorerst nur Vermutungen über die tatsächliche Realisierung und Wirksamkeit des neuen Curriculums hinsichtlich der Lehr_Lern-Prozesse und der Lernergebnisse angestellt werden können.

Kommen wir zurück auf die Frage, welche Erwartungen man in eine Zusammenarbeit zwischen Wissenschaft und Praxis setzen kann, wenn man in dieser Weise eine evaluativ-konstruktive Curriculumstrategie verfolgt.

Der Erfolg, den eine solche kooperative Arbeit bringen kann, liegt darin, dass „die Komplexität des curricularen Reflexionszusammenhanges grundsätzlich gesichert bleibt und doch zugleich so weit reduziert werden kann, dass die Handlungs- und Diskursfähigkeit aller Betroffenen erhalten bleibt" (Tramm 1996, S. 148). Dabei spannen Vertreter von Wissenschaft und Praxis gemeinsam das Feld mit seinen Komplexitäten auf: Die Wissenschaft, indem sie ihre Erkenntnisse einbringt sowohl als Strukturierungshilfen wie auch als Verfremdungen und Verunsicherungen und die Praxis, indem sie ihre vielfältigen situativen Ansprüche und Erfahrungen sowie ihre subjektiven Theorien geltend macht. Eine in ihrer Komplexität angemessene Strategie zu finden ist eine kooperative Aufgabe. Ein solches Zusammenwirken erscheint uns für die Steigerung der Orientierung von praktisch Tätigen und die Steigerung der Relevanz wissenschaftlicher Erkenntnisse, und somit zur kontinuierlichen Verbesserung von Berufsbildungspraxis und –wissenschaft sinnvoll und notwendig.

2 Beschreibung und Analyse des Entwicklungsprozesses

Als Entwicklungsprozess bezeichnen wir die curriculare Arbeit der Lehrerinnen und Lehrer, die der Konkretisierung der Rahmenlehrplanvorgaben diente. Im Fokus standen einerseits die Arbeitsergebnisse, in denen Angaben zu Kompetenzen, zu Lernsituationen, zu Unterrichtsplanungen im weitesten Sinne und zu Unterrichtsmaterialien in Form von Arbeitsblättern o. ä. beinhalten. Andererseits versuchten wir durch die Interviews mit den vier Lernfeldkoordinatoren und einer ergänzenden Befragung der Schul- und Abteilungsleiter aller vier Schulen, die Arbeitsstrategien und Arbeitsbedingungen von Beginn der kooperativen Entwicklungsarbeit bis zum Beginn des Projektes (Mai 2004 – Januar 2007), möglichst genau zu erfassen. Wir fragten nach folgenden Kategorien:

- Akzeptanz der und Einstellungen zur Neuordnung
- Strategie der schulinternen Konzeptarbeit
- Strategie der schulübergreifenden Konzeptarbeit
- Strategie der Implementation des Lernfeldkonzepts
- Informationsveranstaltungen und Qualifizierungsangebote

Diese Informationen wurden ergänzt um Rahmendaten der Schulen (technische, räumliche Ausstattung, Teamstrukturen etc), sodass für jede Schule ein „Schulprofil“ erstellt werden konnte (vgl. Zwischenbericht II Kapitel 2). Diese Schulprofile enden mit einem Fazit, in dem positive Aspekte und Problemfelder in Hinblick auf die Entwicklungsprozesse benannt wurden. Diese schulspezifischen Zusammenfassungen werden im Abschnitt 2.1. erneut wiedergegeben, da wir auf dieser Grundlage Hypothesen zu hemmenden und fördernden Faktoren der curricularen Arbeit bildeten, die in Abschnitt 2.2 erläutert werden. Wir kamen aufgrund dieser Schlussfolgerungen zu der Empfehlung, den kooperativen Entwicklungsprozess zu revitalisieren. Die Vorschläge seitens des IBW, den Aushandlungsprozess mit den Entscheidungsträgern und die im zweiten Projektjahr verfolgte Entwicklungsstrategie stellen wir in Abschnitt 2.3 dar.

2.1 Prozessbeschreibung

Die folgenden Zusammenfassungen der Erträge und Probleme der schulspezifischen Entwicklungsprozesse stammen aus dem 2. Zwischenbericht vom 15. Februar 2008, in dem auch die methodische Anlage der Interviews und der Auswertungsprozess beschrieben werden. Es sei noch einmal betont, dass in den Zusammenfassungen Prozesse und Rahmenbedingungen beschrieben sind, die den Zeitraum April 2004 bis Januar 2007 umfassen. Alle Schulen haben sich seither weiterentwickelt und verändert. In Abschnitt 2.1.5 wird dargestellt, wie wir die Arbeitsergebnisse der schulinternen Konzeptarbeit, d. h. die in WiBeS eingestellten Dokumente analysierten.

2.1.1 Positive Aspekte und Problemfelder an der H1

Die Schulleitung hat zu Beginn der Neuordnung des Ausbildungsberufes sehr schnell mit räumlichen Veränderungen auf die neuen Anforderungen reagiert. Die räumlichen und medialen Möglichkeiten wurden dem lernfeldorientierten Unterricht angepasst.

Obwohl die allgemeine Stimmungslage zur Neuordnung zu Beginn des Entwicklungsprozesses skeptisch war (aufgrund negativer Erfahrungen in anderen Schulbereichen der H1), ist es gelungen, das Berufsschulkollegium des Einzelhandels zur Entwicklungsarbeit zu motivieren. Dabei war bedeutend, dass die Konzeptgruppe des ersten Umsetzungsjahres aus Zweierteams bestand, die sich freiwillig zusammenfanden. Die Schulleitung hat die Arbeit dieser Konzeptgruppe kontinuierlich und intensiv begleitet, stellte Entlastungsstunden zur Verfügung und kommunizierte die Arbeitsstrategie und die Erwartungen an die Arbeitsergebnisse so, dass der Prozess von allen Beteiligten als transparent wahrgenommen wurde. Diese Transparenz und Offenheit wirkte motivierend auf den Arbeitsprozess.

Teamstrukturen sind an der H1 bereits seit langem etabliert. Vor der Neuordnung basierte die Lernkultur auf informellen Lehrerteams, die Erfahrungen und Material austauschten. Seit Einführung der Neuordnung ist die Bildung von Klassenlehrerteams, die auch gemeinsam den Lernfeldunterricht planen, institutionalisiert. Dabei kann auf die Wünsche und Belange der Lehrer weitestgehend Rücksicht genommen werden.

Der Schul-, der Abteilungsleiter und der Lernfeldkoordinator schätzen ihr Kollegium so ein, dass diese es befürworten, den curricularen Entwicklungsprozess dauerhaft zu etablieren. Sie sind der Meinung, dass es für diese Arbeit Entlastungsstunden geben müsste, da sie nicht Teil der Unterrichtsvorbereitungszeit sein könne. Sie glauben, dass dieser Prozess durch weitere Fortbildungsmaßnahmen zu unterstützen sei. Sie vertreten auch die Meinung, dass die Entwicklung und Umsetzung von Lernfeldern zu einem erhöhten Abstimmungsbedarf zwischen Kollegen führt, der schulorganisatorisch zu berücksichtigen wäre.

Die Schulleitung der H1 hat von Anfang an (ab August 2004), im Gegensatz zu den anderen drei Einzelhandelsschulen, ausschließlich Klassen gebildet, die nach dem Lernfeldkonzept unterrichtet werden und darauf vertraut, dass alle Ausbildungsbetriebe im Laufe der Ausbildungszeit ihre Ausbildungsverträge nach der neuen Ausbildungsordnung (2004) abschließen werden (Die Ausbildungsbetriebe hatten ein Wahlrecht durch die Handelskammer Hamburg – ob sie die Verträge nach alter oder neuer Ausbildungsordnung abschlossen). Diesen Prozess, die Verträge nach Neuordnung abzuschließen, hat die H1 aktiv initiiert, in dem sie alle Ausbildungsbetriebe mit der Bitte angeschrieben hat, die Ausbildungsverträge nach der neuen Ausbildungsordnung (2004) abzuschließen. Dass es die H1 mit dem neuen Ausbildungskonzept ernst nahm und nicht abwartete, zeigte sich auch exemplarisch an dem neu entwickelten Blockplan. Damit hatte die H1 einen zeitlichen Vorsprung von mehreren Monaten bei der Umsetzung des Lernfeldkonzeptes und signalisierte den Ausbildungsbetrieben, den Schülern und Kollegen von Anfang an, dass sie vorbehaltlos hinter der Neuordnung steht. Insofern sind Schulleiter, Abteilungsleiter und Lernfeldberater von Anfang an Antreiber einer Entwicklung gewesen, die nicht mit Sicherheit voraus zu sehen war.

Weitere Umsetzungshilfen gab es in Form des kontinuierlich begleiteten Entwicklungsprozesses und durch die Fortbildungsangebote. Schulintern konnte man jedoch keine Strukturen etablieren, die den Entwicklungs- bzw. Implementationsprozess wirksam unterstützt hätten. Das Bottom-up-Engagement der Lehrer aus der „ersten Welle“ war eine Initialzündung für eine nachhaltige Änderung der Lehr-Lernkultur an der H1.

Eine Überarbeitung der im Zuge der Entwicklungsphase erstellten Dokumente fand zwar statt, war aber nicht institutionalisiert. Es fehlten Regeln und Standards, sodass

diese Prozesse zum Zeitpunkt des Interviews stagnierten. Die Konzeptgruppe war inzwischen aufgelöst, d. h. es wurden keine Entlastungsstunden mehr für curriculare Arbeit vergeben und der kontinuierliche, begleitende Feedbackprozess war eingestellt. Die Vermutung lag nah, dass nur noch auf der Ebene der Lehrerteams ein informeller Erfahrungs- und Materialtausch stattfand. Durch die fehlende strukturelle Etablierung von Feedbackschleifen war die qualitative Weiterentwicklung der Produkte gefährdet. Wahrscheinlich fand aufgrund mangelnder Abstimmung zwischen den Teams Doppelarbeit statt bzw. es wurden keine Synergien genutzt. Ein für das gesamte Kollegium gültiges kompetenzbasiertes Curriculum entstand auf diese Weise nicht.

Aufgrund der Unzufriedenheit mit den im Prozess entwickelten Produkten gab es auch neu initiierte Prozesse, in denen versucht wurde, curriculare Orientierung zu erlangen. Es sollte in Betracht gezogen werden, diese Arbeitsprozesse an den Evaluationsprozess zur Entwicklung von Kompetenzdimensionen anzubinden.

2.1.2 Positive Aspekte und Problemfelder an der H6

Das Kollegium an der H6 stand der Neuordnung und der bevorstehenden Aufgabe der curricularen Konkretisierung aufgeschlossen gegenüber. Das gesamte Kollegium des Lernbereichs I beteiligte sich an der Entwicklungsarbeit. Der Prozess verlief nicht immer reibungslos, so dass man eine Steuergruppe einsetzte, um die Produkte redaktionell zu überarbeiten und in ein stimmiges Gesamtkonzept zu bringen. Das zeigt, dass der Entwicklungsprozess an dieser Schule formativ begleitet wurde.

Ebenfalls positiv sind die fest etablierten Teamstrukturen an der H6. Im Gegensatz zu früheren Annahmen gab es keine Tendenz der Rückkehr zur Einzelarbeit, nachdem sich eine gewisse Routine mit der Lernfeldarbeit eingestellt hatte. Das Modell eines Klassenteams, an dem auch die Lehrer des Lernbereichs II beteiligt wurden, war an der H6 etabliert. Das Funktionieren ist in Hinblick auf die mangelhafte Teamraumsituation bemerkenswert und kann nur mit persönlichem Engagement der Kollegen erklärt werden (Abstimmungsprozess per Telefonkonferenz außerhalb der Schule).

Erwähnenswert ist auch die Kontinuität im Engagement bei der Entwicklungsarbeit. Noch bis vor kurzer Zeit wurden überarbeitete Produkte in WiBeS eingestellt. Auch das schulinterne Bemühen, die curricularen Produkte anderer Schulen anzupassen, schien noch vorhanden. Offenbar war man aber mit den Ergebnissen nicht zufrieden. Der Wunsch, die Produkte weiter zu überarbeiten und anzupassen, der Wunsch Kompetenzdimensionen zu formulieren, um damit möglichst mehr Orientierung zu erlangen und der Wunsch einen Abstimmungsprozess darüber zu initiieren, was konkret verbindliches Curriculum sein soll, wurde gegenüber der wissenschaftlichen Begleitung geäußert. Die Schulleitung hat auf diese Wünsche reagiert, indem die Arbeit zur Konkretisierung von Kompetenzdimensionen, wie es von Seiten des IBW vorgeschlagen wurde, in die Ziel-Leistungsvereinbarungen der Schule mit dem HIBB aufgenommen wurde. Dadurch war zunächst sichergestellt, dass es an der H6 einen kontinuierlichen curricularen Arbeitsprozess gab. Inwieweit dieser Prozess mit dem Evaluationsprozess gekoppelt werden kann und sollte, muss geklärt werden.

Auf die Frage, ob die Vorgaben zur curricularen Entwicklungsarbeit klar und deutlich genug waren, antworteten die Funktionsträger nicht einheitlich. Während der Abteilungsleiter der Meinung war, dass der Entwicklungsauftrag klar angegeben war, betonte die Schulleiterin, dass konkretere Vorgaben und Strukturierungshilfen

sinnvoll gewesen wären. Auch in Hinblick auf die Verbindlichkeit der entwickelten Produkte, gab es keine Einigkeit.

Neben den allgemeinen Veranstaltungen, die es zu Beginn des Entwicklungsprozesses gab, wurden keine besonderen Umsetzungshilfen angeboten. Aus Sicht der Schulleitung war dies auch von den Kollegen nicht gewünscht. Interne Umsetzungshilfen, die auf die Institutionalisierung von Erfahrungstransfer abgehoben und so eine Lernkultur positiv hätten beeinflussen können, hat es nicht gegeben. Die Lernkultur an der H6 lebte vom Engagement einzelner informeller Teams. Es ist anzunehmen, dass die Umsetzung des neuen Curriculum besser und nachhaltiger geschehen könnte, würde man seitens der Schul- und Abteilungsleitung gemeinsam eine Strategie zum schulinternen Erfahrungstransfer entwickeln.

Die Schulleiterin, der Abteilungsleiter und die Lernfeldkoordinatorin glaubten, dass der Abstimmungsbedarf in den Teams seit Einführung der Neuordnung höher sei. Hierfür müssten auch die organisatorischen Bedingungen angepasst werden (mehr Teamarbeitsräume).

2.1.3 Positive Aspekte und Problemfelder an der H11

Zum Zeitpunkt der Neuordnung hatte ein Großteil des EH-Kollegiums bereits Erfahrungen in der Umsetzung lernfeldorientierter Curricula. Es gab bereits weit ausdifferenzierte Teamstrukturen, was in Bezug auf den schulinternen Erfahrungstransfer von großem Nutzen war. Die Schulleitung hat schnell mit räumlichen Veränderungen auf die neuen Anforderungen reagiert. Die räumlichen und medialen Möglichkeiten sind auf den lernfeldorientierten Unterricht zugeschnitten. Der Umgang mit Netzwerkstrukturen hat an der H11 bereits Tradition.

Der Schulleiter und der Abteilungsleiter beschrieben das Kollegium an der H11 als tolerant. Die Kollegen verbesserten sich gegenseitig und tauschten Material aus. Informationsprobleme gäbe es höchstens für neue Kollegen. Das Zurechtfinden gelänge aber aufgrund der vorhandenen Hilfsbereitschaft schnell.

Die Schulleitung war sich darüber einig, dass der curriculare Entwicklungsprozess dauerhaft zu etablieren sei, allerdings stünden hierfür keine Entlastungsstunden mehr zur Verfügung. Durch die etablierten Kommunikationsstrukturen und durch das Netzwerk an unterschiedlichen Konferenzen, bei denen Erfahrungsaustausch in je unterschiedlicher Konstellation stattfanden, sah die Schulleitung den Implementationsprozess ausreichend unterstützt. Die erstellten schulinternen Dokumente, die jedem Kollegen zugänglich waren, betrachten sie als verbindliches Curriculum und gingen davon aus, dass der Unterricht inzwischen lernfeldorientiert und somit kompetenzorientiert erfolge. Der Lernfeldkoordinator hingegen glaubte, dass eher prüfungsorientierte Stoffkataloge handlungsleitend seien.

Ein Problemfeld waren die Teambildungskriterien. Während die Schulleitung der Meinung war, die Kollegen an den Entscheidungen zu beteiligen und den Prozess transparent zu gestalten, gab es im Kollegium Kritik. Die Teambildungskriterien seien nicht transparent. Es wurde der Wunsch geäußert, dass stärker auf gewachsene Teamstrukturen Rücksicht genommen werden sollte. Es ist zu vermuten, dass hier eine Analyse der möglichen Einflussfaktoren auf die Teamarbeit hilfreich bei der Auswahl der Teambildungskriterien sein könnte. Diese müssten dann seitens der Schulleitung explizit formuliert und angewandt werden.

Aus Sicht der Befragten spielte der Austausch und die Verwendung von Materialien zwischen den Schulen keine große Rolle, 1) weil man schulintern gut zusammenarbeitete, 2) weil ausreichend Unterrichtsmaterial intern zur Verfügung stünde, 3) weil die H11 über ein gut funktionierendes schulinternes Lehrertauschverzeichnis verfüge, 4) weil die Anpassung der Materialien anderer Schulen an die eigenen Schüler, die Branche und Rahmenbedingungen viel Aufwand koste und weil 5) die Kommunikation zwischen Kollegen unterschiedlicher Schulen zu viel Zeit in Anspruch nähme und Nachfragen nicht zeitnah beantwortet werden könnten. Wollte man die Kollegen der H11 wieder am schulübergreifenden curricularen Entwicklungsprozess beteiligen, müsste hierfür erneut um Akzeptanz geworben werden.

2.1.4 Positive Aspekte und Problemfelder an der H13

Zum Zeitpunkt der Einführung der Neuordnung gab es auf der Ebene der Schulleitung einige personelle Änderungen. Aus Sicht der Befragten war dies keine günstige Voraussetzung, tief greifende Veränderungen wie die Einführung eines lernfeldorientierten Curriculums einzuleiten. Die mehrheitlich eher älteren Kollegen standen der Neuordnung und dem Lernfeldkonzept tendenziell skeptisch gegenüber. Das habe den Beginn und die erste Phase der Entwicklungsarbeit stark blockiert. Der Schulleiter glaubte, dass der Entwicklungsprozess leichter gewesen wäre, würde man zum jetzigen Zeitpunkt mit der Arbeit beginnen. Da gerade ein Generationenwechsel stattfand, träfe man auf eine günstigere Motivationslage im Kollegium. Die neuen jüngeren Kollegen seien offener gegenüber dem Lernfeldkonzept.

Nach Wahrnehmung des Schulleiters hat sich seit Einführung der Neuordnung die Bereitschaft, in Teams zu arbeiten, deutlich erhöht. Das Arbeiten in Klassenlehrerteams scheint noch nicht etabliert. Aber aus Sicht des Schulleiters sei prinzipiell eine große Kollegialität vorhanden. Die neuen Kollegen würden gut eingearbeitet und von den Kollegen mit Unterrichtsmaterial versorgt. Der Schulleiter unterstütze dieses kooperative Klima.

Die Frage, was für die Kollegen ein verbindliches Curriculum darstelle, konnte bisher nicht geklärt werden. Aus Sicht des Schulleiters hatten die im Zuge der Kooperation erstellten Dokumente wahrscheinlich keinen verbindlichen Charakter. Er vermutete, dass die schulinterne Tauschbörse genug gutes Unterrichtsmaterial böte, aus dem sich die Kollegen bedienen könnten. Er ging davon aus, dass überwiegend kompetenzorientierter Unterricht stattfindet, dass also ein Mentalitätswechsel vom alten zum neuen Lehrplan vollzogen wurde. Aus Sicht des Lernfeldkoordinators seien eher prüfungsorientierte Stoffkataloge handlungsleitend.

Die eher negativen Erfahrungen aus dem Entwicklungsprozess aufgrund der Arbeit mit einer großen Gruppe wirkten insofern nach, als die Bereitschaft zur weiteren curricularen Arbeit verhalten war. Es sollte daher eine andere Arbeitsstrategie verfolgt werden. Das Etablieren von kleineren curricularen Arbeitsgruppen verbunden mit einer Implementationsstrategie, die den Transfer von Material und Erfahrungen sicherstellte, wäre nachhaltiger als das Vertrauen darauf, dass sich aufgrund natürlicher Personalfluktuationen ein positiveres Klima einstellt, das automatisch für die Durchsetzung des Lernfeldgedankens sorgte.

Die H13 hat aufgrund ihrer verfügbaren Gebäude erhebliche Probleme, die nötigen räumlichen und technischen Voraussetzungen für den Lernfeldunterricht zu schaffen. Lösungen mit transportablen Laptopwagen stellen hohe Anforderungen an die

Kollegen. Während jedoch zu Beginn des Entwicklungsprozesses diese Rahmenbedingungen noch die Motivation zur curricularen Arbeit erheblich beeinflussten (warum sollen wir Konzepte für Lernfeldunterricht erarbeiten, wenn dieser aufgrund fehlender Rahmenbedingungen sowieso nicht stattfinden kann?), scheint nun das Bemühen zu überwiegen, aus den Gegebenheiten das Beste zu machen. Über die Pläne der Schulleitung zur Verbesserung der räumlichen, materiellen und technischen Rahmenbedingungen sollten auch Möglichkeiten geprüft werden, die Klassengrößen möglichst gering zu halten.

2.1.5 Sichtung der Produkte der curricularen Entwicklungsprozesse

Die Arbeitsergebnisse des Entwicklungsprozesses bis Januar 2007 wurden in WiBeS eingepflegt. Die Lernfeldkoordinatoren legten einen Ordner in WiBeS an, in dem die Dokumente strukturiert abgelegt wurden. Die Arbeitsergebnisse hatten sehr unterschiedliche Inhalte. Es gab Arbeitsblätter für den Unterricht, Informationsblätter für Schüler, Ablaufpläne für Unterrichtseinheiten, Strukturplanungen für Lernfelder, Beschreibungen von komplexen Lernsituationen, Kompetenzbeschreibungen, Vorschläge für Klassenarbeiten usw. Diese Dokumente wurden mit Hilfe von Dateiattributen sortiert. Zum Zeitpunkt der Sichtung durch das IBW fanden wir etwa 750 Dokumente, die unter 13 unterschiedlichen Attributen abgelegt waren. Folgende Abbildung zeigt beispielhaft die Struktur des WiBeS-Ordners, links sind die Dateiattribute angegeben:

Ordnerstruktur WiBeS mit Attributen

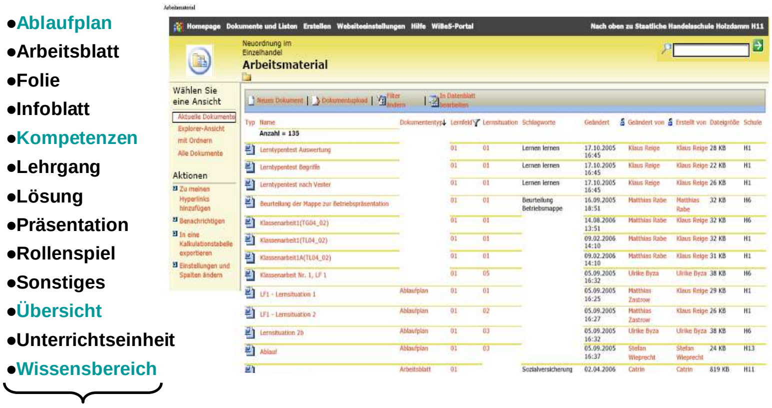

Dateiattribute: Die Blauen sind vom IBW analysiert worden

Abbildung 3: Ordnerstruktur auf der WiBeS-Plattform

Da es nicht möglich war, alle 750 Dokumente durchzusehen, konzentrierten wir uns auf diejenigen, die auf eher konzeptionelle Inhalte schließen ließen, nämlich Ablaufpläne, Kompetenzen, Übersichten und Wissensbereiche. Um die Informationen dieser Dokumente zu ordnen nutzen wir das Planungsformat, dass in Kapitel 3 erläutert wird. Mit Hilfe dieses Instrumentes konnten wir eine Bestandsanalyse durchführen, indem wir die Informationen Lernfeld für Lernfeld in dieses Format überführten. So konnten wir den für die Lernfelder zuständigen Schulen rückmelden, an welchen Stellen unserer Meinung nach Überarbeitungsbedarf vorlag.

2.2 Prozessanalyse

In Abschnitt 2.2.1. beschreiben wir zwei Modelle zur Organisation der Entwicklungsarbeit von April 2004 bis zum Beginn des Projektes, die wir in den vier Schulen vorfanden. Dabei wird deutlich, welche Stärken und Schwächen diese Organisationsformen jeweils hatten. In Abschnitt 2.2.2 erklären wir, worin die schulübergreifende Kooperation bezogen auf den curricularen Entwicklungsprozess bestand und wo wir Defizite sahen. In Abschnitt 2.2.3 schließlich erläutern wir, warum die curricularen Produkte aus unserer Sicht einer weiteren Überarbeitung bedurften.

2.2.1 Analyse der schulinternen Entwicklungsarbeit

In der Abbildung 4 sind zwei Modelle dargestellt, die sich bei Betrachtung der in den vier Schulen verfolgten Strategie zur Entwicklungsarbeit herauskristallisierten. Beide Modelle sind mit Vor- und Nachteilen behaftet, die kurz beschrieben werden:

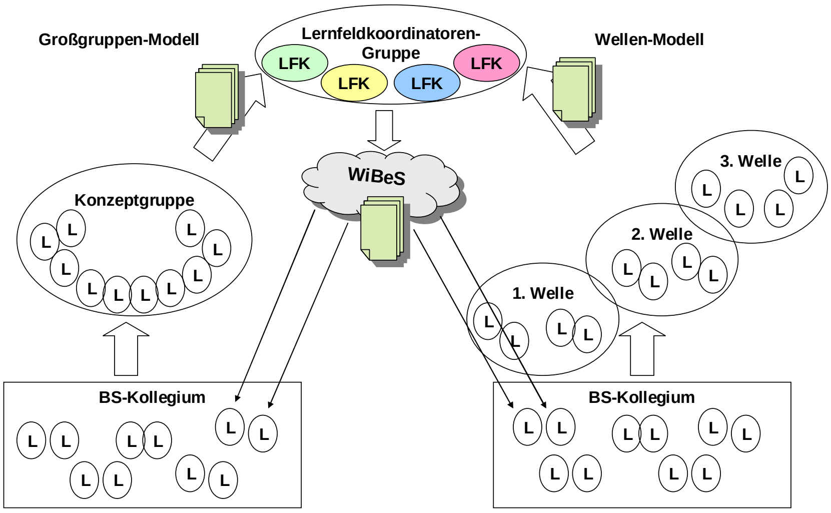

Abbildung 4: Modelle der schulinternen Entwicklungsarbeit

Charakteristisch für das Großgruppenmodell ist, dass möglichst alle Kollegen des Lernbereichs I, also alle, die Lernfeldunterricht durchführen, Mitglieder der Konzeptgruppe sind. Klarer Vorteil ist, dass bereits in der Entwicklungsphase des curricularen Materials möglichst Viele beteiligt sind und deswegen der Transfer von Entwicklern zu Anwendern entfällt. Die Akzeptanz der Produkte dürfte höher sein, wenn alle potenziellen Nutzer bereits bei der Erstellung ihre Ideen einbringen können. Der Nachteil ist, dass mit einer Gruppengröße, die 6-8 Personen übersteigt, konzeptionelles Arbeiten schwierig wird. Bereits die Terminfindung für gemeinsame Sitzungen ist problematisch. Es ist unwahrscheinlich, dass das Interesse und das Engagement aller Beteiligten gleich sind, sodass es immer Teilnehmer gibt, die sich eher zurückhalten. Diese Probleme wurden von einer der vier Schulen als sehr hemmend für die curriculare Entwicklungsarbeit beschrieben. Das Bemühen, Konsens über die curricularen Produkte zu erlangen, um die Akzeptanz zu gewährleisten, schlug fehl, weil die Aushandlungsprozesse aufgrund der Gruppengröße und Heterogenität sehr zäh waren und die Motivation auch der engagierten Kollegen verringerte. Schließlich kam den Lernfeldkoordinatoren der Schulen, die dieses Großgruppenmodell für ihre konzeptionelle Arbeit wählten, eine Schlüsselrolle zu. Sie waren die Moderatoren der Konzeptgruppe, nahmen Diskussionsbeiträge auf und haben überwiegend selbst die Materialien

ausgearbeitet. In diesem Modell bekamen alle Kollegen der Konzeptgruppe die gleichen Entlastungsstunden gut geschrieben. In Anbetracht des unterschiedlichen Engagements waren diese Ressourcen nicht durchgängig effektiv eingesetzt.

Charakteristisch für das Wellen-Modell ist, dass sich die Konzeptgruppen zur Bearbeitung eines Lernfeldes neu bildeten. Es fanden sich die Kollegen, die entsprechende fachliche Kompetenzen zur Bearbeitung des Lernfeldes einbringen konnten oder die davon ausgingen, dass sie gut miteinander arbeiten könnten. Beides ist förderlich für die Arbeitsatmosphäre aber auch für die Bildung von Expertentum. Die Schulen, die dieses Modell wählten, hatten das Transferproblem zu lösen. Sie mussten Strategien entwickeln, wie die erarbeiteten Materialien für alle zugänglich, versteh- und anwendbar gemacht werden können. Dies war in der Regel die Aufgabe der Lernfeldkoordinatoren, die zu diesem Zweck Konferenzen oder ähnliche Veranstaltungen organisierten. Da immer nur diejenigen Entlastungsstunden erhielten, die in einer Konzeptgruppe mitarbeiteten, waren die Ressourcen effektiv eingesetzt.

2.2.2 Der kooperative Entwicklungsprozess der vier EH-Schulen

Der zentrale Gegenstand der schulübergreifenden Kooperation war die Verabredung, welche Schule welches Lernfeld zu bearbeiten hatte. Es wurde vereinbart, dass die Lernfelder sukzessive zu entwickeln wären. Im ersten Schuljahr nach Inkrafttreten der Neuordnung wurden die Lernfelder 2 - 5 bearbeitet, im zweiten Schuljahr die Lernfelder 6 - 9 und im dritten Schuljahr die Lernfelder 10 - 13. Die Übergabe der Ausarbeitungen an die je anderen Schulen erfolgte spätestens dann, wenn das Lernfeld im Zuge des ersten Ausbildungsdurchganges nach der Neuordnung unterrichtet wurde.

Die Schnittstelle zum schulübergreifenden kooperativen Prozess war in beiden Modellvarianten (vgl. 2.2.1) identisch: Die ausgearbeiteten Produkte wurden den Lernfeldkoordinatoren übergeben. Die vier Lernfeldkoordinatoren sammelten und sichteten die Dokumente und stellten sie in WiBeS ein. Von dort konnten sich die Kollegen der anderen Schulen die „fremd-erarbeiteten“ Dokumente herunterladen. Ein Rückkoppelungsprozess zwischen den Entwicklern der erstellenden Schule und den Anwendern der abnehmenden Schulen war jedoch nicht vorgesehen. Den Lernfeldkoordinatoren fehlte ebenfalls die Befugnis, den Autoren Rückmeldungen zum Inhalt und zur Qualität der Beiträge zu geben. Ungeklärt war auch, ob und wie Unterrichtserfahrungen mit den Materialien rückgekoppelt oder in die Planungsdokumente eingearbeitet werden sollten. Es gab keine Strategie oder Verabredungen für Zuständigkeiten bezogen auf Überarbeitungsprozesse. Dies führte dazu, dass weder Lob noch Kritik die Entwickler erreichte und dass fehlerhafte Dokumente nicht überarbeitet oder defizitäre Dokumente nicht angepasst wurden. Es wurden nur neue Dokumente in WiBeS eingestellt, nicht aber überkommene gelöscht. Dies führte zu einem inflationären Anstieg der Einträge zu Lasten der Übersichtlichkeit.

Bei der schriftlichen Befragung der Lehrer im Oktober 2007 formulierten wir u. a. die Frage, welche Schwierigkeiten die Lehrer bei der Anwendung des Materials, das in WiBeS hinterlegt war, hatten. Im Folgenden ist die Zusammenfassung der freien Antworten dieses Items aus dem 3. Zwischenbericht vom 31.8.2008 zu lesen:

Auszug aus dem 3. Zwischenbericht – S.35:

„Die genannten Schwierigkeiten beziehen sich zum großen Teil auf die **inhaltliche Ausarbeitung der Lernfelder**. Den Teilnehmern fehlten Angaben zur Relevanz von Lerninhalten bzw. der „rote Faden“. Sie konnten keine Zusammenhänge in der Fülle des Materials erkennen und auch keine Entwicklungslinien. Sie stellten eine inhaltliche Überfrachtung fest und hatten Schwierigkeiten mit der Zuordnung zu einzelnen Lernfeldern, da die Arbeitsblätter eher fächerorientiert ausgearbeitet und abgelegt wurden. Die Befragten beklagten auch, dass aus dem Material keine Struktur über Sequenzierungen zu rekonstruieren sei und dass Angaben zur Vorgehensweise sowie zur Umsetzung fehlten. Es wäre hilfreich, wenn im Material auch methodische Umsetzungshinweise gegeben worden wären. Die Lernarrangements seien langweilig, inhaltlich überfrachtet und nicht in komplexe Situationen eingebettet.

In Hinblick auf die Arbeitsblätter beklagten die Befragten fehlende oder lückenhafte Angaben. Es fehlten Lösungen, Literaturangaben und Aufgaben. Die qualitativen und lerntheoretischen Niveaus seien sehr unterschiedlich, sodass immer Anpassungen nötig seien. Anpassungen müsse es geben, weil die Branche nicht passe oder die Eingangsvoraussetzungen der Klasse unterschiedlich seien. Zudem müssen die Materialien zum Unterrichtsstil des Lehrers bzw. des Lehrerteams passen. Aus dem Material sei nicht immer ersichtlich, was eigentlich das Lernziel ist. Es fehlten Angaben zur notwendigen Technik. Bestimmte Materialien ließen sich nicht nutzen, da die technischen und räumlichen Gegebenheiten unzureichend waren.

Einige Befragten äußerten ihren Ärger darüber, dass das Material nicht rechtzeitig zum verabredeten Zeitpunkt eingestellt worden sei. Die in den Schulen hinterlegten Papierordner seien nicht aktuell und lückenhaft, da Kollegen entnommenes Material nicht zurücklegten. Die Nutzung von WiBeS sei ärgerlich, weil der Ordner unübersichtlich, nicht sinnvoll gegliedert und viel zu überfrachtet sei. Generell sei die Technik unkomfortabel.“

Diesen Äußerungen sind bereits eine Reihe von Hinweisen auf Defizite der curricularen Produkte zu entnehmen, die wir in folgendem Abschnitt erläutern.

2.2.3 Analyse der curricularen Produkte

In Abschnitt 2.1.5 haben wir erklärt, wie wir uns einen Überblick über die erarbeiteten curricularen Materialien verschafft haben. Wir interessierten uns dabei nicht für Arbeitsblätter zum Einsatz in bestimmten Unterrichtseinheiten und auch nicht für deren Ablaufpläne, sondern für konzeptionelle Überlegungen auf einer didaktischen Makroebene. Wir suchten nach Ergänzungen der KMK-Vorgaben in Form von Lernzielbeschreibungen, Schwerpunktsetzungen in den Lernfeldern, Hinweisen dazu, welche Art von Wissen in welchen Inhaltsbereichen erzeugt werden soll, wie und welche Kompetenzen in Lernsituationen zu entwickeln sind und in welcher Reihenfolge diese Lernsituationen sinnvollerweise zu unterrichten wären. Um diese Informationen zu strukturieren, wurde das von Tramm im Rahmen des Projekts Lerne*MFA entwickelte Planungsformat genutzt (vgl. www.lerne-mfa.de).

Wir fanden heraus, dass für viele Lernfelder Informationen zu den zu entwickelnden Kompetenzen fehlten oder in einer Form strukturiert waren, die wenig hilfreich für eine didaktische Planung ist. Häufig fanden wir Aufzählungen von Lernzielen, die nach Fach-, Methoden-, Sozial- und Persönlichkeitskompetenzen sortiert waren. Diese Strukturierung erwies sich als unübersichtlich, zusammenhanglos und zu

unspezifisch, was deren Nutzen für die Unterrichtsplanung einschränkte. Ebenso verhält es sich mit Inhaltslisten nach dem Vorbild von Prüfungskatalogen, insbesondere weil sie keinen Hinweis auf die Art der Wissensstruktur bzw. das lerntheoretische Niveau liefern. Diese Defizite führten dann zu den berechtigten Klagen über unzureichende Orientierungsleistung der Planungsdokumente für die Unterrichtsvorbereitung.

Die Orientierungsleistung der Materialien war zusätzlich dadurch begrenzt, dass die Lernfelder unverbunden bearbeitet wurden. Es fehlte die lernfeldübergreifende Perspektive, so dass die Lehrer zu Recht beklagten, dass Inhaltsbereiche doppelt vorkamen, dass Bereiche ausgelassen wurden, dass keine Verlässlichkeit in Bezug auf in vorherigen Lernfeldern Gelerntes bestünde. Die Idee eines Spiralcurriculums muss ihren Niederschlag in den Planungsunterlagen finden, indem Querverweise auf die vor- und nachgelagerten Lernfelder gemacht werden. In diesem Sinne haben wir Äußerungen verstanden, die mit dem Begriff „es fehle der rote Faden“ zusammengefasst wurden.

Es war nicht nur die Masse und die Vielfalt der Dokumente, die die Motivation der Anwender schwinden ließ, sondern auch die Qualitätsunterschiede der Dokumente. Überarbeitungen und Verbesserungen wurden häufig nicht schulübergreifend verfügbar gemacht, sodass keine Verlässlichkeit über die Richtigkeit oder Aktualität bestand. Ebenso ungeklärt war die Frage nach der Verbindlichkeit der Materialien. Bis zu welcher Planungsebene sollten bzw. mussten die Beteiligten die curricularen Materialien für ihren Unterricht nutzen? Hierzu wurden zu keinem Zeitpunkt eindeutige Hinweise gegeben.

2.3 Empfehlungen für die kontinuierliche Weiterentwicklung

Die Befunde der Prozessanalysen führten zur Mitte der Projektlaufzeit zu Empfehlungen, die mit den Schul- und Abteilungsleitern sowie den Lernfeldkoordinatoren diskutiert wurden. Ergebnis dieses Austauschs war eine Verlagerung der Projektziele und -schwerpunkte. Dies wird im Folgenden nachgezeichnet, indem die Empfehlungen anhand der auf die Evaluationsbereiche bezogenen Befunde noch einmal kurz zusammengefasst werden.

2.3.1 Empfehlungen bezogen auf die schulinterne Entwicklungsarbeit

Bezogen auf die schulinternen Entwicklungs- und Implementationsprozesse	
Befunde	➢ Nach Auflösung der Konzeptgruppen fehlen Feedbackschleifen<br>➢ Entwicklungsprozesse nur noch auf privater, informeller Ebene; keine Strukturen, um aktuelle Prozesse zu steuern<br>➢ Ohne Steuerung drohen neue wertvolle Prozesse zu versiegen
Empfehlung	**Revitalisierung** des Entwicklungs- bzw. Implementationsprozesses durch<br>➢ Modifikation, Aktualisierung und Weiterentwicklung der vorliegenden Produkte mit Hilfe von **Planungsformaten**<br>➢ **Steuergruppe** einsetzen:<br>- Bündelung und Koordination der derzeitigen Prozesse<br>- Initiierung neuer Prozesse (Teamarbeit)<br>- Herstellen von Informations- und Kommunikationsstrukturen<br>➢ Entwicklung einer **Feedbackkultur** um einen dauerhaften inhaltlichen Diskurs über die Materialien sicherzustellen<br>➢Organisation der **Kommunikation in das Netzwerk**

Tabelle 7: Befunde bezogen auf die schulinternen Entwicklungs- und Implementationsprozesse

Nach der Entwicklungsphase von 2004 bis 2007 wurden die schulinternen Konzeptgruppen, die die Lernfelder in verabredeter Weise nacheinander

bearbeiteten, aufgelöst. Die Ressourcenbereitstellung in Form der Freigabe von Stundendeputaten wurde eingestellt. Die Arbeitsergebnisse der Konzeptgruppen wurden sowohl in WiBeS als auch in schulinternen Laufwerken eingestellt, Teile der Ergebnisse wurden vereinzelt auch bei Lehrerkonferenzen vorgestellt. Sowohl die Weiterarbeit an den Produkten als auch die Aufnahme der Rückmeldungen anderer Kollegen fand nur in informellen bzw. privaten Rahmen statt. Rückkoppelungsprozesse in Bezug auf Unterrichtserfahrungen oder Überarbeitungen und Aktualisierungen der Dokumente waren weder organisiert noch strukturell im Schulalltag verankert. Somit drohten gerade erst auflebende Prozesse zu versiegen.

Die Empfehlung lautete, diese kommunikativen Prozesse zu **revitalisieren**. Dazu sollte das **Planungsformat** (vgl. Kapitel 3) genutzt werden, um bereits vorhandene Beiträge einzuordnen und auf der Grundlage eines einheitlichen Formats über die Materialien zu kommunizieren. Die curriculare Entwicklungsarbeit entlang des Planungsformates sollte helfen, die Lernfelder vollständig abzubilden, d. h. Prozesse, Sequenzierungen, curriculare Interpretationen und Kompetenzen auseinanderzuhalten, sukzessive zu entwickeln und aufeinander zu beziehen. Diese anspruchsvolle Tätigkeit ist nur im Team zu leisten und muss gesteuert werden. Deswegen empfahlen wir den Einsatz von schulinternen **Steuergruppen**, die die einzelnen schulischen Teams betreuen, die Entwicklungsprozesse begleiten und die Arbeitsergebnisse bündeln. Die Ergebnisse müssten dann an die Kollegen weitergegeben und Erfahrungen rückgekoppelt werden, damit sie wieder in den Entwicklungsprozess einfließen. Hierzu muss sich eine **Feedbackkultur** etablieren, die einen dauerhaften inhaltlichen Diskurs über die Materialen erlaubt. Schließlich müssten die Arbeitsergebnisse jedem Interessenten permanent und verlässlich zugänglich sein. D. h. es muss sichergestellt sein, dass in **schulischen Netzwerken** aktuelle und gültige Dokumente hinterlegt sind.

2.3.2 Empfehlungen bezogen auf die schulübergreifende Kooperation

Bezogen auf die Prozessebene der schulübergreifenden Kooperation	
Befunde	➢ Arbeitsteilung hat nur formal geklappt<br>➢ LFK-Gruppe ohne ausreichende Kompetenzzuweisung und ohne Legitimation zur konstruktiven Rückmeldung<br>➢ WiBeS: Keine verlässlichen Aktualisierungen, nur noch wenig Zugriffe.<br>➢ außerhalb der Koordinatorengruppe keine Foren zum Erfahrungsaustausch (insbesondere keine Präsenztreffen)<br>➢ Kein schulübergreifendes Konzept zum Austausch von Implementationserfahrungen oder zur Verständigung über gemeinsame Strategien
Empfehlung	➢ Revitalisierung durch Formulierung **klarer Zielstellungen** und curricular-konzeptioneller **Orientierungspunkte**. Einführung von **Projektstrukturen**<br>➢ Arbeit der **curricularen Entwicklungsgruppe** mit **wissenschaftlichen Begleitung** dauerhaft mit entsprechenden **Ressourcen** ausstatten (Wertschätzung)<br>➢ **Anbindung** der schulinternen Prozesse an das Netzwerk über Koordinatorengruppe<br>➢ Organisation von **Präsenztreffen** mit thematischem Fokus<br>➢ Einbindung einer **Internetseite** mit Verlinkung zum WiBeS-Material

Tabelle 8: Befunde bezogen auf die Prozessebene der schulübergreifenden Kooperation

Die Befunde in Hinblick auf die schulübergreifende Kooperation bezogen sich auf die fehlenden oder defizitären Informations- und Kommunikationsstrukturen. Die chronologische Abarbeitung der Lernfelder wurde arbeitsteilig organisiert. Diesem kooperativen Prozess fehlten Feedbackschleifen, weshalb die Arbeitsteilung nur formal stattfand. Es ging faktisch eher darum, die erarbeiteten Materialien rechtzeitig

in WiBeS einzustellen und nicht darum, sich gegenseitig zur Qualität der Produkte oder zu den Erfahrungen mit den Produkten Rückmeldungen zu geben. Die Lernfeldkoordinatoren verwalteten den virtuellen Raum. Sie waren nicht dazu berechtigt, die Dokumente zu bewerten oder zu selektieren. In Folge dessen gab es weder konstruktiven noch auch nur kritische oder zustimmenden Rückmeldungen an die Autoren.

Natürlich fanden Überarbeitungen zu den eingestellten Dokumenten statt. Aber diese Verbesserungen, Ergänzungen und Aktualisierungen wurden größtenteils nur in schulinterne Netzwerke eingespielt, die Einstellungen in WiBeS nicht gekennzeichnet oder die veralteten Dokumente nicht gelöscht. Dies führte dazu, dass auf den schulübergreifenden curricularen Ordner immer weniger zugegriffen wurde, da er weder aktuell noch verlässlich war.

Ein schulübergreifender Austausch im Sinne eines inhaltlichen Diskurses fand nur auf der Ebene der Lernfeldkoordinatoren statt. Die Betroffenen, d. h. die Autoren der Dokumente bzw. die Lehrer, die die Konzepte im Unterricht einsetzten und dort ihre Erfahrungen machten, hatten kein schulübergreifendes Forum zum Austausch.

All dies wären Aspekte einer Verknüpfung der Entwicklungs- und Implementationsprozesse gewesen. Die Frage an die Schul- und Abteilungsleiter, ob es einen schulübergreifenden Diskurs oder gar Planungen zu Implementationsstrategien gab, wurde konsequenterweise verneint.

Auch bezogen auf die schulübergreifende Kooperation empfahlen wir eine **Revitalisierung** der curricularen Entwicklungsarbeit. Dies benötigt zunächst eine klare **Projektstruktur** für die schulübergreifende Kooperation, also das Formulieren klarer Ziele und Orientierungspunkte und Vorstellungen über die Arbeitsstrategien. Die schulübergreifend arbeitenden Gruppen müssen mit **Ressourcen** ausgestattet werden, letztlich auch, um diesem Arbeitsprozess die entsprechende **Wertschätzung** entgegen zu bringen und zu vermeiden, dass sich Eliten absetzen.

Die schulinternen Entwicklungsprozesse müssen über **Steuerteams bzw. Lernfeldkoordinatoren** an den schulübergreifenden Prozess angedockt werden. Das Netzwerk lebt vom **Input und vom Feedback** der schulinternen Gruppen. Da in vier Schulen dieselben Lernfelder und somit sehr ähnliche Unterrichtsideen verwirklicht werden, müssen einzelne Lehrer die Möglichkeit haben, ihre Erfahrungen schulübergreifend auszutauschen. Um vis-a-vis miteinander zu sprechen zu können, sollten regelmäßig **Präsenztreffen** stattfinden, die jeweils einen spezifischen **thematischen Fokus** haben. Schließlich muss es eine **Plattform** geben, auf der sowohl Konzeptpapiere als auch Unterrichtsmaterial **geordnet, strukturiert, verlässlich und aktuell** eingepflegt werden.

2.3.3 Empfehlungen bezogen auf die curricularen Produkte

Bezogen auf curriculare Produkte	
Befunde	➢ Produkte unvollständig, uneinheitliche Qualitätsniveaus und Formate<br>➢ Fehlende lernfeldübergreifende Perspektive; Beitrag der LF zur Kompetenzentwicklung?<br>➢ Fehlende Verbindlichkeit der Produkte
Empfehlung	➢ Erarbeitung lernfeldübergreifender Dokumente:<br>1) verbindlich anzustrebende **berufsspezifische Kompetenzen,**<br>2) Zuordnung **spezifischer Entwicklungsbeiträge** zu einzelnen LF<br>➢ Einführung **einheitlicher Formate** (lernfeldspezifisch an Schulen)<br>➢ **Verbindung** der lernfeldspezifischen mit lernfeldübergreifenden Perspektive: technisch (Internet), organisatorisch (face-to-face), inhaltlich (Intention der Entwickler – Erfahrung der Anwender)<br>➢ **Verbindlichkeit**: Verständigung über den Status der Produkte

Tabelle 9: Befunde bezogen auf curriculare Produkte

Die Analyse der während der Entwicklungsphase von 2004 bis 2007 in WiBeS eingestellten Dokumente ergab, dass die Produkte unvollständig und in Hinblick auf das Qualitätsniveau und die Formate sehr uneinheitlich waren. Durch die strikte Arbeitsteilung der Schulen ging zudem die lernfeldübergreifende Perspektive verloren. Es fehlte ein erkennbarer roter Faden, der sich durch die Lernfelder zog, und dem Lehrer Hinweise auf den Beitrag des Lernfeldes zur beruflichen Kompetenzentwicklung hätte geben können. Es wurden keine inhaltlichen Abgrenzungen zwischen ähnlichen oder angrenzenden Lernfeldern vorgenommen, sodass nicht verlässlich sichergestellt war, auf welche Inhalte oder Kompetenzen ein Lehrer, der ein bestimmtes Lernfeld unterrichtet, zurückgreifen kann. Schließlich gab es keine Verabredungen darüber, ob und in welchem Ausmaß die Dokumente verbindlich sein sollten.

Hieraus leiteten wir folgende Empfehlungen ab: Die Durchsicht und die Überarbeitung und Ergänzung der lernfeldspezifischen Dokumente soll anhand einer **einheitlichen Planungsstruktur** erfolgen. Die Informationen aus den vielen Materialien müssen in ein gemeinsames Format gegossen werden, um festzustellen, an welchen Stellen Informationen fehlen, wo etwas zu verbessern ist und um überhaupt über die Dinge sprechen zu können. Die Flut an Dokumenten muss verdichtet, eine **gemeinsame curriculare Sprache** gefunden und ein Konzept verabredet werden.

Um den **lernfeldübergreifenden roten Faden** zu identifizieren, empfahlen wir eine Entwicklungsgruppe einzusetzen, die das Konstrukt „berufliche Handlungskompetenz“ in ihren Dimensionen beschreibt. Dies sollte nicht nach den Kategorien „Fach-, Methoden- und Humankompetenz“ erfolgen, sondern **domänenspezifisch**, also bezogen auf Sachgebiete, z. B. Kompetenzen bezogen auf rechtliche Normierungen, gesamtwirtschaftliche Rahmenbedingungen oder betriebswirtschaftliche Problemebenen. Darüber hinaus müssen Kaufleute auch persönlichkeitsbezogene oder soziale Kompetenzen entwickeln, somit müssen auch hierfür Kompetenzdimensionen beschrieben werden. Jede dieser Kompetenzdimensionen entwickelt sich über die Lernfelder hinweg, d. h. es muss formuliert werden, wie sich der **einzelne Beitrag der Lernfelder zur Entwicklung der Kompetenz** darstellt.

Parallel hierzu sollen Planungsteams lernfeldspezifisch arbeiten, um die curriculare Funktion, die zugrundeliegenden Arbeits- und Geschäftsprozesse und schließlich die Sequenzierung der Lernfelder zu erarbeiten. **Beide Arbeitsstränge bedingen und**

ergänzen sich gegenseitig, müssen also permanent aufeinander abgestimmt sein. Diese Abstimmung muss technisch, organisatorisch und inhaltlich gesteuert werden. Schließlich müssen sich die beteiligten Akteure darüber einigen, welchen **Verbindlichkeitsstatus** die curricularen Produkte haben werden.

2.4 Neue Projektausrichtung

Diese Empfehlungen führten dazu, dass der Projektschwerpunkt verlagert wurde. Die verbleibende Zeit sollte dazu genutzt werden, nach dem Vorbild des Projektes „Lerne-MFA“ Lehrplanarbeit zu gestalten und zu begleiten mit dem Ziel, ein durchgängiges und schlüssiges Curriculum für die Einzelhandelskaufleute zu entwickeln. Im Berufsbild der Medizinischen Fachangestellten ist im August 2006 ein lernfeldstrukturierter Rahmenlehrplan in Kraft getreten. Bereits drei Jahre vorher begannen Berufsschulen aus dem gesamten Bundesgebiet Möglichkeiten zu klären, die curriculare Entwicklungsarbeit arbeitsteilig und kooperativ zu gestalten und hierfür ein Netzwerk zu gründen. Inzwischen kooperieren 11 Schulen aus 7 Bundesländern in diesem Netzwerk.

Im Vergleich dazu bilden die vier Hamburger Berufsschulen für Einzelhandelskaufleute ein kleines, überschaubares Netzwerk. Gemeinsam ist den Netzwerken jedoch, dass sich die beteiligten Curriculumentwickler über eine gemeinsame Lehrplanstruktur verständigen mussten. Sie brauchen Foren zum inhaltlichen Diskurs und sollen sich in angemessenen Zeitabständen bei Präsenztreffen vis-a-vis über ihre Arbeitsergebnisse austauschen. Da sich das Planungsformat als Struktur für die Kleinarbeitung der KMK-Vorgaben bei dem Projekt LerNe-MFA als hilfreich erwies, entschlossen wir uns, die Kollegien von EvaNet-EH ebenfalls dafür zu gewinnen. Das Ergebnis der Bemühungen ist eine Lernfeld-Kompetenzmatrix, wie sie auf der Homepage des Projekts LerNe-MFA zu sehen ist (www.LerNe-MFA.de). Lehrer, die sich darüber informieren wollen, was in den einzelnen Lernfeldern zu unterrichten ist, können in dieser Matrix umfassende Informationen einholen.

Lernfeld-Kompetenzmatrix von LerNe-MFA

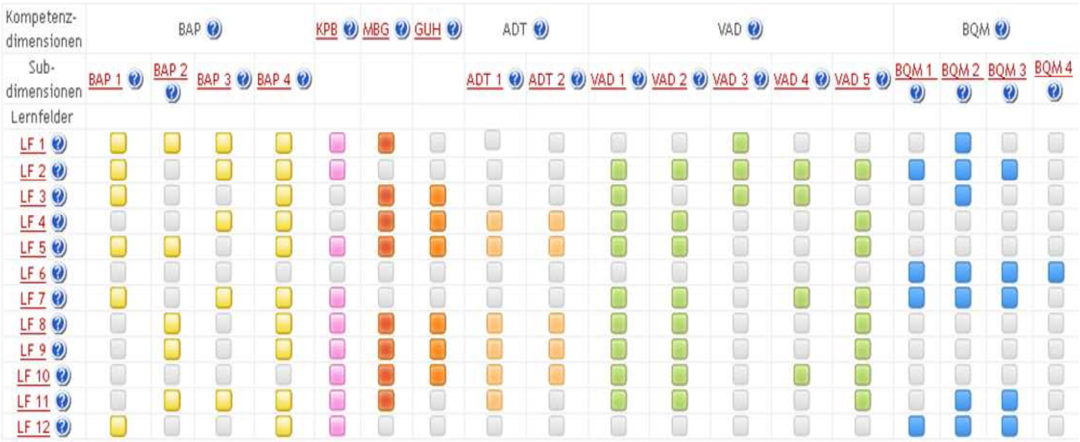

Abbildung 5: Lernfeld-Kompetenz-Matrix von Lerne-MFA

Klickt man auf die einzelnen Lernfelder erhält man Informationen zur curricularen Funktion, also eine Beschreibung dessen, was Aufgabe dieses Lernfeldes im Gesamtcurriculum ist. Man erhält eine Übersicht über die in diesem Lernfeld zu

entwickelnden Kompetenzen, wobei man durch leichtes Navigieren auch herausfindet, in welchem Kontext der Beitrag dieses Lernfeldes zur Kompetenzentwicklung in der jeweiligen Dimension über die Lernfelder hinweg steht.

Die Neuausrichtung des Projektes EvaNet-EH bestand darin, dass auch für die Einzelhandelskaufleute eine Lernfeld-Kompetenz-Matrix entstehen und dass diese in WiBeS für jeden Lehrer zugänglich sein soll. Es wurde vereinbart, dass für jedes Lernfeld zwei Teile des Planungsformats, nämlich die curriculare Analyse und die Strukturplanung, verbindliches Curriculum für die vier Einzelhandelsschulen werden soll. Im dritten Teil, der sogenannten Makroplanung, wird bereits im sehr detaillierten Maße Unterricht geplant, sodass dies mit Rücksicht auf die pädagogische Freiheit und Individualität von Lehrern sowie der Unterschiedlichkeit der Branchen nicht verbindlich sein kann.

Es wurde weiterhin vereinbart, dass die Bearbeitung der Lernfelder wie bisher arbeitsteilig (die Zuweisung der Lernfelder auf die Schulen wurde beibehalten) erfolgt. Schulintern bilden sich zu jedem Lernfeld Planungsteams, die die Planungsformate ergänzen und überarbeiten. Die Planungsteams werden durch das IBW kontinuierlich beraten. Sie werden auf ihre Aufgabe während eines Workshops zum Auftakt dieser Phase vorbereitet. Die Planungsteams organisieren gemeinsam mit den Mitarbeiterinnen des IBW Präsenztreffen, an denen die Ausarbeitungen diskutiert und die Verbindlichkeit verabredet wird.

Parallel zu diesem lernfeldspezifischen Arbeitsstrang wird sich die sogenannte Kompetenzentwicklungsgruppe bilden. Aus jeder Schule werden 1-2 Vertreter zu regelmäßigen Workshops an das IBW eingeladen, um Kompetenzdimensionen zu orten, Subdimensionen und deren spezifische Beiträge zu den Lernfeldern auszudifferenzieren. Beide Arbeitsstränge werden durch das IBW koordiniert.

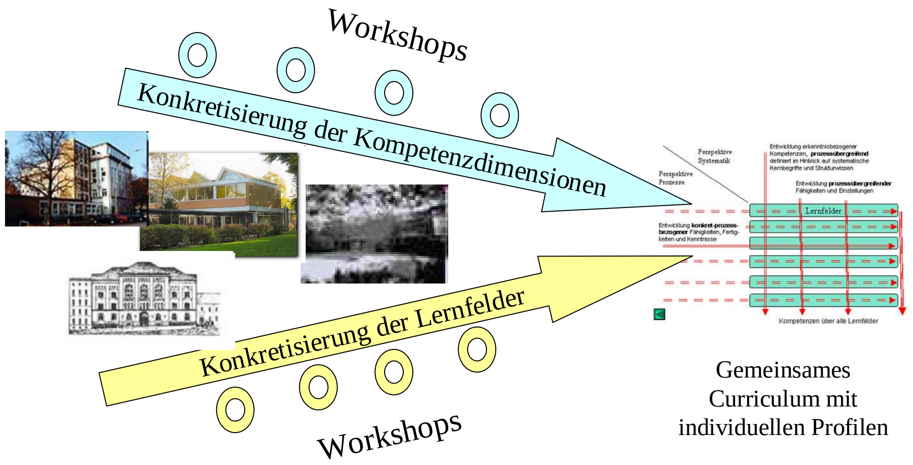

Abbildung 6: Koordination der zwei Arbeitsstränge

3 Analyse und Weiterentwicklung der curricularen Produkte

3.1 Theoriegeleitete Begründung der curricularen Strategie

Die Veränderungen der Ordnungsmittel für berufliche Bildungsgänge auf Grundlage der Handreichung für die Erarbeitung von Rahmenlehrplänen für anerkannte Ausbildungsberufe (KMK 2007, erstmals 1996) bedingen, dass die Aufgabe der Curriculumentwicklung an beruflichen Schulen enorm an Bedeutung gewinnt und von ihren Anforderungen her neu bestimmt werden muss. Das liegt im Wesentlichen an einigen folgenreichen Neuorientierungen, von denen die aus unserer Sicht wichtigsten an dieser Stelle kurz dargestellt werden sollen.

- Die übergeordnete Leitidee beruflicher Bildungsgänge ist die der beruflichen Handlungskompetenz, die sich ausdifferenziert in Fach-, Sozial- und Selbstkompetenz sowie quer dazu liegend Methodenkompetenz, Lernkompetenz und kommunikative Kompetenz (vgl. KMK 2007, S. 10-11). Mit der Handlungskompetenz als oberster Zielsetzung wird nicht nur der normative Bezugspunkt von Unterricht, sondern auch die zentrale Legitimierungsmöglichkeit beruflicher Curricula neu bestimmt: den Wissenserwerb und damit den „Lehrstoff“ ins Zentrum der didaktischen Überlegungen zu stellen, ist nicht mehr legitim. Demgegenüber muss fortan das *Können in beruflichen, gesellschaftlichen und privaten Situationen* als Ausgangspunkt und Zentrum aller Überlegungen zum Unterricht gewählt werden und Lerninhalte sind daraufhin zu prüfen, welchen Beitrag sie zum Erwerb dieser Kompetenzen leisten (vgl. Brand, Hofmeister & Tramm 2005, S. 6-7).
- Die Fächerstruktur der Lehrpläne wurde durch eine Strukturierung entlang von Arbeits- und Geschäftsprozessen[1] ersetzt. Folglich ist es Lehrenden nicht mehr möglich, sich primär an der Struktur der Fachwissenschaften zu orientieren, wenn sie Inhalte und Ziele ihres Unterrichts bestimmen. Vielmehr ist gefordert, dass Lehrende sich einen umfassenden Zugang zu den Arbeits- und Geschäftsprozessen der betrieblichen Realität verschaffen und diese mit Hilfe fachwissenschaftlicher Erkenntnisse analysieren und systematisieren. Dies soll schließlich aus einer didaktischen Perspektive geschehen, also mit blick auf die angestrebten Kompetenzen sowie die vorhandenen Lernvorausaussetzungen. In dieser Verbindung von prozess- und situationsbezogenen mit systematischen Analysen wird die Voraussetzung dafür gesehen, Lernsituationen gestalten zu können, die den Erwerb anwendungs- und transferfähiges Wissens und damit beruflicher Kompetenzen ermöglichen. Zudem liegt in dieser Verknüpfung der entscheidenden Unterschied zu betrieblichen Lernprozessen, so dass die Berufsschule, einen spezifischen und wichtigen Beitrag zur beruflichen Bildung leistet, der auf der auf die Reflexion betrieblicher Prozesse und Probleme sowie ein daraus entwickeltes „Tiefenverständnis“ (Biggs 2003)ausgerichtet ist.

---

[1] Zumindest sollte dies idealtypisch der Fall sein. Dass viele Lehrpläne diesem Anspruch nicht umfassend genügen, lässt sich an fachsystematischen Bündelungen in einigen Lernfeldern und für den jeweiligen Prozess irrelevanten Inhalten erkennen. Diese Abweichungen müssen aber nicht zwangsläufig negativ zu bewerten sein, da es gute Gründe für Phasen des systematisch ausgerichteten Unterrichts geben kann. Dies ist aber im Einzelfall zu entscheiden.

- Die Zielbeschreibungen und Inhalte der Rahmenlehrpläne beanspruchen erklärtermaßen keine Vollständigkeit und sind als Mindestanforderungen zu verstehen (vgl. KMK 2007, S. 8). Dementsprechend haben Lehrende selbst die Aufgabe, curricular tätig zu werden. Der Lehrplan bedarf der Interpretation, der Konkretisierung und Ergänzung sowie der erweiterten Strukturierung, um hinreichend Orientierung für die Unterrichtsplanung zu leisten.

Die drei genannten Gründe für die Neubestimmung der curricularen Aufgabe beruflicher Schulen lassen zwei zentrale Ordnungs- und Relevanzprinzipien beruflicher Curricula deutlich werden, nämlich die Wissenschaftsorientierung und die Situationsorientierung. Die Rahmenlehrpläne stellen mit den Lernfeldern die Situationsorientierung, also die Orientierung an betrieblichen Handlungen, Situationen und Prozessen, in den Vordergrund, während es zur fachlichen Systematik heißt, dass es „unverzichtbar (sei), die jeweiligen Arbeits- und Geschäftsprozesse in den Erklärungszusammenhang zugehöriger Fachwissenschaften zu stellen“ (KMK 2007, S. 17). Den didaktischen Grundsätzen im Teil III der Rahmenlehrpläne lässt sich außerdem entnehmen, dass das Konzept der Handlungsorientierung als Verschränkung von Fachsystematik und Handlungssystematik verstanden wird (vgl. KMK 2007, S. 13). Das dritte Strukturierungs- und Relevanzkriterium neben den Fachwissenschaften und der Situation ist nach Reetz und Seyd (2006, S. 228) die Orientierung an der Person (vgl. Reetz & Seyd 2006, S. 228; siehe dazu auch Zimmermann 1977, S. 53-71; Tyler 1973). Die Handreichung der KMK berücksichtigt die Orientierung an der Person, indem festgestellt wird, dass es aufgrund der heterogenen Schülerschaft einer Anpassung der Rahmenlehrpläne in den Ländern bedarf, auch um unterschiedlichen Interessen, Fähigkeiten und Begabungen gerecht zu werden (vgl. KMK 2007, S. 6). „Die Berufsschule kann ihren Bildungsauftrag nur erfüllen, wenn sie diese Unterschiede beachtet und Schüler und Schülerinnen - auch benachteiligte oder besonders begabte - ihren individuellen Möglichkeiten entsprechend fördert“ (KMK 2007, S. 13). Eine Perspektive der Persönlichkeitsentwicklung oder des Kompetenzaufbaus über die Lernfelder hinweg hingegen wird in der Handreichung der KMK hingegen nicht thematisiert. Allenfalls kann man im Ziel der Entwicklung von Handlungskompetenz (als übergeordnete Leitidee der Berufsausbildung) einen Hinweis auf lernfeldübergreifende Entwicklungsprozesse sehen (vgl. KMK 2007, S. 10).

Das Herausarbeiten der drei Strukturierungs- und Relevanzprinzipien verdeutlicht, dass es nicht ausreicht, die Lernfelder als unabhängig voneinander bestehende Handlungssituationen oder Prozesse zu verstehen und didaktisch auszuformen. Sowohl fachwissenschaftliche als auch persönlichkeitsbezogene Aspekte sind auf allen Planungsebenen eines lernfeldorientierten Unterrichts neben den arbeits- und geschäftsprozessbezogenen Gesichtspunkten in angemessenem Maße zu berücksichtigen[2]. Dies bewirkt aber, wie wir zeigen werden, die Notwendigkeit, neben der lernfeldbezogenen zugleich eine lernfeldübergreifende Perspektive einzunehmen. Die Argumentation führt im Folgenden von a) Überlegungen zur Kompetenzentwicklung über b) die Feststellung, dass der im Rahmenlehrplan verankerte Handlungskompetenzbegriff diese Entwicklungsperspektive nur

---

[2] Eine ausführlichere Begründung für die Notwendigkeit systematischer und situationsbezogener Kompetenzen liefert Tramm in seinem Aufsatz „Prozess, System und Systematik als Schlüsselkategorien lernfeldorientierter Curriculumentwicklung“ (Tramm 2003).

unzulänglich abzubilden vermag, c) zum Entwurf eines curricularen Konzeptes, das die drei Prinzipien Wissenschaft, Situation und Persönlichkeit in angemessener Weise aufeinander bezieht.

a) Entwicklungsperspektive mit Bezug auf die drei klassischen curricularen Prinzipien der Wissenschafts-, Situations- und Persönlichkeitsorientierung

Traditioneller **Unterricht in Fächern** erlaubt es dem jeweils Lehrenden, die Entwicklung der Schüler in einem bestimmten Inhaltsbereich (in Abbildung 7 als Entwicklung fachlicher Kompetenzen bezeichnet) im Auge zu behalten. Auch die persönlichkeitsbezogene Entwicklung der Schüler entzieht sich nicht seiner Wahrnehmung, da er die Schüler i. d. R. über einen längeren Zeitraum unterrichtet. Er kann an fachdidaktische und lernpsychologische Erkenntnisse anknüpfen und die Voraussetzungen seiner Schüler berücksichtigen.

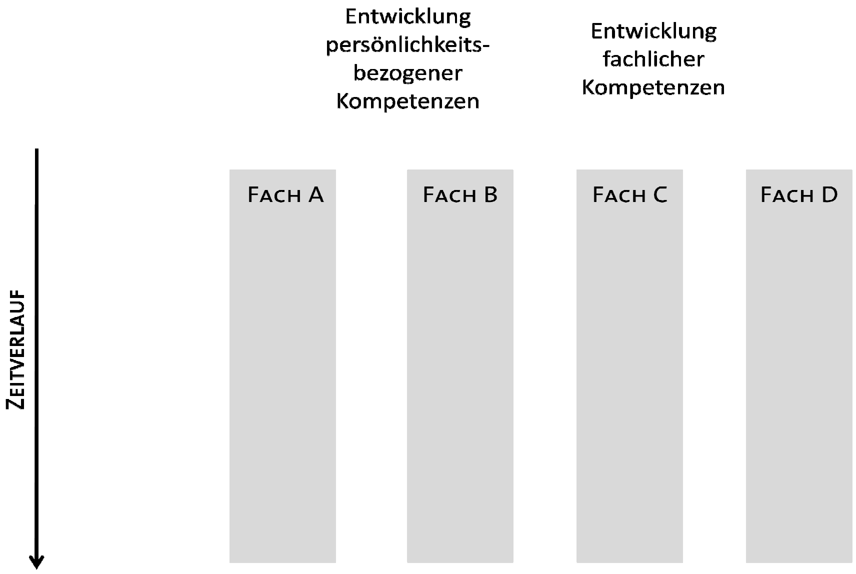

Abbildung 7: Entwicklungsperspektiven fächerstrukturierter Curricula

Die Fächerstruktur ist Ausdruck der Wissenschaftsorientierung von Lehrplänen und die Fächer stehen meist parallel und unverbunden nebeneinander. Das Problem dieser Strukturierung ist, dass sie die Verknüpfung der in den unterschiedlichen Fächern erworbenen Lerninhalte in konkreten lebensnahen Problem- und Anwendungssituationen den Schülern überlässt (vgl. Tramm 2002, S. 25). Durch den Primat der Fächer legt das traditionell strukturierte Curriculum bereits einen fachsystematischen Unterrichtsaufbau nahe. Rechtfertigen müssen sich in diesem Fall diejenigen, die von einer rein fachsystematischen Struktur abweichen. Unter Bezug auf die drei oben beschriebenen Strukturierungs- und Relevanzkriterien führt dies zu einer Vernachlässigung des Situationsprinzips[3].

Lernfeldorientierte Rahmenlehrpläne reagieren auf diese Vernachlässigung, indem sie das Situationsprinzip zum Strukturierungsmerkmal machen.

---

[3] Dieses Defizit kann auch im gefächerten Unterricht durch die Verwendung von Beispielen und Fällen gemildert werden. Mit steigendem Komplexitätsgrad dieser Fälle – und damit wohl auch zugleich mit steigender Realitätsnähe – wächst hier allerdings zugleich die Notwendigkeit einer Behandlung, die die Grenzen des einzelnen Unterrichtsfaches in Richtung auf einen fächerübergreifenden oder gar projektartigen Unterricht übersteigt. .

Ausgangspunkt für ein Lernfeld sind „durch Ziel, Inhalte und Zeitrichtwerte beschriebene thematische Einheiten, die an beruflichen Aufgabenstellungen und Handlungsfeldern orientiert sind und den Arbeits- und Geschäftsprozess reflektieren“ (KMK 2007, S. 17). Unterricht in Lernfeldern muss also von berufsspezifisch relevanten Situationen, Handlungen und Prozessen ausgehen. Im Gegensatz zu fachsystematisch strukturierten Curricula müssen jetzt die Prozesse der betrieblichen Realität betrachtet und zur Grundlage des Unterrichts gemacht werden, was eine genaue Kenntnis des Praxisfeldes erfordert. Im beruflichen Bildungsbereich, dessen kennzeichnende Aufgabe die Berufsqualifizierung ist, liegt die Orientierung an **betrieblichen** Prozessen und Situationen nahe. Die Orientierung an betrieblichen Prozessen intendiert, dass das oben beschriebene Problem, die den Schülern selbst überlassene Verknüpfung unterschiedlicher Inhalte in Anwendungssituationen, behoben wird. Die Lernfelder gleichen als relativ eigenständige curriculare Einheiten Modulen, die nicht erkennbar mit den vor- und nachgelagerten Einheiten verknüpft sind und deren Gesamtarchitektur unklar bleibt. Auf diese Weise entsteht durch das Beheben der einseitigen Wissenschaftsorientierung eine genau entgegengesetzte Problematik. Durch den Primat der Situation und die Förderung situationsbezogener Kompetenzen, geraten fachliche Kompetenzen und persönlichkeitsbezogene Kompetenzen in ihrer über das einzelne Lernfeld hinausgehende Entwicklungsdynamik leicht aus dem Blick. Diese Tendenz wird noch dadurch verstärkt, dass die Lernfelder idealtypisch nacheinander und oft von unterschiedlichen Lehrenden unterrichtet werden.

Abbildung 8 zeigt die dominante Ausrichtung auf des Situationsprinzips im Rahmen lernfeldorientierten Unterrichts. Der Lehrende bearbeitet mit seinen Schülern didaktisch modellierte Situationen, analog solchen, die ihnen im Berufsleben begegnen. In diesen Situationen und Prozessen ist ein fächerverbindender Unterricht notwendig, da sich Situationen und Prozesse genau durch die situative Relevanz ganz unterschiedlicher Inhaltsbereiche kennzeichnen lassen (vgl. Tramm 2003, S. 7-11).

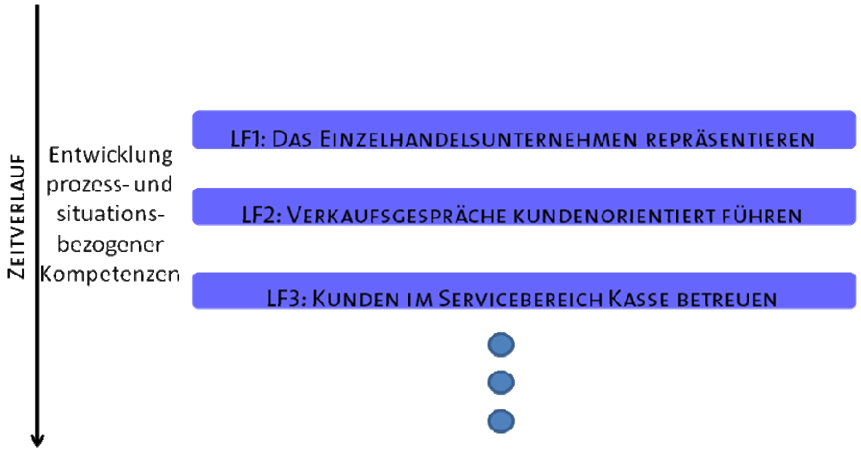

Abbildung 8: Entwicklungsperspektive lernfeldorientierter Curricula

Zusammenfassend ist also festzuhalten, dass die Wissenschaftsorientierung von Curricula dem Lehrenden die Planung und Wahrnehmung fachlicher und persönlichkeitsbezogener Entwicklungsprozesse der Schüler erleichtert. Die Lernfeldorientierung ermöglicht es dem Lehrenden direkt situations- und prozessbezogene Schülerleistungen in den Blick zu nehmen und so an die betriebliche Realität der Schüler anzuknüpfen. Zumeist unbeachtet bleibt aber „die

Problematik des Lernfeldkonzepts, dass nämlich die Überwindung der horizontalen Segmentierung in Fächer erkauft werden könnte durch eine neue, vertikale Segmentierung in unkoordiniert aufeinander folgenden Lernfeldern“ (Tramm, 2009, S. 10).

Mit der lapidaren Feststellung, dass sich aus „der Gesamtheit aller Lernfelder (...) der Beitrag der Berufsschule zur Berufsqualifikation“ (KMK 2007, S. 17) ergibt, verkürzt die KMK die Problematik der Entwicklungsperspektive über die Lernfelder hinweg. Sie unterstellt, dass die Summe der in den Lernfeldern zu erwerbenden situationsbezogenen Kompetenzen den in der Berufsschule zu erwerbenden Teil der Berufsqualifikation ergibt. Dazu, wie sich diese entwickelt und welchen Strukturen eine Entwicklung idealtypisch folgen könnte, äußert sich die KMK nicht, obwohl es unmittelbar einsichtig ist, dass sich beispielsweise kommunikative Fähigkeiten über den gesamten Bildungsgang hinweg entwickeln müssen, von einfachen Verkaufsgesprächen bis hin zu umfassenden Beratungen und Konfliktgesprächen. Weil die Zusammenhänge zwischen den Lernfeldern nicht in den Blick genommen werden, überlassen es lernfeldorientierte Rahmenlehrpläne dem Zufall bzw. dem jeweiligen Lehrerengagement, ob systematische Wissensstrukturen und persönliche Entwicklungen der Schüler gefördert werden (können).

Die Vereinseitigung der lernfeldorientierten Rahmenlehrpläne auf situations- und prozessbezogene Kompetenzentwicklung muss, wie auch von der KMK in der Handreichung gefordert, durch eine Verschränkung von Fach- und Handlungssystematik aufgehoben werden, die in den Rahmenlehrplänen aber nicht per se angelegt ist. Daher besteht die Aufgabe für Schule und Lehrende darin, diese Verschränkung im Rahmen curricularer Entwicklungsarbeit herzustellen, um die fachliche und persönlichkeitsbezogene Schülerentwicklung wieder in den Blick zu bekommen.

b) Handlungskompetenz aus einer lernfeldübergreifenden Entwicklungsperspektive

Um beurteilen zu können, ob der von der Begriff der Handlungskompetenz im Verständnis der KMK mit einer solchen situationsübergreifenden und entwicklungsbezogenen Interpretation vereinbar ist, unterziehen wir ihn im Folgenden einer näheren Betrachtung.

Handlungskompetenz wird von der KMK „als die Bereitschaft und Befähigung des Einzelnen, sich in beruflichen, gesellschaftlichen und privaten Situationen sachgerecht durchdacht sowie individuell und sozial verantwortlich zu verhalten“ (KMK 2007, S. 10), gekennzeichnet. „Handlungskompetenz entfaltet sich in den Dimensionen von Fachkompetenz, Humankompetenz und Sozialkompetenz“ (ebd.), die Methodenkompetenz, Lernkompetenz und kommunikative Kompetenz beinhalten sollen (vgl. KMK 2007, S. 11)[4].

Die in Abbildung 9 ergänzend genannten Ziele der Berufsschule, die der Rahmenvereinbarung über die Berufsschule (Beschluss der Kultusministerkonferenz vom 15.03.1991) enstammen, sind pauschal auf die Entwicklung der

[4] Auf die spezifischen Definitionen der einzelnen Kompetenzen muss an dieser Stelle nicht näher eingegangen werden. Es sei hier lediglich angemerkt, dass sich diese auf einem ähnlich abstrakten Niveau bewegen, wie die Definition der Handlungskompetenz (s. dazu KMK 2007, S. 10-11).

Handlungskompetenz gerichtet (vgl. KMK 2007, S. 10), enthalten aber keine vertiefenden Hinweise dazu, in welchen Schritten sich Handlungskompetenz über die Lernfelder hinweg entwickelt.

> Nach der Rahmenvereinbarung über die Berufsschule (Beschluss der Kultusministerkonferenz vom 15.03.1991) hat die Berufsschule zum Ziel,
>
> "- eine Berufsfähigkeit zu vermitteln, die Fachkompetenz mit allgemeinen Fähigkeiten humaner und sozialer Art verbindet;
>
> - berufliche Flexibilität zur Bewältigung der sich wandelnden Anforderungen in Arbeitswelt und Gesellschaft auch im Hinblick auf das Zusammenwachsen Europas zu entwickeln;
> - die Bereitschaft zur beruflichen Fort- und Weiterbildung zu wecken;
> - die Fähigkeit und Bereitschaft zu fördern, bei der individuellen Lebensgestaltung und im öffentlichen Leben verantwortungsbewusst zu handeln."

Abbildung 9: Ziele der Berufsschule

Einen Hinweis auf die für die KMK leitenden Vorstellungen zur Entwicklung von Handlungskompetenz erhält man aber in den normativen Ausführungen zu den Charakteristika von Lernfeldern. „Im Ziel [‚das für jedes Lernfeld vom jeweiligen Rahmenlehrplanausschuss beschrieben werden soll, Anm. Verf.] wird die Handlungskompetenz, die am Ende des schulischen Lernprozesses in einem Lernfeld erwartet wird, umfassend beschrieben. Dabei wird der didaktische Schwerpunkt und die Anspruchsebene des Lernfeldes zum Ausdruck gebracht“ (KMK 2007, S. 19). Diese Festlegung macht nun deutlich, dass die KMK beim Entwurf ihres Konzeptes entweder davon ausging, dass die situations-/prozessbezogenen Handlungskompetenzen, die in den Lernfeldern beschrieben werden, ausreichend Orientierung böten und in ihrer Summe automatisch eine umfassende berufsspezifische Handlungskompetenz ergäben. Oder aber sie nahm an, dass die jeweiligen Rahmenlehrplanausschüsse in der Lage wären, in einem knapp einseitigen Text für jedes Lernfeld, die Verknüpfungen mit anderen Lernfeldern zu leisten und eine Entwicklungslogik in Form von Anspruchsebenen zu skizzieren. Vielleicht ging sie auch schlicht davon aus, dass die Aufgabe der Verknüpfung der Lernfelder mit Blick auf den Entwicklungsprozess der Schüler von den Lehrenden ohne Weiteres geleistet werden könne.

Keine dieser unterstellten Annahmen hat sich als tragfähig erwiesen. *Automatisch* ergibt sich aufgrund der immer noch relativ abstrakten und komplexen, zudem unvollständigen Zielbeschreibungen der Lernfelder keine Idee zu ihrer Verknüpfungen oder der Kompetenzentwicklung auf Seiten der Schüler. Schon der Umfang der Ziel- und Inhaltsbeschreibung der einzelnen Lernfelder von in der Regel nur wenigen Sätzen und Stichworten für meist zwischen 40 bis 80 Unterrichtsstunden lässt nicht erwarten, dass die Ausschüsse in der Lage wären, Schnittstellen zu anderen Lernfeldern zu beschreiben. Und selbst wenn an Schulen oder von einzelnen Lehrenden erkannt wurde, dass curriculare Entwicklungsarbeit aufgrund der fehlenden Orientierungsleistung der Rahmenlehrpläne notwendig wird, konnten sie ohne entsprechende Anregungen zur Vorgehensweise keine Verknüpfung der Lernfelder untereinander, beispielsweise in Form eines Spiralcurriculums, leisten und keine transparente Entwicklungsperspektive über die Lernfelder hinweg darstellen.

Im Rahmen der kooperativen Curriculumarbeit der vier Einzelhandelsschulen von 2004 bis 2007 waren lernfeldweise zu den drei Kompetenzdimensionen Fach-,

Human- und Sozialkompetenz, teilweise erweitert um Methoden- und Lernkompetenz, Listen von Kompetenzformulierungen verfasst worden, die jedoch von Lernfeld zu Lernfeld sowie von Lernsituation zu Lernsituation i. d. R. in keinerlei Beziehung zueinander standen. Es liegt die Vermutung nahe, dass keine intensive Auseinandersetzung mit Fragen nach der Substanz und Struktur der angestrebten Kompetenzen, nach ihrer jeweiligen Wissensbasis oder nach ihrer Entwicklungslogik stattgefunden hatte (siehe auch Abschnitt 2.2.3). Auch den Fragen danach, über welche sach- und persönlichkeitsbezogenen Kompetenzen in welcher Tiefe die Schüler am Ende ihrer Berufsausbildung verfügen sollen und in welchen Etappen sie diese Kompetenzen im Zeitablauf über die Lernfelder hinweg erwerben könnten, war keine Aufmerksamkeit zugekommen. Auch im Rahmen der schulübergreifenden Curriculumentwicklung, die teilweise durch die Lehrerfortbildungsabteilung des Landesinstituts moderiert wurde, hatte eine Auseinandersetzung mit lernfeldübergreifenden Kompetenzzielen der dualen Berufsausbildung, mit möglichen Kompetenzentwicklungsschritten während des Bildungsganges und die Verbindung der Kompetenzziele mit Lernfeldern und konkreten Inhalten nicht stattgefunden.

Als Belege hierfür können die Ergebnisse unserer Analysen zu den schulinternen und schulübergreifenden Curriculumentwicklungsprozessen in der ersten Projektphase dienen, die wir in den Zwischenberichten I und II des Projekts EvaNet-EH publiziert haben (Hofmeister, Lesch, Steinemann, Tramm 2007; Tramm, Hofmeister, Lesch 2008). Hier wurde deutlich, dass die Lernfelder keine hinreichende Orientierung für Lehrende und für Schüler boten; zugleich war erkennbar, eine Verknüpfung der Lernfelder unter Berücksichtigung der Entwicklungsperspektive ohne ein geeignetes Strukturierungsmodell nicht gelingen konnte. Die globalen Dimensionen der Handlungskompetenz eigneten sich aufgrund ihrer inhaltlichen Unbestimmtheit und funktionalen Hetrogenität nicht, um lernfeldübergreifende Entwicklungsprozesse zu modellieren.

Abbildung 10 zeigt den Versuch, die lernfeldübergreifende Perspektive mit Hilfe der drei Dimensionen[5] der Handlungskompetenz darzustellen. Die Darstellung ist durchaus dazu geeignet, die Leitidee der Handlungskompetenz analytisch abzubilden und zu vergegenwärtigen, dass allen dreien Rechnung zu tragen ist. Jedoch ist diese Dimensionierung der Handlungskompetenz, wie all unsere bisherigen Erfahrungen gezeigt haben, für die Unterrichtsgestaltung wenig orientierend und für die konkrete Curriculumentwicklung an beruflichen Schulen ungeeignet (vgl. z. B. Tramm, Steinemann & Gramlinger 2004). Dies vor allem deshalb, weil sie keine inhaltlichen Orientierungen bietet, sondern auf der Ebene von formalen Leitideen stecken bleibt. Und dass aus Leitideen keine einfache Ableitung konkreterer Zielsetzungen möglich ist, zumindest wenn man den Anspruch erhebt, diese begründen und rechtfertigen zu können, ist bereits eine Erkenntnis der 70er Jahre (vgl. Meyer 1971).

[5] Methoden- und Lernkompetenz sowie kommunikative Kompetenz könnten ergänzt werden.

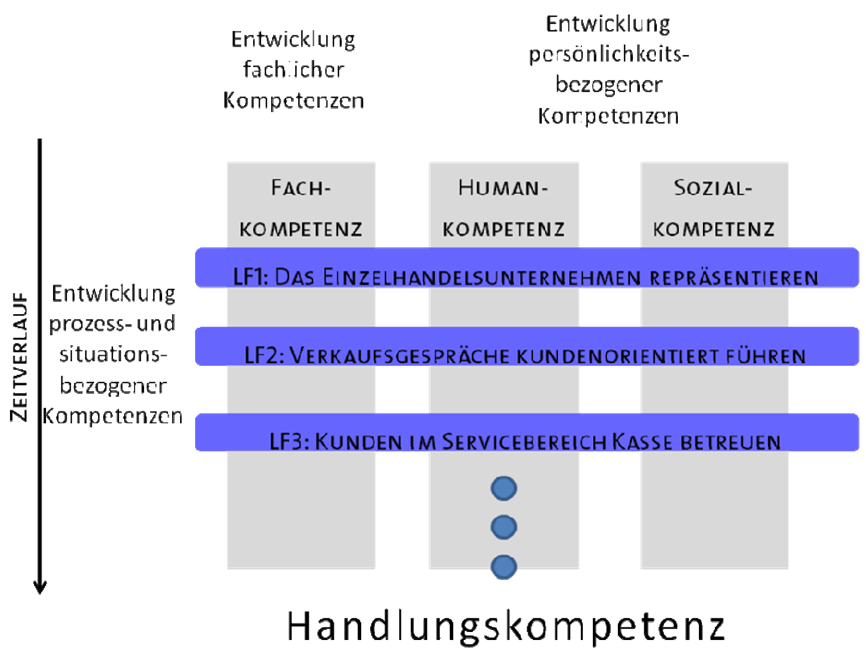

Abbildung 10: Handlungskompetenz als lernfeldübergreifende Entwicklungsperspektive

c) Wiederherstellung der fachlichen und persönlichkeitsbezogenen Entwicklungsperspektive durch lernfeldübergreifende Kompetenzdimensionen

Drei Punkte sind festzuhalten. *Erstens:* Es muss insbesondere eine Verknüpfung von Situations- und Wissenschaftsorientierung hergestellt werden, damit die Schüler zum einen in beruflich relevanten Situationen handlungskompetent sind und zum anderen über strukturiertes, sachbezogenes Wissen verfügen, das ihnen die verstehende Deutung von Situationen, die kritische Analyse von Prozessen und Handlungen oder auch dieBewältigung unbekannter Situationen und Probleme ermöglicht. Daneben muss aber auch die Persönlichkeitsentwicklung in einer curricularen Konzeption Berücksichtigung finden, die die dem Bildungsauftrag der Berufsschule aber auch der wachsenden Bedeutung von „Softskills“ in den Anforderungsprofilen der Wirtschaft gerecht werden will. *Zweitens:* Systematische, sachlogische Wissensstrukturen entwickeln sich kontinuierlich, neues Wissen knüpft an bereits vorhandenes an, revidiert oder verändert dieses, d. h. die Entwicklung sachlogischer Wissenssysteme ist eine Entwicklung, die sich über die gesamte Dauer der Ausbildung erstreckt. Ebenso entwickelt sich die Persönlichkeit der Schüler kontinuierlich. *Drittens:* Das von der KMK verwendete Konzept beruflicher Handlungskompetenz ist in seinen Definitionen und Strukturen inhaltlich unbestimmt, zu allgemein und zu abstrakt, um bei der Konkretisierung lernfeldorientierter Rahmenlehrpläne hilfreich zu sein, die eine lernfeldübergreifende Entwicklung der Schüler abbilden müssten. Deswegen ist eine weiterführende Konzeptualisierung der Lernfeldorientierung und damit verbunden des Konzepts der Handlungskompetenz notwendig. Als einen Beitrag dazu verstehen wir unsere curricularen Entwicklungsarbeiten im Rahmen der praxisbezogenen Projekte[6] seit Beginn der Neuordnung der Ausbildungsberufe.

---

[6] Zu nennen sind an dieser Stelle die Projekte CULIK, LerneMFA, EvaNet-EH, EARA.

Abbildung 11 zeigt die Verknüpfung der situations- und prozessbezogenen Kompetenzentwicklung in den Lernfeldern mit sach- und persönlichkeitsbezogenen Kompetenzentwicklung über die Lernfelder hinweg. Wenn wir von sachbezogenen Kompetenzen (statt von Fachkompetenz) sprechen soll damit betont werden, dass bei der Bestimmung und Bearbeitung der Kompetenzdimensionen nicht die Orientierung an tradierten Fächern oder Fachwissenschaften konstitutiv ist, sondern die Orientierung an sachlogisch unterscheidbaren Inhaltsbereichen, von denen angenommen wird, dass sie berufsspezifisch[7] relevant sind. Auf diese Weise wird also zunächst ein curriculares Modell abgebildet, dass die situative Kompetenzentwicklung in der Horizontalen abbildet und die sach- und persönlichkeitsbezogene Kompetenzentwicklung in vertikaler Richtung. Die vertikal verlaufenden Kompetenzdimensionen sind lernfeldübergreifend als Gesamtentwicklungsziel und lernfeldspezifisch als jeweiliger Beitrag zum Gesamtziel auszudifferenzieren. „Diese Ausdifferenzierung von Kompetenzen dient hauptsächlich der Komplexitätsreduktion und der Strukturierung des curricularen Gestaltungsraumes; sie erfüllt damit eine ähnliche Funktion, wie eine vorgegebene Fächerung. Im Unterschied dazu, beruht sie aber nicht auf Tradition oder administrativer Setzung, sondern rekurriert auf die Analyse der auszubildenden Fähigkeiten, Einsichten und Einstellungen, und ist damit Resultat originär curricular-didaktischer Überlegungen“ (Tramm 2009, S. 11-12).

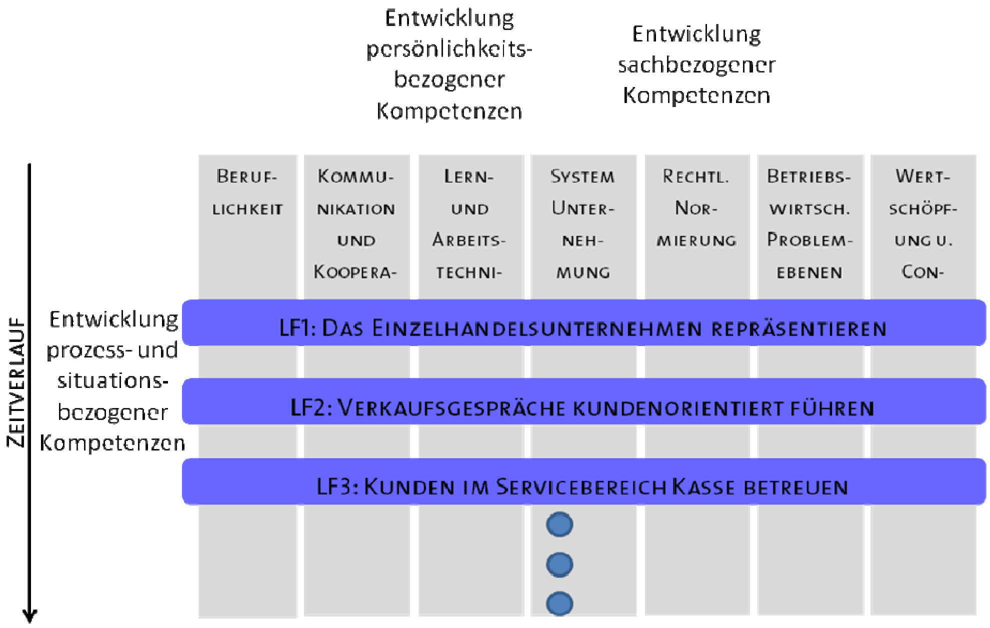

Abbildung 11: Verknüpfung der Entwicklungsperspektiven von Lernfeldern und Kompetenzdimensionen

---

[7] Für die Festlegung der Kompetenzdimensionen wurde zu Beginn des Projektes EvaNet-EH ein Vorschlag gemacht, der im Verlauf der curricularen Weiterentwicklung mehrfach verändert und angepasst wurde. Die Kompetenzdimensionen sind auf den Beruf der Einzelhandelskaufleute bzw. Verkäufer/Verkäuferinnen abgestimmt. Eine Übertragbarkeit weiter Teile des Systems von Kompetenzdimensionen auf andere (kaufmännische) Berufe ist wahrscheinlich und wird derzeit im Rahmen des Projektes EARA überprüft, das sich u. a. mit der Gestaltung und Evaluation eines neu eingerichteten Bildungsganges im Bereich Bürokommunikation befasst.

Jede Kompetenzdimension bildet einen curricular intendierten, spezifischen Entwicklungsprozess über die Lernfelder hinweg ab, der unterrichtlich anzubahnen ist. Dabei ist mit Hilfe didaktischer Analysen und berufsbezogener Rekonstruktionen zu entscheiden, welchen Beitrag das jeweils betrachtete Lernfeld zur Entwicklung in einer spezifischen Kompetenzdimension leistet. Dieser Beitrag kann von „nicht vorhanden" bis „besonders ausgeprägt" variieren (vgl. Tramm 2009, S. 13).

Wo im Lernfeldunterricht diese lernfeldübergreifende Planungsebene nicht realisiert wird, steht dieser in der Tat in der Gefahr, sich im Sinne einer Berufskunde in der Betrachtung einzelner Prozesse zu verzetteln und darüber den Aufbau einer systematischen beruflichen Wissensbasis zu versäumen. Solche Ansätze sind jedoch nicht nur defizitär im Hinblick auf die Wissensbasis beruflicher Kompetenz, sondern stehen zudem in der Gefahr, die Kompetenz- und Persönlichkeitsentwicklung der Schüler aus dem Blick zu verlieren. Dies gilt vor allem dann, wenn Lernfelder durch unterschiedliche Lernfeldspezialisten unterrichtet und als Kette von Modulen durchlaufen werden. Der paradoxe Effekt wäre dann, dass ein Konzept, das angetreten ist, die fachliche Segmentierung des Unterrichts durch die berufliche Prozessperspektive zu überwinden, dazu führte, den Lern- und Entwicklungsprozess der Schüler aus dem Blick zu verlieren.

Abbildung 12 zeigt die Verallgemeinerung des Modells lernfeldübergreifender Kompetenzdimensionen.

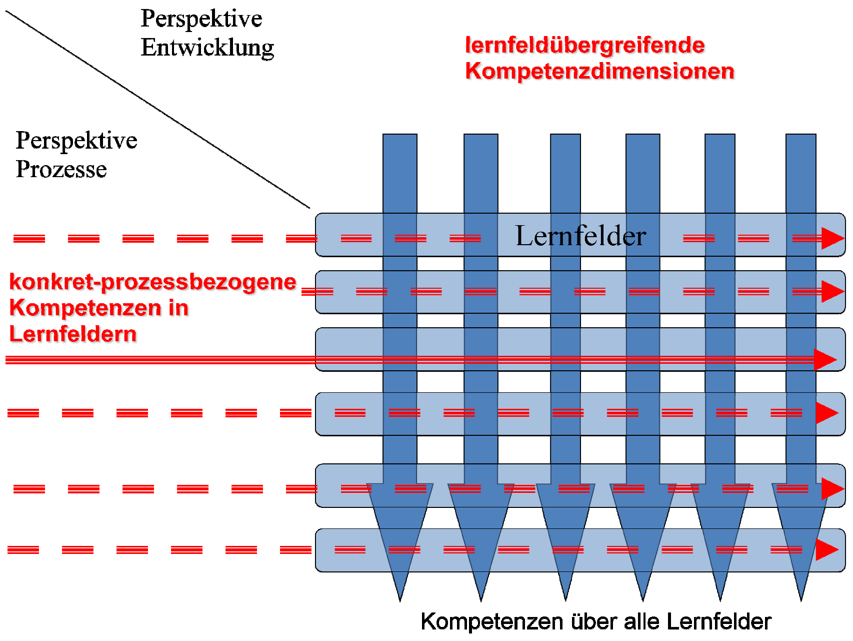

Abbildung 12: Verknüpfung lernfeldspezifischer und lernfeldübergreifender Kompetenzentwicklung (Tramm 2008)

3.2 Prozesse

Im Folgenden werden die auf die curricularen Produkte bezogenen evaluativen (Kapitel 3.2.1) und konstruktiven (Kapitel 3.2.2) Prozesse im Rahmen von EvaNet-EH beschrieben.

3.2.1 Evaluativer Prozess

Die im Zeitraum der schulübergreifenden Kooperation von 2004 bis 2007 arbeitsteilig von Kolleginnen und Kollegen der beteiligten Schulen erstellten curricularen Produkte wurden von uns zunächst gesichtet, geordnet und dann einer kategorialen Inhaltsanalyse unterzogen. Dazu nutzen wir das von Tramm im Rahmen des Projektes Lerne*MFA entwickelte und dort bewährte Planungsformat (vgl. Kapitel 1.2, 2.1.5 und 2.2.3). Das Ergebnis der unter 2.2.3 dargestellten Analyse der curricularen Produkte stellten wir mit einer Übersicht (Abbildung 13) dar, die Auskunft über das Vorhandensein und das Niveau bzw. den Entwicklungsstand der jeweiligen curricularen Dokumente gab. Zusammen mit den Aussagen der schulischen Kollegen zum Stand und zur Qualität der Produkte, war die geringe Orientierungsleistung dieser curricularen Dokumente im Einzelnen wie auch in der Gesamtstruktur evident. Aus diesem Analysebefund resultierte unmittelbar die Neuausrichtung des Projektes EvaNet-EH (vgl. Kapitel 2.3.3 und 2.4), die sich als Wiederaufnahme des konstruktiven Entwicklungsprozesses darstellte. Der Wiederaufnahme der curricularen Entwicklung lag die leitende Idee zugrunde, Curriculumentwicklung als einen kontinuierlichen kooperativen Verbesserungsprozess anzulegen, in dem evaluative und konstruktive Arbeiten iterativ aufeinander zu beziehen waren.

Konzeptionelle Produkte der Entwicklungsarbeit im Rahmen der schulübergreifenden Kooperation

I Curriculare Analyse

Kompetenzdefinition, Inhalte und Zeitrichtwert nach KMK-RLP: *Text aus RLP*

RLF1	RLF2	RLF3	RLF4	RLF5	RLF6	RLF7	RLF8	RLF9	RLF10	RLF11	RLF12	RLF13	RLF14

Curriculare Funktion: *Aufgabe des Lernfeldes im Curriculum*

CLF1	CLF2	CLF3	CLF4	CLF5	CLF6	CLF7	CLF8	CLF9	CLF10	CLF11	CLF12	CLF13	CLF14

Zentrale Prozesse, relevante Prozessvarianten und berufliche Problemkontexte: *Kurze sequenzielle Beschreibung des Kernprozesses. Auflistung von Prozessvarianten, die besonders praxisrelevant oder von hoher systematischer Bedeutsamkeit sind*

PLF1	PLF2	PLF3	PLF4	PLF5	PLF6	PLF7	PLF8	PLF9	PLF10	PLF11	PLF12	PLF13	PLF14

Kompetenzen: *Zu erwerbende Fähigkeiten, Erkenntnisse und Einstellungen und Hinweis auf das angestrebte Kompetenzniveau*

KLF1	KLF2	KLF3	KLF4	KLF5	KLF6	KLF7	KLF8	KLF9	KLF10	KLF11	KLF12	KLF13	KLF14

Wissensbasis: *Faktenwissen, Konzeptuelles Wissen und operatives Wissen Struktur und Schlüsselkonzepte*

WLF1	WLF2	WLF3	WLF4	WLF5	WLF6	WLF7	WLF8	WLF9	WLF10	WLF11	WLF12	WLF13	WLF14

Beitrag zum lernfeldübergreifenden Kompetenzerwerb:

ULF1	ULF2	ULF3	ULF4	ULF5	ULF6	ULF7	ULF8	ULF9	ULF10	ULF11	ULF12	ULF13	ULF14

II Strukturplanung

Szenario (situativer Rahmen des Lernfeldes): *Beschreibung der Ausgangssituation, Ort, Personen, Handlungsperspektive*

SLF1	SLF2	SLF3	SLF4	SLF5	SLF6	SLF7	SLF8	SLF9	SLF10	SLF11	SLF12	SLF13	SLF14

Teilsequenzen (Aufgabe/ Problemstellung im Szenario)	Curriculare Funktion/ Kompetenz-schwerpunkt	Kommentar	Zeit-budget
1 *Ausgangsproblematik und Aufgabenstellung für die Lernenden*	1 *was soll erreicht werden, was ist der intentionale Schwerpunkt, wo liegt der inhaltliche Akzent*		
2 *Variation der Ausgangsproblematik, neue oder ergänzende Aufgabenstellung*	2 *s.o.*		
3 *Variation oder Störung*	3 *s.o.*		
4	4		

TLF1	TLF2	TLF3	TLF4	TLF5	TLF6	TLF7	TLF8	TLF9	TLF10	TLF11	TLF12	TLF13	TLF14

Legende rot: kein Material vorhanden
orange: Material nur rudimentär vorhanden
gelb: Material muss überarbeitet werden
grün: Material liegt vor

bspw. PLF1: Prozessbeschreibung zu Lernfeld 1

Abbildung 13: Übersicht der Produktanalyse

3.2.2 Konstruktive Prozesse

Die revitalisierte Entwicklungsarbeit erfolgte entsprechend der im Kapitel 3.1 dargestellten theoretischen Überlegungen zur Verzahnung situativer und entwicklungsbezogener Aspekte in zwei Strängen. Die an den vier Schulen eingerichtenen Planungsteams widmeten sich der weiteren Ausarbeitung bzw. Überarbeitung oder Revision der lernfeldspezifischen Planungen, während eine kleine Gruppe von Lehrenden in Kooperation mit der Wissenschaftlichen Begleitung als „Kompetenzentwicklungsgruppe„ lernfeldübergreifenden Kompetenzdimensionen ausdifferenzierte (vgl. Kapitel 2.3.3 und 2.4). Diese Prozesse sollten in die Erstellung einer Lernfeld-Kompetenz-Matrix münden (vgl. Kapitel 3.3.3).

Von entscheidender Bedeutung war in diesem Zusammenhang, dass diese zwei personell unterschiedlich besetzten Arbeitsstränge, der lernfeldspezifische und der lernfeldübergreifende, kontinuierlich miteinander verknüpft wurden. Dies geschah

einerseits durch die Aufnahme der Ergebnisse der Kompetenzentwicklungsgruppe in die lernfeldbezogenen Planungen und die Diskussion dieser Planungen auf einer Reihe von Präsenztreffen, an denen ein Großteil der Kollegien teilnahm. Andererseits wurden beide Arbeitsstränge inhaltlich und organisatorisch durch die Mitarbeiterinnen des IBW begleitet, die somit Einblick in den gesamten Entwicklungsprozess hatten. In ähnlicher Weise hatten daneben nur noch die Lernfeldkoordinatoren einen Einblick in beide Arbeitsstränge.

Die Arbeit der Kompetenzentwicklungsgruppe war also kein unabhängig von der Lernfeldentwicklung bestehender Arbeitsstrang, sondern nahm die Ergebnisse der Lernfeldbearbeitungen kritisch-konstruktiv auf und spiegelte die eigenen Arbeitsergebnisse zeitnah in die Lernfeldgruppen zurück. Auf diese Weise wurde die Arbeit der Kompetenzentwicklungsgruppe zum entscheidenden Unterstützungsprozess für die Lernfeldentwicklung. Er stellte sicher, dass, neben der Bearbeitung einzelner Lernfelder durch die Planungsteams und auf den Präsenztreffen, die lernfeldübergreifende Perspektive auf die Kompetenzentwicklung im Gesamtprozess wirksam verankert blieb.

3.2.2.1 Lernfeldspezifischer Prozess

Die Zuständigkeiten der Schulen für die einzelnen Lernfelder ergaben sich aus der bereits 2004 vorgenommenen Zuordnung (vgl. Abbildung 1). Ein Planungsteam aus je zwei bis drei Lehrenden der zuständigen Schule entwickelte gemeinsam mit den Mitarbeiterinnen des IBW die Planung für ein Lernfeld. Dabei legte das IBW in aller Regel einen ersten Planungsvorschlag vor, der vom Planungsteam überarbeitet wurde und dann die Basis eines gemeinsamen Treffens darstellte, auf dem die Planung detailliert diskutiert wurde. Auf diese Weise wurde eine Fassung des Lernfeldes erarbeitet, die auf dem kommenden Präsenztreffen für einen großen Teil des Kollegiums zur Diskussion gestellt werden konnte. Den Mitarbeiterinnen des IBW kam hierbei regelmäßig die Rolle zu, die Ideen, Vorstellungen und Ausarbeitungen der Kompetenzdimensionengruppe in diese Arbeit einzuspeisen, sie argumentativ zu vermitteln und ggf. Einwänder und Modifikationen der Lernfeldgruppe ins Kompetenzdimensionenteam zurückzuvermitteln.

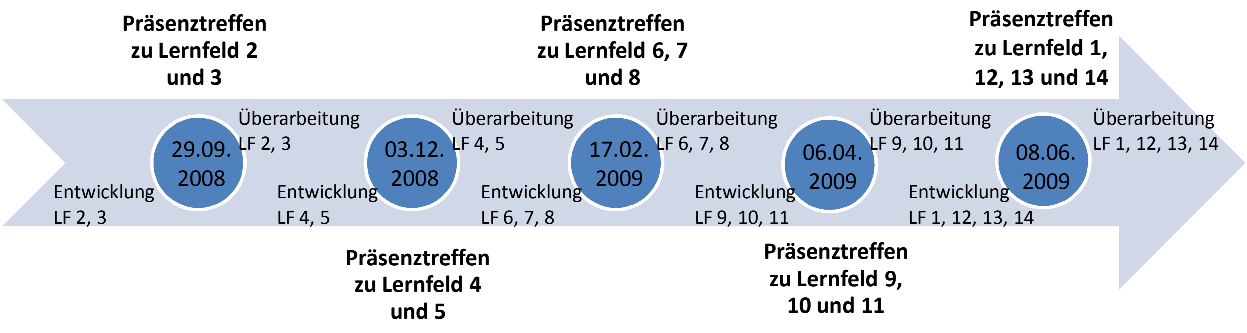

Abbildung 14: Lernfeldbezogener Entwicklungsprozess im Rahmen von EvaNet-EH

Abbildung 14 zeigt die lernfeldbezogenen Entwicklungsprozesse und Veranstaltungen. Mit Hilfe aller Beteiligten ist es gelungen, die lernfeldbezogenen Planungen für den gesamten Bildungsgang fertigzustellen. Der Pfeil deutet an, dass

der begonnene curriculare Entwicklungsprozess jedoch nicht als abgeschlossen betrachtet werden kann. Vielmehr müssen die Schulen über die Projektlaufzeit hinaus Verabredungen treffen die sicherstellen, dass der Rahmen für eine kontinuierliche Weiterentwicklung und Verbesserung des Curriculums geschaffen wird (vgl. Kapitel 4).

Während an der vorbereitenden Erarbeitung der Lernfelder und der Ausdifferenzierung der Kompetenzdimensionen nur relativ wenige Lehrende mitwirkten, war es das Ziel der Präsenztreffen, einen großen Teil der Kollegien an der curricularen Entwicklungsarbeit zu beteiligen und einen direkten und persönlichen Austausch im Rahmen der schulübergreifenden Kooperation zu ermöglichen. Dieses Vorgehen sollte die Akzeptanz, Verbindlichkeit und Legitimität des gemeinsamen Curriculums stärken, den Sachverstand und die Erfahrung der beteiligten Kolleginnen und Kollegen produktiv einbeziehen und schließlich auch eine Kultur des curricularen Diskurses entwickeln helfen, die über die Laufzeit des Projekts hinaus eine Verstetigung der Projektimpulse gewährleisten kann.

Auf den einzelnen Präsenztreffen wurden die Lernfelder jeweils vom zuständigen Planungsteam vorgestellt. Daran anschließend wurden die Lernfeldplanungen in Arbeitsgruppen diskutiert und überarbeitet. Um die schulübergreifende Kooperation auch längerfristig zu sichern, sollte auf den Präsenztreffen je Schule und Lernfeld ein Ansprechpartner benannt werden. Die Ansprechpartner für die Lernfelder haben über die Projektlaufzeit hinaus die Aufgabe, inhaltlich mit den Planungsteams zusammenzuarbeiten, die Erfahrungen der Kollegen beim Umgang mit den Planungen dahingehend zu reflektieren, ob sie Veränderung der Planungen nötig machen, Anpassungen der Planungen zu diskutieren und vorzunehmen sowie das eigene Kollegium über veränderte Planungen zu informieren.

Zum Teil gab es zum Beginn oder Ende der Präsenztreffen kurze Vorträge, die auf den Informationsbedarf der Teilnehmer reagierten. Insbesondere im Rahmen des ersten Präsenztreffens sahen wir die Notwendigkeit, die teilnehmenden Lehrer über die curriculare Strategie zu informieren, damit sie bei der Überarbeitung der Lernfeldplanungen die Ideen und Begründungen, die hinter den einzelnen Planungsbestandteilen stehen, berücksichtigen und einfließen lassen konnten.

Die Abbildungen 15-1 bis 15-3 zeigen beispielhaft die Tagesordnung, die Zielsetzungen und die Aufgabenstellungen eines Präsenztreffens.

Tagesordnung:

13:00 – 13:15	Begrüßung<br>Ablauf und Zielsetzungen
13:15 – 13:40	Informationen zur curricularen Arbeit im Rahmen von EvaNet-EH
13:40 – 14:00	Begründungszusammenhang LF 2 (H1)
14:00 – 14:20	Begründungszusammenhang LF 3 (H6)
14:20 – 14:30	Arbeitsauftrag
14:30 – 14:45	Kaffeepause
14:45 – 16:30	Bearbeitung von LF 2 und LF 3
16:30 – 17:30	Zusammenführung und -fassung der Arbeitsergebnisse, Verabredungen
17:30 – 17:50	Didaktische Jahresplanung
17:50 – 18:00	Verabschiedung

Abbildung 15-1: Beispiel für eine Tagesordnung

Zielsetzungen der Veranstaltung:

Die TeilnehmerInnen …

- sind über die Zielsetzung und den Nutzen der LF-KD-Matrix informiert
- kennen den aktuellen Planungsstand der Lernfelder 2 und 3
- haben den „Planungsstand LF 2+3“ in Kleingruppen diskutiert
  - und sich mehrheitlich auf (ab sofort) verbindliche Teile dieses Planungsstands festgelegt
  - und sich mehrheitlich auf Verbesserungsvorschläge für Teile dieses Planungsstands geeinigt.
- haben je eine/n Ansprechpartner/in aus jeder Schule für die Lernfeld-Teams bestimmt.
- haben ein Beispiel für eine didaktische Jahresplanung kennen gelernt

Abbildung 15-2: Beispiel für Zielsetzungen eines Präsenztreffens

Bitte bearbeiten Sie die folgenden drei Arbeitsaufträge:

1. Schritt: Curriculare Funktion (Bezug: Curriculare Analyse)

Die curriculare Funktion soll ein LF von anderen LF abgrenzen. Hier ist beschrieben, welchen Beitrag dieses Lernfeld zur Entwicklung der beruflichen Handlungskompetenz leistet.

Bitte überprüfen Sie, ob die Aussagen zur curricularen Funktion Ihnen eine erste verständliche **Orientierung über Ziele, thematische Schwerpunkte und die Einordnung des Lernfeldes** im Bildungsgang ermöglicht.

(Zeitrichtwert: 30 Min.)

2. Schritt: Prozesse und Kompetenzen (Bezug: Curriculare Analyse)

Die Abbildung des betrieblichen Prozesses vergegenwärtigt, wofür die Schüler in ihrer Ausbildung qualifiziert werden sollen. Der Arbeitsprozess ist grundlegend für die Beschreibung der Kompetenzziele, da diese nur unter Berücksichtigung ihrer Bedeutung für das Berufsleben der Schüler gerechtfertigt sein können.

Die Kompetenzziele in den Kompetenzdimensionen sind wichtiger Bestandteil des gemeinsamen Curriculums. Sie bestimmen die Schwerpunktsetzung der Lernfelder und sollen die Orientierung hinsichtlich der Ziele und Inhalte in den Lernfeldern erleichtern.

a) Bitte überprüfen Sie, ob die angeführten zentralen Prozesse und Schlüsselsituationen die **Anforderungen** beschreiben, **die ein Einzelhandelskaufmann bewältigen können soll**.

b) Bitte überprüfen Sie,

- ob **alle wichtigen** Kompetenzen angesprochen werden,
- ob **alle angesprochenen** Kompetenzen **wichtig** sind,
- ob die Kompetenzformulierungen hinsichtlich des **Inhalts und Niveaus** Ihren Vorstellungen und Erfahrungen entsprechen und
- ob die Kompetenzen **verständlich** formuliert sind.

(Zeitrichtwert: 45 Min.)

3. Schritt: Sequenzierung (Bezug: Strukturplanung)

Die Sequenzierung soll den „Gang durch das Lernfeld“ vor dem Hintergrund didaktischer Überlegungen abbilden.

Bitte prüfen Sie, ob die Einteilung und Abfolge der Sequenzen sowie die jeweilige Darstellung der curricularen Funktion für Sie **verständlich, praxistauglich und didaktisch angemessen** sind.

(Zeitrichtwert: 45 Min.)

Abbildung 15-3: Aufgabenstellungen für die Arbeitsgruppen

3.2.2.2 Lernfeldübergreifender Prozess

Wenn die Arbeit an den Kompetenzdimensionen im Einzelhandel auch etwa ein Jahr vor dem ersten Präsenztreffen begann, so waren bis dahin dennoch nicht alle Kompetenzdimensionen ausdifferenziert. Aus diesem Grund mussten auch die Mitglieder der Planungsteams noch nicht vorhandene Kompetenzbeschreibungen für die von ihnen zu bearbeitenden Lernfelder formulieren. Dies taten sie, im Gegensatz zur Kompetenzentwicklungsgruppe, in der Regel aus der Perspektive des jeweiligen Lernfeldes.

Das konsequente Einnehmen einer lernfeldübergreifenden Perspektive erfordert einen sehr intensiven und ins Detail reichenden Arbeits- und Diskussionsprozess, der zwischen systematischen und situativen Betrachtungen wechselte. Die von den Planungsteams beschriebenen Kompetenzen aus Lernfeldperspektive boten der Kompetenzentwicklungsgruppe Anknüpfungspunkte bei der Arbeit an der entsprechenden Kompetenzdimension und wurden bei Bedarf aus der lernfeldübergreifenden Perspektive überarbeitet.

Es fanden insgesamt zwölf Workshops zu den Kompetenzdimensionen im Einzelhandel statt, von denen der erste zum Zweck hatte, einen größeren Kollegenkreis in die Arbeit aus der lernfeldübergreifenden Perspektive einzuführen. Ergebnisse dieses ersten Workshops waren die Verständigung auf eine erste Grobstruktur von Kompetenzdimenionen sowie Anregungen zur Struktur der einzelnen Kompetenzdimensionen. Zudem rekrutierten sich aus diesem Kreis die sechs Kollegen, die während der verbleibenden Projektlaufzeit die Kompetenzentwicklungsgruppe bildeten und an einer Ausdifferenzierung der Kompetenzdimensionen arbeiteten.

Folgende Übersicht enthält die Termine und Gegenstände der zwölf Workshops.

Datum	Gegenstand des Workshops
**20.09.2007**	- Einführung in die Arbeit mit lernfeldübergreifenden Kompetenzdimensionen<br>- erste Ansätze zur Ausdifferenzierung der einzelnen Kompetenzdimensionen in Arbeitsgruppen
**30.10.2007**	- Ausdifferenzierung der Kompetenzdimension Identität, Berufsrolle, Berufsausbildung, Berufsperspektiven<br>- erste Ansätze zur Ausdifferenzierung der Kompetenzdimension prozessübergreifende Lern- und Arbeitstechniken
**27.11.2007**	- Ausdifferenzierung der Kompetenzdimension Identität, Berufsrolle, Berufsausbildung, Berufsperspektiven – Subdimension Berufsethos<br>- erste Überlegungen zur Kompetenzdimension Rechtliche Normierung
**30.01.2008**	- Ausdifferenzierung der Kompetenzdimension Rechtliche Normierung
**08.04.2008**	- Ausdifferenzierung der Kompetenzdimension Betriebswirtschaftliche Prozessdimension
**16.06.2008**	- Ausdifferenzierung der Kompetenzdimension Betriebswirtschaftliche Prozessdimension – Subdimensionen Absatzmarkt- und Kundenbeziehungen sowie Logistik
**09.09.2008**	- Ausdifferenzierung der Kompetenzdimension Wertschöpfung und Controlling<br>- Ausdifferenzierung der Kompetenzdimension Wertschöpfung und Controlling – Subdimension Liquiditätssicherung
**12.11.2008**	- Ausdifferenzierung der Kompetenzdimension Wertschöpfung und Controlling

	– Subdimension Kosten- und Leistungsrechnung sowie Finanzbuchhaltung<br>- Weiterarbeit an der Kompetenzdimension prozessübergreifende Lern- und Arbeitstechniken
**19.01.2009**	- Anpassung der Kompetenzdimensionen<br>- Überprüfung der Kompetenzdimension Wertschöpfung und Controlling – Subdimension Kosten- und Leistungsrechnung sowie Finanzbuchhaltung<br>- Überprüfung der Kompetenzdimension prozessübergreifende Lern- und Arbeitstechniken
**04.03.2009**	- Ausdifferenzierung der Kompetenzdimension Betriebswirtschaftliche Prozessdimension – Subdimensionen Informationswirtschaft sowie Personalwirtschaft
**15.04.2009**	- Ausdifferenzierung der Kompetenzdimension Systemverständnis
**13.05.2009**	- Ausdifferenzierung der Kompetenzdimension Lern- und Arbeitstechniken sowie Kommunikation und Kooperation

Tabelle 10: Termine und Gegenstände des Workshops Kompetenzdimensionen

Die einzelnen Workshops begannen in der Regel mit einem Beitrag seitens der MitarbeiterInnen des IBW. Es wurden Arbeitsergebnisse der vergangenen Workshops zusammengefasst und/oder erste Überlegungen zur der jeweils zu bearbeitenden Kompetenzdimensionen vorgestellt. Darauf folgte der Arbeitsprozess an einer oder mehreren Kompetenzdimensionen, je nach Bedarf und Fall arbeitsteilig oder in der Gesamtgruppe.

Idealtypisch haben sich die Arbeitsprozesse an vier Schritten orientiert, die der folgenden Abbildung zu entnehmen sind.

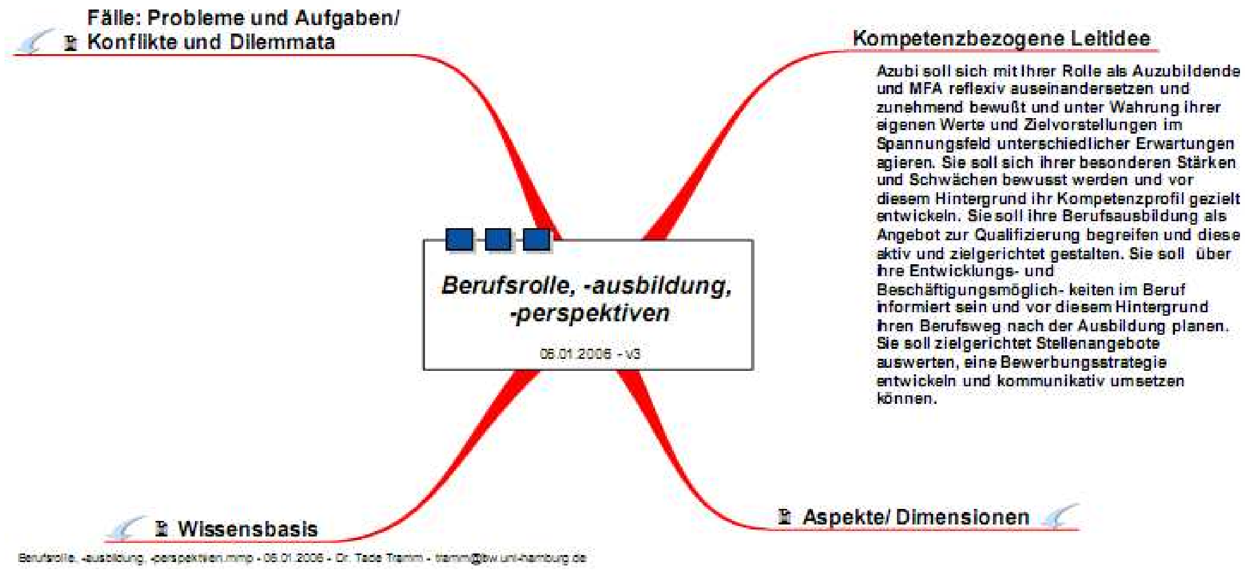

Abbildung 16: Idealtypische Arbeitsschritte im Workshop Kompetenzdimensionen (Tramm 2006)

Zu Beginn eines jeden Workshops stand zumeist der Versuch, sich ein umfassendes Bild von einer Kompetenzdimension zu machen. Dabei wurde die Komplexität bewusst nicht reduziert, um den Bereich zunächst zu „öffnen“. Erst dann wurden diese Überlegungen um Fragen der Relevanz für die Schüler erweitert, um als Grundlage für die Formulierung einer Leitidee der Kompetenzdimension zu dienen. Weiter ging es darum, die Aspekte der jeweiligen Kompetenzdimension herauszuarbeiten, die sich unter Umständen als Subdimensionen eignen könnten (je

nachdem, welchen Umfang ein solcher Aspekt im Curriculum einnehmen sollte). Bei der Bestimmung von Aspekten bzw. Subdimensionen einer Kompetenzdimension kann nicht zwangsläufig von fachsystematischen Strukturen ausgegangen werden. Diese stellen nur eine Möglichkeit dar, einen Inhaltsbereich zu strukturieren. Sie wurden um situative (berufliche, gesellschaftliche und private) und persönlichkeitsbezogene Überlegungen ergänzt, wobei sich die Suche nach relevanten Fällen, nach Schlüsselsituation, Dilemmata und Konflikten, die der jeweiligen Kompetenzdimension zugeordnet werden konnten, als hilfreich erwies. Die Wissensbasis wurde meist im Rahmen der Überlegungen zur lernfeldübergreifenden Entwicklung ergänzt, die auf die beschriebene, von den Lernfeldern eher unabhängige, Strukturierungsarbeit folgte. Denn nachdem wir uns ein zunächst lernfeldunabhängiges Bild von einer Kompetenzdimension gemacht hatten, folgte die Ausarbeitung einer lernfeldübergreifenden Entwicklungslogik. Es wurden die Lernfelder markiert, die sich offensichtlich zur Thematisierung der Inhalte der jeweiligen Kompetenzdimension eigneten und Notizen zum jeweiligen Kompetenzerwerb gemacht. Entsprechend mussten Überlegungen zur Sachlogik und zum Anspruchsniveau einfließen. Als Grundlage der Kompetenzformulierungen für die einzelnen Lernfelder dienten die Informationen des Rahmenlehrplanes, der Ausbildungsordnung und die im vorangegangenen Entwicklungsprozess von 2004 bis 2007 bereits beschriebenen Kompetenzen.

Die abgebildeten vier Arbeitsschritte stellen lediglich eine analytische Einteilung dar. Im konkreten Arbeitsprozess sind sie weder in bestimmter Reihenfolge noch strikt voneinander trennbar oder frei von Wiederholungen verlaufen. Es handelt sich vielmehr um einen heuristisch strukturierten Verständigungsprozess, bei dem unterschiedliche Argumentationen i. d. Regel einem der Arbeitsschritte zugeordnet werden können.

Die Mitarbeiterinnen des IBW übernahmen die Verschriftlichung der Ergebnisse in Form der Formulierung einer Leitidee der Kompetenzdimension und der Beiträge, die die einzelnen Lernfelder zum Kompetenzerwerb in der Kompetenzdimension leisten sollten. Diese Ergebnisse der Kompetenzentwicklungsgruppe wurden zur Überprüfung an die Teilnehmer zurückgespiegelt. Die Nutzung eines Weblogs (siehe http://evanettemichaela.wordpress.com/) für eine kontinuierliche Zusammenarbeit zwischen den Workshopterminen erwies sich zum Teil als nützlich und ermöglichte eine transparente Entwicklung der lernfeldübergreifenden Kompetenzdimensionen.

Die Kompetenzformulierungen als Ergebnis dieses Arbeitsprozesses wurden in die Lernfeldplanungen übernommen und im Rahmen der Arbeit der Planungsteams und auf den Präsenztreffen ein weiteres Mal überprüft.

3.3 Produkte

Im Folgenden werden die Produkte der kooperativen Curriculumentwicklung beschrieben und exemplarisch dargestellt. Die Lernfeldplanungen einerseits (Kapitel 3.3.1) und die Kompetenzbeschreibungen andererseits (Kapitel 3.3.2) fließen in der Lernfeld-Kompetenz-Matrix (Kapitel 3.3.3) zusammen.

3.3.1 Lernfeld-Planungen

Die Planungsstruktur (Abbildung 17-1 bis 17-3) diente im Wesentlichen dazu, der Arbeit an den Lernfeldern eine einheitliche Struktur zu geben. Sie ermöglichte, auf Basis eines gemeinsamen Vokabulars, über curriculare Fragen zu sprechen. In der curricularen Analyse unter *Kompetenzdimensionen*, *Kompetenzen* und *Wissensbasis*

fließt die lernfeldspezifische Entwicklungsarbeit mit der lernfeldübergreifenden Ausdifferenzierung der Kompetenzdimensionen zusammen.

**Lernfeld**	Name des Lernfeldes		**Ausbildungsjahr:**<br>**Zeitrichtwert:**
**Verantwortlich:**<br>**(Planungsteam)**		**Ansprechpartner:**	**Version:**<br>**Datum:**
**I Curriculare Analyse**			
**Zielformulierung nach KMK-RLP:** *Ziele und Inhalte aus RLP*			
**Curriculare Funktion:** *Aufgabe des Lernfeldes im Curriculum*			
**Zentrale Prozesse, relevante Prozessvarianten, Prozessschritte und Tätigkeiten der betrieblichen Praxis:**<br>*(Betrachtung der dem Lernfeld zugrundeliegenden Arbeits-/Geschäftsprozesse, keine didaktische Aufbereitung)*<br>**Kernprozess:**<br>**Prozessvarianten:**<br>**Konflikte, Dilemmata, Schlüsselsituationen, Störungen (Beispiele:)**<br><br>*sequenzielle Beschreibung des Kernprozesses*<br><br>*Auflistung von Prozessvarianten, Konflikten und Dilemmata, Schlüsselsituationen und Störungen, die besonders praxisrelevant oder von hoher systematischer Bedeutsamkeit sind*			

Kompetenzdimensionen in der Übersicht (die relevanten Kompetenzdimensionen sind angekreuzt)

KD	Beruflichkeit					Lern- und Arbeitstechniken					Rechtliche Normierung			Betriebswirtschaftliche Problemebenen			Wertschöpfung und Controlling		
	BE					LAT					NORM			BWP			WUC		
	Identität und Berufsrolle	Berufsethos	Gesundheitsförderung	Berufsbildung und -perspektiven	Kommunikation & Kooperation KOKO	Kaufmännisches Rechnen	Arbeitshandeln organisieren, Probleme lösen	Informationen gewinnen, verarbeiten, vermitteln	Lernen planen und optimieren	Das System Unternehmung SYST	Vertrags- und Gesellschaftsrecht	Arbeits- und Schuldrecht	Steuerrecht	Beschaffung und Logistik	Absatzmarkt- und Kundenbeziehungen	Informationswirtschaft	Liquiditätssicherung	Kosten- und Leistungsverständnis	Finanzbuchhaltung
LF1																			
LF2																			
LF3																			
LF4																			
LF5																			
LF6																			
LF7																			
LF8																			
LF9																			
LF10																			
LF11																			
LF12																			
LF13																			
LF14																			

**Kompetenzdimension:** *Kompetenzen und Wissensbasis für alle im Lernfeld relevanten Kompetenzdimensionen*	
**Kompetenzen:** *Zu erwerbende Fähigkeiten, Erkenntnisse und Einstellungen und Hinweis auf das angestrebte Kompetenzniveau*	**Wissensbasis:** *Faktenwissen, Konzeptuelles Wissen und operatives Wissen, Struktur und Konzepte*

Abbildung 17-1: Curriculare Analyse

**Lernfeld X**	Name des Lernfeldes		**Ausbildungsjahr:**<br>**Zeitrichtwert:**
	**II Strukturplanung**		
**Teilsequenzen**<br>**(Aufgabe/Problemstellung)**		**Curriculare Funktion**<br>**Erkenntnisschritte/ Kompetenzschwerpunkte**	**t**
**1.**	*Ausgangsproblematik und Aufgabenstellung für die Lernenden*	*was soll erreicht werden, was ist der intentionale Schwerpunkt, wo liegt der inhaltliche Akzent, welche Kompetenzdimensionen werden angesprochen?*	
**2.**	*z. B. Variation der Ausgangsproblematik, neue oder ergänzende Aufgabenstellung*		
**3.**	*z. B. Variation oder Störung*		
**4.**			

Abbildung 17-2: Strukturplanung

**Lernfeld X**	Name des Lernfeldes		**Ausbildungsjahr:**<br>**Zeitrichtwert:**
	**III Makroplanung**		
	**Teilsequenz 1:**		
**Zielformulierung/Kompetenzen**		**Wissensbasis**	
		•	
**Intendierter Lernschritt**<br>*Was wird gelernt?*	**Lernhandlungen**<br>*Was tut der Lernende?*	**Lernaufgaben/-situation**<br>*Impuls des Lehrenden*	**Erfolgsindikatoren**<br>*Wie zeigt sich der Lerneffekt?*
	**Teilsequenz 2 bis ....:**		
**Zielformulierung/Kompetenzen**		**Wissensbasis**	
		•	
**Intendierter Lernschritt**<br>*Was wird gelernt?*	**Lernhandlungen**<br>*Was tut der Lernende?*	**Lernaufgaben/-situation**<br>*Impuls des Lehrenden*	**Erfolgsindikatoren**<br>*Wie zeigt sich der Lerneffekt?*

Abbildung 17-3: Makroplanung

Teil I des Planungsformates, die **curriculare Analyse** (Abbildung 17-1), stellt eine Interpretation des Lernfeldes dar. Auf Basis der Informationen des Rahmenlehrplanes wird die **curriculare Funktion** des Lernfeldes beschrieben. Sie enthält Informationen zu den inhaltlichen Schwerpunkten des Lernfeldes und zur Einordnung des Lernfeldes in das Gesamtcurriculum. Im Gegensatz zu den KMK-Formulierungen erhebt sie nicht den Anspruch, Ziele zu formulieren. Stattdessen soll sie in einigen Sätzen einen ganzheitlichen Eindruck davon vermitteln, worum es in dem Lernfeld geht und welche Zusammenhänge mit anderen Lernfeldern bestehen. Um der Orientierung an Arbeits- und Geschäftsprozessen Rechnung zu tragen, folgt auf die curriculare Funktion die **Rekonstruktion des betrieblichen Prozesses**, der dem Lernfeld zugrunde liegt, sowie seiner Varianten und seiner spezifischen Merkmale in Form von Konflikten und Schlüsselsituationen. Entscheidend ist, dass es sich hier noch nicht um didaktische Überlegungen zum Inhalt und zur Gestaltung des Unterrichts handelt, sondern um den Versuch, die situativen Referenzen des jeweiligen Lernfeldes möglichst umfassend in den blick zu nehmen, um auf dieser Grundlage reflektierte und begründete Reduktions- und Akzentuierungsentscheidungen treffen zu können. Dieser didaktische Entscheidungsaspekt kommt im nächsten Schritt der curricularen Analyse zur

Geltung, in dem es um die Frage geht, welche Kompetenzdimensionen im Lernfeld eine Rolle spielen und welche **Kompetenzen** die Schüler erwerben sollen.[8]

In der **Strukturplanung** (Abbildung 17-2) wird die Sequenzierung des Lernfeldes vorgenommen. Sie entspricht der Entscheidung über die Abfolge der Lernsituationen, also der Frage, über welche Lernarrangements den Schülern der Arbeits- bzw. Geschäftsprozess des Lernfeldes zugänglich gemacht wird. Dabei wird jede Sequenz möglichst mit der konkreten Problem- oder Aufgabenstellung benannt und wiederum in einer curricularen Funktion beschrieben. Die curriculare Funktion einer Teilsequenz fasst diese zusammen, trifft erste Aussagen zu den Erkenntnisschritten und benennt die relevanten Kompetenzdimensionen.

Im Rahmen der **Makroplanung**[9] (Abbildung 17-3) werden zunächst jeder Teilsequenz die zu erwerbenden Kompetenzen inkl. der Wissensbasis zugeordnet. Darauf folgen konkrete Entscheidungen zur Unterrichtsgestaltung. Es werden Lernschritte, Lernhandlungen, Lehrhandlungen sowie didaktische Erfolgsindikatoren benannt.

Die Aufgabe der Planungsteams war es, ihre Arbeit entlang der einzelnen Kategorien dieses curricularen Planungsformates zu organisieren und zu dokumentieren. Dem Format kam damit die doppelte Aufgabe zu, einerseits die Arbeit der jeweiligen Lernfeldgruppe entlang einer curricularen Entwicklungslogik zu strukturieren und andererseits durch die redundante Grundstruktur allen Beteiligten die Rezeption und die strukturierte Reflexion der Arbeit anderer Gruppen zu erleichtern.

3.3.2 Kompetenzdimensionen

Das Arbeitsergebnis der Kompetenzentwicklungsgruppe besteht aus einem System von Kompetenzdimensionen, das die Zieldimension des schulischen Teils der Berufsausbildung der Einzelhandelskaufleute sowie Verkäufer und Verkäuferinnen strukturiert abbildet und beschreibt.

Die Kompetenzdimensionen wurden im Projektverlauf mehrmals verändert, um den Ansprüchen der Ordnungsmittel, den betrieblichen Prozessen, der Sachlogik der Inhalte und den Erfahrungen der Lehrenden Rechnung zu tragen. Das Endergebnis sind sieben Kompetenzdimensionen, die inklusive ihrer Subdimensionen in Abbildung L dargestellt sind.

---

[8] Die Bestimmung der Kompetenzdimensionen und die Beschreibung von Kompetenzen sind im Idealfall bereits vor der Lernfeldentwicklung erfolgt (, weil dies zuvor aus einer lernfeldübergreifenden Entwicklungsperspektive unter Berücksichtigung der einzelnen Lernfelder geschehen ist, vgl. Kapitel 3.2.2.2 und 3.3.2) und werden im Rahmen der curricularen Analyse aus der Lernfeldperspektive überprüft. Im Fall des Projekts EvaNet-EH waren bei Beginn der Arbeit an den Lernfeldplanungen noch nicht alle Kompetenzdimensionen ausdifferenziert. Dies hatte zur Folge, dass fehlende Kompetenzbeschreibungen von den Planungsteams ergänzt wurden. Diese wurden später aus einer lernfeldübergreifenden Perspektive von der Kompetenzentwicklungsgruppe überarbeitet.

[9] Die Makroplanung wurde im Rahmen des Projektes EvaNet-EH weitestgehend nicht bearbeitet, da sie Entscheidungen beinhaltet, die aufgrund unterschiedlicher Rahmenbedingungen und Schülerklientel nur schulspezifisch getroffen werden können.

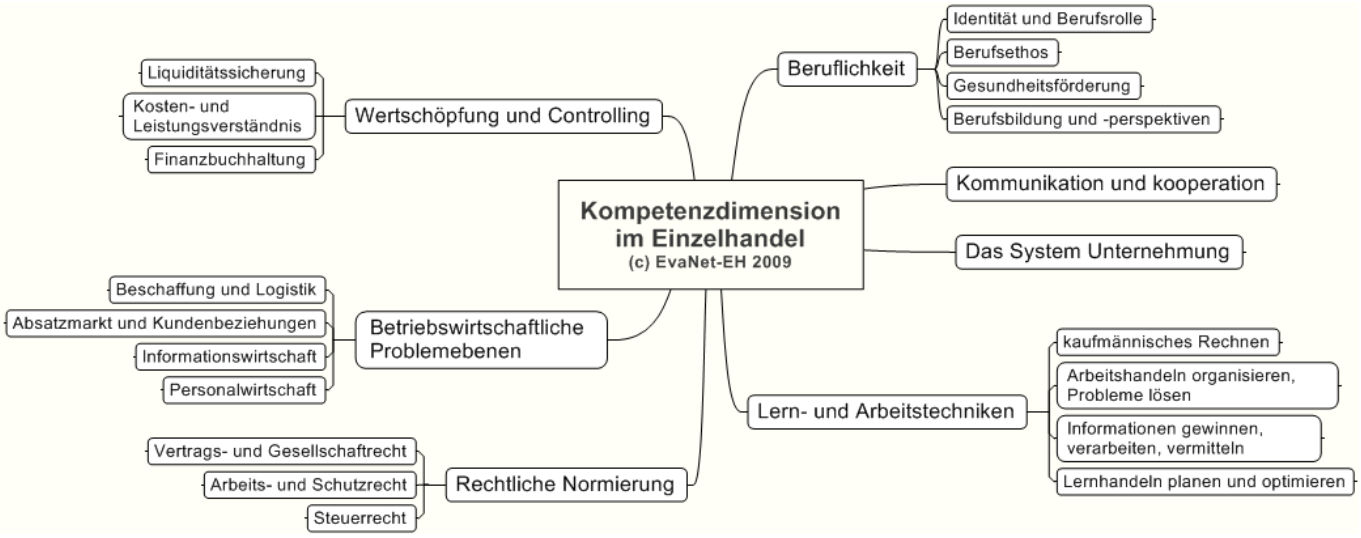

Abbildung 18: Kompetenzdimensionen im Einzelhandel

Insbesondere war es die Aufgabe der Kompetenzentwicklungsgruppe, zu entscheiden, welche Lernfelder einen Beitrag zur Kompetenzentwicklung in einer bestimmten Kompetenzdimension leisten und zu beschreiben, worin dieser Beitrag besteht. Darüber hinaus sollte für jede Kompetenzdimension eine Leitidee formuliert werden, die das Kompetenzentwicklungsziel der Kompetenzdimension, bezogen auf die gesamte Ausbildung, beschreibt. Exemplarisch ist dies hier für die Subdimension Berufsethos dargestellt.

<table>
<tr><td colspan="3">Leitidee der Kompetenzdimension Beruflichkeit
Im Mittelpunkt dieser Kompetenzdimension steht das Verständnis und die Motivation für die aktive, eigenverantwortliche, zielgerichtete und erfolgreiche Gestaltung des eigenen Berufslebens. Die Schülerinnen und Schüler entwickeln zum einen eine positive Einstellung zu ihrem Beruf, zu den Anforderungen, die an sie gestellt werden und lernen mit typischen ethischen/moralischen Problemen umzugehen. Zum anderen werden die Schüler darin gefördert, ihre eigenen Stärken und Schwächen kennenzulernen, diese aus- bzw. abzubauen, sich ein individuelles Kompetenzprofil anzulegen und dementsprechende berufliche Ziele zu setzen.</td></tr>
<tr><td colspan="3">Leitidee der Subdimension Berufsethos
Den Schülern sind moralische Konflikte zugänglich. Sie können Konflikte erkennen, differierende Interessen wahrnehmen, diese abwägen und verantwortlich Position beziehen. Zur Beurteilung moralischer Konflikte können sie eigene Interessen, Interessen des direkten Gegenübers, Interessen aller beteiligten Personen, Gesetze, die Legitimität von Gesetzen und universelle moralische Prinzipien (i.S. Kohlbergs) berücksichtigen. Je mehr der genannten Aspekte berücksichtigt werden, desto komplexer ist die Argumentation, auch wenn für die Positionsfindung schließlich einige Aspekte bewusst ausgeblendet werden (soll heißen, der Schüler ist sich zwar bewusst, dass etwas z. B. nicht den höchsten moralischen Prinzipien gerecht wird, bezieht aber zu Gunsten der Interessen der Beteiligten Stellung). Das Ziel in dieser Kompetenzdimension ist nicht die Vermittlung moralischer Prinzipien, sondern die Förderung moralischer Urteilsbildung in zunehmender Tiefe.</td></tr>
<tr><td>LF</td><td>Kompetenzformulierung</td><td>Wissensbasis</td></tr>
<tr><td>1</td><td></td><td></td></tr>
<tr><td>2</td><td>Die Schüler kennen potenzielle Konflikte zwischen Kundenwünschen und Unternehmensinteressen, die im Verkaufsgespräch auftreten können. Sie sind in der Lage Strategien zu entwickeln, mögliche moralische Konflikte auszuhalten und im Verkaufsgespräch zu berücksichtigen. Sie können ihr Verhalten zwischen den Zielen der Kundenorientierung und der Gewinnmaximierung austarieren. Die Schüler wollen ihren Informationsvorsprung gegenüber dem Kunden einsetzen, um einen auf seine Bedürfnisse</td><td>• Bedeutung von Werten und Normen beim Verkaufen
• Strategien der intraindividuellen Konfliktlösung</td></tr>
</table>

	abgestimmten Kaufabschluss herbeizuführen und nicht, um die Unwissenheit des Kunden einseitig zu Gunsten der Umsatzsteigerung auszunutzen.	
3	Die Schüler erkennen, dass ein gutes und offenes Verhältnis zwischen den Kollegen wichtig ist, um erfolgreich im Team zusammenzuarbeiten. Sie verhalten sich solidarisch und kollegial, können aber auch Probleme in offenen und respektvollen Gesprächen thematisieren.	• Teamarbeit und kollegiales Verhalten<br>• Konfliktlösung
4	Die Schüler identifizieren in Bezug auf die Warenpräsentation unternehmerische Strategien, die unter Umständen in Konflikt mit den Kundenwünschen stehen. Sie können diese Unternehmensstrategien beurteilen.	• Impulsware (z. B. Süßigkeiten in Kinderaugenhöhe)<br>• Regeln zum Verkauf von Schaufenster- oder Dekorationsware
5	Die Schüler kennen den Konflikt zwischen umsatzsteigernden Werbemethoden und der moralischen Vertretbarkeit gegenüber Kunden. Sie verfügen über eine eigene Werthaltung in Hinblick auf die soziale Verantwortung gegenüber den Kunden im Verhältnis zu den Unternehmenszielsetzungen.	• Grenzen der Werbung in Hinblick auf soziale Verantwortung, moralische Vertretbarkeit<br>• Verhaltensregeln der ZAW
6		
7		
8		
9	Die Schüler verstehen Preis-Leistungs-Verhältnisse und können hieraus ableiten, ob Preisgestaltungen den Informationsmangel des Verbrauchers oder die schwächere Position anderer Marktteilnehmer ausnutzen.	• Verteilung der Marktmächte im Einzelhandel<br>• Preiskampf mit Konkurrenten<br>• Auswirkungen von Verdrängungswettbewerb
10	Die Schüler erkennen, dass insbesondere schwierige Verkaufssituationen ein großes Konfliktpotenzial wegen der unterschiedlichen Interessen von Kunden und Unternehmen beinhalten. Sie wägen Kunden- und Unternehmensinteressen gegeneinander ab, vergleichen und beurteilen unterschiedliche Strategien des Umgangs mit besonderen Situationen und können für konkrete Situationen konfliktlösende Strategien entwickeln, die ihren eigenen Berufs- und Wertvorstellungen entsprechen.	• Konfliktlösungsstrategien<br>• Typische Interessenskonflikte<br>• Bedeutung von Werten und Normen beim Verkaufen
11		
12		
13		
14		

Tabelle 11: Kompetenzentwicklung in der Subdimension Berufsethos

3.3.3 Lernfeld-Kompetenz-Matrix

Zusammengefügt werden die Ergebnisse beider Arbeitsstränge in der Lernfeld-Kompetenz-Matrix, die nach bereits beschriebener Systematik aufgebaut ist. Im Modell werden die horizontal verlaufenden Lernfelder durch die vertikal verlaufenden Kompetenzdimensionen ergänzt, um so die Orientierungsleistung des Curriculums zu

erhöhen sowie die Betrachtung sachlicher und persönlichkeitsbezogener Schülerentwicklungen über die Lernfelder hinweg zu erleichtern.

Der Nutzen der Lernfeld-Kompetenz-Matrix ist wesentlich durch ihre mediale Darstellung (siehe www.ibw.uni-hamburg.de/evaneteh) begründet. Die Verlinkung der Kompetenzdimensionen und der Lernfelder ermöglicht einen schnellen und komfortablen Wechsel der beiden angesprochenen Perspektiven.

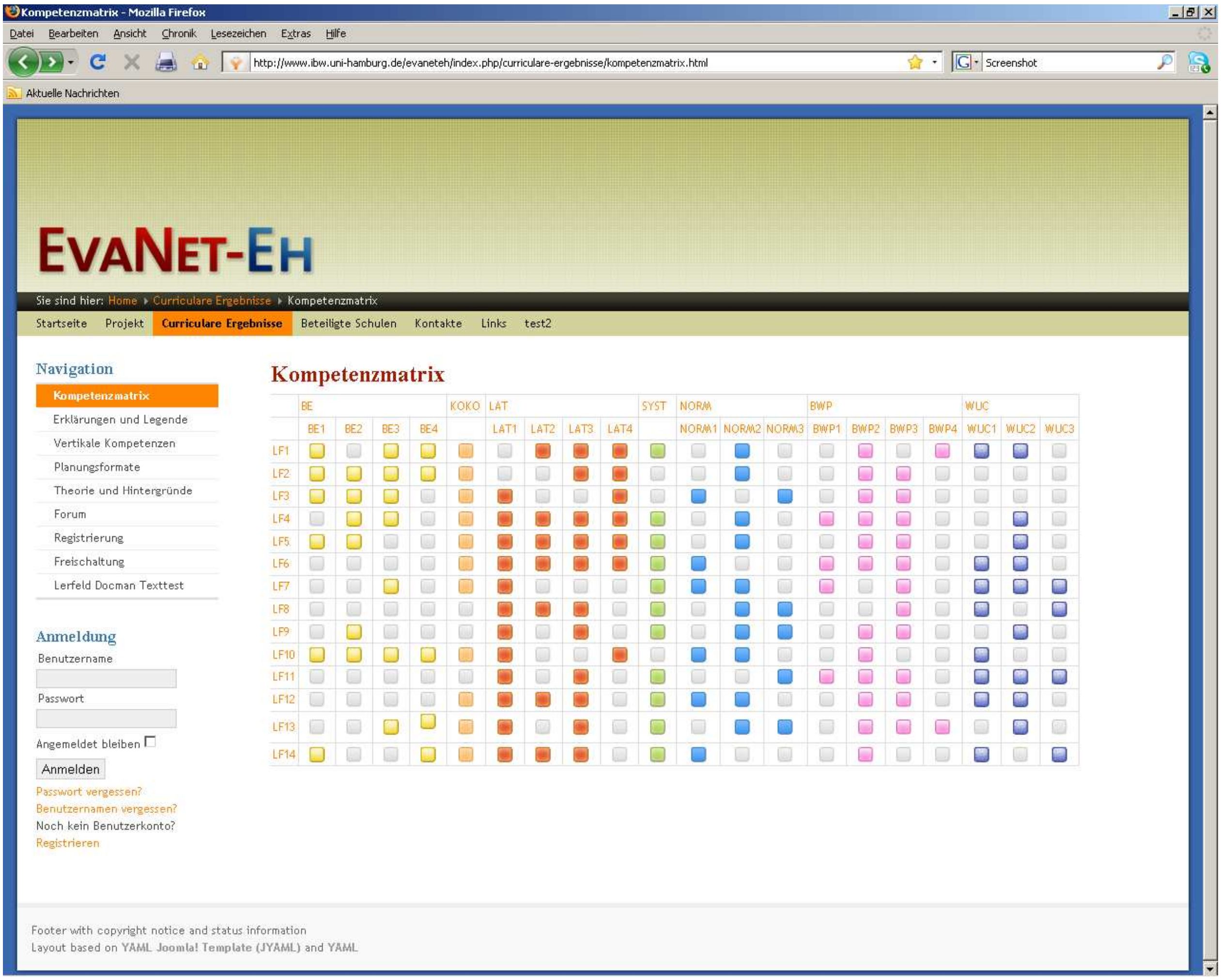

Abbildung 19: Die Lernfeld-Kompetenzmatrix auf www.ibw.uni-hamburg.de/evaneteh

Will man sich über ein Lernfeld informieren, erscheinen die wichtigsten Bestandteile der Planung sowie die gesamte Lernfeldplanung zum Download (Abbildung 20). Über den Matrix-Ausschnitt unter der Überschrift „Lernfeld 2 im Überblick“ kann man direkt weiter zu den Kompetenzdimensionen navigieren (in Abbildung 21 beispielhaft für BE1 geschehen) und von dort wieder zurück zu den Lernfeldern.

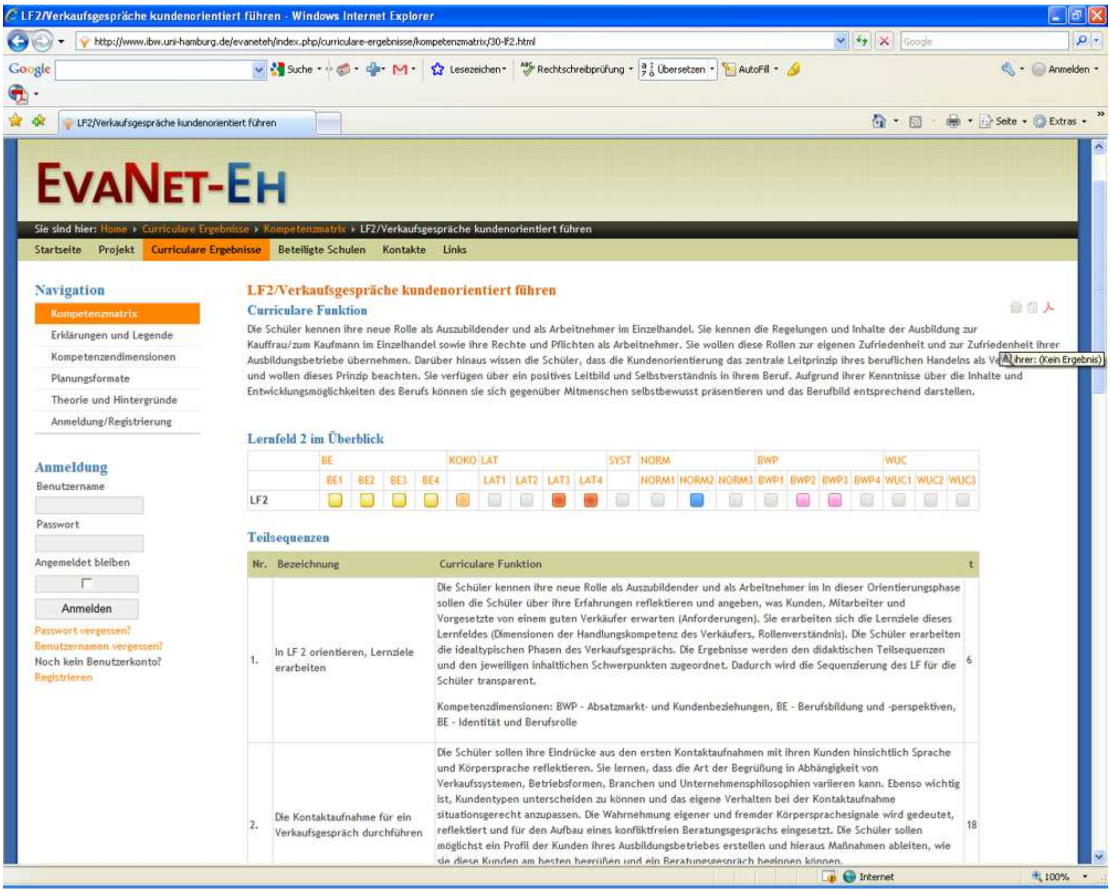

Abbildung 20: Lernfeld 2 auf www.ibw.uni-hamburg.de/evaneteh

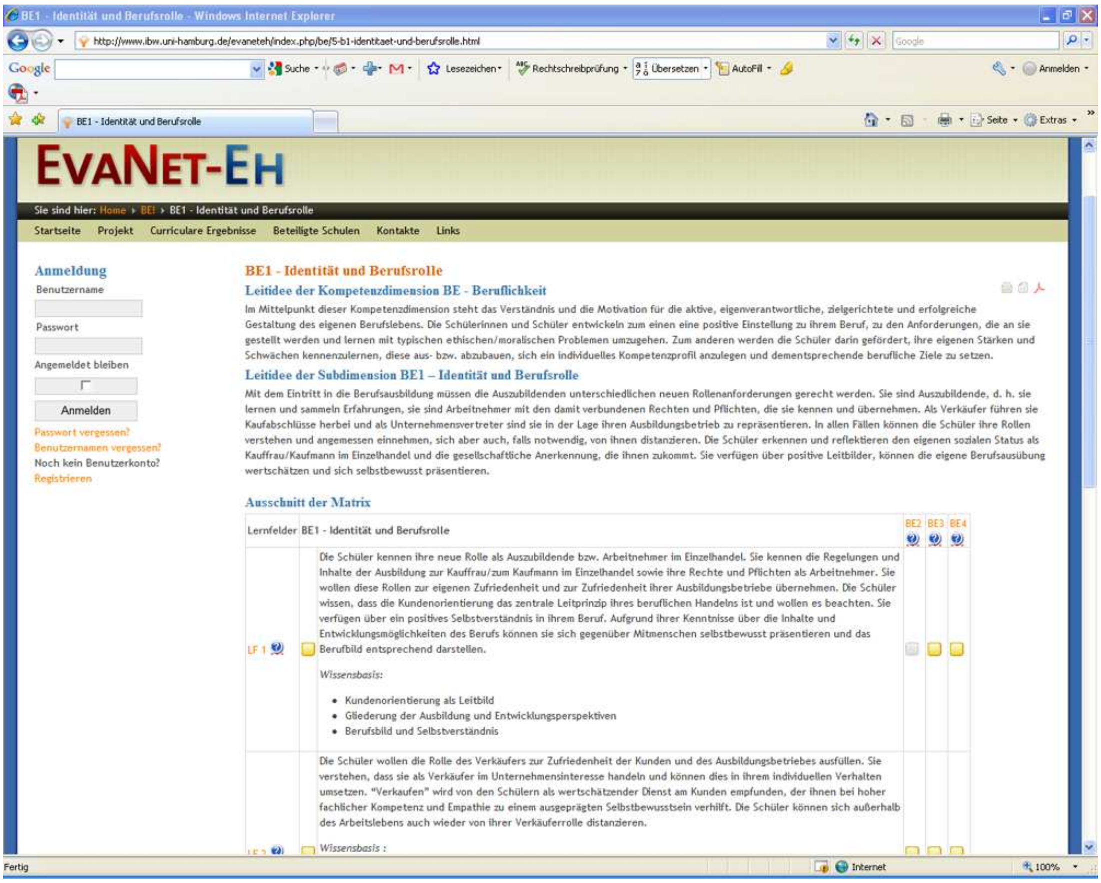

Abbildung 21: Kompetenzdimension Identität und Berufsrolle auf www.ibw.uni-hamburg.de/evaneteh

3.4 Gesamtreflexion

Das Projekt EvaNet-EH profitierte in curricularer Hinsicht entscheidend von den Vorarbeiten der Schulen und Lehrenden von 2004 bis 2007. Nur aufgrund der bereits etablierten Strukturen der Zusammenarbeit, der Bereitschaft der Lehrenden, sich mit curricularen Fragen auseinanderzusetzen, sowie der bereits vorhandenen Produkte konnte im Rahmen des Projektes, das als Evaluationsprojekt gestartet ist, eine so umfangreiche Weiterentwicklung des schulübergreifenden Curriculums stattfinden. Diese Weiterentwicklung bewerten wir als ausgesprochen positiv und anschlussfähig für weitere Innovationen.

Mit der entstandenen Lernfeld-Kompetenz-Matrix sind unterschiedliche Erfolge und Chancen verknüpft, von denen die aus unserer Sicht wesentlichsten an dieser Stelle abschließend benannt werden sollen:

- Für die vier beruflichen Schulen Hamburgs, die Kaufleute im Einzelhandel und VerkäuferInnen ausbilden, gibt es ein gemeinsames Curriculum, das geeignet ist sicherzustellen, dass sich die Lehrenden des Bildungsganges an den gleichen Grundlagen orientieren und der Unterricht nicht unangemessen voneinander abweicht.
- Im Rahmen von EvaNet-EH wurde eine Form der curricularen Arbeit gepflegt, die geeignet ist, über die Projektlaufzeit fortgeführt zu werden. Die angelegten Strukturen können optimiert und weiterentwickelt sowie an die Möglichkeiten und Bedürfnisse von Schulen und Lehrenden angepasst werden.
- Die Lernfeld-Kompetenz-Matrix wurde von nahezu allen im Einzelhandel unterrichtenden Kollegen erarbeitet, so dass gute Voraussetzungen für eine breite Akzeptanz des Curriculums geschaffen sind.
- Die vorstrukturierte Arbeit an der Lernfeld-Kompetenz-Matrix intendierte auch den Aufbau eines gemeinsamen Verständnisses von curricularer Entwicklung sowie einer gemeinsamen Sprache. Diese Errungenschaft ist besonders nützlich, um die Vision einer kontinuierlichen kooperativen Weiterentwicklung des Curriculums zu verwirklichen.
- Die stets geforderte Verknüpfung von Situations-, Sach- und Persönlichkeitsbezug ist auf curricularer Ebene gelungen. Ein Curriculum, dem diese Verbindung bereits innewohnt, kann Lehrende bei der Berücksichtigung der drei Aspekte im Unterricht hilfreich sein.
- Einzelnen Lehrenden steht eine umfangreiche Sammlung unterschiedlicher Materialien auf ganz unterschiedlichen Abstraktionsstufen zur Verfügung, die eine Einarbeitung in ein bestimmtes Lernfeld erleichtern kann.

Zugleich lassen sich mit Bezug auf diese Matrix (praktische) Entwicklungs- und (theoretische) Forschungsbedarfe identifizieren, die den aktuellen Nutzen dieses Instruments nicht in Frage stellen, aber Perspektiven einer weiteren rationalen Absicherung curricularer Entscheidungen deutlich machen:

- Die Kompetenzdimensionen im Hinblick auf den aktuellen Forschungsstand der BWP sowie relevanter Nachbardisziplinen elaborieren und weiterentwickeln
- Die Entwicklungsperspektive der unterschiedlichen Kompetenzdimensionen mit Blick auf Modelle und Befunde einschlägiger Wissenschaften weiter aufklären

- Die Referenzprozesse und die darauf bezogenen Kompetenzformulierungen über Arbeitsprozess- und Qualifikationsanalysen sowie über die Einbeziehung betrieblicher Experten validieren bzw. revidieren
- Bezogen auf die Kompetenzdimensionen Ansätze einer graduellen Erfassung entwickeln (Kompetenzniveaus)
- Bezogen auf unterschiedliche Kompetenzniveaus Instrumente zur Selbsteinschätzung durch Schüler und zur Fremdeinschätzung entwickeln
- Modelle der kompetenzdimensionsbezogenen leistungsmessung und –bewertung entwickeln und erproben.

4 Implementation der curricularen Produkte und Verstetigung der Arbeitsprozesse

Bei allen Projekten, die Innovationen zum Gegenstand haben, muss gefragt, werden, wie diese nach Projektende in der Praxis etabliert, in die täglichen Arbeitsprozesse integriert und kontinuierlich evaluiert und weiterentwickelt werden können. Dieses Etablieren, Integrieren, Evaluieren und Weiterentwickeln bezeichnen wir als *Implementation* sowohl der curricularen Produkte als auch der hiermit zusammenhängenden Arbeitsprozesse. Wir haben zu Beginn des Projektes die Bereiche Entwicklungsprozess und Implementation sowie Produkte analytisch voneinander unterschieden. Im Verlauf des Projektes wurde zunehmend deutlich, dass Entwicklungs- und Implementationsprozesse ineinander übergehen, sich gegenseitig bedingen und im Falle der Curriculumentwicklung immer auf die Produkte zu beziehen sind. Es ist nicht so, dass am Ende eines zeitlich begrenzten Entwicklungsprozesses fertige Produkte zur Verfügung stünden, die dann zu implementieren wären. Die Erfahrungen, die wir in EvaNet-EH sammelten, zeigen deutlich, dass die curricularen Produkte nur einen zurzeit akzeptierten Status erreicht haben, der jedoch künftig angepasst, angereichert, konkretisiert und überarbeitet werden muss. Diese Arbeit an den curricularen Produkten und die damit verbundenen Arbeitsstrategien, Teamstrukturen und sonstigen Rahmenbedingungen bezeichnen wir als *Implementationsprozess*.

In Abschnitt 4.1 fassen wir den bei Projektende erreichten Status von EvaNet-EH zusammen und beschreiben somit den Ausgangspunkt für den weiteren Implementationsprozess. In Abschnitt 4.2 wird erläutert, wie wir die Einschätzungen der Schulleitungen zu diesem Thema erfasst haben. In Abschnitt 4.3 werden die wesentlichen Aussagen der Interviews mit den Schulleitungen zusammengefasst. Schließlich empfehlen wir in Abschnitt 4.4 die Schaffung bestimmter Bedingungen für die künftige curriculare Arbeit.

4.1 Der Status bei Projektende als Ausgangspunkt zur Weiterentwicklung

Wie in Kapitel 3 beschrieben, sind alle 14 Lernfelder in das Planungsformat (curriculare Analyse und Strukturplanung) überführt und ausdifferenziert worden. Bei den Präsenztreffen, wurden die jeweiligen Lernfeldplanungen von Kollegen der Schulen intensiv und detailliert diskutiert und schließlich für vorläufig verbindlich erklärt. Es wurden sieben Kompetenzdimensionen erarbeitet, in denen jeweils die Beiträge der einzelnen Lernfelder zur Kompetenzentwicklung sowie eine komplexe Leitidee als Zusammenfassung dessen formuliert wurde, was die Schüler am Ende ihrer Ausbildung können und verstehen sollten. Die curricularen Produkte beider Arbeitsstränge sind in der Lernfeld-Kompetenz-Matrix zusammengefasst. Diese Matrix kann im Detail sowohl in WiBeS unter https://www2.wibes.de/sites/eh/default.aspx als auch auf der folgenden Homepage http://www.ibw.uni-hamburg.de/evaneteh/index.php/curriculare-ergebnisse/kompetenzmatrix.html eingesehen werden. Die wissenschaftliche Begleitung übergab die Dokumente des letzten Arbeitsstandes an die Lernfeldkoordinatoren in digitaler Form. Sie sind ebenfalls diesem Bericht als Anhang beigefügt.

In jeder Schule stehen die aktuellen Lernfeldplanungen sowie die Ausdifferenzierungen der Kompetenzdimensionen jedem Lehrer zur Verfügung.

Künftig wird der Lernfeldunterricht verbindlich entlang dieser Planungsunterlagen ausgerichtet.

An der Erstellung dieser *schulübergreifend verbindlichen* curricularen Planungsunterlagen beteiligten sich die Mitglieder der Planungsteams (aus jeder Schule drei Kollegen) und der Kompetenzentwicklungsgruppe (aus jeder Schule 1-2 Kollegen). Bereits während dieser Entwicklungsphase wurde auf die Erfahrungen bestimmter Kollegen zurückgegriffen, bis sich schließlich bei den Präsenztreffen der Großteil des Kollegiums mit den Planungen auseinandersetzte. Damit dürfte sichergestellt sein, dass alle Kolleginnen und Kollegen des Lernbereichs I die verbindlichen Planungen kennen.

Ansatzpunkte einer schulübergreifenden wie auch einer schulspezifischen Weiterentwicklung sind sowohl auf der Ebene der Produkte als auch auf der Ebene der Arbeitsprozesse zu finden:

	Produkte	Prozesse
Schulintern	• Wie werden die Unterrichtserfahrungen in die Planungsunterlagen aufgenommen?<br>• Wie werden die Planungsunterlagen branchenspezifisch konkretisiert?<br>• Wie wird das Unterrichtsmaterial den Planungsdokumenten zugeordnet?<br>• Welches sind Qualitätskriterien für Unterrichtsmaterial, wer bewertet das?<br>• Wie wird die Aktualität, Transparenz und Zugänglichkeit der Dokumente gesichert?<br>• …	• Wer koordiniert den Prozess, dass Erfahrungen explizit aufgenommen und in die Planungsdokumente eingearbeitet werden?<br>• Wer übernimmt diese Überarbeitungen?<br>• Wie werden curriculare Planungsteams zusammengestellt, welche Aufgaben werden definiert?<br>• Wie wird diese Arbeit im Rahmen des Arbeitzeitmodells bewertet?<br>• …
Schul-übergreifend	• Wie werden Anregungen zur Anpassung der Lernfeldplanungen und Kompetenzdimensionen aufgenommen und eingearbeitet?<br>• Wie wird weiterhin die Verbindlichkeit und Aktualität der Planungsdokumente sichergestellt?<br>• Wie kann das Qualitätsniveau der Planungsdokumente sichergestellt werden?<br>• Wie kann sichergestellt werden, dass die lernfeldübergreifende Perspektive kontinuierlich überprüft, weiter ausdifferenziert und mit den lernfeldspezifischen Ausarbeitungen abgeglichen wird?<br>• …	• Wer übermittelt schulinterne Änderungswünsche in den schulübergreifenden Prozess?<br>• Wie können Anpassungen und Veränderungen verhandelt werden?<br>• Wie oft finden Präsenztreffen statt? Wer organisiert diese, welche inhaltliche Ausrichtung haben diese?<br>• Auf welcher Ebene (SL, AL, LFK und Lehrer) soll welche Art von Austausch stattfinden?<br>• Wer sorgt für die Pflege der schulübergreifenden Zugriffsmöglichkeiten (WiBeS und Homepage)?<br>• …

Tabelle 12: Ansatzpunkte zur Weiterentwicklung

Die Auslassungspunkte deuten an, dass diese Liste nicht den Anspruch auf Vollständigkeit erhebt. Sie enthält aber wesentliche Arbeitsschwerpunkte, die während des Projektzeitraumes teilweise von der wissenschaftlichen Begleitung wahrgenommen wurden. Diese Tätigkeiten müssten nunmehr von den Schulen übernommen werden. Die Erstellung von Unterrichtsmaterial sowie die branchenspezifische Konkretisierung der Planungsunterlagen ist nun die dringendste Aufgabe, da nur mit diesen Makroplanungen die Güte auch der curricularen Analyse und der Strukturplanung erprobt werden kann. Einen Arbeitsstrang bilden die schulinterne Konkretisierungsarbeit und die systematische Erfassung von Unterrichtserfahrungen als Evaluationsgrundlage der Lernfeld-Kompetenzmatrix. Ein zweiter Arbeitsstrang ist der schulübergreifende Austausch hierüber und die Sicherstellung der kontinuierlichen Fortschreibung des verbindlichen Curriculums.

4.2 Interviews mit den Schulleitungen zur Erfassung der Einschätzungen

Die Frage, wie der curriculare Entwicklungsprozess fortgeführt und dauerhaft etabliert werden kann, weist naturgemäß über die Laufzeit des Projekts hinaus. Im Juli 2009 beschlossen wir dennoch, zumindest die derzeitige Einschätzung der Schulleitungen zur Implementationsproblematik zu erfragen. Hierzu führten wir leitfragengestützte Interviews durch. In Anlehnung an die Überlegungen zur Implementation orientierten wir uns an folgenden Leitfragen:

Leitfragen für Abschlussinterviews mit Schulleitungen:

- Wie würden Sie die **Stimmung in Ihrem Kollegium** zu EvaNet beschreiben?
- Welches sind für Sie die herausragenden **Erfolge des Projektes** EvaNet?
- Was hat sich durch das Projekt an **Ihrer Schule geändert**?
- Beschreiben Sie aus **Ihrer Sicht** den **Nutzen** des Projekts und/oder der erstellten Produkte? (Erkenntnisse,Verhalten)
- Wie stellen Sie sich idealtypisch die Nutzung der **Lernfeldplanungen** durch die Kollegen vor? (**lernfeldspezifisch, bezogen auf konkreten Unterricht**)
- Wie können die kontinuierliche **Weiterentwicklung** der Lernfeldplanungen gesichert und **Rückkopplungsprozesse** gewährleistet werden?
- Worin bestünde der **Schwerpunkt** der Tätigkeiten in Bezug auf LF-Planungen
- Wer ist hierfür **verantwortlich** und wie können diese Prozesse **institutionalisiert** werden?
- Wie stellen sie sich idealtypisch die Nutzung der Kompetenzmatrix vor, damit die Kollegen die **Kompetenzentwicklung** der Schüler über die Lernfelder hinweg im Auge behalten? => (**lernfeldübergreifend, bezogen auf konkreten Unterricht)**
- Wie können die kontinuierliche **Weiterentwicklung** der Kompetenzdimensionen gesichert und **Rückkopplungsprozesse** gewährleistet werden?
- Worin bestünde der **Schwerpunkt** der Tätigkeiten in Bezug auf LF-Planungen
- Wer ist hierfür **verantwortlich** und wie können diese Prozesse **institutionalisiert** werden?
- Wie wird künftig die schulübergreifende **Kooperation** organisiert, worin bestehen die curricularen Aufgaben und Arbeitsprozesse?

Abbildung 22: Leitfragen für die Abschlussinterviews

Teilweise wünschten die Schulleiter, die Abteilungsleiter zu den Interviews hinzuzuziehen (H6, H13) bzw. empfahlen uns, ergänzend noch ein Interview mit diesem zu führen (H1). Die Interviews fanden im Juli bzw. August 2009 an den beteiligten Schulen statt. Sie wurden transkribiert und den Interviewten anschließend

noch einmal zur Durchsicht und ggf. Korrektur vorgelegt. Alle Interviewpartner waren einverstanden, dass in diesem Bericht Inhalte der Interviews einfließen dürfen. Aus Zeitgründen konnte bisher noch keine systematische inhaltsanalytische Auswertung vorgenommen werden. Deswegen werden im folgenden Abschnitt im Vorgriff auf eine solche systematische Analyse die wesentlichen Aussagen der Interviews kurz zusammengefasst und schließlich der subjektive Gesamteindruck der Interviewerin dargestellt.

4.3 Zusammenfassungen der Interviews und Gesamteindruck

4.3.1 Interviews an der H1

Der herausragende Nutzen des Projektes war aus Sicht der Schulleitung der H1, dass die an der Projektarbeit beteiligten Kollegen erheblich mehr Sicherheit im Umgang mit dem neuen Curriculum erlangt haben (AL: „Viele… sind froh, dass jetzt klar ist, wie sie die Ansprüche [KMK-Vorlagen, Anmerkung des Verfassers] umsetzen können“). Bereits während des Projektzeitraums gab es an der H1 eine Reihe von neuen und jungen Kollegen, die auf das in EvaNet-EH erstellte curriculare Material zurückgriffen. Der herausragende Nutzen sei die Orientierungsleistung, Systematik und Verlässigkeit der Unterlagen. Diese positiven Effekte hätten die Akzeptanz der Produkte und der curricularen Arbeit gesteigert. Es gäbe jetzt mehr Bereitschaft und Einsicht in die Notwendigkeit, curriculare Arbeit kontinuierlich durchzuführen. Die Kollegen seien weitgehend zufrieden mit den Projektergebnissen.

Eine Besonderheit an der H1 ist die Funktion des Lernfeldkoordinators als Multiplikator. Er sorgt nicht nur dafür, die Planungsunterlagen aus dem Projekt in der Schule zugänglich zu machen, sondern er organisiert und leitet Arbeitsgruppen, die Konkretisierungen bis hin zum Unterrichtsmaterial vornehmen und systematisch an die Planungsunterlagen andocken. Die Zufriedenheit der Kollegen wird weitgehend der Arbeit des Lernfeldkoordinators zugeschrieben, da sich alle darauf verlassen (können), dass er die Planungsunterlagen konkretisiert und für den Unterricht nutzbar macht (SL: „Wir haben das große Glück, dass bei uns durch Matthias Odinga alles [Planungsunterlagen der Lernfeld-Kompetenz-Matrix – Anmerkung des Verfassers] wunderbar runtergebrochen wird und sich alle darauf verlassen. Wir wissen, er verändert das und macht es für uns machbar...“).

Die Interviewten betonten, dass die Kollegen die Gesamtheit der Matrix wahrscheinlich noch nicht erfasst haben. Zwar sei die Idee der lernfeld-übergreifenden Kompetenzen angekommen, aber es gäbe noch Unsicherheiten, wie dies im Unterricht konkret umzusetzen wäre. Aus Sicht der Schulleitung ist klar, dass die curricularen Planungsunterlagen kontinuierlich angepasst und weiterentwickelt werden müssen. Diese Arbeit könne aus Ressourcengründen nur von wenigen Kollegen übernommen werden. Eine Arbeitsstrategie hierfür sei noch nicht verabredet.

Die Interviewten erklärten, dass auch auf der Ebene des Kollegiums schulübergreifende Rückkoppelungsprozesse erwünscht seien. Dies habe sich durch die Projektarbeit geändert, denn anfangs war man skeptisch, ob dies einen Nutzen hätte. Die durch das Projekt etablierte Arbeitsstrategie (Präsenztreffen mit schulgemischten Arbeitsgruppen) wurde im Kollegium positiv bewertet, sodass man sich auch in Zukunft diesen Austausch wünscht, aus Ressourcengründen allerdings höchstens zweimal im Jahr und nur mit einigen Repräsentanten des Kollegiums.

Genauere Verabredungen zur schulübergreifenden Kooperation würden erst noch verabredet.

4.3.2 Interview an der H6

Beide Interviewten beschrieben die Stimmung im Kollegium als gut aber erleichtert darüber, dass die „Kraftanstrengung“ vorbei sei. Die Kollegen freuten sich über die „grandiosen“ Planungsunterlagen, die ihnen einen Ausgangspunkt für weitere Konkretisierungen böten. Die Lernfeldplanungen seien für neue und jüngere Kollegen aufgrund der Klarheit eine große Hilfe. Die Mehrheit sei der Meinung, dass dieser Arbeitsstand ohne EvaNet nicht erreicht worden wäre.

Herausragender Erfolg sei ebenfalls, dass sich die schulübergreifende Kooperation auf Ebene der Schul- und Abteilungsleiter verstetigt habe. Die Präsenztreffen hätten Rückkoppelungsprozesse zwischen den Kollegien ermöglicht, sodass die schulübergreifende Kooperation auf allen Ebenen initiiert und intensiviert wurde.

Während des Projektzeitraumes wurden Lernfeldordner neu eingerichtet, die jedem zur Verfügung stünden und kontinuierlich gepflegt würden. Es gäbe jetzt mehr Austausch und mehr Kommunikation zwischen den Kollegen. Der Teamgedanke sei durch das Projekt an der H6 gefördert worden.

Diese positiven Effekte sollen nachhaltig gesichert werden. Ab dem laufenden Schuljahr wird ein Arbeitskreis gebildet, der sich aus den Lehrern zusammensetzt, die mit dem ersten Ausbildungsjahr beginnen. Die Teilnahme sei verpflichtend; es würden ca. 0,5 Funktionsstunden angerechnet. Die Arbeitskreise sollen zum „Anschub“ bis zum dritten Ausbildungsjahr unterstützt werden. Im nächsten Schuljahr wird ein weiterer Arbeitskreis mit denjenigen gebildet, die dann mit dem ersten Ausbildungsjahr beginnen. Auf diese Weise sollen in einem dreijährigen Prozess alle Kollegen an der curricularen Arbeit beteiligt werden. Ob während des gesamten Zeitraums alle Kollegen Funktionsstunden erhalten, könne nicht vorhergesehen werden. Nach drei Jahren solle die curriculare Arbeit nachhaltig etabliert sein. Die Arbeitstreffen seien wöchentlich geplant und würden durch die Lernfeldkoordinatorin angeleitet.

Die Aufgaben können aus folgender Situationsbeschreibung rekonstruiert werden: „Vor EvaNet hatten wir Unterrichtsmaterial zu den Lernfeldern, aber keinen Plan und jetzt haben wir die Lernfeldplanungen aber kein passendes Unterrichtsmaterial.“ Die wesentliche Aufgabe sei, die curricularen Planungen zu konkretisieren. Dabei würden zunächst die Kompetenzschwerpunkte der Lernfelder vergegenwärtigt, um das Unterrichtsmaterial diesen anzupassen. Nur so sei gewährleistet, dass man die lernfeldübergreifenden Kompetenzdimensionen nicht aus dem Auge verlöre. Hierauf zu achten, sei die wesentliche Aufgabe der Lernfeldkoordinatorin.

Das Gesamtkonstrukt der Lernfeld-Kompetenz-Matrix sei von den Lehrern verstanden worden. Die Interviewten räumten jedoch ein, dass die Umsetzung aufgrund der hohen und neuen Ansprüche noch Zeit bräuchte. Auch um diesen Lernprozess zu unterstützen, seien die Arbeitskreise eingerichtet worden. „Hier kommen die Lernfeldplanungen immer wieder auf den Tisch und die Diskussion wird am Leben erhalten“.

Die schulübergreifende Kooperation soll erhalten bleiben. Die Schulleitung der H6 wünscht sich 1-2 Präsenztreffen pro Schuljahr, nach dem Muster der EvaNet-Treffen. Eine Informationsplattform zur Unterstützung der schulübergreifenden Kooperation hielte die Schulleitung für notwendig, WiBeS scheint hierfür aufgrund der

mangelnden Akzeptanz aber nicht geeignet. Die im Projekt erstellte Homepage weckt die Hoffnung, geeigneter zu sein, wobei die Frage der kontinuierlichen Pflege ungeklärt sei.

4.3.3 Interview an der H11

Der Schulleiter nannte Einstellungsänderungen seines Kollegiums bezüglich lernfeldstrukturierter Curricula als wesentliche Wirkungen der Projektarbeit. Die Bereitschaft zu curricularen Diskursen und zur Konzeptarbeit sei gewachsen. Die Kollegen haben den Nutzen eines gemeinsamen und verbindlichen Curriculums erkannt. Die Nachfrage nach curricularen Planungsunterlagen sei angestiegen. Ebenso hervorzuheben sei die schulübergreifende Kooperation in Form eines curricularen Arbeitsprozesses, an dem sich fast alle betroffenen Kollegen beteiligten. Auch die intensive Kooperation mit der Universität habe es in dieser Form bisher nicht gegeben.

Durch die Projektarbeit verstetigten sich die mit dem Lernfeldkonzept verbundenen curricularen Ideen. Mit Hilfe der Planungsformate konnten diese Ideen für die Lehrer konkretisiert und fassbarer gemacht werden. Der jetzige Planungsstand sei somit eine gute Ausgangslage, um weitere Konkretisierungen vorzunehmen. Allerdings glaubt der Schulleiter auch, dass die Lernfeld-Kompetenz-Matrix noch weiter „durchdekliniert“ werden muss, damit alle Lehrer die Orientierungsleistung erfahren. Die Gesamtheit der Matrix sei noch nicht so angekommen, dass sie im Unterrichtsalltag spürbaren Nutzen brächte. Die lernfeldübergreifende Perspektive und damit die Frage nach der Kompetenzentwicklung der Schüler sei noch unzureichend transparent. Dieser Arbeitsstrang soll weiterhin explizit verfolgt werden.

Die H11 hat in Hinblick auf die Verstetigung der curricularen Arbeitsprozesse die notwendige Konkretisierungsarbeit als Teil der nächsten Zielleistungsvereinbarung aufgenommen. Die entscheidende Arbeit sei nun, branchenspezifisches Unterrichtsmaterial zu erstellen. Mit Beginn des Schuljahres hat sich eine Arbeitsgruppe aus den Lehrern gebildet, die die neuen Klassen übernahmen. Somit werden im laufenden Schuljahr (09/10) die Lernfelder des ersten Ausbildungsjahres auf der Ebene der Makroplanung konkretisiert. Die Arbeitsgruppe überarbeitet bei Bedarf auch die Planungsunterlagen der Lernfeld-Kompetenz-Matrix. Im nächsten Schuljahr werden dann andere Kollegen die Unterrichtsmaterialien für die Lernfelder des ersten Ausbildungsjahres evaluieren und ergänzen, während die Kollegen des ersten Jahres die Lernfelder des zweiten Ausbildungsjahres bearbeiten, im dritten Jahr dann die des dritten Ausbildungsjahres. Auf diese Weise werden alle Kollegen an der Ausarbeitung aller Lernfelder beteiligt. Der Prozess ist auf drei Jahre angelegt. Die Kollegen sollen nach Möglichkeit für diese curriculare Arbeit, sofern sie Gegenstand der ZLV bleibt, Entlastungsstunden erhalten.

Die schulübergreifende curriculare Zusammenarbeit würde im Wesentlichen durch die Lernfeldkoordinatorin gesteuert, deren Aufgabe es sei, die Erfahrungen und Überarbeitungswünsche zu den Planungsunterlagen an die anderen Schulen zu übermitteln. Auch auf Ebene der Kollegen solle es weiterhin einen Austausch geben. Hierzu wären ein bis zwei Präsenztreffen jährlich denkbar, zu denen man sich auch die Beteiligung der wissenschaftlichen Begleiter wünschte. Wichtig wäre allerdings, dass die Kollegen das Gefühl hätten, dass die anderen Schulen ebenso intensiv und zielstrebig an diesem Prozess mitarbeiteten.

Der Umfang des Ressourcenaufwands wird in Hinblick auf andere wichtige Entwicklungsbereiche der Schule angepasst. Durch die Aufnahme der curricularen

Entwicklungsarbeit in die Zielleistungsvereinbarung seien jedoch Ressourcen gesichert.

4.3.4 Interview an der H13

Die Interviewten beschrieben als wesentliches Verdienst des Projektes die sozialen Kommunikationsprozesse, die zwischen den Schulen entstanden seien und die es auf Ebene der Kollegien vorher nicht gab. Auch auf Ebene der Abteilungsleiter sei die Zusammenarbeit durch das Projekt intensiviert worden. Ein spürbarer Konkurrenzdruck unter den Schulen habe den positiven Effekt gehabt, dass mit viel Engagement und hoher Motivation an der Qualitätsverbesserung der curricularen Planungsunterlagen gearbeitet wurde.

Die im Projekt erarbeiteten Planungsunterlagen sind als verbindliche Orientierung zur Unterrichtsplanung in Ordnern im Lehrerzimmer hinterlegt. Die Ordner werden durch Lernfeldpaten gepflegt, die gemeinsam eine Arbeitsgruppe bilden, um die Planungsunterlagen kontinuierlich zu überarbeiten, zu konkretisieren und den Unterrichtserfahrungen anzupassen. Diese Arbeitsgruppe besteht aus weniger als 14 Personen, da einige Paten mehrere Lernfelder betreuen. Wie der Rückkoppelungsprozess mit den anderen Schulen aussehen wird, sei noch unklar. Der Lernfeldkoordinator solle mit den Kollegen der anderen Schulen eine Strategie für die schulübergreifende curriculare Weiterentwicklung erarbeiten.

Die Schulleitung betont, dass es künftig eine schulübergreifende Kooperation bezogen auf das Curriculum geben wird. Selbstverständlich müssten zunächst auch andere Bereiche mit Ressourcen ausgestattet werden. Auf Ebene der Lernfeldkoordinatoren und Abteilungsleiter gäbe es auf jeden Fall eine kontinuierliche Zusammenarbeit. Die Schulleitung glaubt, dass auf Ebene des Kollegiums gewisse Ermüdungserscheinungen durch die Intensität der curricularen Arbeit während des Projektzeitraumes aufgetreten seien, sodass man zunächst froh wäre, jetzt „ruhiger“ arbeiten zu können. Dies bezöge sich sowohl auf schulinterne als auch auf schulübergreifende Rückkoppelungsprozesse. Man wolle das „verbindlich“ Verabredete nun erst einmal ausprobieren.

Während des Projektzeitraumes wurden an der H13 nicht zuletzt aufgrund der Rückspiegelungen in den Zwischenberichten, „stabile Kollegenteams“ eingerichtet, die gemeinsam den Lernfeldunterricht durchführen. Man habe auf diese Weise Kommunikationsstrukturen etablieren können. Die Kollegen diskutierten über curriculare Fragen, um mit der Komplexität der Lernfeldplanungen umzugehen. Allerdings ist das „Gesamtkunstwerk“, die Lernfeld-Kompetenz-Matrix, aus Sicht der Schulleitung eher noch nicht zur Kenntnis genommen. Dies betrifft insbesondere die lernfeldübergreifende Perspektive, deren Notwendigkeit die Kollegen erkennen, aber noch Unsicherheiten haben, welche Konsequenzen dies für den Unterricht habe.

4.3.5 Zusammenfassender Eindruck aus den Interviews

Alle Schulleiter(innen) betonten, dass die intensive Zusammenarbeit der vier Einzelhandelsschulen das herausragende Merkmal der Projektarbeit gewesen sei. Eine große Anzahl von Kollegen sei miteinander in einen curricularen Kommunikationsprozess getreten und habe es geschafft, gemeinsam Verbindlichkeiten herzustellen. Das Projektergebnis in Form der Lernfeld-Kompetenz-Matrix wird als ein gemeinsames Produkt angesehen und die Steigerung des Qualitätsniveaus anerkannt. Ein positiver Nebeneffekt sei auch, dass sich die

Zusammenarbeit auf der Abteilungsleiter- und Schulleiterebene ebenfalls intensiviert habe.

Die enge Zusammenarbeit zwischen Wissenschaft und Praxis, d. h. die Kooperation mit dem Institut für Berufs- und Wirtschaftspädagogik bezeichneten die Schulleitungen als sehr bereichernd und als Novum in Hamburg.

Wenngleich die derzeitigen Planungen zur künftigen curricularen Arbeit sich noch in unterschiedlichen Stadien befinden und sich auch verschiedene schulische Strategien abzeichnen, besteht Einigkeit darüber, dass die Lernfeld-Kompetenz-Matrix einerseits branchenspezifisch konkretisiert, d. h. auf der Ebene der Makroplanung mit Unterrichtsmaterial angereichert werden muss und dass andererseits die Lernfeldplanungen nach einer Bewährungsprobe weiter überarbeitet werden müssen. Es ist durch die Projektarbeit gelungen, schulische und schulübergreifende curriculare Diskurse anzuregen und die hierzu notwendige Sprache und Formate einzuführen. Dadurch konnte gemeinsam das verbindliche Curriculum für den Berufsschulunterricht im Einzelhandel erarbeitet werden. Die lernfeldübergreifende Perspektive in Form der Kompetenzdimensionen ist den Kollegen einsichtig, jedoch noch nicht handhabbar und in den Konsequenzen noch nicht hinreichend präzisiert. Hier wünscht man sich weitere Unterstützung seitens der Universität.

Die im zweiten Projektjahr durchgeführten Präsenztreffen sind sowohl in ihrer Ausgestaltung als auch Effektivität lobend hervorgehoben worden. Es besteht der Wunsch, diese Arbeitsstrategien beizubehalten (mit weniger Ressourcenaufwand und seltener) und hierfür auch die Hilfe der wissenschaftlichen Begleitung anzufordern.

4.3.6 Empfehlungen für die künftige curriculare Arbeit an den vier Einzelhandelsschulen

Aus den Zusammenfassungen der Interviews im vorherigen Abschnitt lassen sich die herausragenden Erfolge des Projektes ablesen, die zugleich die Anknüpfungspunkte für die weitere kooperative Curriculumarbeit sein können. Sie beziehen sich einerseits auf die intensive schulübergreifende Zusammenarbeit und Kommunikation auf unterschiedlichen schulischen Ebenen und andererseits auf die Lernfeld-Kompetenz-Matrix als zentrales curriculares Produkt. Diese Erfolge und die Zufriedenheit mit dem Projekt sind u. E. darauf zurückzuführen, dass die Empfehlungen, die wir zur Halbzeit des Projektes aufgrund der Evaluationsbefunde formulierten, weitestgehend angenommen und umgesetzt wurden. Es liegt deswegen Nahe, Gelingensbedingungen für die künftige Kooperation und curriculare Entwicklungsarbeit entlang dieser Empfehlungen zu rekonstruieren:

- **Revolvierender Entwicklungs- und Implementationsprozess:**

Curriculare Entwicklungs- und Implementationsprozesse sind eng miteinander verbunden. Schnittstelle sind jeweils die Prozessergebnisse in Form von curricularen Produkten, die zugleich immer auch Ausgangspunkt eines neuen Entwicklungsprozesses sind. Folgende Abbildung macht dies deutlich.

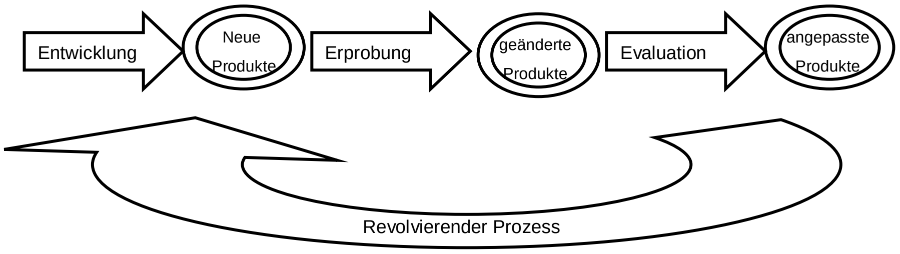

Abbildung 23: Revolvierender Prozess

Wir empfehlen dieses Gedankenmodell der künftigen curricularen Arbeit zugrunde zu legen, da es kontinuierliche Evaluation, längerfristige Planungshorizonte und dynamische Anpassung impliziert.

- **Von der Revitalisierung zur Verstetigung:**

Die Revitalisierung des curricularen Entwicklungsprozesses dürfte gelungen sein. Wichtig ist nun, sowohl schulintern als auch schulübergreifend durch das Projekt etablierte Strukturen und Prozesse bei vertretbarem Ressourceneinsatz[10] beizubehalten:

Schulintern ist sicherzustellen, dass Zeit und Raum für curriculare Diskurse geschaffen werden. Wichtigste Arbeiten sind die Konkretisierung der Lernfeldplanungen, die systematische Verknüpfung der Unterrichtsmaterialien mit dem Planungsformat, im Bedarfsfall die Überarbeitung der curricularen Analysen und der Strukturplanungen, die Sicherstellung der Aktualität und Zugänglichkeit der Planungsdokumente sowie die Verständigung über die Verbindlichkeit curricularer Verabredungen. Gelingensbedingungen wären die Schaffung klarer Zuständigkeiten sowie die Bildung curricularer Arbeitskreise mit definierten Aufgaben, Zielen und Zeitvorgaben. Wichtig sind ebenfalls die Berücksichtigung des Transfers in das gesamte Kollegium und die Einigung auf ein allseits akzeptiertes Informationssystem.

Schulübergreifend ist sicherzustellen, dass schulinterne Überarbeitungen und Anpassungen in den Dokumenten der Lernfeld-Kompetenz-Matrix in einen schulübergreifenden Aushandlungsprozess treten. Dabei ist situativ zu entscheiden, welcher Personenkreis geeignet ist. An die hohe Akzeptanz und Effektivität der Präsenztreffen sollte angeknüpft werden. Um die schulübergreifenden curricularen Diskurse prinzipiell aufrechtzuerhalten, sollte mindestens einmal im Jahr ein Treffen mit Beteiligung von Kollegen aller vier Schulen stattfinden. Wichtigste curriculare Aufgabe ist die kontinuierliche Verständigung über die Verbindlichkeit und Begründetheit des gemeinsamen Curriculums. Gelingensbedingung wäre die Definition von Schnittstellen zwischen schulinternen Entwicklungsprozessen und dem schulübergreifenden Diskurs. Es muss geklärt werden, wer zu welchem Zeitpunkt mit welchen inhaltlichen Diskussionsbedarf miteinander in Verhandlung tritt. Die

[10] Diese Wendung hat zwei Facetten: Ressourcen hierfür werden weiterhin notwendig sein – Innovation im curricularen Bereich ist nicht zum Nulltarif zu haben. Und zweitens: Aufwand und Ertrag müssen in einem (auch im Hinblick auf Opportunitätskosten) vertretbaren Verhältnis zueinander stehen.

Organisation der obligatorischen Präsenztreffen sollte den Lernfeldkoordinatoren übertragen werden. Zur inhaltlichen und strukturellen Ausrichtung empfehlen wir die Inanspruchnahme unserer wissenschaftlichen Begleitung.

- **Die lernfeldübergreifende Perspektive der Kompetenzdimensionen**

Im Projektzeitraum beinhaltete diese Perspektive einen eigenen Arbeitsstrang, dessen Verknüpfung zur lernfeldspezifischen Perspektive weitestgehend von der wissenschaftlichen Begleitung geleistet wurde. Die weitere Ausdifferenzierung und Anpassung dieser Ausarbeitungen an die Erfordernisse der schulischen Unterrichtsplanungen und Erfahrungen muss künftig gewährleistet sein. Offenbar ist die curriculare Idee bei der Mehrheit der Kollegen angekommen und akzeptiert. Es gibt aber weiteren Konkretisierungsbedarf in Hinblick auf die Konsequenzen für die Unterrichtsplanung und –durchführung. Diese Unklarheiten müssen erfasst und Fortbildungsbedarf muss formuliert und organisiert werden. Da die Kompetenzformulierungen Teil der Lernfeldplanungen sind, muss diese Arbeit parallel zur weiteren lernfeldspezifischen Arbeit erfolgen. Sonst wäre die Stimmigkeit des Gesamtcurriculums nicht mehr gewährleistet. Wir empfehlen daher, diesen Arbeitsstrang in die curricularen Arbeitskreise zu integrieren.

- **Die lernfeldspezifischen Planungsunterlagen**

Der zum Abschluss des Projektes übergebene Arbeitsstand zu den Lernfeldplanungen ist ebenso verbindlich wie vorläufig. Wichtig ist, die kontinuierliche Evaluation dieser Planungen. Zur Erprobung müssen schulspezifische Konkretisierungen vorgenommen werden. Jede Schule muss geeignete Strategien entwickeln, wie diese curriculare Arbeit organisiert wird. Entsprechend der Erfahrungen müssen die Planungsformate angepasst werden. Wichtig ist hierbei einerseits, die lernfeldübergreifenden Kompetenzdimensionen ebenfalls anzupassen und andererseits, diese Anpassungen in den schulübergreifenden Prozess zu transportieren. Über Präsenztreffen müssen dann jeweils Verbindlichkeiten neu verabredet werden. Gelingensbedingung ist neben der Verabredung klarer Strategien, Aufgaben und Zuständigkeiten die Beibehaltung des Planungsformats als Grundlage einer gemeinsamen Sprache und Arbeitsunterlage.

- **Curriculare Arbeitskreise und curriculare Konferenzen**

Curriculare Entwicklungsarbeit lebt von Diskussion und Aushandlungsprozessen, d. h. von Kommunikation. Lehrer müssen miteinander im Team an den curricularen Produkten arbeiten. Für die schulspezifischen Konkretisierungen empfehlen wir Arbeitskreise einzurichten, deren Aufgaben genau zu definieren sind. Die Leitung und Koordination sollten die Lernfeldkoordinatoren leisten können. Um Verbindlichkeiten und curriculare Verabredungen sowie Transfer herzustellen, müssten regelmäßig curriculare Konferenzen stattfinden. Diese sind schulintern durchzuführen (Bildungsgangkonferenzen mit Schwerpunktverlagerung zum curricularen Diskurs), um das gesamte Kollegium über das Fortschreiten der curricularen Entwicklungen auf dem Laufenden zu halten und schulübergreifend, um den Grad der Verbindlichkeit und die Veränderung der curricularen Analysen und Strukturplanungen zu verhandeln. Die Präsenztreffen wären solche schulübergreifenden curricularen Konferenzen.

- **Aufgaben und Kompetenzen der Lernfeldkoordinatoren**

Wir empfehlen den Lernfeldkoordinatoren folgende Aufgaben und Kompetenzen zuzuweisen und hierfür die entsprechenden Ressourcen zur Verfügung zu stellen:

Die Lernfeldkoordinatoren steuern die gesamte curriculare Entwicklungsarbeit und sorgen für die Dokumentation der Ergebnisse. Schulintern leiten sie die Arbeitsgruppen und achten darauf, dass nach den im Projekt etablierten Qualitätsstandards gearbeitet wird. Das bedeutet, dass sie das Planungsformat beibehalten, die schulinternen Konkretisierungen hier andocken (Makroplanung) und dass sie die Kompetenzdimensionen immer wieder zur Diskussion stellen und anpassen. Sie arbeiten inhaltlich mit und haben die Kompetenz, curriculare Produkte zu bewerten und rückzuspiegeln. Sie sorgen für die Aktualität der curricularen Planungen in den Informationssystemen. Die Lernfeldkoordinatoren könnten die Schnittstelle für die schulübergreifende Kommunikation sein. Entweder sie verhandeln schulübergreifend relevante curriculare Probleme im Kreise der Lernfeldkoordinatoren oder sie sorgen dafür, dass entsprechende Kollegen zusammentreffen. Sie organisieren regelmäßig Präsenztreffen in Form von schulübergreifenden curricularen Konferenzen.

- **Informationssysteme**:

Wir empfehlen, sowohl schulinterne Informationssystem als auch ein schulübergreifendes, z. B. als Homepage zur Ablage der curricularen Produkte zu etablieren. Wichtig ist die Akzeptanz und die schnelle und sichere Zugriffsmöglichkeit dieser Orte. Die kontinuierliche Pflege muss personell geregelt sein.

- **Formen der kompetenzorientierten Lernerfolgskontrolle entwickeln**

Wir sehen die dringliche Notwendigkeit, parallel zur Implementation des kompetenzorientierten Lernfeldcurriculums der Entwicklung von Strategien und Instrumenten zur lernprozessbegleitenden Kompetenzdiagnostik und zur summativen Lernerfolgskontrolle hohe Aufmerksamkeit zu widmen. In diesem Komplex bündelt sich eine ganze Reihe wichtiger Funktionen und Herausforderungen:

- Wie können, bezogen auf die einzelnen Kompetenzdimensionen, die Lernausgangslagen der Schüler erfasst werden?
- Wie lassen sich angestrebte Entwicklungsschritte so operationalsieren, dass die Lehrenden Fortschritte auf Seiten der Schüler erkennen und zugleich auch den Erfolg ihres eigenen Unterrichts einschätzen können?
- Sind über das angestrebte Mindestniveau in den Kompetenzdimensionen auch von Schülern erreichte höhere Kompetenzgrade erfassbar und zertifizierbar?
- Wie können schulische Leistungsnachweise zu einzelnen Lernfeldern oder in Jahres- bzw. Halbjahreszeugnissen mit Bezug auf die Kompetenzdimensionen begründet und ausdifferenziert werden?

- Wie kann es gelingen, eine Perspektive kompetenz(dimensions)orientierter Leistungsmessung und –bewertung auch in die Zwischen- und Abschlussprüfungen der Kammern hineinzutragen.

Wir sehen hierin Fragestellungen, die weit über den Projektrahmen von EvaNet hinausweisen und auch für alle anderen Ausbildungsberufe geklärt werden müssen. Insofern empfehlen wir einerseits, in pragmatischer Hinsicht leistbare und dringliche Handlungsschritte zu identifizieren, zu priorisieren und zeitnah zu bearbeiten. Parallel dazu sollten die grundsätzlicheren Probleme und Fragestellungen an das HIBB herangetragen werden, um nach Wegen einer bildungsgangübergreifenden Bearbeitung im Rahmen von Forschungs- und Entwicklungsprojekten zu suchen.

- **Angestrebten Kompetenzen den Schülern transparent machen**

Aus dem Problemzusammenhang der Kompetenzdefinition und -erfassung heraus ist eine weitergehende wichtige Herausforderung zu identifizieren, mit der sich die Schulen teilweise schon intensiv auseinandersetzen, die aber klarer auf das Kompetenzdimensionenkonzept von EvaNet bezogen werden sollten.:

- Wie können Schülerinnen und Schülern die im Curriculum angestrebten Kompetenzen transparent dargestellt und nachvollziehbar begründet werden?
- Ist es möglich, die Kompetenzdimensionen auch dafür zu nutzen, dass Schüler sich mit ihrem eigenen Kompetenzprofil reflexiv auseinandersetzen, dieses auf die Qualifikationsanforderungen des Arbeitsmarktes beziehen und aus diesem Vergleich Konsequenzen für eine eigene, aktiv verfolgte Qualifizierungsstrategie entwickeln?
- Wie können Schüler in den Prozess der Kompetenzdiagnostik und der Lernerfolgskontrolle aktiv und reflexiv einbezogen werden?
- Lassen sich die Ansätze der Kompetenzdimensionenmatrix und der „Kompetenzraster“ miteinander verbinden?

Auch hier empfehlen wir die Unterscheidung zwischen relativ kurzfristig durch die beteiligten Schulen zu erarbeitenden Konzepten einerseits und eher mittelfristig zu klärenden, über den Kreis der in EvaNet beteiligten Schulen hinausweisenden, grundsätzlich-konzeptionellen Fragen andererseits.

- **Betriebliche Erfahrungen in die curricularen Planungen einbeziehen**

Eher kurzfristig anzugehen scheint uns die Herausforderung, die ausbildenden Betriebe stärker in den curricularen Diskurs der Schulen mit einzubinden. Dies könnte über eine unmittelbare Beteiligung an der Curriculumarbeit geschehen, indem Vertreter der Wirtschaft als Mitglieder in künftig zu bildende Arbeitsgruppen eingeladen werden. Es könnte aber auch in der Weise erfolgen, dass die Sichtweisen, Interessen und Positionen der Betriebe systematisch erhoben und damit im Prozess wirksam gemacht würden. Beides sollte sich schwerpunktmäßig auf die curricularen Analysen als Grundlage aller weiteren konstruktiv-didaktischen Überlegungen beziehen:

- Wie beurteilen die betrieblichen Vertreter die Referenzprozesse der einzelnen Lernfelder und die darin ausgewiesenen Kernprozesse, Varianten und Störungen? Entsprechen diese der betrieblichen Praxis, wo gibt es andere

oder ggf. auch verschiedene Varianten, welche Prozesse sind für die Praxis besonders relevant, welche irrelevant?

- Mit welchen betrieblichen Problemen, Konflikten, Aufgaben und Dilemmata sollten sich die Berufsschüler aus Sicht der Betriebe auseinandersetzen und welche Kompetenzen sollen in Bezug darauf erwerben?

Wie beurteilen die betrieblichen Vertreter die Ziele der einzelnen Kompetenzdimensionen? Diese Beurteilung sollte sich einerseits auf den Anforderungskomplex beziehen, der mit der Kompetenzdimension angesprochen wird. Ist dieser aus Sicht der Betriebe klar formuliert und relevant? Sie sollte sich andererseits auf das zu erreichende Kompetenzniveau beziehen und hier wären auch die Stufungen des Deutschen Qualifikationsrahmens mit in den Blick zu nehmen.

Literaturverzeichnis

Altrichter, H., Kannonier-Finster, W. & Ziegler, M. (2005). Das Theorie-Praxis-Verhältnis in den Sozialwissenschaften im Kontext professionellen Handelns. In: *Österreichische Zeitschrift für Soziologie*, Vol. 30, Nummer 1, 22 – 43.

Biggs, J. (2003). Teaching for Quality Learning at University. Maidenhead/Berkschire: Open University Press.

Brand, W.; Hofmeister, W. & Tramm, T. (2005). Auf dem Weg zu einem Kompetenzstufenmodell für die berufliche Bildung – Erfahrungen aus dem Projekt ULME. In: bwp@ Berufs- und Wirtschaftspädagogik – online, Ausgabe 8. Online: http://www.bwpat.de/ausgabe8/brand_etal_bwpat8.shtml (20.08.2009).

Hofmeister, W; Lesch, M.; Steinemann, S & Tramm, T. (2007). EvaNet-EH. Unveröffentlichter Zwischenbericht zum 31.07.2007. Hamburg.

KMK (2007). Handreichungenür die Erarbeitung von Rahmenlehrplänen der Kultusministerkonferenz für den berufsbezogenen Unterricht in der Berufsschule und ihre Abstimmung mit Ausbildungsordnungen des Bundes für anerkannte Ausbildungsberufe. Online: http://www.kmk.org/fileadmin/veroeffentlichungen_beschluesse/2007/2007_09_01-Handreich-Rlpl-Berufsschule.pdf (26.08.2009).

Meyer, H. (1971). Das ungelöste Deduktionsproblem in der Curriculumforschung. In: Achtenhagen & Meyer, Curriculumrevision - Möglichkeiten und Grenzen, München: Kösel, S. 106-132.

Neuweg, G. H. (2007). Wie grau ist alle Theorie, wie grün des Lebens goldner Baum? LehrerInnenbildung im Spannungsfeld von Theorie und Praxis. In: bwp@ Berufs- und Wirtschaftspädagogik – online, Ausgabe 12. Online: http://www.bwpat.de/ausgabe12/neuweg_bwpat12.shtml (20.08.2009).

Reetz, L. & Seyd, W. (2006). Curriculare Strukturen beruflicher Bildung. In: Arnold, R. (Hrsg.), Handbuch der Berufsbildung, Wiesbaden: VS, S. 227-259.

Reinisch, H. (2003). Zu einigen curriculumtheoretischen Implikationen des Lernfeldansatzes. Überlegungen anlässlich der Beiträge von CLEMENT, KREMER, SLOANE und TRAMM in bwp@ Ausgabe 4. In: bwp@ Berufs- und Wirtschaftspädagogik – online, Ausgabe 4. Online: http://www.bwpat.de/ausgabe4/reinisch_bwpat4.shtml (20.08.2009).

Robinsohn (1975). Bildungsreform als Revision des Curriculum. Neuwied, Berlin: Luchterhand.

Tramm, T. (1994). Die Überwindung des Dualismus von Denken und Handeln als Leitidee einer handlungsorientierten Didaktik. In: Wirtschaft und Erziehung, 46. Jg., 39-48.

Tramm, T. (1996). Lernprozesse in der Übungsfirma. Rekonstruktion und Weiterentwicklung schulischer Übungsfirmenarbeit als Anwendungsfall einer evaluativ-konstruktiven und handlungsorientierten Curriculumstrategie. Habilitationsschrift Göttingen.

Tramm, T. (2002). Kaufmännische Berufsbildung zwischen Prozess- und Systemorientierung. In: Tramm (Hrsg.), Perspektiven der kaufmännischen Berufsbildung, Entwicklungen im Spannungsfeld globalen Denkens und lokalen Handelns, Bielefeld: Bertelsmann, S. 22-35.

Tramm, T. (2003). Prozess, System und Systematik als Schlüsselkategorien lernfeldorientierter Curriculumentwicklung. In: bwp@ Berufs- und Wirtschaftspädagogik – online, Ausgabe 4. Online: http://www.bwpat.de/ausgabe4/tramm_bwpat4.shtml (20.08.2009).

Tramm, T. (2008). Lernfeldarbeit und Kompetenzdimensionen – Zur Struktur der curricularen Arbeit im Projekt EvaNet*EH. Unveröffentlichter Vortrag zum ersten Lernfeld-Präsenztreffen im Rahmen des Projektes EvaNet-EH. Hamburg.

Tramm, T. (2009). Vom geduldigen Bohren dicker Bretter – Antworten und Überlegungen eines „beglückten“ Kollegen zum Praxisbezug der Wirtschaftspädagogik. In: bwp@ Berufs- und Wirtschaftspädagogik – online, Profil 2. Online: http://www.bwpat.de/profil2/tramm_profil2.shtml (31.08.2009).

Tramm, T.; Hofmeister, W. & Lesch, M. (2008). EvaNet-EH. Unveröffentlichter Zwischenbericht II zum 15.02.2008. Hamburg.

Tramm, T., Steinemann, S. & Gramlinger, F. (2004). Der Modellversuch CULIK – Konzeption, Zwischenergebnisse und künftige Arbeitsschwerpunkte. In bwp@ Berufs- und Wirtschaftspädagogik – online, Spezial 1. Online: http://www.bwpat.de/spezial1/tramm-steinemann-gramlinger.shtml (31.08.2009).

Tyler, R. W. (1973). Curriculum und Unterricht. Düsseldorf: Schwann.

Zimmermann, W. (1977). Von der Curriculumtheorie zur Unterrichtsplanung. Paderborn: Schöningh.
