# Supplementary material for: Theme-centered interaction and developmental tasks as research method and pedagogical tool regarding identity development in VET
Source: Front Psychol. 2023 Oct 10;14:1201305. doi: 10.3389/fpsyg.2023.1201305 (PMC10597703; doi:10.3389/fpsyg.2023.1201305)
Supplement: Supplementary file 3 [file Data_Sheet_3.pdf]

Universität Hamburg

Fakultät Erziehungswissenschaften, Psychologie und Bewegungswissenschaft

Sektion Berufliche Bildung und Lebenslanges Lernen

Implementation und Evaluation kompetenzorientierten Lernfeldunterrichts im Ausbildungsberuf Einzelhandelskaufmann

Abschlussbericht (November 2015)

Author: Christiane Thole

Supervisor: Prof. Dr. Tade Tramm

Sedanstraße 19, 20146 Hamburg, Germany

https://www.ew.uni-hamburg.de/ueber-die-fakultaet/personen/tramm.html

Evanet EH website: http://evaneteh.ibwhh.de/

Inhaltsverzeichnis

INHALTSVERZEICHNIS .... **I**

ABKÜRZUNGSVERZEICHNIS .... **III**

ABBILDUNGSVERZEICHNIS .... **IV**

1 EINLEITUNG **1**

1.1 STAND DER LERNFELDCURRICULUMENTWICKLUNG IN FORSCHUNG UND PRAXIS 1

1.2 ZIEL DIESER ARBEIT UND VORGEHENSWEISE 2

2 DAS LERNFELDBASIERTE CURRICULUMENTWICKLUNGSPROJEKT EVANET-EH UND SEINE HEUTIGE PRAXIS **3**

2.1 CURRICULARE ENTWICKLUNGSPRODUKTE DES PROJEKTS 3

2.1.1 Die Lernfeld-Kompetenz-Matrix als theoriegeleitete curriculare Strategie *3*

2.1.2 Entwicklungsstand gemäß Abschlussbericht vom 11.09.2009 .... *4*

2.2 ERKENNTNISLEITENDES INTERESSE UND OPERATIONALISIERTE FRAGESTELLUNG 5

2.3 METHODE DER ERKUNDUNG 5

2.4 ERGEBNISSE DER UNTERSUCHUNG 6

2.4.1 Organisatorische Vorkehrungen zur Verstetigung des curricularen Entwicklungsprozesses *6*

2.4.2 Erstellung von Makroplanungen nebst Unterrichtsmaterial *7*

2.4.3 Umsetzung der Planungskonzepte in der Praxis *7*

2.4.4 Evaluativ-konstruktive Weiterentwicklung des Curriculums *9*

3 SCHLUSSBETRACHTUNG **11**

LITERATURVERZEICHNIS **14**

ANHANG 1: MAßNAHMENBÜNDEL ZUR VERSTETIGUNG DES PROJEKTERFOLGES **16**

ANHANG 2: AUSWERTUNG DER STRUKTURIERTEN LEHRERBEFRAGUNG **17**

ANHANG 3: ERGEBNISPROTOKOLL DER ARBEITSKREISSITZUNG **21**

ANHANG 4: AUFSTELLUNG DER AUSGEWERTETEN DOKUMENTE **23**

ANHANG 5: AUSWERTUNG DER EVALUATIONSBÖGEN, TEIL A **24**

ANHANG 6: AUSWERTUNG DER EVALUATIONSBÖGEN TEIL B 37

ANHANG 7: EINSATZ DER MAKROPLANUNG LF 6, TS 4 IM EIGENEN UNTERRICHT...... 42

Abkürzungsverzeichnis

AB……………………………………………………………………….Arbeitsblatt

AK………………………………………………………………………Arbeitskreis

CULIK……………………….Curriculumentwicklungs- und Qualifizierungsnetzwerk – Lernfeldinnovation für Lehrkräfte in Berufsschulfachklasse für Industriekaufleute

EH……………………………………………………………………..Einzelhandel

EQR……………………………………………....Europäischer Qualifikationsrahmen

EvaNet-EH…………Evaluation des Innovationsnetzwerkes Einzelhandels in Hamburg

HIBB…………………………………..….Hamburger Institut für Berufliche Bildung

IBW………… Institut für Berufs- und Wirtschaftspädagogik der Universität Hamburg

KMK………Ständige Konferenz der Kultusminister der Länder in der Bundesrepublik ………………………………………………………………………Deutschland

LerNe-MFA…………………………………………….LernfeldentwicklungsNetzwerk

…………………………………für den Ausbildungsberuf Medizinische Fachangestellte

LF……………………………………………………………………………...Lernfeld

NELE……………………………………Neue Unterrichtsstrukturen und Lernkonzepte ……………………………………………….durch berufliches Lernen in Lernfeldern

SELUBA………………………………Steigerung der Effizienz neuer Lernkonzepte ……………………………….und Unterrichtsmethoden in der dualen Berufsausbildung

SuK……………………………………………………...Sprache und Kommunikation

TS………………………………………………………………………….Teilsequenz

WK…………………………………………………………………. Warenkunde

WuG/WiG…………………………………………………...Wirtschaft und Gesellschaft

ZLV………………………………………………….Ziel- und Leistungsvereinbarung

Abbildungsverzeichnis

Abb. 1: Lernfeld-Kompetenz-Matrix…………………………………………………..4

1 Einleitung

1.1 Stand der Lernfeldcurriculumentwicklung in Forschung und Praxis

Mit der Einführung des Lernfeldkonzepts läutete die KMK 1996 einen Paradigmenwechsel in der beruflichen Bildung ein. Ihre Handreichung für die Entwicklung neuer Rahmenlehrpläne sieht zwei wesentliche Veränderungen vor. Einerseits werden Lerninhalte nicht mehr nach fachsystematischen, sondern nach didaktisch begründeten, beruflichen handlungssystematischen Gesichtspunkten (Lernfelder) geordnet. Andererseits werden Lernziele nun statt in Form detaillierter Inhaltskataloge als für die berufliche Handlungsfähigkeit relevante Kompetenzen formuliert, denen als Wissensbasis fachsystematische Inhalte zugeordnet werden (vgl. TRAMM, 2003, S.6ff.; TRAMM et al., 2009, S.30-32, KMK, 2007, S17f, Riedl, 2004, S.9ff., S.62ff). Mangels Konkretisierung des Kompetenzbegriffs soll hierunter im Rahmen dieser Arbeit der in der Pädagogik auf große Akzeptanz stoßende Kompetenzbegriff Weinerts verwendet werden. Kompetenzen sind demnach „die bei Individuen verfügbaren oder durch sie erlernbaren kognitiven Fähigkeiten und Fertigkeiten, um bestimmte Probleme zu lösen, sowie die damit verbundenen motivationalen, volitionalen und sozialen Bereitschaften und Fähigkeiten [sic], um die Problemlösungen in variablen Situationen erfolgreich und verantwortungsvoll nutzen zu können“ (vgl. LESCH, 2008, S.13f. zit. n. WEINERT, 2001b, S.27f.). Durch die theoriegeleitete Durchdringung beruflicher Handlungssituationen soll die berufliche Handlungskompetenz und Persönlichkeitsentwicklung der Auszubildenden gefördert werden, damit sie den komplexen Anforderungen von Arbeitswelt, Gesellschaft und Privatleben gerecht werden können. (vgl. TRAMM et al., 2009, S.31, RIEDL, 2004, S. 62))

Zur Prozessbeschleunigung sollen Rahmenlehrpläne möglichst unmittelbar auf Landesebene übernommen werden. Die inhaltliche und organisatorische Umsetzung der Rahmenlehrpläne obliegt damit den Bildungsgangkonferenzen der Berufsschulen (vgl. RIEDL, 2004, S. 61, 69f.). Hierbei ist im Sinne einer didaktischen Analyse nach KLAFKI zu klären, welche konkreten Kompetenzen für die Lernenden relevant sind, um berufliche und lebensweltliche Problemstellungen bewältigen zu können und wie diese durch exemplarische Lernsituationen gefördert werden können. (RIEDL S. 62/68/S. 75, LESCH, 2008, S.37-50). Damit stehen die Berufsschulen vor

einer gewaltigen curricularen Entwicklungsarbeit, für die es bei Einführung des Lernfeldkonzepts keine theoretisch und wissenschaftlich begründeten Konzepte gab. Während dies sowohl für manche Lehrkräfte als auch manche Wirtschaftspädagogen ein wesentlicher Grund war, das Lernfeldkonzept grundsätzlich anzuzweifeln, wurde die praktische curriculare Entwicklungsarbeit vielerorts in Projekten wie NELE, SELUBA und CULIK in enger Kooperation mit der Wissenschaft geleistet, so dass heute – 15 Jahre später – theoriegeleitete Lösungsansätze für die Umsetzung des Lernfeldkonzepts bestehen, welche berechtigte Kritikpunkte der Lernfeldgegner aufgreifen. (vgl. KREMER, 2002, 2011, TRAMM, 2009),

1.2 Ziel dieser Arbeit und Vorgehensweise

Diese Arbeit basiert auf der von Prof. Dr. TRAMM am IBW entwickelten Lernfeld-Kompetenz-Matrix, welche in der curricularen Entwicklungsarbeit für Medizinische Fachangestellte (LerNe-MFA), Industrie- (CULIK) und Einzelhandelskaufleute (EvaNet-EH) im Rahmen mehrjähriger Forschungsprojekte entwickelt wurde. Konkret geht diese Arbeit der Frage nach, wie sich die Entwicklungsergebnisse und -impulse des Projektes EvaNet-EH bei einer der teilnehmenden Schulen heute in der Unterrichtspraxis auswirken. Anlass hierfür ist ein Forschungsaufenthalt der Verfasserin an einer der beteiligten Schulen. Dieses bot einen sonst schwer herzustellenden Zugang zum Forschungsfeld. Empirische Untersuchungskriterien sind dabei die Empfehlungen für die curriculare Arbeit gemäß EvaNet-EH-Abschlussbericht vom 11.09.2009. Nachfolgend werden zunächst die der Lernfeld-Kompetenz-Matrix zu Grunde liegende curriculare Strategie sowie die im Rahmen des Projekts entwickelten curricularen Planungsformate vorgestellt. Auf Basis der Empfehlungen des Abschlussberichts werden dann die Fragestellung für die empirische Untersuchung und die hierfür geeigneten Erkundungsmethoden abgeleitet. Abschließend werden die Untersuchungsergebnisse vorgestellt und Konsequenzen für die künftige curriculare Entwicklungsarbeit an beruflichen Schulen sowie die Unterrichtspraxis reflektiert.

2 Das lernfeldbasierte Curriculumentwicklungsprojekt EvaNet-EH und seine heutige Praxis

2.1 Curriculare Entwicklungsprodukte des Projekts

2.1.1 Die Lernfeld-Kompetenz-Matrix als theoriegeleitete curriculare Strategie

Der in der Einleitung dargestellte Übergang vom Fächer- zum Situationsprinzip soll Auszubildenden den Transfer theoretischer Kenntnisse auf die Praxis erleichtern. Das Situationsprinzip als Ordnungsmerkmal birgt aber die Gefahr, dass die fachwissenschaftliche Systematik nicht mehr erkennbar ist und die Lernfelder nicht sinnvoll und logisch im Sinne eines systematischen Bildungsprozesses aneinander anknüpfen. Darüber hinaus besteht ein Problem darin, dass die in den Rahmenlehrplänen genannten Kompetenzziele zu abstrakt und allgemein formuliert sind und sich daher nicht unmittelbar für die operative Unterrichtsplanung eignen. (vgl. TRAMM et al., 2009, S.32-36) Das IBW Hamburg unter Leitung von Prof. TRAMM hat daher in Kooperation mit der Praxis die Lernfeld-Kompetenz-Matrix entwickelt. Die ihr innewohnende curriculare Strategie besteht darin, ergänzend zur vorgegebenen Strukturierung nach Lernfeldern lernfeldübergreifende Kompetenzdimensionen zu definieren und deren systematische Entwicklung über die Lernfelder hinweg zu beschreiben. Diese Kompetenzdimensionen umfassen konkrete, für die berufliche Praxis relevante sach- und persönlichkeitsbezogene Kompetenzen (vgl. ebda. S.37-39). Diese Matrix (vgl. Abb.1) soll in einem formativen evaluativ-konstruktiven Rückkopplungsprozess zwischen Praxis und Wissenschaft weiterentwickelt werden. Hiermit ist die gezielte Abkehr vom bisher üblichen Entwickler-Anwender-Dualismus zwischen Wissenschaft und Behörden einerseits sowie Schulen und Lehrkräften andererseits angestrebt, um die curriculare Akzeptanz durch Einbindung der Lehrenden als maßgebliche Akteur/-innen zwischen Theorie und Praxis zu fördern. (vgl. TRAMM, 1992, S.51, TRAMM et al., 2009, S.10f).

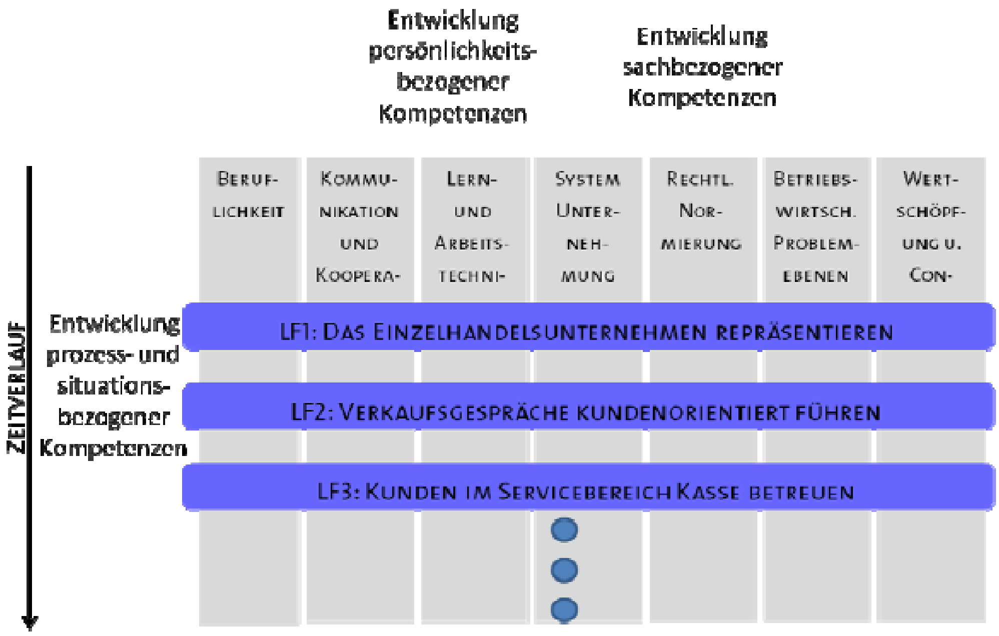

Abb.1: Lernfeld-Kompetenz-Matrix 1

Quelle: Tramm et al., 2009, S. 38

2.1.2 Entwicklungsstand gemäß Abschlussbericht vom 11.09.2009

Das Eva-Net-EH-Projekt war nach Einführung des lernfeldbasierten Rahmenlehrplans 2004 auf Initiative der Schule ins Leben gerufen worden, um die curriculare Entwicklungsarbeit gemeinsam mit den drei weiteren Hamburger Berufsschulen des Einzelhandels und dem IBW zu bewältigen. Die Schule konnte hierbei Erfahrungen aus kleineren Bildungsgängen (z. B. Ausbildung zum/-r Automobilkaufmann/-frau) einbringen. Die dargestellte curriculare Strategie wurde unter Verwendung von Planungsformaten operationalisiert und strukturiert, die sich bereits in den Projekten LerNe-MFA und CULIK bewährt hatten. Zunächst wurde die Bedeutung eines jeden Lernfeldes für den Bildungsgang im Rahmen einer *Curricularen Analyse* beschrieben. Diese enthält die zu vermittelnden Kompetenzen und Wissensbasis, deren Relevanz für berufliche Arbeits- und Geschäftsprozesse sowie deren Einbettung in lernfeldübergreifende Kompetenzdimensionen. Durch eine *Strukturplanung* erhält das Lernfeld eine erste Konkretisierung durch eine Sequenzierung. Erst im Rahmen der *Makroplanung* erfolgt die didaktische Entscheidung über Inhalte und Gestaltung der Teilsequenzen.

Im Rahmen des Eva-Net-EH-Projektes wurden curriculare Analysen und Strukturplanungen in enger Kooperation zwischen IBW und den vier teilnehmenden

Berufsschulen des Einzelhandels entworfen. Diese wurden bei Projektabschluss an allen Schulen für alle Lehrkräfte verbindlich. Die schulübergreifende didaktische Konkretisierung und Erstellung von Unterrichtsmaterial (Makroplanung) sowie deren Erprobung und Evaluation waren die zentrale Weiterentwicklungsaufgabe bei Projektabschluss. Zu diesem Zweck waren die Schulleitungen aufgerufen, geeignete Prozesse zur Verstetigung des Entwicklungsprozesses zu implementieren.

2.2 Erkenntnisleitendes Interesse und operationalisierte Fragestellung

In Vorbereitung auf den Forschungsaufenthalt hatte sich die Verfasserin an Hand der Internetpräsenz und des Abschlussberichtes intensiv in das Projekt eingearbeitet und entwickelte mit diesem Vorverständnis die folgenden operationalisierten Fragestellungen für diese Arbeit:

1. Welche schulinternen und schulübergreifenden organisatorischen Maßnahmen hat die Schulleitung zur Verstetigung des Entwicklungsprozesses ergriffen?
2. Wie wurden die Strukturplanungen für die Unterrichtspraxis als Makroplanung konkretisiert und mit Unterrichtsmaterial angereichert?
3. Welchen Stellenwert haben diese Planungsprodukte für die Lehrkräfte und deren Unterrichtspraxis?
4. Inwieweit werden die curricularen Produkte auf Basis von Unterrichtserfahrungen evaluiert und verbessert?

2.3 Methode der Erkundung

Bei Beginn des Forschungsaufenthalts erhielt die Verfasserin von der Schule wertvolle Hinweise zu Informationsquellen zur Erkundung dieser Fragen. Frage 1 und 2 konnten durch Befragung des Schulleiters und der Lernfeldkoordinatorin sowie Einblick in Arbeitskreisprotokolle (Anhang 3) untersucht werden. Zudem erhielt die Verfasserin auch Zugang zu den derzeitigen curricularen Produkten, die allen Lehrkräften in schriftlicher und digitaler Form zur Verfügung stehen. Der Vergleich dieser Dokumente mit dem Stand bei Projektabschluss ließ ergänzende Rückschlüsse für Frage 2 zu. Der Stellenwert dieser verbindlichen Planungsmaterialien für die Lehrkräfte wurde durch ca. 15-minütige strukturierte Befragungen erkundet (Auswertung s. Anhang 2). Eine sehr aufschlussreiche

Ergänzung lieferten Unterrichtsbeobachtungen im Lernfeld 1 (Abweichungen von der Makroplanung s. Anhang 7). Zur Beantwortung von Frage 4 standen für alle Lernfelder schulinterne und schulübergreifende Evaluationsprotokolle zur Verfügung, die im Wege einer qualitativen Inhaltsanalyse ausgewertet wurden (s. Anhang 4, 5 und 6).

Zur Gewährleistung der Gütekriterien qualitativer Forschung (vgl. Mayring, 2002, S. 144-148) wurde das *Verfahren* im Anhang *dokumentiert* und so *Regelgeleitetheit* sichergestellt. Eine *schlüssige Interpretation* wurde in vielfacher Weise unterstützt: Offenlegung des Vorverständnisses, die *Nähe zum Gegenstand,* eine relativ große Stichprobe (11 Befragte von 24 betroffenen Lehrkräften), eine *kommunikative Validierung* mit der Lernfeldkoordinatorin und Schulleitung sowie eine wechselweise Plausibilisierung durch Heranziehung der unterschiedlichen Methoden im Sinne einer Triangulation. Hierbei war ein essentielles Gütekriterium, sich nicht auf eine „Außensicht" des Unterrichts zu beschränken, sondern „Eingang in die handlungsleitenden Theorien, in die Wahrnehmungs- und Beurteilungsmuster von Lehrern zu finden" (TRAMM, 1992, S.51)

2.4 Ergebnisse der Untersuchung

2.4.1 Organisatorische Vorkehrungen zur Verstetigung des curricularen Entwicklungsprozesses

Die curriculare Weiterentwicklung wurde durch die Schulleitung der Schule konsequent durch ein Bündel schulinterner und schulübergreifender Maßnahmen sichergestellt (Detail siehe Anhang 1). Im Schuljahr 2009/2010 wurde an der Schule ein Arbeitskreis mit der Entwicklung der Makroplanungen für das 1. Ausbildungsjahr beauftragt. Kleine Lehrerteams übernahmen hierbei die Verantwortung für einzelne Teilsequenzen. In schulübergreifenden Treffen wurden parallel dazu die verbindlichen Strukturplanungen und deren Praxistauglichkeit evaluiert. In Vorbereitung hierzu waren die Lehrkräfte der Schule aufgerufen, ihre Erfahrungen einzubringen. Das im Unterricht eingesetzte Material war hierbei von Lehrkraft zu Lehrkraft und Schule zu Schule unterschiedlich und nicht unmittelbar Gegenstand der Evaluation. Im Schuljahr 2010/2011 wurden an der Schule Makroplanungen für das 3. Ausbildungsjahr entworfen und evaluiert, während sich die schulübergreifende Evaluation auf das 2. Ausbildungsjahr bezog. Dieses

Vorgehen an der Schule ist darin begründet, dass die Erarbeitung und Umsetzung auf eine breite personelle Basis gestellt werden sollten. Das o.g. Arbeitskreistreffen war Auftakt für die Entwicklung und Evaluation der Makroplanungen des 2. Ausbildungsjahres im Schuljahr 2011/12. Durch Aufnahme dieser Entwicklungsaufgabe in die Ziel- und Leistungsvereinbarung (ZLV) mit der HIBB-Zentrale wurde die schulische Schwerpunktaufgabe verdeutlicht. Die Lernfeldkoordinatorin stellt sicher, dass der jeweils aktuelle Stand der curricularen Produkte allen Lehrkräften sowohl digital als auch in Papierform zugänglich ist und stellt den schulübergreifenden Austausch sicher. Sie ist auch Ansprechpartnerin für Änderungsvorschläge und Anregungen der Lehrkräfte außerhalb der beschriebenen Prozesse und über das Projektende hinaus. Diese werden bei Bedarf mit der Schulleitung abgestimmt. Nach Projektabschluss haben alle Lehrkräfte, die im Einzelhandel unterrichten, mindestens ein Jahr lang die Entwicklerrolle im Arbeitskreis übernommen und sollen dann (ohne Entlastung) kontinuierlich im curricularen Prozess mitwirken.

2.4.2 Erstellung von Makroplanungen nebst Unterrichtsmaterial

Nach derzeitigem Stand stehen Makroplanungen einschließlich Unterrichtsmaterial vollständig für das erste und dritte Ausbildungsjahr zur Verfügung. Ein Beispiel für eine Makroplanung mit Bezug auf das entsprechende Unterrichtsmaterial liefert Anhang 7 für Teilsequenz 4 im Lernfeld 1. Die entsprechenden Produkte für das 2. Ausbildungsjahr wurden von den Mitgliedern des Arbeitskreises entwickelt. Für die Lernfelder 8 und 11 stehen bereits evaluierte Unterrichtsmaterialien zur Verfügung, da im Rahmen eines separaten Projektes der Übergang zum wirtschaftsinstrumentellen Ansatz im Rechnungswesen umgesetzt wurde.

2.4.3 Umsetzung der Planungskonzepte in der Praxis

Die Lehrkräfte verfügen über sehr heterogene Erfahrungen in Bezug auf Lernfeldunterricht nach dem EvaNet-Konzept. Einige sind Berufsanfänger/-innen und fühlen sich noch vorrangig als Anwendende, andere haben das Lernfeldkonzept und seine Konkretisierung seit 2004 maßgeblich im Projekt mitentwickelt und fast alle Lernfelder nach EvaNet-Format unterrichtet. Bemerkenswert ist, dass inzwischen alle in irgendeiner Form (mindestens im Arbeitskreis) die Entwicklerrolle einnehmen.

Alle Befragten richten sich nach den verbindlichen Strukturplanungen und empfinden dies als zeitsparende Erleichterung für die Unterrichtsvorbereitung. Besonders wird dies von Kolleg/-innen mit geringer Erfahrung oder bei Vorbereitung neuer Lernfelder geschätzt. Das im Lehrertauschverzeichnis digital zur Verfügung gestellte Unterrichtsmaterial wird fast immer gesichtet. Viele Lehrkräfte greifen aber auf selbst entwickeltes, erprobtes Material im umfangreichen Lehrertauschverzeichnis zurück oder passen das Material an ihren speziellen Bedarf an. Die Verfasserin konnte dies anhand eines exemplarischen Unterrichts von Lernfeld 1, Teilsequenz 4 nachvollziehen. In Anhang 7 ist dokumentiert, welche Änderungen in Bezug auf die Makroplanung vorgenommen wurden.

Die Auswirkung auf Unterrichtsdurchführung und Lernziele wird je nach Erfahrungshintergrund unterschiedlich empfunden. Wer bereits vorher handlungsorientierten Unterricht gestaltet hat oder als Berufsanfänger/-in nur das Lernfeldkonzept kennt, empfindet wenig Veränderung. Positiv wahrgenommen werden Qualitätssteigerungen durch die einheitliche Struktur und Zeitersparnis bei der Vorbereitung, der gestiegene Methodenreichtum und eine stärkere Schülerzentrierung. Hinsichtlich der Lernziele wird jedoch kritisch angemerkt, dass der Fokus immer noch stark auf der Wissensbasis liege und der rote Faden durch die gestiegene Komplexität insbesondere für schwächere Schüler/-innen fehle.

Als wesentliche positive Auswirkung des Projekts werden mit großer Mehrheit der Kulturwechsel durch die gemeinsame schulische Entwicklungsarbeit und der schulübergreifende Austausch hervorgehoben. Dies hat die an der Schule bestehende kooperative Tradition unter Lehrkräften eines Bildungsganges weiter gefördert. Fast alle empfinden die gemeinsame Lernfeldarbeit in kleinen Lehrerteams angenehm, wobei die jeweilige Intensität der Kooperation selbst bestimmt wird. Auch die Verbindlichkeit der curricularen Struktur wird nicht als einschränkend, sondern als qualitätsfördernde Basis für gegenseitigen Austausch empfunden.

Hinsichtlich der Schwierigkeiten und Verbesserungspotenziale kristallisieren sich zwei Schwerpunkte heraus: die unzureichende Qualität des Unterrichtsmaterials und die Vernachlässigung der lernfeldübergreifenden Kompetenzdimensionen, wobei eine Wechselwirkung zwischen beiden Aspekten gesehen wird. Bemängelt werden sachliche und formale Fehler, Aktualität, Redundanzen und logische Strukturbrüche im Unterrichtsmaterial. Gewünscht wird ein roter Faden vorzugsweise in Form eines

durchgängigen Musterunternehmens mit aneinander anschließenden Lernsituationen. Benötigt werden auch methodische Bezüge zu den zu fördernden Kompetenzen – idealerweise sollten die Funktionen der Kompetenzentwicklung und -messung integriert sein.

Mehrfach wird zudem hervorgehoben, dass die umfangreiche Wissensbasis und überdimensionierte Kompetenzanforderungen insbesondere für schwächere Schüler/-innen eine vermeidbare Überforderung darstellen. In diesem Zusammenhang ist auch ein möglicher Zielkonflikt zwischen Kompetenzdimensionen und Prüfungsanforderungen auf Grund von zentralen Abschlussprüfungen zu berücksichtigen. Implizit wird hier die fehlende Individualisierungskomponente im EvaNet-Konzept kritisiert, welche im Arbeitskreis durch Begriffe wie „Little Boxes" und „Testeritis" zum Ausdruck kommt. Das Leistungsvermögen und Anforderungsprofil eines Auszubildenden in den Fachrichtungen Elektro und Tankstelle unterscheiden sich z. B. ebenso wie die Eingangsniveaus hinsichtlich der erforderlichen Lern- und Arbeitstechniken bei unterschiedlichem Schulabschluss.

Aber auch die Lehrkräfte fühlen sich durch die Anforderungen der Lernzielmatrix stark gefordert, was im Arbeitskreis als Bedarf nach Fortbildung zur Kompetenzmessung zum Ausdruck kommt. Die Schulleitung hatte die Mängel hinsichtlich des Unterrichtsmaterials bereits im Vorfeld des Treffens erkannt und entsprechende Anforderungen an das zu erstellende Material formuliert (s. Protokoll im Anhang 3). Insbesondere waren Hinweise auf die zu fördernden Kompetenzen und Instrumente zur Kompetenzmessung (Klassenarbeiten, Beobachtungsbögen) gewünscht. Ob die angestrebten Kompetenzen tatsächlich gefördert werden, wurde auch im Rahmen der Evaluationsprozesse untersucht, deren Ergebnisse nachfolgend dargestellt werden.

2.4.4 Evaluativ-konstruktive Weiterentwicklung des Curriculums

Teil A der Evaluationsbögen ging der Frage nach, ob die Teilsequenzen die Inhalte in geeigneter Weise strukturieren und welche Probleme bei der Umsetzung der Strukturplanung aufgetreten sind. Bei der Inhaltsanalyse der Evaluationsbögen (Anhang 5) fielen vier Kategorien von Äußerungen auf: 1. Zeitkontingent, 2. Relevanz der Inhalte und roter Faden, 3. Abgrenzung von Inhalten zu anderen

Lernfeldern, Teilsequenzen oder Fächern sowie 4. Anmerkungen zur Lerngruppe teilweise mit methodischen Hinweisen.

Hinsichtlich des Zeitkontingents fällt auf, dass dieses überwiegend als zu knapp empfunden wird, besonders am Schuljahresende. Beschrieben werden insbesondere Zielkonflikte mit Anforderungen wie Klassenarbeiten, Administration und Prüfungen sowie deren Vorbereitung. Häufig wird in diesem Zusammenhang auch die Komplexität der Themen beklagt.

Die Angemessenheit des Zeitkontingents wird auch dadurch beeinflusst, ob bestimmte Inhalte/Kompetenzen im Lernfeldunterricht oder anderen Fächern angesiedelt werden. Hier stellt sich die Frage, inwieweit rechtliche und gesamtwirtschaftliche Themen in Wirtschaft und Gesellschaft abgedeckt werden bzw. sprachliche und kommunikative Fähigkeiten in Sprache und Kommunikation gefördert werden. Dies zeigte sich z. B. deutlich bei einer Unterrichtshospitation im Lernfeld 1, Teilsequenz 2. Die Auszubildenden zeigten sich irritiert, da sie zeitgleich Präsentationen in Warenkunde, SuK und im Lernfeldunterricht vorbereiten sollten. Auf flankierendes Hintergrundwissen für Teilsequenz 4 konnte dagegen nicht zurückgegriffen werden, da das Fach WuG erst im 2. Ausbildungsjahr unterrichtet wird. Die Abgrenzung zu anderen Lernfeldern und Teilsequenzen nimmt einen sehr großen Raum in der Evaluation ein. Dies lässt auf ein lernfeldübergreifendes Bewusstsein der Lehrkräfte schließen. Für mehrere Lernfelder wurden die Anregungen bereits in geänderten schulübergreifenden Strukturplanungen aufgegriffen. Sehr viel schwieriger gestalten sich die organisatorischen Konsequenzen bei anderen Fächern. Aus diesem Grunde konnte noch keine schulübergreifende, einheitliche Regelung gefunden werden.

Eng verbunden mit diesen Abgrenzungsfragen ist die nach der didaktischen Relevanz von Inhalten. Immer wieder wird gefragt, wie breit und tief bestimmte Themen behandelt werden sollen und ob diese ggf. an einem anderen Ort oder zu einem anderen Zeitpunkt vermittelt werden. Vielfach besteht bei den Lehrkräften zudem Unklarheit, was mit bestimmten Begriffen/Themen gemeint ist, so dass Klärungsbedarf besteht. Diese Fragen müssten sich durch die Konkretisierung in der Makroplanung und dem Unterrichtsmaterial inzwischen geklärt haben. Wie dargestellt wurden diese parallel zur Evaluation erstellt und waren daher noch nicht verfügbar. Es bleibt aber wiederholt der Eindruck einer Überfrachtung mit

Wissensinhalten, die nicht immer den Bedarf der Lerngruppe treffen. Darüber hinaus werden in dieser Kategorie viele Situationen beschrieben, bei denen der rote Faden nicht gewährleistet ist. Dies ist häufig mit der oben dargestellten Zuordnung zu Teilsequenzen und Lernfeldern verbunden, so dass die geänderten Strukturplanungen hier Abhilfe schaffen sollten.

In der Kategorie „Lerngruppe und Methodik" überwiegen wie auch schon in 2.4.3 festgestellt Hinweise auf eine Überforderung schwächerer Schüler/-innen durch die Komplexität und Abstraktheit der Themen und fehlende Basiskompetenzen. Methodische Hinweise sind selten und haben eher den Charakter einer Idee als einer grundsätzlichen didaktischen Strategie. Es entsteht paradoxerweise der Eindruck, dass gerade bei schwächeren Klassen bevorzugt vom Prinzip der Handlungsorientierung abgewichen wird, um Prüfungsanforderungen und Zeitrestriktionen gerecht zu werden.

Teil B der Evaluationsbögen geht der Frage nach, ob die angestrebten Kompetenzen tatsächlich gefördert werden. In der Auswertung (Anhang 6) wurden die in den Interviews und im Arbeitskreis geäußerten Kategorien Kompetenzformulierung, Kompetenzmessung, Umsetzbarkeit und Qualifikation der Lehrkräfte analysiert. Hier zeigt sich erneut, dass die formulierte Kompetenzanforderung vielfach als Überforderung empfunden wird. Zudem wird bemängelt, dass das Abstraktionsniveau der Kompetenzformulierung nicht für eine Messbarkeit geeignet ist. Hinsichtlich der Umsetzbarkeit bestehen Herausforderungen in der fehlenden Beherrschung von Lern- und Arbeitstechniken sowie Zeitknappheit. Seltener scheitert die Kompetenzvermittlung an anderen Aspekten (wie z. B. durch EDV-Probleme), da die Schule auf eine lernfeldgerechte Ausstattung der Lernwerkstätten großen Wert legt. Forderungen nach einer Fortbildung wurden im Evaluationsprozess noch nicht gestellt. Diese hängen offenbar in engem Zusammenhang mit der Anforderung, Instrumente zur Kompetenzmessung als Unterrichtsmaterial zu erstellen.

3 Schlussbetrachtung

Insgesamt fällt bei der Evaluation auf, dass an der Schule strukturelle Themen des Lernfeldunterrichts mit großer Konsequenz einer Lösung zugeführt werden. Hier ist für den evaluativ-konstruktiven Prozess interessant, ob die geänderten

Strukturplanungen Verbesserungen hinsichtlich Zeitkontingent, didaktischer Analyse und innerer Stringenz bringen. Die hierfür erforderlichen Rückmeldungen durch Lehrkräfte dürften organisatorisch auch nach Beendigung des Arbeitskreises mit überschaubarem Aufwand darstellbar sein. Schwieriger gestaltet sich dagegen die geforderte Verbesserung des Unterrichtsmaterials, denn diese wirft vorab grundlegende didaktische Fragen auf:

- Wie können die abstrakten Kompetenzdimensionen in messbare Feinlernziele übertragen werden? (vgl. TRAMM, 2011, S.4-6)
- Welches methodische Vorgehen empfiehlt sich didaktisch und organisatorisch in Hinblick auf die angestrebten Kompetenzen?
- Welche Eingangskompetenzen sind erforderlich? Sind diese auch vorhanden?
- Wie kann der Heterogenität der Lerngruppen Rechnung getragen werden?
- Wie können Prüfungsanforderungen sinnvoll integriert werden, um Zielkonflikte zu vermeiden?
- Wie kann Unterrichtsmaterial so gestaltet werden, dass es sich sowohl zum Fördern als auch zum Messen der Kompetenz eignet?

Die Bewältigung dieser anspruchsvollen und zeitaufwändigen Aufgabe wäre nach Einschätzung vieler Lehrkräfte nur mit einem angemessenen Stundenkontingent oder externen Ressourcen darstellbar. Vorab müssen jedoch die Schulleitungen der Hamburger Einzelhandelsschulen im Kontext der Debatte um den Europäischen Qualifikationsrahmen[1] entscheiden, ob die Kompetenzdimensionen überhaupt in der jetzigen Form beibehalten oder vereinfacht werden. Denkbar wären unterschiedliche Kompetenzniveaus im Sinne einer Pflicht und Kür. Diese sollten mit den künftigen Kompetenzstufen des EQR kompatibel sein (vgl. EUROPÄISCHE KOMMISSION, 2008).

Hiermit wäre bereits eine Basis für eine Individualisierung des Lernens gelegt. Voraussetzung hierfür sind jedoch Instrumente zur Kompetenzdiagnostik und die Transparenz der Kompetenzentwicklung für die Schüler/-innen, wie bereits im

---

[1] Ziel des Europäischen Qualifikationsrahmens (EQR) ist, nationale Qualifikationssysteme auf einen gemeinsamen Referenzrahmen zu beziehen und so die grenzüberschreitende Mobilität von Beschäftigten und Lernenden zu fördern. Details siehe http://ec.europa.eu/education/lifelong-learning-policy/doc44_de.htm

EvaNet-Abschlussbericht als Empfehlung formuliert (vgl. TRAMM et al., 2009 S. 67f).

Ein pragmatisches Vorgehen könnte in der Aushändigung eines Lernpasses (analog der Lernfeld-Kompetenz-Matrix) zur Dokumentation des Lernfortschritts während der Ausbildung liegen. Dieser wäre besonders für schwächere Schüler/-innen ein wertvolles Strukturierungs- und Selbstorganisationswerkzeug. Das im Lehrertauschverzeichnis befindliche Material könnte diesem Kompetenzkatalog zugeordnet werden, um eine transparente Ablagesystematik zu schaffen, und den Lernenden sogar zum selbstgesteuerten Lernen zur Verfügung gestellt werden. Zu Ausbildungsbeginn wären Maßnahmen zur Selbst- und Fremdeinschätzung der Eingangskompetenzen vorzusehen. Die Verfasserin hält die Teilsequenz 2 in Lernfeld 1 für diesen Zweck sehr geeignet, da die Unternehmenspräsentation ohnehin an Hand eines Beobachtungsbogens bewertet wird und in dieser Sequenz die unterschiedlichen Eingangsvoraussetzungen bezüglich der Lern- und Arbeitstechniken sehr deutlich werden. Sinnvoll wäre, den Erwerb dieser Basiskompetenzen am Beginn des Bildungsgangs durch geeignete Angebote sicherzustellen, da bestehende Defizite sonst während des gesamten Bildungsganges die Lernprozesse behindern. Vielleicht können solche Angebote bei Ressourcenknappheit durch Kooperation mit Dritten (z. B. ausbildungsbegleitenden Hilfen) dargestellt werden. Eine andere Möglichkeit stellt die im Rahmen des EvaNet-Projektes schon praktizierte Arbeitsteilung zwischen den Handelsschulen dar.

Die Schule zeichnet sich wie dargestellt durch eine ausgesprochen intensive Kooperation des Lehrerkollegiums aus und liefert mit ihrem herausragenden Abschneiden bei der Schulinspektion einen empirischen Beleg für eine hohe Korrelation von Kooperation und Qualität: „Durchweg scheint es so zu sein, dass in nachweislich guten Schulen das Ausmaß…und..die Art von Kooperation zwischen den Lehrkräften anspruchsvoller ist als in weniger erfolgreichen Schulen (vgl. Roggatz, 2011, zit. n. Terhardt/Klieme, 2006, S.163). Bei Stillstand droht aber Rückschritt. Die Schule wird dieses Qualitätsniveau bei gegebenen Ressourcen nur durch geeignete Kooperationen halten können. Hierdurch steigt die Komplexität. Aber dieses allseits im Berufsleben anzutreffende Phänomen ist der Grund, weshalb

das Lernfeldkonzept das Fächerprinzip abgelöst hat und seiner Natur nach komplex sein muss.

Literaturverzeichnis

EUROPÄISCHE KOMMISSION (2008). Der Europäische Qualifikationsrahmen für lebenslanges Lernen (EQR). Luxemburg: Amt für amtliche Veröffentlichungen der Europäischen Gemeinschaften. Online: http://ec.europa.eu/dgs/education_culture/publ/pdf/eqf/broch_de.pdf

INSTITUT FÜR BERUFS- UND WIRTSCHAFTSPÄDAGOGIK der Universität Hamburg. Internetpräsenz des EvaNet-EH-Projektes. http://www.ibw.uni-hamburg.de/evaneteh/ (04.01.2012)

KLIEME,E.; TERHARD,E.(2006): Kooperation im Lehrerberuf: Forschungsproblem und Gestaltungsaufgabe. In: Zeitschrift für Pädagogik Heft 2/2006, S. 163-166

KMK (2007). Handreichung für die Erarbeitung von Rahmenlehrplänen der Kultusministerkonferenz für den berufsbezogenen Unterricht in der Berufsschule und ihre Abstimmung mit Ausbildungsordnungen des Bundes für anerkannte Ausbiildungsberufe. Online: http://www.kmk.org/fileadmin/veroeffentlichungen_beschluesse/2007/2007_09_01-Handreich-Rlpl-Berufsschule.pdf

KREMER, H.-H. (2002). Lehren mit Lernfeldern – Erfahrungen aus dem Modellversuch NELE. BIBB-Fachtagungskongress 2002. Dokumentation 4

KREMER, H.-H./ TRAMM, T. (2011). Editorial Fachtagung Wirtschaft und Verwaltung: Zwischenbilanz des Lernfeldkonzepts – erfolgreiche Neuorientierung oder Irrweg. In: *bwp@* Spezial 5 – Hochschultage Berufliche Bildung 2011, Fachtagung 19, hrsg. v. KREMER, H.-H./ TRAMM, T., 1-13. Online:http://www.bwpat.de/ht2011/ft19/editorial_ft19-ht2011.pdf (19-11-2011).

LESCH, M. (2008). Die Entwicklung lernfeldübergreifender Kompetenzdimensionen mit Bezug auf KLAFKIs Konzept kategorialer Bildung am Beispiel der Ausbildung von Kaufleuten im Einzelhandel. Unveröffentlichte Diplomarbeit. Universität Hamburg. Online: http://www.ibw.uni-hamburg.de/evaneteh/images/Dokumente/Theorie/lesch%20m.%20entwicklung%20lernfeldbergreifender%20kompetenzdimensionen.pdf

MAYRING, P. (2002). Einführung in die qualitative Sozialforschung. Eine Anleitung zu qualitativem Denken. 5. Aufl. Weinheim: Beltz

RIEDL, A. (2004). Didaktik der beruflichen Bildung. Pädagogik. Stuttgart: Steiner.

ROGGATZ, C. (2011). Unterrichtsentwicklung und Teamarbeit. Ziele, Hindernisse und Gelingensbedingungen. In: Behörde für Schule und Berufsbildung (Hrsg.). Hamburg macht Schule. Heft 1/2011. Hamburg: Pädagogische Beiträge

TRAMM,T. (1992). Konzeption und theoretische Grundlagen einer evaluativ-konstruktiven Curriculumstrategie – Entwurf eines Forschungsprogramms unter der Perspektive des Lernhandelns. Dissertation. Göttingen: Seminar für Wirtschaftspädagogik der Georg-August-Universität Göttingen

TRAMM, T. (2003). Prozess, System und Systematik als Schlüsselkategorien lernfeld-orientierter Curriculumentwicklung. bwpat, Nr. 4, (Mai 2003). Online: http://www.bwpat.de/ausgabe4/tramm_bwpat4.shtml

TRAMM, T. (2009). Vom geduldigen Bohren dicker Bretter - Antworten und Überlegungen eines "beglückten" Kollegen zum Praxisbezug der Wirtschaftspädagogik. bwpat, Profil 2 (Januar 2009). Online: http://www.bwpat.de/profil2/tramm_profil2.shtml

TRAMM, T. (2011). Präzisierung und Taxonomisierung von Lernzielen. Aufsatz für Wirtschaftspädagogikstudenten. Hamburg: IBW, Educommsy

TRAMM, T., HOFMEISTER, W., & DERNER, M. (2009). EvaNet-EH Evaluation des Innovationsnetzwerks Einzelhandel in Hamburg: Abschlussbericht zum 11.9.2009. Hamburg.

WEINERT, F. E. (2001b). Vergleichende Leistungsmessung in Schulen – eine umstrittene Selbstverständlichkeit. In F. E. Weinert (Hrsg.), Leistungsmessung in Schulen (17-31). Weinheim u. a.: Beltz.

Anhang 1: Maßnahmenbündel zur Verstetigung des Projekterfolges

<u>Schulübergreifend</u>

- Kooperationsvertrag der vier Einzelhandelsschulen
- Bestellung von Eva-Net-EH-Verantwortlichen
- schulübergreifende Workshops zur Makroplanung der Lernfelder und zum Austausch von Materialien
- arbeitsteilige Bearbeitung der Lernfelder

<u>Schulintern</u>

- Einrichtung einer A 14 Stelle (Lernfeldkoordinatorin für die Betreuung Lernfelder Einzelhandel/Eva-Net-EH)
- Einrichtung eines schulinternen Arbeitskreises von Lehrkräften eines Ausbildungsjahres mit Entlastung für Konzeptarbeit (abwechselnd 1., 3. und 2. Ausbildungsjahr, damit möglichst viele Lehrkräfte an der Konzeptarbeit und der Umsetzung mitarbeiten können)
- Einrichtung eines digitalen Verzeichnisses (Lehrertauschverzeichnis) im pädagogischen Netz
- Einrichtung von Lernfeldräumen mit Präsentations-, Moderations- und Gruppenausstattung
- schulische Schwerpunktsetzung durch Aufnahme in die Ziel- und Leistungsvereinbarung mit dem HIBB

Anhang 2: Auswertung der strukturierten Lehrerbefragung

(1) Welche Lernfelder haben Sie/hast Du bereits mit Eva-Net-Material unterrichtet?

(1) 1 2 3 4 5 6 7 9 10
(2) 1 2 3 4 5 6
(3) 1
(4) 1 2 3 4 5 6 7 8 9 10 11 12 13 14
(5) 1 2 3 4 5 7 8 9 10 11
(6)
(7) 1 6
(8) 1 2 3 4 5 11 12 13 14
(9) 1 4 5 11
(10) 1 2 3 6 8 10 12 14
(10)1 2 3 4 5 8 11
(11)1 2 3 4 5

(2) An welchen Projektveranstaltungen/Maßnahmen/Arbeitskreisen in Zusammenhang mit Eva-Net waren Sie/warst Du beteiligt?

(1) Koordinatoren-Treff, schulübergreifende Workshops, Lehrer-Fragebogen, Planungsformate erarbeitet im Projekt
(2) Nebenprojekt Kompetenzmessung, Abschlussmeeting
(3) AK 1. Ausbildungsjahr
(4) Mitwirkung am Projekt, AK 1. Ausbildungsjahr
(5) Mitwirkung am Projekt, AK 1. Ausbildungsjahr, schulübergreifende Treffen 2. Ausbildungsjahr
(6) Schulübergreifende Evaluation, AK 3. Ausbildungsjahr
(7) AK 1. Ausbildungsjahr
(8) AK 1./2. Ausbildungsjahr
(9) Schulübergreifende Treffen, AK 1. Ausbildungsjahr, LF 2
(10) AK LF 8 und LF 11
(11) Kompetenzteam Projekt, LF 8 und 11, AK Kompetenzmessung

(3) Die Strukturplanungen des EvaNet-Projekts sind seit dem Schuljahr 2009/2010 verbindlich. Welche konkreten Veränderungen hat dies für Sie/Dich mit sich gebracht für…
(4) Welchen Nutzen bringen die zusätzlich erstellten Makroplanungen und das zur Verfügung gestellte Unterrichtsmaterial für…?

a) …Unterrichtsvorbereitung

(1) Orientierung an Sequenzierung /Planungsformat, nutze eher eigenes Material
(2) Erleichterung, Struktur, Kompetenzen, Teilsequenzen
(3) Inhalte und Kompetenzen laut Planungsformat, alle Materialien in einem Ordner
(4) Teilweise schneller, Anpassungen trotzdem erforderlich

(5) Immer Material da, sichten was gefällt, dann Lehrertauschverzeichnis, ggf. anpassen
(6) Auch mal spontan möglich, Arbeitsmaterialien immer vorhanden – auch für unterschiedliches Lerntempo
(7) Zeitlich minimiert, da didaktische Reduktion schon erfolgt
(8) Erleichterung bei neuen Lernfeldern, Leitfaden für Unterrichtsplanung, nicht immer das Material genutzt
(9) Totale Umstellung, Sequenzen vorstrukturiert, man kann auf vorbereitete Unterlagen zurückgreifen
(10)Material sichten, Strukturplanung hilft für Makroplanung als Leitfaden, besonders bei neuen Lernfeldern, weniger aufwändig, mehr Struktur
(11)Aufwand ist geringer geworden, da Material zur Verfügung steht, Strukturplanung hilft dabei

b) ….Unterrichtsdurchführung

(1) Keine Änderung (eigenes Material)
(2) Besserer Unterricht durch bessere Vorbereitung
(3) Unterrichtsmaterial mit kleinen Änderungen
(4) Weniger Lehrervortrag, mehr Ergebnissicherung
(5) Verbindlicher Lehrplan, alle halten sich daran
(6) Bessere Teamabsprache
(7) Methodenreicher, neue Ideen – ein Jahr konsequent nach Eva-Net unterrichtet
(8) Konsequente Umsetzung nicht möglich, wg. Auslagerung WuG, uneinheitlich an allen Schulen, sonst keine Änderungen
(9) Veränderte Schwerpunkte, s. Lernziele
(10)Material wird teilweise genutzt als Arbeitsblatt für Stationenlernen, Kompetenzformulierung wird für Lernzielabgleich genutzt, sonst wenig Änderungen
(11) Materialien ermöglichen schülerzentrierten Unterricht

c) ….Erreichung der Lernziele?

(1) Keine Änderung
(2) Immer noch starke Orientierung an Wissensbasis, weniger an Kompetenzen
(3) Keine Änderung
(4) Roter Faden für Lernende nicht erkennbar, Eingangsvoraussetzungen für bestimmte Klassen zu hoch, haben selbstständiges Arbeiten nicht gelernt
(5) Text von KMK zu wenig konkret, Sicherheit, Qualitätssicherung für Schule
(6) Lernziele nicht immer erreichbar, bei schwachen Schüler/-innen Vertiefung mangels Zeit nicht möglich
(7) Hat nicht alles gut geklappt, mehr Kompetenzen, didaktische Reduktion überwiegend erreicht
(8) Keine Änderungen
(9) Lernziele neu formuliert, da veränderte Schwerpunkte
(10)Induktives Lernen auf Anwendungsebene (hat sich nicht geändert), kein fachsystematischer Unterricht
(11)Erreichung der Kompetenzen (messbar?)

(5) Was hat sich durch Eva-Net im positiven Sinne verändert?

(1) Auseinandersetzung mit Curriculum
(2) Kulturwechsel angestoßen für Schulentwicklung
(3) Gemeinsame Curriculumentwicklung
(4) Doppelbesetzung mit Lehrerteams
(5) Verbindlichkeit, Hilfestellung für Anfänger (sind auf der sicheren Seite) Einheitlichkeit, schulübergreifende Abstimmung gut bei Schulwechsel
(6) Kritisch-konstruktiver Austausch innerhalb des Kollegiums
(7) Arbeitserleichterung, Methodenvielfalt
(8) Für neue Kollegen sehr hilfreich, Abstimmung zwischen Schulen ist bei Schulwechsel vorteilhaft
(9) Engere Zusammenarbeit mit Kollegen
(10)Auch bei viel Stress roter Faden durch Strukturplanung/Materialsammlung, Einheitlichkeit, Klassenwechsel einfacher
(11)Inhalte und Struktur weitgehend vereinheitlicht

(6) Welche Schwierigkeiten bringt Eva-Net mit sich?

(2) Fülle des Materials überwältigend für neue Mitarbeiter, (Papier-)Ordner überflüssig
(3)
(4) Lernfelder nicht gesteuert/zu offen, Unterrichtsmaterial für Lernfeld 12 fast identisch mit Lernfeld 5, Redundanzen
(5) Keine
(6) Absprache mit anderen Einzelhandelsschulen (kein Austausch), Material teilweise fehlerhaft
(7) Prüfungsvorbereitung separat (nicht integriert)
(8) Vermittlung von Kompetenzen sicherzustellen ist zusätzlich nicht praktikabel, manche Kollegen halten sich nicht an das Eva-Net-Konzept
(9) die einzelnen Kompetenzen zu messen
(10)Material aktuell halten (Zuständigkeit), Zurückgreifen auf vorangegangene Lernfelder
(11)Da das entworfene Material nicht verbindlich ist, kann Unterricht zum Selben Lernfeld sehr verschieden sein, Problem beim Zusammenlegen von Klassen

(7) Was gilt es noch zu verbessern und wie ist dies zielführend zu erreichen?

(1) Blick auf Kompetenzen, Unterrichtsmaterial fehlerfrei/aktuell halten
(2) Lernziele kaum zu bewältigen, Noten in Fächern nicht nachvollziehbar, eventuell Neuordnung mit Modularisierung der Berufsausbildung auf EU-Ebene?
(3)Verschiedene Kompetenzen ansprechen/ immer wieder fördern(lassen sich nicht vermitteln wie Qualifikationen), lernfeldübergreifende Perspektive, Kompetenzdimensionen zu theoretisch
(4) Materialien überarbeiten (Hinweis, welchen Kompetenzen angesprochen werden, auf Arbeitsblättern) - Lernsituationen an einem Modellunternehmen anknüpfen
(5) Teilsequenzen teilweise drehen, z.B. bei Lernfeld 3, zu wenig Orientierung an Kompetenzen, für Unterrichtspraxis zu theoretisch, gute Idee, aber zu trocken, nicht schematisch möglich, thematische Vermittlung steht im

Mittelpunkt (teilweise Inhalte zu aufgebläht, Entzerrung/Abspeckung war ursprüngliches Ziel)

(6) Material evaluieren und verbessern, auch eigenes Material einsetzen, Kompetenzdimensionen für schwache Schüler/-innen zu anspruchsvoll (können noch nicht selbstständig arbeiten)

(7) Aktualisierung/Verbesserung des Materials sicherstellen (Verantwortlichkeit)

(8) Kompetenzorientierte, fehlerfreie Materialien (Ablagelogik), es wurde nur die Strukturplanung evaluiert, nicht das Material, ein durchgängiges Beispielunternehmen, Kompetenzen zu aufgebläht (wissenschaftlich)

(9) Verwendung einer durchgängigen Eingangssituation als „roter Faden"/einheitliche Struktur (bei Wechsel der Teams innerhalb des Lernfeldes wichtig)

(10) Nicht geeignet für alle Lerngruppen/Situationen, Eva-Net deckt unterschiedliche Bedarfe nicht ab

(11) ständige Überarbeitung/Aktualisierung/Verbesserung der Materialien, stärkere Berücksichtigung der Kompetenzen: Welche Kompetenzen werden durch die eingesetzten Materialien gefördert? Es fehlen Instrumente zur Kompetenzmessung. Zu Beginn eines Lernfeldes müssten den Azubis die Kompetenzen genannt werden, die sie in diesem Lernfeld erreichen können. Anschließend brauchen die Schüler/-innen ein Feedback über die Erreichung der Kompetenzen. Dies scheitert zurzeit an der fehlenden Prüfmöglichkeit der Kompetenzen.

Anhang 3: Ergebnisprotokoll der Arbeitskreissitzung

Hinweis: Äußerungen wurden den Fragen der strukturierten Befragung zugeordnet. Hierbei ergab sich der Natur eines Arbeitskreises entsprechend ein Schwerpunkt auf Frage 5 und 6 (Schwierigkeiten und Verbesserungspotenzial)

Äußerungen der Lernfeldkoordinatorin Teilnehmerbeiträge

1. **An welchen Projektveranstaltungen/Maßnahmen/Arbeitskreisen in Zusammenhang mit Eva-Net waren Sie/warst Du beteiligt?**

16 geladene Teilnehmer, davon 12 anwesend, zuzüglich der moderierenden Lernfeldkoordinatorin, 2 Teilnehmer nehmen zum ersten Mal am Arbeitskreis teil, die übrigen haben bereits einmal an einem der vorherigen Arbeitskreise teilgenommen.

5. **Welche Schwierigkeiten bringt Eva-Net mit sich?**

- In Praxis schwierig, Unterrichtsmaterial immer selbst zu erproben
- Durch Arbeitsteiligkeit (Zuständigkeit einzelner Lehrerteams für einzelne Teilsequenzen) sind Sequenzen nicht immer schlüssig verbunden

- Lehrerteams haben unterschiedliche Arbeitsstile

- Kompetenzen stehen noch nicht ausreichend im Fokus des Materials

- Die vorhandenen Arbeitsblätter orientieren sich nicht an den zu fördernden Kompetenzen und enthalten diesbezüglich keine (didaktischen) Hinweise. Dies wurde in der bisherigen Arbeit des Arbeitskreises aus den Augen verloren.

- Es fehlt der erforderliche Sachverstand für die Kompetenzmessung. Hierfür ist externe Hilfe erforderlich. Erste Lösungsansätze in Kooperation mit dem IBW (Prof. Tramm) sind gescheitert. Am Lehrstuhl von Prof. Dr. Klaus Breuer an der Universität Mainz soll es Lösungsansätze für handlungsorientierte Abschluss-prüfungen zum Versicherungskaufmann geben.

- Kompetenzen sind zu formalistisch und zu vage beschrieben, um diese messen zu können

- Kompetenzen müssten neu formuliert werden. Ist es wirklich Ziel, dass alle Little Boxes sind, die aus dem Bildungsgang alle gleich herauskommen?

- Aufgabe von Schule ist es eher, Angebote zu machen, die es ermöglichen, Kompetenzen zu entwickeln

- Es besteht die Gefahr einer „Testeritis“ zur Qualitätsmessung von Schulen. Feedback von Lernenden zeigt, dass sie in Klassenarbeiten (Ankreuzaufgaben) nicht zeigen können, was sie gelernt haben. Ein Rückschritt zur Handlungssituation im offenen Unterricht (z.B. Lernbüro) wäre sinnvoller.

- Es besteht bei den Lehrkräften Fortbildungsbedarf für Kompetenzfeststellung und -formulierung. Sie haben nicht die Ausbildung, um Kompetenzmess-instrumente zu entwickeln.

- Es bestehen Brüche im System. Die Prüfungsanforderungen passen nicht zu den Kompetenzzielen.

6. Was gilt es noch zu verbessern und wie ist dies zielführend zu erreichen?

im Rahmen des Arbeitskreises:

- Unterlegung der Strukturplanungen des 2. Ausbildungsjahres (Lernfeld 6-10) mit Unterrichtsmaterial (einschließlich Lösungen/Erwartungshorizont), Hinweis auf zu fördernde Kompetenzen
- Zusätzlich Instrumente zur Kompetenzmessung (Klassenarbeiten, Fragebögen, Beobachtungsbögen für Rollenspiele)
- Grobe Makroplanung
- vorherige Erprobung des Unterrichtsmaterials im eigenen Unterricht
- Idealvorstellung: ein Unternehmen für Lernsituationen eines Lernfeldes
- Einheitliche formale Anforderungen an Layout (keine Jahreszahlenverwenden)
- Präsentation der eigenen Unterrichtserfahrungen (mit Fokus auf Kompetenzförderung) im Arbeitskreis -> Teilnahme offen für alle interessierten Lehrkräfte

laufend:

- bei Rechtschreibfehlern Kopie des Arbeitsblattes an Ersteller zwecks Verbesserung
- Paten für Lernfeldordner (zuständig für Aktualisierung)

- Warum werden nicht schulübergreifende Synergieeffekte genutzt? An den anderen Einzelhandelsschulen wurde das Material für das 2. Ausbildungsjahr schon entworfen. Warum kann darauf nicht zurückgegriffen werden?

- Warum sind die Schulen so zurückhaltend im Austausch von Material? Das Material für Lernfeld 8 und 11 (wirtschaftsinstrumentelles Rechnungswesen) könnte im Austausch angeboten werden. Die anderen Schulen sollen auch über Material für individualisierten Unterricht verfügen.

- Im Lehrertauschverzeichnis ist bereits viel Material vorhanden, das 1:1 verwendet werden kann.

Anhang 4: Aufstellung der ausgewerteten Dokumente

Aufstellung der ausgewerteten Eva-Net-Dokumente

Die fett hervorgehobenen Dokumente wurden in der Auswertung unmittelbar berücksichtigt.

Da sich die Forschungsfrage auf die H11 bezieht, wurden vorrangig schulinterne Dokumente ausgewertet und nur bei Fehlanzeige auf schulübergreifende Dokumente zurückgegriffen.

Nicht ausgewertete Dokumente wurden zur Plausibilitätsprüfung/Interpretation im Zuge einer Triangualation hinzugezogen.

	Evaluationsbogen H11	schulübergreifender Evaluationsbogen	
	**19. Februar 2009**	29. April 2010	**Arbeitskreisprotokolle H11**
Lernfeld 1	**26. April 2009**	29. April 2010	02.12.2009
Lernfeld 2	**22. Februar 2010**	29. April 2010	26.04.2010
Lernfeld 3	**5. Juli 2010**		22.02.2010
Lernfeld 4	**5. Juli 2010**		05.07.2010
Lernfeld 5		**ohne Datum****	05.07.2010
Lernfeld 6		**ohne Datum****	
Lernfeld 7	**ohne Datum***	ohne Datum	
Lernfeld 8		**ohne Datum****	
Lernfeld 9		**ohne Datum****	
Lernfeld 10	**ohne Datum***		
Lernfeld 11	**8. Mai 2011**		
Lernfeld 12	**20. Juni 2011**		
Lernfeld 13	**20. Juni 2011**		
Lernfeld 14			

* wirtschaftsinstrumenteller Ansatz

** die schulübergreifenden Evaluationen wurden ersatzweise herangezogen, da eine Evaluation für das 2. Ausbildungsjahr an der H11 noch nicht stattgefunden hat. Die Äußerungen der anderen Schulen wurden in der Auswertung kursiv kenntlich gemacht.

Anhang 5: Auswertung der Evaluationsbögen, Teil A

Das Einzelhandelsunternehmen repräsentieren

	Zu geringes/zu großes Zeitkontingent	Relevanter Inhalt und roter Faden/innere Logik	Abgrenzung zu anderen Lernfeldern/Fächern/TS	Anpassung an Lerngruppe/ methodische Hinweise
LF 1 TS 1	..Orientierung erfordert mehr Zeit			…einfachere Kriterien (für Betriebserkundung vorgeben)…
TS 2	..Zeit ist zu knapp für…<br><br>..so dass dort viele Wiederholungen erforderlich waren..	..sehr abrupter Übergang von der Praxis (Unternehmenspräsentation) zur Theorie (Wirtschaftskreislauf)..<br>..unklar ist, wie weit man in die Tiefe gehen soll (Wissensbasis hat zu viele Stichworte)…<br>..konnte zu diesem Zeitpunkt nicht vertiefend behandelt werden..<br>..Erarbeitung der Betriebs- und Verkaufsformen im Rahmen der Präsentation nicht sinnvoll..	… die Unterrichtsinhalte, die vorher in WiG ausgelagert waren<br><br>Betriebsformen, Verkaufsformen, Sortiment, Aufgaben …des EH sind eigene Themen, die nicht nur im Zuge der Betriebspräsentation auftauchen dürfen.	S bringen Sortiments-begriffe schnell durcheinander..<br>S haben Schwierigkeiten mit ..Charakterisierung der Betriebsformen..
TS3	Zeit ist zu knapp für…	…klarer Zusammenhang fehlt..<br>Sehr abrupter Übergang von der Praxis…zur Theorie..<br>unklar ist, wie weit man in die Tiefe gehen soll (Wissensbasis hat zu viele Stichworte)…<br>…es fehlen teilweise Übergänge bzw. Verknüpfungen zu den sehr umfangreichen, theoretischen Themen untereinander..	…die Unterrichtsinhalte, die vorher in WiG ausgelagert waren<br><br>…welche Themen sollen explizit in WuG ausgelagert bzw. dort vertieft werden?—	…z.T. zu abstrakt für die S<br><br>Anpassung der Formulierungen und Zielsetzungen an die unterschiedlichen Lerngruppen hinsichtlich der

TS4		..sollte früher unterrichtet werden, da sich aus dem Berufseinstieg Fragen zum Ausbildungsvertrag ergeben… …Rahmen schaffen für das später folgende LF 2..<br><br>..wie tief, nicht überschaubar…		Niveaus und des Ausbildungsziels (Verkäufer – Kaufmann im EH<br><br>.., die eine Einführung..in Hinblick auf die Lebenssituation der S erforderlich macht.
TS5	…zu viele Themen – Zeitproblem..<br>…Zeitvorgabe ..zu knapp .. 10 Std. sind nicht ausreichend für die ..umfangreichen..Themen		Kündigungsschutz: Abgrenzung zu LF 13 unklar, nicht unterrichtet (-> WuG?)<br>Kündigungsschutz sollte in TS 3 an Ausbildungsvertrag anschließen…<br>..Kündigung sollte auf Ausbildungsverträge beschränkt werden, ansonsten gehört die K. in LF 13<br>..Arbeitszeiten gehören in TS 4..<br>..Anforderungen an das Verkaufspersonal..und Berufsbild sind hier fehl am Platz – entweder TS 1 oder LF 2 oder LF 13	..für die S schwer zu durchdringende Themen..
TS6			Sozialversicherung: Abgrenzung zu LF 13 unklar, Hinweise auf Anteile in WuG geben..	
TS7	..nicht geschafft…<br>..ausgelassen, weil zu wenig Zeit vorhanden..	Konglomerat von Themen, deren	..Unklarheit bezüglich. zukünftiger WuG-Inhalte<br>..kommt noch einmal in LF 7 vor…<br>..Hinweise auf Anteile in WuG geben..	

Verkaufsgespräche kundenorientiert führen

Alle TS		Zusammenhang nicht immer erkennbar ist..		…und die S verwirrt..
LF 2 TS 2	..zeitliche Rahmen zu umfangreich..<br>… zu viel, zu lang..		Inhalte..,die in S+K vermittelt werden, müssen herausgenommen werden..<br>Kundentypen gehören in TS 5	
TS3	…zu kurz bemessen..	…LAT passt inhaltlich nicht, da S erst in TS4 lernen, wie sie Informationen über Ware beschaffen..<br>…Kaufmotive sollten bereits in der TS3 unterrichtet werden..<br>…wozu TS 5 ?..	..gehört nicht in die Bedarfsermittlung, sondern in TS 5..<br>…Instrumente zur Kundenbefragung eher in Marketing einzuordnen..	
TS4		…wie breit und tief soll Thema behandelt werden?...<br>Reduktion nötig hinsichtlich Breite/Tiefe	..nicht behandelt, da die S Warenkunde haben,.. Abstimmungsbedarf<br>..Markenzeichen, Trends auch im LF 7<br>…Überschneidung mit Warenkunde..<br>..Abstimmungsbedarf mit WK-Lehrern hinsichtlich Tiefe/Art der Waren..<br>..wird z.T. auch.. im späteren LF (Lager) gemacht..<br>..Abstimmungsbedarf hinsichtlich Körpersprache mit SuK	
TS5		…Verkaufsargumente ohne Warenvorlage schwierig…		
TS6		…Warenvorlage gehört vor TS5 und sollte mit Bedarfsermittlung TS3		

Kunden im Servicebereich Kasse betreuen

TS9	..14 Std. zu viel…	verbunden werden..<br>…die Preisnennung sollte mit der Verkaufsargumentation (TS5) verbunden werden..		
LF 3 TS 2	…kann aus zeitlichen Gründen nur sehr kurz ansprochen werden..<br>…Ergonomie rausgelassen/am Rande besprochen, da sonst zeitliche Probleme..	…	…Gesundheitsschutz sollte in WuG verlegt werden..<br>..Verhaltensregeln und Kassieranweisungen (TS3) zusammengefasst..	..ergonomische Anforderung schwierig für Tankstellenklassen
TS3		…es wäre sinnvoll, die rechtlichen Aspekte (TS6) an den Anfang zu stellen..<br>..Inhalte aus LF2 können noch nicht vorausgesetzt werden..(wg. Tausch LF2 und LF3)	EAN-Code erst in TS7, Warenwirtschaftssystem angesprochen ->Thema nach LF6/TS6 verlagern	
TS4	…zu Gunsten TS5+6 gekürzt..		…Überschneidung mit Warenkunde, Abgrenzung zu LF8 und 11 fehlt..<br>..Rabatte: Einkaufs- und Verkaufs-kalkulation wird erst im LF9 unterrichtet..	..18 Std. sinnvoll hin-sichtlich der Rechen-fähigkeiten einiger S
TS5	S benötigen mehr Zeit als vorgesehen…	…es wäre sinnvoll, die rechtlichen Aspekte (TS6) an den Anfang zu stellen..	..Geld- und Güterströme ..gehört in ein späteres LF..	…selbstständige Erarbeitung zeitaufwändig, aber

	TS6	..20 Std. zu wenig in schwacher Verkäufer-klasse..<br>S benötigen mehr Zeit als vorgesehen	..Passage streichen, da LF2 zeitlich hinter LF3 unterrichtet wird..<br>..Sequenzen getauscht..		erfolgreich..<br><br>..Inhalte sehr vielfältig und für viele S zu komplex…
	TS7		Abfolge der TS ist für schwächere S nicht ganz nachvollziehbar. TS 4+6 haben keinen praktischen Bezug zur Kasse.<br>..TS6 + 7 sollten getauscht werden..		
	Alle TS	…vorhandene Zeit für LF2-5 zu knapp..	LF 3 in anderer, sinnvollerer Reihenfolge unterrichtet..<br>..Zusatzverkäufe, Serviceleistungen geht nicht, da LF2 und LF3 getauscht..		
Waren präsentieren	LF 4 TS 1	2 Std. sind zu lang..	..Sequenzierung zu detailliert..<br>..Ansprechen vieler Sinne zu früh..		
	TS2		…besser wäre eine Fokussierung auf rechtliche Hintergründe in TS4..	..Kriterien zu Unfallvermeidung: wurde bereits in LF1 behandelt	
	TS3	..12 Std. zu umfangreich..<br>..Fokus lag aus zeitlichen Gründen stark auf prüfungsrelevanten Aspekten..	..Preisauszeichnung..Voraussetzung. Diese wird erst in TS 4 behandelt..<br>..unklar, was ethische Aspekte bedeuten..	Unfallvermeidung und Sicherheits-bestimmungen -->WuG bzw. späteres LF	
	TS4		TS 3+4 in Workshop zusammen-gefasst	Ethische Aspekte redundant, besser am Ende von TS5..	

	TS5			Eine weitere Einheit TS5 ist eigentlich nicht nötig -> streichen	
Werben und den Verkauf fördern	LF 5<br>TS4	..4 Std. sind zu knapp..			
	TS6			Überschneidung mit Werbegrundsatz „soziale Verantwortung", Kombination mit TS3?	
	TS7			Thematische Überschneidung mit LF1, TS ist „herangehängt" -> eher WuG?	
	Alle TS	..leider zu wenig Zeit für dieses LF am Ende des Schuljahres..<br>Zeitrichtwert ok, stand aber nicht zur Verfügung			Tempo teilweise sehr/zu schnell..
Waren beschaffen	*LF 6*<br>*TS2*			*..statt in TS3 schon hier…Einkaufs-kooperationen unterrichten..*	
	TS3		*Beispiele aus der eigenen Branche gibt es nicht..*<br><br>*Verteilungsrechnen mit aufnehmen*		
	TS4				
	TS5			*Anfragen/Bestellungen formulieren besser in SuK*	
	TS6			*Wiederholungssequenz nach TS 5..* optional begleitend zu TS2-5	

	TS7			*ausgelassen*	
Waren annehmen, lagern und pflegen	*LF 7*			Lagerbuchhaltung nicht behandelt	
	TS2		*Wissensbasis überfrachtet*	*Integriert in TS1/2*	
	TS5			Unfallgefahren-> WuG	
	TS6		Lagerzinssatz nicht im Lehrplan enthalten	*Lagerkennzahlen in LF6 integrieren*	TS eignet sich für selbstständiges individualisiertes Arbeiten
	TS7			An den Anfang von LF8 setzen (wirtschaftsinstrumenteller Ansatz)	
Geschäftsprozesse erfassen und kontrollieren	LF 8 TS 1	.. ggf. kürzen, da mit 6 USt. zu umfangreich..			
	TS 2.3				TS wurde ausge-schlossen (Tankklasse)
	TS 2.4			….bereits in TS 2 integriert..	
	TS 3			…Interpretation der Bilanz kann in TS4 verschoben werden..	
	Alle TS	..Verlauf sehr engmaschig..	TS7 (Inventur) des LF7 gibt beim wirtschaftsinstrumentellen Ansatz keinen Sinn..	..Auswertung der GuV in LF11?<br>Tausch der TS2 und TS3 beim wirtschafts--instrumentellen Ansatz	..kein Spielraum für eigene Ideen..
Preispolitische Maßnahmen vorbereiten und durchführen	*LF 9 TS2*	..vor der Prüfung keine Zeit, Excel-Kalkulation durchzuführen…	*..detailliertere Beschreibung der Handlungskosten/Grenzen der Verwendbarkeit von Kennzahlen..*<br>*..wie ausführlich/differenziert..*		

			Umsatzermittlung wird nicht erwähnt..		
	TS3		*Informationsbeschaffung zu Einflussgrößen der Preisfestlegung – Wie?*		Preisbildungsfaktoren zu theoretisch
	TS4		*Handelsspanne mit einbeziehen*<br>*Differenzkalkulation/Mischkalkulation*<br>*Umsatzsteuer*		
Besondere Verkaufssituationen bewältigen	*LF 10*<br>*TS1*	*Nicht alle TS vor der Prüfung geschafft*			*Rollenspiele – klassenindividuelle Entscheidung*
	TS2		*Ggf. splitten 1. Kundentypen 2.Verhalten in bsd. Situationen*	*Wdh./Vertiefung LF 2 und 7*	
	TS3		*Produkthaftung fehlt*	*Wdh./Vertiefung LF2 und 7*	
	TS4	*Ggf. TS2 z.G. TS4 kürzen*<br>*Zinsrechnung braucht sehr viel Zeit*<br>*..nur angerissen…*	*Teilzahlungskäufe fügen sich nicht logisch ein.*<br>*Berechnung sehr kompliziert..*<br>*Klären, welche Formel die S können müssen..*	*Nach LF3 verschieben*<br>*Könnte auch mit TS 5 getauscht werden*	*..da diese TS in einigen Klassen sehr tief vermittelt werden muss*
	TS5		Unterstützung durch EH-Unternehmen – was ist gemeint?	*Warum LF7/8 genannt und andere LF-Rückgriffe nicht?*	*Ggf. Polizeibeamten in die Klasse holen*
Geschäftsprozesse erfolgsorientiert steuern	LF 11<br>TS2	..Zeitrichtwert zu knapp<br>..in ET-Klasse	..unklar bei Wareneinsatz, Bedeutung Rohgewinn?	Wareneinkauf und –verkauf werden in LF8 behandelt	..Schwierigkeiten mit den Bestands-veränderungen..
	TS3		..nach Behandlung des Rohgewinns	Afa zum Einstieg nicht geeignet ..aber	..schwierig sind die

	TS4		ist Ermittlung des Gewinns sinnvoller als Afa<br><br>..S sollten Abgrenzung von Kosten/Leistungen und Aufwand/Ertrag nicht nur „optional" lernen	wohin?<br>TS 3 und TS4 tauschen	ständigen Veränderungen bei den eingesetzten Verfahren..
	TS5		..Unterschied zwischen Vollkosten- und Deckungsbeitragsrechnung deutlich machen…		…sonst haben S Verständnisprobleme
	TS6		..Kostensenkungsmaßnahmen-welche?	..ggf. nicht als separate TS..	
	Alle TS		Durch die Einführung des wirtschaftsinstrumentellen Ansatzes sollte die Strukturplanung, was LF8 und 11 betrifft, zunächst angepasst werden..		
Mit Marketingkonzepten Kunden gewinnen und binden	LF 12<br>TS1		..Einstieg erst in TS3 (zunächst Marktforschung in TS2)<br><br>..zur Orientierung wäre ein informierende Unterrichtseinstieg sinnvoller…	..ggf. Marketinginstrumente noch nicht ansprechen..<br>..Marketingziele und –instrumente sollten in TS2 wie in TS7 vorgesehen .. an einem komplexen Beispiel erarbeitet werden..<br>..Vertiefung dann in der TS3..	…da zu erklärungs-bedürftig..<br>..ohne konkrete Ausgangssituation fehlt die Handlungs-orientierung
	TS2	Zeithorizont erschien zu knapp..<br>Zeitverlust durch GA+Präsentationen..		..gemeinsam mit LF14 durchführen<br>..das führt zur Wissensanwendung aus LF3	..recht abstrakte Begriffe<br>Unterrichtsidee, einen Fragebogen zu

				entwickeln
TS3	Zeitumfang von 20 Std. angebracht, da Prüfungs-vorbereitung bevorsteht, für diese TS 14 Std. eingeplant.. ..Präsentation und Auswertung…bedarf eines hohen Zeitaufwandes..			Es stellt sich die Frage des Methoden-einsatzes bei diesem Zeitkontingent..nur in schnell arbeitenden, leistungsstarken Lerngruppen umsetz-bar... für leistungs-schwächere Schüler-gruppen bietet sich eher eine arbeitstei-lige Gruppenarbeit an.
TS4		Warum sollen S Webseite erarbeiten?...rechtliche Regelungen..für das E-Commers..besonders relevant… …relevanter Zusammenhang zwischen Internetmarketing und Marketingmix	TS7 nimmt diese Ideen auf..	.. haben Vorwissen, an die angeknüpft werden kann.. …können die Schüler eigene Erfahrungen einbringen..
TS5			TS5 und TS 6 tauschen	..bietet besonders für leistungsschwächere S die Möglichkeit, Erfahrungen aus ihrem Betrieb einzubringen…
TS6		..stellt sich die Frage, …wie S ..Marketingkonzepte…selbst		.. für leistungs-schwächere S ist es

			bewerten sollen.		überaus schwierig, den Erfolg der Marketing-instrumente … zu analysieren
	TS7		..warum Marketingkonzepte ..in verschiedenen Marktumfeldern arbeitsteilig bearbeiten.…gute Erfahrungen, einen Beispielfall arbeitsgleich zu bearbeiten…sehr unterschiedliche (vergleichbare) Ergebnisse zu einer Ausgangsidee		…gute Möglichkeit zu Ergebnissicherung für das LF12. Für leistungsschwächere S aber eher schwierig durchzuführen.
Personaleinsatz planen und Mitarbeiter führen	LF 13<br>TS1	..zwei Unterrichtsstunden ..nicht ausreichend.	..es fehlen zur Orientierung die Ziele der Personalwirtschaft..<br>..was ist mit Arbeitskraft..als wichtiger Einsatzfaktor gemeint?		
	TS2		..was ist mit relevanten Größen gemeint?	Tarifrechtliche/gesetzliche Bestimmungen jetzt in WuG	
	TS3		..was ist gemeint mit „allgemeinen Unternehmensvoraussetzungen“?		
	TS4	…sollte Stundenumfang gekürzt werden (zu Gunsten TS5)..	Erstellen eines Arbeitsvertrages… Hier sollte auf fertig formulierte Vertragsbeispiele zurückgegriffen werden. Die Auszubildenden erhalten eine kaufmännische und keine juristische Ausbildung.	Tarifverträge, Betriebsvereinbarungen aufbauend auf WuG	Rechtliche Aspekte, die vom S gar nicht überblickt werden können
	TS5	Für diese TS könnten dann mehr Stunden zur	Berechnung des Gehaltes.. komplizierter geworden	Tarifverträge siehe auch WuG	S haben z.T. Schwierigkeiten, sich

	Verfügung stehen. Eingesparte Zeit sollte TS 7 „Konflikte bewältigen“ zugeschlagen werden.			diese Regelungen zu merken
TS6		Kündigungen…sehr komplex.. klären, wie weit inhaltlich auf diese rechtlichen Probleme eingegangen werden kann	Überschneidung mit WuG	
TS7	Komplexes Thema, für das mehr als 5 Stunden zur Verfügung stehen müssten..	..muss geklärt werden, wie tiefgehend die TS bearbeitet werden soll		
TS8	Gekürzt z.G. TS5	Zu hoher Anspruch: Zielerreichung mit Hilfe von Personalentwicklungsmaßnahmen optimieren		
Ein Einzelhandelsunternehmen leiten und entwickeln<br>LF 14<br>TS1			Sequenzierung muss angepasst werden	..ist auf Prüfungsinhalte zu unterrichten
TS2	Zeitlich ungünstig und kaum realistisch in der verfügbaren Zeit durchzuführen..		Handlungsvollmacht/Prokura/Rechtsformen werden in WuG unterrichtet	..Gründung stellt eine Überforderung für S dar.. Anspruch..trifft nicht die Interessenlage<br>..S haben starkes Interesse an Prüfungsvorberitung und Wiederholungen zu anderen LF-Inhalten
TS3/TS			Zu den Finanzierungsmöglichkeiten sollte	

4			überlegt werden, ob solche Inhalte nicht schon in vorhergehenden LF zu behandeln wären	
TS5	Ggf. aus Zeitgründen streichen..	Maximal inner- und außerbetriebliche Gründe einer Unternehmenskrise… ..Reduktion der Inhalte…	Privatinsolvenz passt nicht in LF 14 -> macht Sinn in WuG	prüfungsrelevant? …um für S bearbeitbar zu machen
Alle TS	..wegen der kurzen zur Verfügung stehenden Zeit..		Großteil der Inhalte wurde in WuG vermittelt	..in einer sehr leistungsschwachen Tankstellenklasse sind ..prüfungsrelevante Themen..verkürzt, also nicht handlungsorientiert unterrichtet worden..

Anhang 6: Auswertung der Evaluationsbögen Teil B

Hinweis: Es wurden lediglich Äußerungen mit Bezug zu im Arbeitskreis genannten Kategorien ausgewertet. Der verbleibende überwiegende Teil der Äußerungen bezieht sich auf die Zuordnung von Kompetenzdimensionen zu den korrekten Lernfelder/Teilsequenzen. Dies ist essentieller Kern des curricularen Entwicklungsprozesses und kann daher keine Hinweise auf etwaige Verbesserungsbedarfe der curricularen Strategie liefern.

Fundort	Kompetenzformulierung	Kompetenzmessung	Umsetzbarkeit	Qualifikation der Lehrkräfte
LF1 WUC	…viel zu abgehoben…			
LF1 LAT	.		In schwachen Klassen noch sehr viel Hilfestellung und Feedback notwendig. S benötigen Regeln und Tipps für Gruppenarbeiten. S benötigen Tipps, um Arbeitsaufträge zu verstehen, die Zeit richtig einzuteilen..	
LF1 LAT			S haben ..keine Vorstellung davon, wie sie an Informationen gelangen können und wie sie die wesentlichen Informationen selektieren und komprimieren können	
LF1	…an vielen Kompetenzen wird nur gekratzt, weil sie sehr aufgebläht erscheinen..			
LF2 BWP	..Auswirkungen der Kaufmotive auf die Sortimentsplanung..zu weit gegriffen			
LF2 BWP	Produktlebenszyklus an dieser Stelle überzogen			
LF3 BE	zu hohe Erwartung an S,		fehlende Sensibilität für psychische Belastungen	

LF4 LAT			S erhalten Lernmaterial, aber erarbeiten nicht extra welches	
LF5 LAT	Gruppenarbeit für den eigenen Prozess nutzbar machen – was bedeutet das?			
LF7 BE	S können physische Anstrengungen beim Annehmen und Einlagern der Ware einschätzen.	Diese Kompetenz ist nicht überprüfbar.<br>Durch ein Lehrer-Schüler-Gespräch möchte ich nicht einschätzen müssen, ob die S die Kompetenz tatsäch-lich erlangt haben.	Sollte das nicht der Betrieb leisten?	Ich möchte auch nicht „rückenschonende Hebetechniken“ mit ihnen einüben.
LF7 LAT			Arbeit mit dem WWS kommt zu kurz	
LF8 LAT	Die S können Software nutzen		machen wir nicht	
LF8 WUC	Interpretation der G+V		bei uns nicht erfolgt	
LF8 BWP			Erschließt sich beim wirtschaftsinstrumentellen Ansatz erst in TS 2, da aus der Handlung Sinnhaftigkeit einer Buchhaltung erkannt wird	
LF8 WUC			Erschließt sich erst in TS3, die beim wirtschafts-instrumentellen Ansatz nach TS 1 folgt	
LF9 LAT			Arbeit mit Excel entfallen aus zeitlichen Gründen	
LF10 KOKO			Aufgrund der knappen Zeit nicht alle dort angesprochenen Verkaufs-situationen angesprochen	
LF10 LAT	Welche Formel soll angewendet werden? Was davon ist prüfungsrelevant?			

LF10 NORM	Sprengt den Rahmen			
LF10 WUC	S verstehen Einfluss von Finanzierungen auf die Liquiditätssituation – glaube ich nicht!			
LF11	Inhalte sind für einige S weit weg von ihrem betrieblichen Alltag…dies ist sicherlich auch von der einzelnen Klasse abhängig.<br>Bei leistungsstarken S können die ..Kompetenzen gefördert werden		Wie können kurz- und langfristige Auswirkungen der Steuerung der Geschäftsprozesse für die Mitarbeiter inhaltlich deutlich gemacht werden?	
LF12 LAT			Aufgrund des Zeitdrucks war die Umsetzung von LAT problematisch, da zeitaufwändiger und der Fokus auf dem prüfungsrelevanten Wissen lag	
LF13 BE			Was ist davon wirklich bei den S angekommen?	
LF 13 NORM	Die Ansprüche an die S sind aus meiner Sicht überzogen und sollten auf ein realistisches Niveau korrigiert werden.			
LF13		Die Formulierung der Kompetenzdimensionen lässt häufig nicht erkennen, welche tatsächlichen Kompetenzen die S konkret und überprüfbar zeigen sollen, weil sie zu allgemein gehalten sind. Dies		

		erschwert die Erstellung von konkretem Unterrichtsmaterial.		
LF14			Da keine Neugründung geplant ist, werden Fragen der Standortwahl oder Absatzstatistiken nicht behandelt. Ein Unternehmenskonzept wird nicht erstellt. Umsetzung der Kompetenzen ist in der Unterrichtspraxis nicht in der Form erfolgt wie in der Strukturplanung vorgesehen	
LF1		*Sinnvoll wäre, ein AB zu entwickeln, um die Kompetenzen am Anfang und Ende der Sequenz oder LF zu messen*	*Thema Kompetenzen für einige schwierig zu begreifen (Tankklasse).*	
LF1 LAT			*Phasen der Projektarbeit zu schwer für S*	
LF1	*Kompetenzen teilweise zu hoch angesetzt*	*Überprüfbarkeit der Kompetenzen ist schwierig/unmöglich*		
LF2 LAT			*Umgang mit Excel von den Rahmenbedingungen abhängig, sollte optional sein*	
LF2 BE2	*Zu viel für den Anfänger einer Ausbildung*			
LF2 BE4			*Ist zwar wichtig, aber kann bei der Adressatengruppe nicht erreicht werden*	
LF2 BWP	*Produktlebenszyklus geht zu weit*			

LF2 BWP2			*z.T. schwierig wegen Betriebsform der Ausbildungsbetriebe (Kiosk)*	
LF8 LAT			*Excel optional, soweit Zeit vorhanden*	
LF8 BWP			*Einsatz von Buchhaltungsprogrammen erscheint unrealistisch*	

Anhang 7: Einsatz der Makroplanung LF 6, TS 4 in einem exemplarischen Unterricht

	**Lernfeld 1:**	**Das Einzelhandelsunternehmen repräsentieren**	**1. Ausbildungsjahr**<br>**Zeitrichtwert: 80 Std.**
	**III Makroplanung**		
•	• **Teilsequenz 4: Aufgaben und Rechte als Auszubildender im dualen System wahrnehmen**		
	**Zielformulierung/Kompetenzen**	**Wissensbasis**	
	**BE- Identität und Berufsrolle:**<br>Die Lernende kennen ihre neue Rolle als Auszubildende bzw. Arbeitnehmer im Einzelhandel. Sie kennen die Regelungen und Inhalte der Ausbildung zur Kauffrau/zum Kaufmann im Einzelhandel sowie ihre Rechte und Pflichten als Arbeitnehmer. Sie wollen diese Rollen zur eigenen Zufriedenheit und zur Zufriedenheit ihrer Ausbildungsbetriebe übernehmen. Die Lernenden wissen, dass die Kundenorientierung das zentrale Leitprinzip ihres beruflichen Handelns ist und wollen es beachten. Sie verfügen über ein positives Selbstverständnis in ihrem Beruf. Aufgrund ihrer Kenntnisse über die Inhalte und Entwicklungsmöglichkeiten des Berufs können sie sich gegenüber Mitmenschen selbstbewusst präsentieren und das Berufsbild entsprechend darstellen.<br><br>**KOKO – Kommunikation und Kooperation:**<br>Die Lernenden können ihre Rechte in der Ausbildung ggü. Vorgesetzten argumentativ vertreten, indem sie ihre Anliegen angemessen formulieren. Dabei berücksichtigen sie ihre Rolle als Auszubildender gegenüber dem Vorgesetzten.<br><br>**LAT – Lernhandeln planen und optimieren:**<br>Die Lernenden<br>können ihre Stärken und Schwächen einschätzen, ihren persönlichen Lerntyp charakterisieren und kennen Möglichkeiten zur Gestaltung	• Kundenorientierung als Leitbild<br>• Gliederung der Ausbildung und Entwicklungsperspektiven<br>• Berufsbild und Selbstverständnis<br><br>• Argumentationstechnik (SuK)<br>• Rollenbegriff, -klarheit, -distanz<br>• Erwartungen an Auszubildende<br><br>• Lernziele und Lerninhalte der Berufsausbildung,<br>• Lerntypen, Bestimmungsfaktoren für ein positives Lernklima<br>• Lernstrategien, Lernen lernen<br>• Kooperatives Lernen	

	ihres Lernprozesses. Die Lernenden wissen, dass sie auch Verantwortung für die Lernatmosphäre in ihrer Klasse tragen und hierdurch auch zum Lernerfolg der Mitschüler/-innen beitragen können.<br><br>**NORM – Arbeits- und Schutzrecht**:<br>Arbeitsrecht aus Sicht des Auszubildenden:<br>Die Lernenden kennen die für sie wichtigen gesetzlichen Regelungen, die ihre Ausbildung und ihre berufliche Tätigkeit betreffen. Sie können die Regelungen beschreiben und beurteilen sowie auf ihre eigene Berufstätigkeit beziehen. Die Lernenden kennen Rechte und Pflichten von Arbeitnehmern und Arbeitgebern und können diese nutzen, um die eigene Lage zu beurteilen und Handlungsalternativen zu entwickeln.<br><br>Die Lernenden kennen die im Einzelhandel relevanten Tarifverträge und aktuellen Tarifverhandlungen. Sie können Interessenskonflikte der Sozialpartner beschreiben und kennen die Phasen von Tarifverhandlungen. Die Lernenden erkennen die Notwendigkeit des Sozialversicherungssystems und kennen die unterschiedlichen Träger, Leistungen und Beiträge. Sie beurteilen die Leistungsfähigkeit und kennen Möglichkeiten der privaten Vorsorge. Sie kennen die besondere Bedeutung und die Aufgaben der Berufsgenossenschaften.	• Berufsbildungsgesetz<br>• Jugendarbeitsschutzgesetz<br>• Berufsausbildungsvertrag<br>• Arbeitszeitverordnung, Sonntagsarbeit<br>• Bundesurlaubsgesetz<br>• Betriebsverfassungsgesetz<br>• Betriebsrat, Jugend- und Auszubildendenvertretung<br>• Arbeitssicherheit (i. A.)<br>• Kündigung<br>• Bildungsplan, Ausbildungsordnung, Prüfungen<br>• Tarifverträge, Tarifverhandlungen, Autonomie<br>• Sozialversicherungssystem, -träger<br>Berufsgenossenschaften

	**Lernschritt laut Makroplanung**<br>**Lernschritt laut Unterrichtsplanung**	**Lernhandlungen**<br>*Was tut der Lernende?*	**Lernaufgaben/-situation**<br>*Impuls des Lehrenden*	**Konkrete Umsetzung**
A	Berufsausbildungsvertrag<br><br>Ziele und Inhalte der dualen Ausbildung	▪ *S recherchiert nach dem BGB im Internet*<br>▪ *Versteht, wie ein Vertrag zustande kommt*	▪ *LS-Gespräch*<br>▪ *AB „Der Berufsausbildungsvertrag - Teil I“ (ca. 20 Min)*	Entfallen, da Schwerpunkt im Lernfeld 7 TS 3<br><br>Stattdessen: eigenes AB zur beruflichen Handlungskompetenz und Übersicht Lernfelder

B	Exkurs:<br><br>Umgang mit Gesetzestexten<br><br>Lernhandeln planen und optimieren	▪ *S recherchiert Quellen im Internet*<br>▪ *Liest Gesetzestexte*<br>▪ *Versteht Gliederung von Gesetzestexten*<br>▪ *Arbeitet in der Gruppe*<br>▪ *Füllt Tabelle aus (ca. 30 Min)*<br>▪ *Erörtert Ergebnis vor der Klasse (ca. 30 Min)*	▪ *Auszug aus dem BBiG*<br>▪ *AB „Umgang mit Gesetzestexten“*	Entfallen, da optional<br><br>Stattdessen: eigenes AB zwecks Förderung der Kompetenz LAT
C	Fortsetzung:<br><br>Berufsausbildungsvertrag<br><br>Orientierung hinsichtlich relevanter Rechtsquellen	▪ *S beantwortet Fragen mithilfe des BBiG eigenständig*<br>▪ *S arbeitet in der Gruppe*<br>▪ *S trägt Ergebnis der Klasse vor*	▪ *AB „Der Berufsausbildungsvertrag – Teil II“ (ca. 90 Min)*	Mindmap Rechtsquellen für Einzelhandelskaufleute<br><br>AB in leicht modifizierter Form eingesetzt zwecks erster Orientierung (Internetrecherche) in den relevanten Rechtsquellen<br><br>Zur Ergebnissicherung Lösungen ausgehändigt. Übersicht zum JArbSchG neu erstellt inkl. Regelungen für Volljährige, da nur zwei Minderjährige in der Lerngruppe.<br><br>Handlungsorientierte Vertiefung in den nachfolgenden Sequenzen
D	Rechte und Pflichten aus dem Ausbildungsvertrag	▪ *S wendet seine Kenntnisse aus den vorangegangenen Stunden an*<br>▪ *S füllt eine Übersicht selbstständig aus*<br>▪ *S erörtert Ergebnis vor der Klasse*<br>▪ *S löst in der Gruppe Fälle zum BBiG*<br>▪ *S präsentiert Ergebnisse vor der Klasse*	▪ *AB „Rechte und Pflichten in der Ausbildung“ (ca. 30 Min)*<br><br>▪ *AB „Fälle BBiG“ (ca. 45*	Dieses AB fehlte im Eva-Net-Ordner<br><br>Hier Arbeitsauftrag „Ausbildungsvertrag“ in branchenspezifisch modifizierter Form eingesetzt (aus nachfolgender Sequenz vorgezogen)

			Min)	AB “Fälle BBiG“ bearbeitet
E	Jugendarbeitsschutzgesetz	▪ *S versteht den Sinn und Zweck des JArbSchG*<br>▪ *S erarbeitet wesentliche Regelungen zum Jugendarbeitsschutz selbstständig mithilfe des Gesetzes*<br>▪ *S arbeitet in der Gruppe*<br>▪ *S hält Ergebnisse in einer Übersicht fest*<br>▪ *S trägt Ergebnisse der Klasse vor*<br>▪ *S löst Fälle zum JArbSchG*<br>▪ *S diskutiert in der Gruppe*<br>▪ *S argumentiert vor der Klasse anhand der Vorschriften*<br>▪ *S prüft Vertrag auf seine Richtigkeit bzgl. BBiG und JArbSchG*	▪ *Auftrag + Übersicht „Jugendarbeitsschutz“ (ca. 45 Min)*<br>▪ *Auftrag + Fälle „Jugendarbeitsschutz“ (ca. 45 Min)*<br>▪ *Auftrag „Ausbildungsvertrag“ und Vertrag (ca. 45 Min)*	Auftrag + Fälle „Jugend-arbeitsschutz“ mit Hilfe der selbst erstellten Übersicht aus Sequenz C
F	Das duale Ausbildungssystem	▪ *S reflektiert seine Berufsausbildung*<br>▪ *S kennt die beiden Zweige Ausbildung in Betrieb und Schule*<br>▪ *S füllt Übersicht mithilfe eines Info-Blattes aus*	▪ *LS-Gespräch*<br>▪ *Übersicht „Das Duale System der Berufsausbildung“ (ca. 15 Min)*<br>▪ *Info-Blatt „Das duale System“*	Vorgezogen (bereits zwischen Sequenz B und C behandelt)<br>„Übersicht „Das Duale System der Berufsausbildung“ bearbeitet mit Hilfe des Info-Blattes und

		▪ *S liest einschlägige Vorschriften im BBiG nach*<br>▪ *S trägt Ergebnisse der Klasse vor*	▪ *Übersicht „Berufsausbildung – rechtliche Rahmenbedingungen“ (ca. 20 Min)*	einer einschlägigen Powerpoint-Präsentation<br><br>Ergebnissicherung an Pinnwand wegen Verständnisschwierigkeiten (AB zu abstrakt)<br><br>Übersicht „rechtliche Rahmen-bedingungen“ war nicht im EVA-Net-Ordner
G	Betrieblicher Ausbildungsrahmenplan	▪ *S reflektiert den eigenen Ausbildungsvertrag und seine Anlagen*	▪ *Anlage zum Berufsausbildungsvertrag „Sachliche und zeitliche Gliederung der Berufsausbildung“ (ca. 15 Min)*	Als Anlage zum Ausbildungsvertrag in Sequenz D mitbehandelt.
H	Schulische Lehrpläne	▪ *S reflektiert den eigenen schulischen Ausbildungsplan*	▪ *Übersicht „Lernfelder für den Ausbildungsberuf Kaufmann / -fau im Einzelhandel und Verkäufer / -in“ (ca. 10 Min)*	Bereits in Sequenz A mit behandelt
I	Prüfungsstrukturen und Inhalte	▪ *S erarbeitet anhand der Verordnung Fragen zur Abschlussprüfung*	▪ *Ausdruck „Verordnung über die Erprobung abweichender Ausbildungs- und Prüfungsbestimmungen in der Berufsausbildung im Einzelhandel…“*<br>▪ *AB „Die Abschlussprüfung“ (ca. 30 Min)*	Hinweis auf Verordnungen in Sequenz C .<br><br>Behandlung prüfungsrelevanter Aspekte laut Lehrkraft an dieser Stelle zu früh und zu komplex.
