# Supplementary material for: Theme-centered interaction and developmental tasks as research method and pedagogical tool regarding identity development in VET
Source: Front Psychol. 2023 Oct 10;14:1201305. doi: 10.3389/fpsyg.2023.1201305 (PMC10597703; doi:10.3389/fpsyg.2023.1201305)
Supplement: Supplementary file 4 [file Data_Sheet_4.PDF]

Supplement 4: Sample for case studies

Codename	Retail sector	Company size	Gender	migration background	Age	1. Interview	2. Interview
**Ciara**	Telecommunication	large	F	Yes	18	05.07.2016	12.06.2017
**Ahmet**	Filling station	small	M	Yes	24	09.05.2016	25.04.2017
**Markus**	Automotive equipment	middle	M	No	19	07.06.2016	29.03.2017
**Jennifer**	Consumer electronics	large	F	No	20	08.07.2016	09.06.2017
**Lara**	Consumer electronics	large	F	No	21	14.06.2016	30.06.2017
**Nils**	Food/Non-Food	large	M	No	20	19.04.2016	10.04.2017
**Bastian**	Telecommunication	large	M	No	21	18.07.2016	21.06.2017
**Jasper**	Filling station	small	M	Yes	23	13.05.2016	dropout
**Berat**	Filling station	small	M	Yes	18	13.05.2016	06.06.2017
**Alina**	Telecommunication	large	F	No	27	26.04.2016	12.06.2017
**Hendrik**	Food	large	M	No	18	06.04.2016	18.04.2017
**Kostas**	Jeweler	small	M	Yes	19	07.06.2016	12.05.2017
**Sophie**	Jeweler	large	F	Yes	18	07.06.2016	14.06.2017
**Hauke**	Consumer electronics	large	M	No	26	21.06.2016	19.05.2017
