# Supplementary material for: Theme-centered interaction and developmental tasks as research method and pedagogical tool regarding identity development in VET
Source: Front Psychol. 2023 Oct 10;14:1201305. doi: 10.3389/fpsyg.2023.1201305 (PMC10597703; doi:10.3389/fpsyg.2023.1201305)
Supplement: Supplementary file 5 [file Data_Sheet_5.pdf]

Supplement 5a- Guideline 1st interviw

What is the apprentice's self-concept at the beginning of the apprenticeship?

Socio-cultural background:

Gender: m/f Age: Nationality: Company:

1. How have you grown up (siblings, parents' vocation, living situation, domicile, school)? What did you like to spend time on? What did you dislike?
2. Have there been any critical incidents in your childhood/adolescence, that have had an impact on your life? Please describe? *(e. g. parents' divorce, strokes of fate, removals, if applicable question noticeable aspects from question 1)*
3. How would you describe the parenting style you experienced? Please give examples.

Self-concept:

4. How would you describe yourself? How would your parents/peers describe you?
5. What are you good at? What is difficult for you?

Career choice motives:

6. How did you happen to start an apprenticeship in the retail sector? Why do you think the vocation suits you?
7. Which goals do you pursue with your apprenticeship?

Values/Life goals:

8. What is important for you apart from the apprenticeship, e. g. in private life (boy-friend/girl-friend, peers, hobbies)?
9. How would you personally rank the following life goals?
   - Having power/a status/prestige
   - Develop and improve competences/abilities
   - Entertainment/Fun
   - Trustful relations with others
   - To undertake something with others
   - Help and support others

Typical vocational conflicts/problems:

10. How did you experience the first months of your apprenticeship? What did you enjoy? What was difficult for you or what did you dislike? Did you experience any problems/difficulties? *(if applicable ask explicitly for the following typical problems:*
   - *Working hours/-rhythm*
   - *New role as apprentice/School-to-work transition*
   - *Dealing with mistakes/missing knowledge and skills/demands of the vocation)*

Vocational action competence:

11. Please describe your workplace (superiors, colleagues, company, customers, work tasks). What activities/subject matters have you dealt with **at your workplace** during the first months? Please give examples.

	Details
Knowledge about the company	
Economical role of the retail sector	
Rights and duties during the apprenticeship	

Merchandise management	
Protection of the environment	
Customer-orientation/ Customer communication	
Publicity	
Presentation of health	
Security/Health	
Cashpoint	
Cashpoint settlement	
Product expertise	
Teamwork/Work organization	

12. What do superiors, customers and colleagues primarily expect from you?
    How do you feel facing these expectations? What feedback do you receive from superiors, customers and colleagues?

Change in career choice motives:

13. Would you still decide to start an apprenticeship in the retail sector? Why?

What is the state of identity-related competences?

Self-presentation/Role distance/emotional competence/personal assessment of situations:

14. Please describe a challenging situation you experienced during the first months of your apprenticeship *(if applicable refer to aspects from question 10).*
    - What was the problem?
    - How did you deal with the situation?
    - How did superiors, colleagues and customers react?
    - What did you learn from this experience?

Dealing with dilemmas:

15. How would you assess the following situation? Would you sell alcohol to the customer? Please give reasons.

    How would you assess the following arguments, if a vendor argued this way?

A primed regular customer comes to the cashpoint und wants to buy three bottles of spirits. You know his wife and that he has a severe problem with alcohol addiction and that he will start a withdrawel treatment soon.

**Pro arguments for selling spirits**	acceptable	rather acceptable	Rather inacceptable	inacceptable
Selling spirits is only prohibited for adolescents.<br>As the customer is of full age, I am allowed to sell spirits.(4)				
I have to accept the customer’s freewill to harm himself. (6)				
If I do not sell the spirits, he will buy them in another shop.(3)				

My superior would criticize me if I impair turnover.(1)				
The customer expects me to sell the spirits and selling is my job. (2)				
Unfortunately, my company does not considerate ethical issues. (5)				
**Contra arguments against selling spirits**	Acceptable	Rather acceptable	Rather inacceptable	inacceptable
Another customer could complain about me to my superior. (1)				
I would have a guilty conscience afterwards. (2)				
The majority of my customers would not appreciate, that my employer makes profit with peoples' illness. (3)				
The mission statement of my employer obliges me to act in a socially responsible way. (4)				
It should be prohibited to sell spirits to addicted people. (5)				
Because of his addiction, the customer does not have a free will.(6)				

Learning environment

16. Which support have you received during the first months of your apprenticeship? Which support would you have needed?
17. What was especially helpful at VET school? What did you miss?
    Which learning contents were useful for you or not? Why?

**Curriculum content**	**Please delete where inapplicable**	**Reasons**
Presentation of the company	Helpful/less helpful/unnecessary	
Rights and duties in the apprenticeship	Helpful/less helpful/unnecessary	
Economical role of the retail sector	Helpful/less helpful/unnecessary	
Sales conversation with customers	Helpful/less helpful/unnecessary	
Cashpoint service	Helpful/less helpful/unnecessary	
Individualized learning	Helpful/less helpful/unnecessary	
Learning status talk	Helpful/less helpful/unnecessary	

Please assess yourself concerning the following statements. Please tick the applicable box.

**Sociableness/ Decision-making/Problem-solving/ Stableness of self-presentation**<br>Deusinger (1986)<br>Non-validated translation from German	Very true	True	Rather true	Less true	Not true	Not true at all
1. I have a kind of dealing well with others.	1	2	3	4	5	6
2. It is easy for me to make contact with others.	1	2	3	4	5	6
3. I am not shy of entering a room in which people are already sitting together and talking.	1	2	3	4	5	6
4. I should be more polite to others.	1	2	3	4	5	6
5. I am rather shy and unsure in contact with other people.	1	2	3	4	5	6
6. I am not afraid of meeting foreign people.	1	2	3	4	5	6
7. I am rather unsure to assess my own skills.	1	2	3	4	5	6
8. I often feel like apologizing for my behavior.	1	2	3	4	5	6
9. In most cases I can decide in a quick and determined way.	1	2	3	4	5	6
10. I can come to important decisions without help.	1	2	3	4	5	6
11. I think I can justify my behavior in most cases.	1	2	3	4	5	6
12. I have difficulties to do what is right.	1	2	3	4	5	6
13. When I am part of a group, I don't dare to say anything.	1	2	3	4	5	6
14. I often do not agree with others, but I am afraid of expressing my criticism frankly.	1	2	3	4	5	6
15. I advance my view in a group that does not agree with me.	1	2	3	4	5	6
16. When I disagree I even contradict authorities.	1	2	3	4	5	6
17. It worries me when I get the impression that somebody does not agree with me.	1	2	3	4	5	6
18. In my life I comply too much with others' points of view.	1	2	3	4	5	6
19. To avoid having ennemies I often agree with opinions or decisions that appear not justifiable to me.	1	2	3	4	5	6
20. I am afraid to say no.	1	2	3	4	5	6
21. It is difficult for me to express my opinion in front of a group.	1	2	3	4	5	6
22. I have difficulties to express my opinion in front of a group even if I have something important to say.	1	2	3	4	5	6
23. It is difficult for me to object to a group's point of view.	1	2	3	4	5	6
24. In a group I feel unsure, as others have more ideas than me.	1	2	3	4	5	6
25. I easily loose my temper.	1	2	3	4	5	6
26. I easily cope with my personal problems.	1	2	3	4	5	6
27. I will also cope with my problems in the future.	1	2	3	4	5	6
28. I can look after myself in any situation.	1	2	3	4	5	6
29. My personal problems exist to be solved.	1	2	3	4	5	6
30. I get along as well as others.	1	2	3	4	5	6
31. I try to escape from my problems.	1	2	3	4	5	6
32. I wish I would not give up that easily.	1	2	3	4	5	6
33. I am looking ahead hopefully.	1	2	3	4	5	6
34. Nothing derails me easily.	1	2	3	4	5	6

**Ambiguity tolerance**<br>Lind (1987)<br>Non-validated translation from German	I reject completly	I rather reject	I partially reject	I rather agree	I largely agree	I agree completely
1. An expert who does not give a clear answer is probably not that competent.	1	2	3	4	5	6
2. A person who subordinates his/her life to a scheme misses lust for life.	1	2	3	4	5	6
3. The most interesting and inspiring people are often those who do not mind to be different from others.	1	2	3	4	5	6
4. Teachers who do not assign precise tasks give the chance to demonstrate initiative and originality.	1	2	3	4	5	6
5. A person who insists on a yes or no has not realized how complicated things really are.	1	2	3	4	5	6
6. I prefer invitations where I know most people to those where some people are strangers.	1	2	3	4	5	6
7. The earlier all people share the same values and ideals the better.	1	2	3	4	5	6
8. A person who lives a calm and orderly life in which few surprises and unexpected events occur can really be grateful.	1	2	3	4	5	6

**Emotional Competence**<br>Rindermann (2009)<br>Non-validated translation from German	Not true at all	Rather not true	middle	Rather true	Absolutely true		
1. In precarious situations I try to keep calm.	1	2	3	4	5	3,62	RE
2. When I am doing badly I do not tell others as this could bother them.	1	2	3	4	5	3,11	EX
3. I quickly realize when others feel bad.	1	2	3	4	5	3,91	EA
4. Sometimes I am sad without knowing why.	1	2	3	4	5	2,98	EE
5. When I feel anger rising I know how to calm down.	1	2	3	4	5	3,26	RE
6. Nothing will upset my easily.	1	2	3	4	5	3,09	RE
7. I can understand others' feelings well.	1	2	3	4	5	3,81	EA
8. I can control my anger and rage.	1	2	3	4	5	3,27	RE
9. I can infer from others' facial expression how they feel.	1	2	3	4	5	3,59	EA
10. I do not turn my inside out.	1	2	3	4	5	2,81	EX
11. Sometimes my mood is changing although I do not know why.	1	2	3	4	5	2,91	EE
12. I can put myself in others' emotional position.	1	2	3	4	5	3,53	EA

13. When I become angry I am hardly able to control myself.	1	2	3	4	5	3,3	RE
14. Others call me empathetic.	1	2	3	4	5	3,43	EA
15. I often talk about my emotions.	1	2	3	4	5	2,7	EX
16. When I talk to a friend on the phone I feel how he is doing.	1	2	3	4	5	3,51	EA
17. I often do not understand my mistakes.	1	2	3	4	5	3,37	EE
18. I often tell others how I am doing.	1	2	3	4	5	2,93	EX
19. Sometimes I do not know how I happened to feel bad.	1	2	3	4	5	3,2	EE
20. When my emotions change I nearly almost know the cause.	1	2	3	4	5	3,4	EE
21. Even in difficulties I can maintain my inner balance.	1	2	3	4	5	3,18	RE
22. I have problems to express my feelings.	1	2	3	4	5	3,24	EX
23. Sometimes I cannot understand my own behavior.	1	2	3	4	5	3,03	EE
24. I prefer not to talk about my feelings.	1	2	3	4	5	2,88	EX
25. I can easily understand others feelings and behavior.	1	2	3	4	5	3,63	EA
26. I often realize the trigger for emotional fluctuations.	1	2	3	4	5	3,34	EE
27. When I am angry I have difficulty to calm down.	1	2	3	4	5	2,95	RE
28. I show frankly how I feel.	1	2	3	4	5	2,92	EX
29. Others realize more quickly than me that I am doing badly.	1	2	3	4	5	3,22	EE
30. In the course of a talk I realize quickly how my conversional partner is doing and when his/her mood changes.	1	2	3	4	5	3,68	EA
31. I could get upset for hours about bad events and news.	1	2	3	4	5	2,97	RE
32. I have learned not to talk about my feelings.	1	2	3	4	5	3,67	EX
33. Others contact me when they are doing badly.	1	2	3	4	5	3,36	RA
34. In difficult situations I can calm others.	1	2	3	4	5	3,57	RA
35. When others become angry I manage to balance the atmosphere.	1	2	3	4	5	3,28	RA
36. I easily cope with others‘ extreme emotions.	1	2	3	4	5	3,29	RA
37. Others say that I am helpful when they are doing badly.	1	2	3	4	5	3,56	RA
38. I easily motivate others.	1	2	3	4	5	3,5	RA
39. I have difficulties to counterbalance others’ emotions.	1	2	3	4	5	3,45	RA
40. I can easily influence others‘ feelings.	1	2	3	4	5	3,11	RA

Supplement 5b – Guideline 2nd interview

Questions concerning the course of apprenticeship during the second year

1. In the first interview we talked about the initial phase of your apprenticeship. Meanwhile, the end of the 2nd year approaches. Please report how you experienced the time since the 1st interview.

Curriculum content of the 2nd year (continued from 1st interview)

According to the VET school curriculum respectively the workplace curriculum you should have dealt with the following subject matters during the second year. Please report if you did so and how you experienced them

	**Workplace**	**School**
Waren präsentieren, LF 4	Done during 1st year	
Werbemaßnahmen, LF 5	Done during 1st year	
Customer complaints (LF 10)		
**Customer service**		
**Pricing** LF 9 Special offers		
**Stock control, inventory,** LF 7		
**Goods receipt and storing** LF 7		
Cost accounting LF 9		
Purchasing LF 6	Not due	
Accountig, Profit and loss, balance sheet LF8	Not due	
Optional matters		

Which subject matters from "Language and communication" and „Economy and society" did you perceive as especially helpful and useful?

Goals after the apprenticeship

2. During the first interview you reported that you pursue the following goals:….. Have your expectations been meet and did you reach your goals?

3. When will you finish your apprenticeship? What will be your next steps (after the apprenticeship)? Which opportunities do you see? Which risks? What would you do if anything was possible? Why do you hesitate to do so?

4. You have stated the following life goals: .... ? How do they reconcile with your current vocation?

Amendments of self-concept

5. How whould you answer the following question today: "What do you do for a living?"
   - How satisfied are you with your vocation as a retail salesperson and/or merchant?
   - How satisfied are your with your company?
   - What do you like most in your vocation?
   - Do you feel sufficiently recognized?
   - What meaning do you see in your work?
   - Do you sometimes feel stressed? In what kind of situations?

6. What experience during your apprenticeship was formative for you? (experience that has changed you in a sustainable way). What points of view did you change during your apprenticeship? Are there things you would do differently in the future?

7. During the last interview you did a self-assessment concerning the following characteristics:
   - Emotional competence (Rindermann, 2009)
   - Ambiguity tolerance (Lind, 1987)
   - Problem-solving (Deusinger, 1986)
   - Decision-making (Deusinger, 1986)
   - Stableness of self-presentation (Deusinger, 1986)
   - Sociableness (Deusinger, 1986)

   I would like to go through this with you again and compare if something has changed (cf. guideline 1st interview, supplement 4a)
   What were the reasons for the transformations in your opionion?

8. What distinguishes you from other retail salespersons/colleagues?
   a) Why should your employer be interested to hire you?
   b) Why would your customers appreciate you?

Coping

9 . In the last interview you had reported on the follwing adjustment difficulties and conflicts: …. How did this develop in the course of the apprenticeship?

10. Have there been any other major challenges since our last interview? How did you cope with them? What was helpful?

11. Please imagine the following vocational conflict. How would you react? Please give reasons.

Situation 1:
You have got two products on sale which are equal regarding quality. Product A is a branded product and 30% more expensive than own-label product B. Product A's margin is twice that of product B. A customer enters and is interested in such a product. How do you behave? Why?

Situation 2:
You have got a conflict with a colleague regarding workforce planning. She is supposed to work next Saturday, but she wants to swap working hours with you because of a funeral. You have got tickets for a concert that you have been looking forward to for so long. Your superior prompts you and your colleague to take a decision soon.

Situation 3:
Your company is willing to offer you a permanent contract after your apprenticeship. The position they offer you is low-paid and not in your preferred department. How do you react?
