# Supplementary material for: Theme-centered interaction and developmental tasks as research method and pedagogical tool regarding identity development in VET
Source: Front Psychol. 2023 Oct 10;14:1201305. doi: 10.3389/fpsyg.2023.1201305 (PMC10597703; doi:10.3389/fpsyg.2023.1201305)
Supplement: Supplementary file 6 [file Data_Sheet_6.PDF]

Supplement 6 – Coding Scheme for case studies

Note: These are categories derived from identity theories and qalitative research methodologies. They are likely to facilitate case understanding but need not be applied completely.

Statements about self-concept:

Statements from others about the person:

Sociocultural background (Bourdieu 2012)

- Symbolic capital
- Economical capital
- Social capital
- Cultural capital

Biographieanalyse nach Schütze (1983); Nohl (2006):

- Planned action schemes (life goals?)
- Internal and external contingencies
- Periods of personal transformation
- Institutionally induced life stages
- Relevant transformations since the last interview

Developmental tasks (Kutscha et al., 2009; Duemmler et al., 2017; Havighurst 1974):

Identification, Competence, Recognition, Shaping

Coding scheme for coping processes (crises, transitions, conflicts) for each event (Tiefel, 2005)

- *Which conflicts, transitions and/or challenges can be observed?*
- *How does the respondent react?*
- *How does he/she assess the situation?*
- *Which internal/external factors influence the decisions?*
- *Which identity-relevant abilities are shown?*
- *Which sustainable effects can be seen?*
- *Overall: Is there a common thread between the different situations?*

Documentary method (Bohnsack, 2014, Nohl, 2006)

WHAT is said?

Do self-assessment scales (Rindermann, 2009; Deusinger, 1986; Lind, 1987) coincide with what is reported? What is striking compared to others?

Reflexivity (Fonagy et al., 2016; Dauert, 2001) :

- Perception and consideration of own and others' emotions
- Reflexivity style
- Moral judgment ability:
  - Which moral judgment stage is shown (Kohlberg, 1984)?
  - Does the respondent tolerate others' opinions?
  - Is there a thread in the way of arguing?

Social dimensions of identity balance (Krappmann, 1975; Veith, 2010)
Role distance:

- Coping: way of problem-solving, self-assessment

- Goal orientation: existence of a reflexive project (Giddens, 1991), life goal ranking, Self-assessment decision-making/Stableness (Deusinger, 1986)

Empathy/change of perspective:

- Perception and regulation of others‘ emotions (reported and self-assessment (Rindermann, 2009))
- sociableness (reported and self-assessment (Deusinger, 1986))

Ambiguity tolerance:

- reported and self-assessment (Lind, 1987)
- *Objective hermeneutics (Oevermann et al., 1987)*
  Which statements are irritating or surprising? What would have been expected?
  Are there interrelations among the observations? Is the pattern recurring?

Self-presentation:

- Self-concept und Self-presentation
- Expressivität, regulation of own emotions (Rindermann, 2009)
- How does the person react to others' feedback?
- Comparison with others

HOW is it said?

Distinctive feature of the narrative style – comparison with others – Is the narrative style consistent with the content? Does the person (implictly) identify with a group?

Qualitative Content Analysis (Mayring, 2000): theoretical categories (deductive und inductive)

- Subjective assessment of the curriculum content
- Ecological factors in VET school and at the work place (Bronfenbrenner, 1979)

  Only cross-case analysis
