# Supplementary material for: Theme-centered interaction and developmental tasks as research method and pedagogical tool regarding identity development in VET
Source: Front Psychol. 2023 Oct 10;14:1201305. doi: 10.3389/fpsyg.2023.1201305 (PMC10597703; doi:10.3389/fpsyg.2023.1201305)
Supplement: Supplementary file 7 [file Data_Sheet_7.PDF]

Supplement 7 – Case analysis Ciara (after the 1st interview)

Statements about self-concept: reports predominantly perceptions by others (cf. below), no own idea of the self, more complex thinking than others (Ib, 62-71), shy, does not like to talk (Ib, 57), empathetic (Ib, 83), avoids contact (Ib, 86-89)

Statements from others about the person: smiles often (Ib, 58-59), calm, loner, not sociable (father's point of view, Ib, 74f.), other people say she is good at building relationships (Ib, 93), polite, friendly (Ib, 96-100), customers react in an understanding way (Ic, 158-161), is simulteously praised and critizised in the assessment discussion

Sociocultural background (Bourdieu 2012): Father is a migrant (Ia, 10), painter (Ib, 21), mother is German, grown up **without** her mother in a suburb of Hamburg (Ia, 19-24), younger sister has moved out with 15 (Ib, 77), father has often changed his girl-friends, another half sister aged 3 is living with the mother (Ib, 15), speaks German at home (Ib, 62-65), no intimate relationship with her father, respectful (Ib, 75)

- *Symbolic capital:* pretty, attractive, smiling, sympathetic
- *Economical capital:* depends on father's financial support
- *Social capital:* recognized by customers
- *Cultural capital:* school-leaving qualification (Ib, 36)

Biographical analysis (Schütze, 1983; Nohl, 2006):

- *Planned action schemes (life goals?):* could imagine to stay in the vocation, but only in the same company (Ic, 163),
- *Internal and external contingencies:* career choice was influenced by a counselor who has worked at the same company (Ib, 91), person-vocation fit was claimed by others (Ib, 93)
- Periods of personal transformation: would like to become more self-confident, mature and independent (Ic, 13)
- *Institutionally induced life stages:* happiest time of life at the nursery, sheltered childhood (Ia, 16), felt well at school (Ib, 39), school-leaving qualification (Ib, 36), school-to-work-transition most dramatic event in life (Ib, 47)
- Relevant transformations since the last interview

Developmental tasks (Kutscha et al., 2009; Duemmler et al., 2017; Havighurst 1974):

Identification: identifies with the company as there is a lot of support for apprentices, no identification with the vocation itself, would not dare to do an apprenticeship in another retail company (Ic, 162-165)

Competence: perceives the retail sector as a very demanding vocation (Ic, 165), has difficulty to learn the tariffs (Ic, 182-185), feels uncomfortable communicating with customers (Ib, 91),

Rocognition: is critizised by her superior as she does not know the tariffs, feels ashamed (Ic, 185), customers like talking to her (Ib, 95ff.)

Shaping: did not know what to do after school, happened to find the vocation/apprenticeship with support from others, others' advice is the most important orientation for her life (Ib, 90f.)

Coding scheme for coping processes (crises, transitions, conflicts) for each event (Tiefel, 2005)

- *Which conflicts, transitions and/or challenges can be observed?*

Self-concept and feedback from others do not coincide at all (Ib, 93)

- *How does the respondent react?*
  Compares herself to other apprentices (Ic, 48-51), aims to become more self-confident and experienced (Ic, 12-13)
- *How does he/she assess the situation?*
  It is her biggest problem (Ib, 93), thinks that the problem is not only due to her young age (Ic, 61)
- *Which internal/external factors influence the decisions?*
  Social comparisons (Ic, 48-51), she is younger and less experienced than the others, not mature, missing communication abilities (Ic, 61)
- *Which identity-relevant abilities are shown?*
  Does not manage to show her real self, she is recognized for a self other than her real self, ambiguity tolerance is strained, does not find any reasons for the contradictions (Ib, 59, 92), is successful to satisfy customers (Ic, 209), very empathetic
- *Which sustainable effects can be seen?*
  Depends largely on others' feedback for her decisions, appreciates feedback and support by others and her company as she feels disorientated
- *Overall: Is there a common thread between the different situations?*
  Absence of a real self-concept

Documentary method (Bohnsack, 2014, Nohl, 2006)

WHAT is said?

Do self-assessment scales (Rindermann, 2009; Deusinger, 1986; Lind, 1987) coincide with what is reported? What is striking compared to others?

Reflexivity (Fonagy et al., 2016; Dauert, 2001) :

- *Perception and consideration of own and others' emotions:*
  Extremely low perception and expression of own emotions, high score on perception and regulation of others' emotions
- *Reflexivity style:* relies on others' feedback and social comparisons, reflects on herself without result as self-perception is missing
- *Moral judgment ability:*
  - *Which moral judgment stage is shown (Kohlberg, 1984)?*
    Prefers higher stages of moral judgment but applies lower ones if her role demands this (Ic, 223-238)
  - *Does the respondent tolerate others' opinions?*
    Only if her role demands this.
  - *Is there a thread in the way of arguing?*
    Self-presentation relies on social acceptance

Social dimensions of identity balance (Krappmann, 1975; Veith, 2010)
Role distance:

- *Coping: way of problem-solving, self-assessment*
  no examples of autonomous problem-solving, low scores on decision-making and problem-solving (Deusinger, 1986), action requires stimuli from others, vital importance of feedback and support
- *Goal orientation: existence of a reflexive project (Giddens, 1991), life goal ranking, self-assessment decision-making/stableness (Deusinger, 1986)*
  Helping others and relatedness are the most important life goals (IC, 27ff.), would like to become more self-confident, mature and independent (Ic, 13)

Empathy/change of perspective:

- *Perception and regulation of others' emotions (reported and self-assessment Rindermann, 2009)*
  High score on perception and regulation of others' emotions, aims to satisfy others and succeeds to to so in interrelation with customers as they reflect that they are satisfied, helping others is the most important life-goal (Ic, 27-28)
- *sociableness (reported and self-assessment Deusinger, 1986)*
  extreme low score on sociableness contradicts customers' feedback

Ambiguity tolerance:

- *reported and self-assessment (Lind, 1987)*
  high score, but as the gap between own self-concept and others' feedback differs extremely and as this is perceived as her major problem she suffers
- *Objective hermeneutics (Oevermann et al., 1987)*
  Which statements are irritating or surprising? What would have been expected?
  Are there interrelations among the observations? Is the pattern recurring?
  She does not report any events of vital importance despite a missing mother, a distanced relationship with her father and his changing girl-friends, the departure of her sisters (Ib, 52-57), she has allegedly had a sheltered childhood (Ia, 16)
  Importance of the nursery (compension for missing mother?) (Ia, 16)
  Does not learn the tariffs although she is intelligent enough and meeting customers' demands is important for her.

Self-presentation:

- *Self-concept und Self-presentation*
- *Expressivität, regulation of own emotions (Rindermann, 2009)*
- *How does the person react to others' feedback?*
- *Comparison with others*

  Low scores on stableness (Deusinger, 1986) and expressivity (Rindermann, 2009), as she is desorientated she cannot show a self-concept, meeting others' expectations is of vital importance, she emphazises that she is different than the other apprentices

HOW is it said?

Distinctive feature of the narrative style – comparison with others – Is the narrative style consistent with the content? Does the person (implicitly) identify with a group?

Her narrative style shows features of a disorganized bonding style (cf. Fonagy et al., 2016), lack of memory and explanations, idealization of the father despite a distanced relationship, on the other hand the relationship to her parents seem to be of no importance – narrative style corresponds to SRS 0 Negation of Reflexive Self Functioning in Fonagy's scale, no group adherence

Qualitative Content Analysis (Mayring, 2000): theoretical categories (deductive und inductive)

- *Subjective assessment of the curriculum content*
- *Ecological factors in VET school and at the work place (Bronfenbrenner, 1979)*

Only cross-case analysis
