# Supplementary material for: Theme-centered interaction and developmental tasks as research method and pedagogical tool regarding identity development in VET
Source: Front Psychol. 2023 Oct 10;14:1201305. doi: 10.3389/fpsyg.2023.1201305 (PMC10597703; doi:10.3389/fpsyg.2023.1201305)
Supplement: Supplementary file 8 [file Data_Sheet_8.PDF]

Supplement 8 - MAXQDA-categories

THEORY/CONCEPT	DEDUCTIVE CATEGORIES	INDUCTIVE CATEGORIES	TRIANGULATION WITH
**Biographical Research**	internal contingency		
(Schütz, 1983; Nohl, 2006)	external contingency		
	action scheme		
	institutional passage		
**Reflexive project**			
Giddens (1991)	reflexive project		
**Experiential Learning**			
Dewey (1938); Kolb (1984)	experiential learning		
**Habitus**	cultural capital	migration background	
Bourdieu(2012)	economical capital	gender	
	symbolic capital	parenting style	
	social capital		
**Identity regulation**	self-concept		
Hausser (1995)	perception by others		
	emotions		EE-scale (perception of own emotions) – Rindermann (2009)
	authenticity		
	individuality-uniqueness		
	biographical continuity		
	ecological consistency cf. Bronfenbrenner below		
	self-esteem		
	meaning/goals		
	locus of control/resilience		
	motivation-interests		
**Self-regulation**			
Kaak et al. (2013)	goal orientation		
Lazarus and Folkman (1987)	coping strategies		FSAP-scale (problem-solving) – Deusinger (1986)
**Developmental tasks**	recognition		
(Havighurst, 1974)	competence		
	identification		
	shaping		
**Conflicts**	lack of recognition in role as apprentice	trial period	
Kutscha et al. 2009	dealing with mistakes	two bosses dilemma	
Duemmler et al. 2017	career choice as compromise		
	working hours		
**Identity balance**	self-presentation		FSST (stableness towards groups and others) – Deusinger (1986)
Krappmann (1969); Veith (2010)	role distance		Exp-scale (express own emotions) – Rindermann (2009)
Lind (1987)	ambiguity tolerance		ambiguity-tolerance-scale – Lind (1987)
	co-construction of mutual perspectives/		EA-scale (perception of others' emotions) – Rindermann (2009)
	empathy		FSKU-scale (sociableness) – Deusinger (1986)
			RA-scale (regulate others' emotions) – Rindermann (2009)
**Social self-concept**		External demands from	
Mead (1934)		colleagues	
Krappmann (1969)		superiors	
		customers	
		family/friends etc.	
**Moral judgment**			
Veith (2010); Kohlberg (1984);	stage of moral judgment development	closeness	moral judgment test – Lind (1985)
Lind (1985)			
**Ecology of human development**	role changes	VET school*	*detailed categories below
(Bronfenbrenner (1979)	communication among life areas	workplace	
	compatibility of life areas	health	
	mentoring		
	subjective assessment		
**Perception of VET Curriculum**	Curriculum content	redundance	
	economic role of retail	missing at VET school	
	knowledge about the employer	missing at the workplace	
	rights and duties during apprenticeship	theoretical add-on	
	merchandise management	individualization	
	protection of the environment	prior knowledge - boreout	
	publicity	not relevant for practice	
	presentation of wares	point of time	
	product expertise	revision	
	safety and health at work	learning status talk	
	teamwork and organization		
	customer orientation - selling		
	cashpoint		
