# Supplementary material for: Theme-centered interaction and developmental tasks as research method and pedagogical tool regarding identity development in VET
Source: Front Psychol. 2023 Oct 10;14:1201305. doi: 10.3389/fpsyg.2023.1201305 (PMC10597703; doi:10.3389/fpsyg.2023.1201305)
Supplement: Supplementary file 9 [file Data_Sheet_9.PDF]

**Supplement 9: Matrix for within-case and cross-case analysis**

| Codename | Sector | | Age | Identification* | Shaping+ | Recognition* | Competence* | Career choice motives | Action competence/ mistakes | role as apprentice | working hours | Ziel Prestige | Fähigkeiten | Spass | Bindung | mit Menschen | Helfen | FSKU | FSVE | FSST | FSAP | Ambiguity tolereance | ER | FR | EE | FE | Exp | Problems Conflicts | Habitus | Learning environment |
|---|---|---|---|---|---|---|---|---|---|---|---|---|---|---|---|---|---|---|---|---|---|---|---|---|---|---|---|---|---|---|
| Ciara | Tele-communication | w | 18 | +- | -- | +- | +- | advice from career counselor - satisfied with the company, not with the retail sector | afraid of making mistakes, works slowly, asks not to make mistakes, has difficulty to learn quickly, takes advantage of safe haven | is an advantage, as less pressure and goals, more support | had been protected as a minor, but now meeting friends will not be possible any more | 6 | 3 | 5 | 2 | 4 | 1 | 1 | 5 | 2 | 6 | 14 | 33 | 36 | 19 | 37 | 14 | young, shy, unexperience, discrepancy self-concept- others' perception, no initiative to acquire product expertise, more active after criticism | grown up without mother, fater (migrant) was single parent, craftsmen | good support and low pressure at workplace |
| Ahmet | Filling station | m | 24 | ++ | -+ | -- | +- | least of few evils but achievement for welfare recipient | not afraid (mistakes are normal in apprenticeship, opportunity to learn), but afraid of trial period, | not possible to address nuisance because of imbalance of power and trial period | stressing shift changes- sleep disorder - stand in for sick colleagues, in general good working hours | 6 | 3 | 1 | 4 | 2 | 5 | 96 | 62 | 91 | 36 | 5 | 31 | 28 | 24 | 26 | 30 | only unskilled work, still in trial period, salary is paid too late | no contact with father (migrant), mother single parent and ill, welfare beneficiary | no support, a lot of criticism |
| Markus | Automotive equipment | m | 19 | | | | | third choice after automobile salesman, IT-system-manager, interested in technical advice, unsatisfied with the company | prefers not to take risks, checks several times to avoid mistakes | employer does not stick to the workplace curriculum - gives no time for writing reports | works himself too much, does not mind working overtime | 6 | 1 | 3 | 5 | 4 | 2 | **98** | 75 | **95** | **98** | 11 | **38** | **38** | **38** | **39** | **34** | critizised for bad sales activity due to private problems, no feedback, has moved up to top of the race list | grown up in a social flashpoint | bad assessment of the company on online platforms |
| Jennifer | Consumer electronics | w | 20 | | | | | third choice after carpenting and visual marketing, sociable, able to adjust | thinks permanently of making mistakes, is critizised in case of mistakes | customers often expect too much, missing authorizations impede work process | adjustment difficulties to the new rhythm, working hours incl. long ways to work are tiring , meeting friends is difficult | 3 | 2 | 1 | 1 | 1 | 1 | 97 | **5** | 53 | 56 | 24 | 27 | 38 | 20 | 38 | **39** | customers expect her to know everything immediately - inner distance | divorced parents | good support at workplace |
| Lara | Consumer electronics | w | 21 | | | | | third choice after media designer, IT-merchant, interested in techn. expertise, continues to look for alternatives | not afraid because of prior knowledge, closes knowledge gaps actively, mistakes are normal in the first year | feels as the last link in the chain, has to obey to orders, sometimes from different people | start of school is too early in case of late shift, difficulties to meet boy-friend | 6 | 3 | 2 | 1 | 5 | 4 | 92 | **33** | 49 | 89 | 7 | 23 | 23 | **14** | 29 | 24 | customers dissatisfied because of unrealistic expectations concerning prices, women are not respected in a technical domain | bullying at school, loner, obese | good support at workplace |
| Nils | Food/ Non-Food | m | 20 | | | | | interested in commerce because of his parents | perfectionist, asks too much of himself, initially high demands -> anxious, works more quickly | hard to come out as an apprentice in front of customers | difficulties to meet friends on Fridays | 6 | 4 | 5 | 3 | 2 | 1 | 99 | 75 | 77 | 83 | -6 | 34 | 33 | 29 | 30 | 23 | time pressure/high expectations from colleagues (inner distance)-> initially anxious | long periods of illness, orderly family | no communication with management, friendly support from colleagues |
| Bastian | Tele-communication | m | 21 | ++ | ++ | +- | +- | good vocation, sociableness, second choice because of missing A-levels | makes mistakes, accepts criticism, tries to improve, conflicts with the shop leader, as he cannot meet demands, refers to his role as apprentice | Déjà-vu with parental conflicts, colleagues are older | good working hours - but start of school too early | 4 | 1 | 2 | 5 | 3 | 6 | 96 | 82 | 71 | 97 | 16 | 31 | 35 | 23 | 31 | 31 | conflicts with parents, bullying at school, shop leader and customers have excessive, unrealistic demands | father authoritarian (middle class), mother ill, lives on his own, no girl-friend but a good mate | good support at workplace |
| Jasper | Filling station | m | 23 | -- | - | -- | +- | last choice, interested in real estate, problematic child | afraid of mistakes in case of stress - has not yet happened | sufficient scope of action, no excessive demands, is autonomous | has to get up at 3 am - gets a day off if he asks in advance | 1 | 2 | 3 | 5 | 6 | 4 | 23 | 49 | 88 | 93 | -3 | 36 | 26 | 28 | 19 | 18 | school absenteeism, bullying, always on his own, problematic child | parents divorced, has been living abroad with his father, has a 2-year-old daughter, lives with his mother, intimate relationship with his grandfather | scope of action, but no skilled work |
| Berat | Filling station | m | 18 | | | | | no interest in vocation, strives for the final qualification certificate | afraid of trial period, has made few mistakes, sometimes superiors' expectations are demanding, complies to avoid conflicts | Shift leader demands too much, has difficulty to meet them | adjustment difficulties to shift work - late shift in conflict with early start of school, could be improved after asking the superior | 6 | 5 | 3 | 4 | 2 | 1 | **99** | 28 | **92** | 64 | -1 | 34 | 37 | 32 | 30 | 23 | superior gives too many tasks, complies to avoid conflict | grown up in a social flashpoint, distances himself clearly, aims for education as a means for social advancement | superior supports school attendance |
| Alina | Tele-communication | w | 27 | ++ | +- | ++ | ++ | university drop-out, positive assessment of good conditions in the company and sociableness | no difficulties | dropping out of university is a step back, apprenticeship is like a honeymoon period | used to such working hours from previous jobs | 6 | 4 | 5 | 1 | 2 | 3 | 96 | 62 | 88 | 93 | 11 | 33 | 35 | 32 | 39 | 29 | older than experienced colleagues | orderly middle-class family, university drop-out, good family cohesion, living with his boy-friend | good support at workplace |
| Hendrik | Food | m | 18 | | | | | last chance as marks were not good enough (absence because of illness), unsatisfied with the company | cannot meet all demands (time pressure, no mistakes), superior's fault | some colleagues devalue apprentices, expectations are too demanding (no protection for apprentices) | difficult to reconcile activity at fire brigade with late shift - no leisure time | 1 | 1 | 1 | 1 | 1 | 1 | **100** | **100** | **92** | **100** | 1 | **35** | **38** | **40** | 35 | 23 | time pressure - no support from VET school concerning absence due to illness, afraid of mistakes | living with his girl-friend - financially independent suspicion of traumatization in early childhood | time pressure, high demands, no tolerance for mistakes |
| Kostas | Jewelry | m | 19 | | | | | had to give up apprenticeship as electrician for health reasons, last chance, sociable | sometimes forgets something, he thinks it is human, but colleagues do not tolerate | Feels like a cleaning woman, underestimated by his superior, sometimes recognized | no leisure time, needs more relaxation than before | 4 | 5 | 3 | 2 | 3 | 1 | 76 | **14** | 41 | **6** | **-13** | 21 | 27 | **17** | 30 | **16** | superior does not appreciate his competence, allegations not justified | Christian (altruistic), political engagement, parents were always short of money, intimate relations with relatives, social flashpoint | regular disputes with the superior |
| Sophie | Jewelry | w | 18 | | | | | Interested in costumers and product expertise, likes vocation but too boring in the long run | receives support in case of mistakes | coming out as apprentice in front of customers prevents excessive demands, accepted as a novice | workforce planning difficult, no regular participation in sports, leisure time is needed for writing reports/preparing exams, no real leisure time | 5 | 1 | 2 | 6 | 4 | 3 | 92 | 52 | 57 | 64 | 12 | 28 | 32 | 26 | 37 | 25 | reports delivered too late due to missing leisure time, has been tolerated | orderly middle-class family | appreciating, supporting colleagues |
| Hauke | Consumer electronics | m | 26 | ++ | -- | ++ | ++ | second choice after apprenticeship as a sound engineer, girl-friends' advice, sociable, technical expertise | tries to avoid mistakes, mistakes are normal | feels like a step back, apprenticeship less recognized than studies at university | no problem as used to worse conditions in previous jobs | 5 | 2 | 4 | 1 | 4 | 3 | 92 | 29 | 49 | 72 | 4 | 34 | 38 | 28 | 34 | 17 | afraid of selling and phone calls, avoids these situations | parents workers, grown up in Eastern Germany, nestling, lives independently with his girl-friend, probable traumatization in early childhood | helpful mentors, always available |
| | | 9 | male | | | | | | | | | **65** | **37** | **40** | **41** | **43** | **36** | **83** | **48** | **68** | **68** | **5,9** | **31** | **33** | **26** | **32** | **25** | | | |

*detailed statements in Thole, 2021, pp. 443ff.
